# The advertisement calls of Brazilian anurans: Historical review, current knowledge and future directions

**DOI:** 10.1371/journal.pone.0191691

**Published:** 2018-01-30

**Authors:** Vinicius Guerra, Diego Llusia, Priscilla Guedes Gambale, Alessandro Ribeiro de Morais, Rafael Márquez, Rogério Pereira Bastos

**Affiliations:** 1 Programa de Pós-Graduação em Ecologia de Ambientes Aquáticos Continentais (PEA), Universidade Estadual de Maringá (UEM), CEP: 87020-900, Maringá, Paraná, Brazil; 2 Laboratório de Herpetologia e Comportamento Animal, Departamento de Ecologia, Instituto de Ciências Biológicas, Universidade Federal de Goiás, Campus Samambaia, CEP 74001-970, Goiânia, Goiás, Brazil; 3 Terrestrial Ecology Group (TEG), Departamento de Ecología, Universidad Autónoma de Madrid, C/ Darwin, 2, Ciudad Universitaria de Cantoblanco, Facultad de Ciencias, Edificio de Biología, Madrid, Spain; 4 Universidade Estadual do Mato Grosso do Sul, R. General Mendes de Moraes, Jardim Aeroporto, CEP 79804-970, Coxim, Mato Grosso do Sul, Brazil; 5 Instituto Federal Goiano, Departamento de Ciências Biológicas, CEP 75901-970, Rio Verde, Goiás, Brazil; 6 Fonoteca Zoológica, Departamento de Biodiversidad y Biología Evolutiva Museo Nacional de Ciencias Naturales (CSIC), C/ José Gutiérrez Abascal 2, Madrid, Spain; Museum National d'Histoire Naturelle, FRANCE

## Abstract

Advertisement calls are often used as essential basic information in studies of animal behaviour, ecology, evolution, conservation, taxonomy or biodiversity inventories. Yet the description of this type of acoustic signals is far to be completed, especially in tropical regions, and is frequently non-standardized or limited in information, restricting the application of bioacoustics in science. Here we conducted a scientometric review of the described adverstisement calls of anuran species of Brazil, the world richest territory in anurans, to evaluate the amount, standard and trends of the knowledge on this key life-history trait and to identify gaps and directions for future research strategies. Based on our review, 607 studies have been published between 1960 to 2016 describing the calls of 719 Brazilian anuran species (68.8% of all species), a publication rate of 10.6 descriptions per year. From each of these studies, thirty-one variables were recorded and examined with descriptive and inferential statistics. In spite of an exponential rise over the last six decades in the number of studies, described calls, and quantity of published metadata, as revealed by regression models, clear shortfalls were identified with regard to anuran families, biomes, and categories of threat. More than 55% of these species belong to the two richest families, Hylidae or Leptodactylidae. The lowest percentage of species with described calls corresponds to the most diverse biomes, namely Atlantic Forest (65.1%) and Amazon (71.5%), and to the IUCN categories of threat (56.8%), relative to the less-than-threatened categories (74.3%). Moreover, only 52.3% of the species have some of its calls deposited in the main scientific sound collections. Our findings evidence remarkable knowledge gaps on advertisement calls of Brazilian anuran species, emphasizing the need of further efforts in standardizing and increasing the description of anuran calls for their application in studies of the behaviour, ecology, biogeography or taxonomy of the species.

## Introduction

Although the role of natural history in current science is subjected to a long debate [1–3], hypothesis testing, experimental design, observational studies or modelling are strongly based on information of the general biology of living beings [4–6]. Basic knowledge about the species traits, such as those involved in behaviour, ecology or distribution, are key to assist multiple research disciplines. The lack of knowledge on the traits ecologically relevant for the species is known as Raunkiaeran shortfall [7,8]. This shortfall can be related to the ecological functions played by each trait, to the trait variation both within and between species, and to the interaction among traits acting together to perform specific ecosystem functions [8,9]. This lack is further aggravated by the gap of knowledge about the taxonomic diversity of species and the accelerated rate of habitat loss and species extinction [10–12].

In adult anuran amphibians, acoustic signals are the predominant form of communication of almost all species, being a key life-history trait mainly associated with sexual selection and reproduction [13,14]. Frog calls may have different functions and are used to attract mating patterns, in disputes over territory and in other aggressive interactions, and thus acting in social organization [15,16]. Emerging early in the evolutionary history of Anura and by their crucial role in the recognition and discrimination of conspecific individuals, the vocalizations can reflect evolutionary relations among taxa [13]. Moreover, acoustic signals are subject to changes through the influence of biotic and abiotic factors [17], resulting in inter-individual and inter-populational differences [18,19].

Thereby, bioacoustic information has been particularly relevant in studies of behaviour [17], ecology [20], evolution [21,22], conservation [23], thermal biology in light of climate change [24], biodiversity inventory (passive acoustic monitoring; [25]), and is becoming a suitable tool for studies on taxonomy (integrative taxonomy; see [14,26]). Since call is a particularly powerful premating isolation mechanism, it has been used as an undisputed means for species delimitation [14]. Comparative bioacoustical analyses have also resulted in the discovery of many morphologically cryptic anuran species [26–28].

The advertisement call can also be considered as a crucial functional trait of most anuran amphibians. Functional traits are any phenotypic attribute that affects a species’ individual fitness and population dynamics and/or their influence on other organisms and ecosystem functions [8,29]. Besides the advertisement call being an honest indication of position, body size, body temperature or energetic condition of the calling male [13], it incorporates characteristics for species recognition, serving as an essential criterion in female choice [13,14], although acoustic signals can also attract the attention of predators [30,31]. Moreover, species may compete for calling sites or acoustic space during breeding season, leading to competitive exclusion [32] or changes in acoustic parameters of their advertisement calls [33]. Thus, such acoustic signals may be a prominent trait in studies on community ecology and functional diversity.

The variety of applications of bioacoustics relies on the scientific knowledge of the signals, and basically, as a first step, on the quantitative and qualitative descriptions of the species calls. Yet this task is far to be completed, especially in tropical regions, which hold most of the planet’s biodiversity [10–12]. Moreover, call descriptions are often limited in information and usually restricted to comparisons between closely related taxa [34]. In this sense, metadata related to environmental conditions, behavioural context and/or methodological procedures can be succinct or absent in many studies of call descriptions [14,35]. Only recently a comprehensive guideline for the description of anuran calls has been published [14].

Brazil is the country with the richest diversity of anuran amphibians in the world [36]. Brazilian anurans are distributed throughout various biomes and may also be found in transition areas [37,38], representing different climate types and a characteristic fauna of each region. Brazil also houses a high endemism rate of amphibians (for example, in the Cerrado biome the endemism rate is around 51.7%; [37]). The wide variety of habitats and the changes of the climate throughout evolutionary time-scale were likely responsible for the generation such a high diversity of endemic species [39,40]. Nevertheless, many taxonomic problems associated with sympatric and cryptic species still remain, especially in larger taxa, such as the *Terrarana* species [41], being necessary to increase the knowledge in biology, distribution and phylogenetic relationships of these Neotropical anurans.

Examining the amount, standard and trends of the study of species traits allows to identify knowledge gaps and to guide future research strategies. In this study, we used a quantitative analysis of research literature (scientometric review; [42]) to understand the state of the art of the call description of Brazilian anuran species, addressing a series of essential aspects: (i) historical revision of the description of species calls in the last six decades, (ii) number and percentage of species with described calls across families, biomes, and categories of threat, (iii) available metadata in the call descriptions, and (iv) publication of descriptions in relation to journals, number of authors and countries. In addition, we searched for voucher recordings of each taxon deposited in scientific sound archives, and compared the description of anuran calls with the original description of the species, concluding with general directions for future studies.

## Materials and methods

### Bibliographical survey

Scientific literature about the description of anuran calls of Brazil was search in the databases of published articles of the Institute for Scientific Information (ISI; www.isiknowledge.com), Scientific Electronic Library online (Scielo; www.scielo.org), Google Scholar (www.scholar.google.com), and Amphibiaweb (http://www.amphibiaweb.org/). For this survey, we considered only anuran species occurring, exclusively or not, within the territory of the Brazilian federation and which were described until December 2016. These species were determined based on the list of amphibians from Brazil provided by Segalla et al. [36], completed with recent species descriptions published in the site Amphibian Species of the World [43]. As a result, 1,045 Brazilian anuran species were identified and used for further analysis. Among them, three species classified as "Incertae sedis" (*Eleutherodactylus bilineatus*, *Hyla imitator*, and *Calamita melanorabdotus*) were kept since they are on the list of the Brazilian Society of Herpetology [36]. The species names and year of species description were registered according to the taxonomic classification proposed by Frost [43]. The Brazilian biomes where each taxon occurs were also recorded using the species distribution range which was based on the manuscript of the species description, on the calls description and according to Frost [43]. Moreover, the threatened category of each species was obtained from the IUCN Red List of Threatened Species [44] and the Brazil Red Book of Threatened Species of Fauna (BRB) [45]. We considered both threatened categories because they are evaluated differently. While the IUCN list is developed to be applied on a global scale, the BRB list is applied on a smaller scale, giving a complementary picture about the species' risk of extinction [46,47]. Accordingly, significant differences between these two lists in the categorization of several amphibian species were found [48].

The bibliographical review was focused on the description of advertisement calls since this call type is the most prevalent, biologically relevant and easiest to record acoustic signal in anuran species [14,16]. In the search for articles, two sets of terms were used. The first set encompassed the combination of the specific name of each taxon and all their synonyms (for species with taxonomic revisions). The second set included the terms *advertisement call*, *call*, *vocalization*, *canto*, and *vocalização*. Scientific articles containing at least one term of each set, and published before 2017, were retrieved and added to the study database. Additionally, we searched for references cited by collected studies that were not recovered by the bibliographic search, which included articles not available from on-line databases. Only works that presented numerical data for the acoustic parameters of the calls (e.g., temporal and spectral parameters) were considered. Grey literature (theses and conference abstracts) and sound guides (CD Audio) were not included, as well as many papers, particularly older, having subjective descriptions of the calls (e.g., [49,50]). No other filter was used during the search.

In addition, six classical textbooks reviewing natural history of anurans [51–56] were also examined in order to complete the review of the call descriptions. We also searched for recordings of each taxon in the three largest scientific animal sound collections (Fonoteca Zoológica of the National Museum of Natural Sciences, Madrid ─ http://www.fonozoo.com/; Maculay Library of the Cornell Lab of Ornithology, New York ─ https://www.macaulaylibrary.org; and Fonoteca Neotropical Jacques Vielliard of the Campinas State University, São Paulo ─ www.2.ib.unicamp.br/fnjv/). The data were collected from July 2016 through January 2017.

### Data analysis

From each retrieved study, the following variables were obtained: (i) species name, (ii) year of publication, (iii) first author name, (iv) institution of the first author, (v) number of authors, (vi) journal name, and (vii) biome of ocurrence. We also registered the presence or absence of specific metadata associated with the sound recordings: (i) locality, (ii) date, (iii) water temperature, (iv) air temperature, (v) relative humidity, (vi) activity period, (vii) habitat, (viii) perch height, (ix) distance to the nearest calling male, (x) number of recorded males, (xi) recorder, (xii) software, (xiii) microphone distance, (xiv) voucher specimen, (xv) voucher recording, (xvi) sonogram, (xvii) oscillogram, (xviii) power spectrum, (xix) call duration, (xx) note duration, (xxi) pulse number, (xxii) call rate, (xxiii) call frequency, (xxiv) sound pressure level, and (xxv) harmonics presence (see S1 Text for the specific description of these variables).

Firstly, descriptive statistics of single variables or a combination of variables were calculated from this database. Secondly, to evaluate the annual progress in the descriptions of anuran calls, Generalized Linear Models with Poisson error structure and log link function [57] were used. Specifically, three GLMs were fitted to model the relationship between the year of publication and a series of response variables, such as (i) the annual number of described calls, (ii) the number of metadata variables provided per study, and (iii) the number of authors per study. Overall statistical assumptions were carefully checked for each of the three models. Leverage values as well as DFBeta values indicated no obviously influential cases [58,59]. Overdispersion was only identified in the first model (dispersion parameter = 5.3, chi-2 = 289.9, df = 55, p<0.001), but not in the other models (dispersion parameter = 0.8, chi-2 = 1182.2, df = 1401, p = 0.9; dispersion parameter = 0.7, chi-2 = 491.4, df = 717, p = 1, respectively). To correct for overdispersion, estimated regression coefficients of the first GLM were multiplied with the square root of the overdispersion parameter and then recalculated the statistics and the p-value. All models were fitted in R (version 3.2.1, [60]) using the function *glm*. We assume a criterion of significance of p ≤0.05.

## Results

### Historical perspective

**Fig 1:**
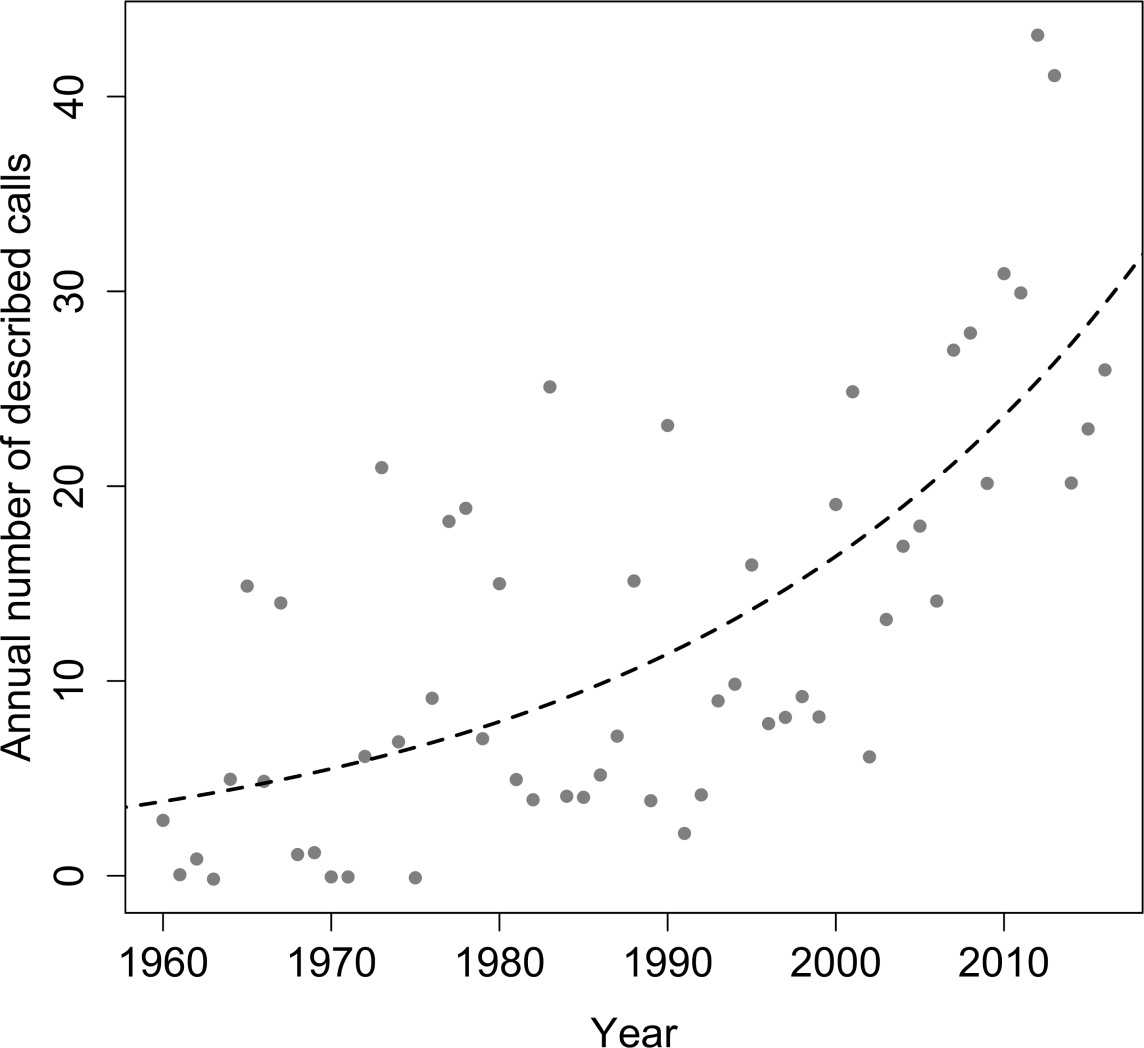
Evolution in the descriptions of the advertisement calls of Brazilian anurans between 1960 and 2016. Regression function and line fitting the annual number of new described calls over the last six decades.

Based on our literature review, a total of 607 studies including descriptions of advertisement calls of Brazilian anuran species have been published between 1960 and 2016 (S1 Table and S2 Text), resulting in a publication rate of 10.6 descriptions per year. The first description was for the calls of three species of the family Leptodactylidae (*Leptodactylus bolivianus*, *L*. *mystacinus* and *L*. *pentadactylus*), published in 1960 in the Texas Journal of Science [61]. Since then, the annual number of call descriptions has increased with an exponential progress (estimate±SE = 0.036±0.006, z = 14.5, p<0.001, df = 55), as shown in Fig 1. The year 2012 had the greatest number of calls described (43 calls), followed by 2013 (41 calls), 2010 (31 calls), and 2011 (30 calls; see S1 Table). On the other hand, the first description of a Brazilian anuran species that also included the call description was for the species *Scinax berthae*, published in 1962 in the journal Physis [62]. According to our dataset, the description of the species calls that did not have the call described in the original description occurred on average 60.17 ± 56.72 (0─238) years after the species was described.

### Described calls

**Fig 2:**
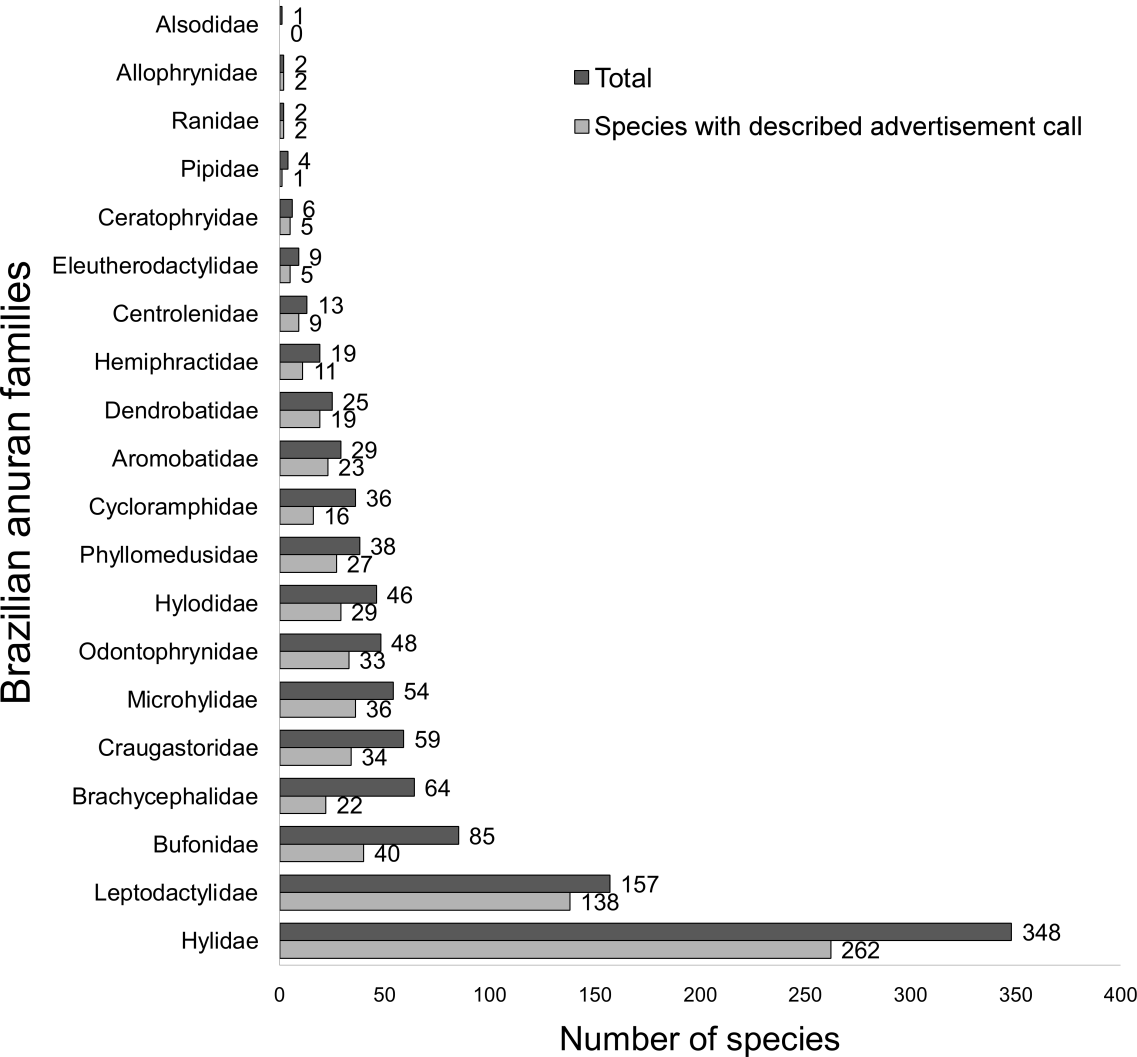
Total number of species with described advertisement call from each of the Brazilian anuran families.

During the last six decades, 719 Brazilian anuran species have had their advertisement call described (68.8% of the total, i.e., 1,045 species). More than half of them corresponded to species of the two largest anuran families in Brazil (Fig 2): 262 species to the family Hylidae (75.3% of its species) and 138 species to the family Leptodactylidae (88.0% of its species). Some families, with considerably less number of species, also show more than 75% of their species with described calls, namely, Aromobatidae (79.3%), Dendrobatidae (76.0%), Ceratophrynidae (83.3%), Ranidae (100%), and Allophrynidae (100%). On the contrary, the families Brachycephalidae (34.4%), Bufonidae (47.1%), Cycloramphidae (44.4%) and Pipidae (25.0%) have less than half of their species with described calls (Fig 2). The species *Limnomedusa macroglossa* (single representative of the Alsodidae family) does not have the call described. A large proportion of the species (302 species, 42.0% of all species with descriptions) have two or more published descriptions of their calls. As a result, a total of 1,403 descriptions have been published between 1960 and 2016. The species with greatest number of call descriptions are *Dendropsophus minutus* (15 descriptions), *Leptodactylus fuscus* (14 descriptions), and *L*. *pentadactylus* (13 descriptions; S1 Table).

### Metadata

**Fig 3:**
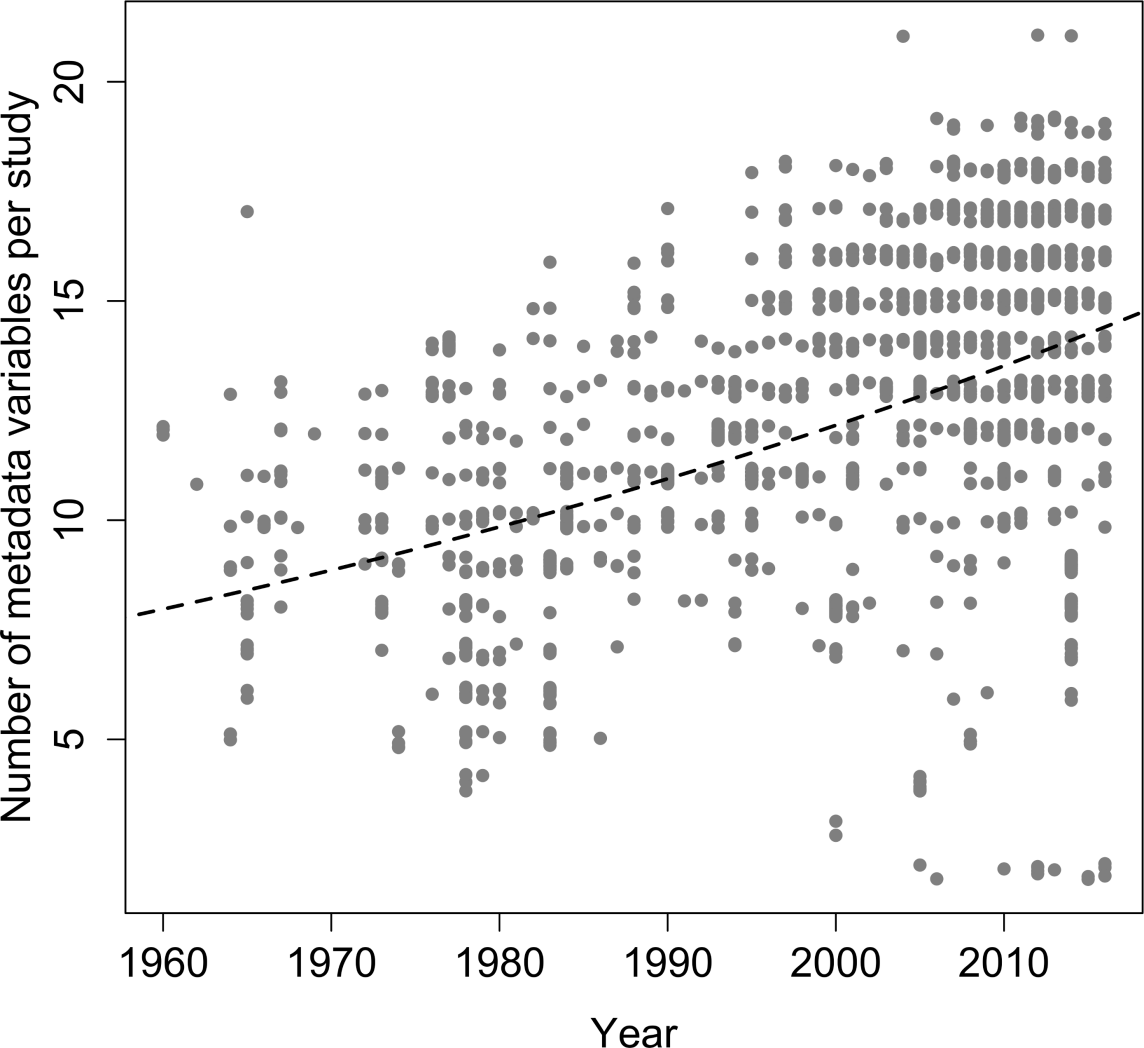
Evolution in the presence of metadata in the studies with descriptions of advertisement calls of Brazilian anurans between 1960 and 2016. Regression function and line fitting the number of metadata variables provided in each study and the year in which was published.

The presence of metadata associated with the call description is highly variable across studies. However, the number of metadata variables included in the description tended to increase over the time (estimate±SE = 0.011±0.001, z = 17.8, p<0.001, df = 1401; Fig 3). None of the reviewed studies presented all variables considered in the analysis. The most common information included in the descriptions were: call frequency (97.7%), call sonogram (90.0%), study locality (88.5%), call duration (81.9%), analysis software (81.0%), and note duration (75.7%; Table 1). Information of perch height, number of recorded males, voucher recording, power spectrum, call rate and presence of harmonics were showed in between 10 and 50% of the studies. Information of water temperature, relative humidity, distance to the nearest calling male, microphone distance, and sound pressure level were showed in less than 10% of the studies. Only a total of 547 Brazilian anuran species (52.3% of all species; 76.1% of species with described calls) have some of its calls deposited as voucher recordings in the scientific animal sound collections analysed (S1 Table). The Fonoteca Neotropical Jacques Vielliard (FNJV) included the greatest number of species with voucher recordings (445 species; 61.9% of the species with described calls). A small portion of species (59 species; 5.6% of all Brazilian anurans) has recordings deposited in the collections although their calls have not been described yet (S1 Table). Moreover, only 214 original descriptions of Brazilian anurans (20.5% of all species; 29.8% of species with described calls) included information about call parameters of the described species.

**Table 1:** Metadata variables shown in the 1,403 descriptions of advertisement calls of Brazilian anuran species.

Information	Number of descriptions	%
Locality	1242	88.52
Date	846	60.30
Water temperature	123	8.77
Air temperature	755	53.60
Relative humidity	64	4.56
Activity period	752	53.60
Habitat	895	63.79
Perch height	488	34.78
Distance to the nearest calling male	49	3.49
Number of recorded males	669	47.68
Recorder	995	70.92
Software	1136	80.97
Microphone distance	133	9.48
Voucher specimen	894	63.72
Voucher recording	335	23.88
Sonogram	1262	89.95
Oscillogram	941	67.07
Power spectrum	182	12.97
Call duration	1136	80.97
Note duration	1062	75.69
Pulse number	850	60.58
Call rate	621	44.26
Call frequency	1371	97.72
Sound Pressure Level	28	2.00
Harmonics presence	443	31.58

### Call descriptions per biome

**Fig 4:**
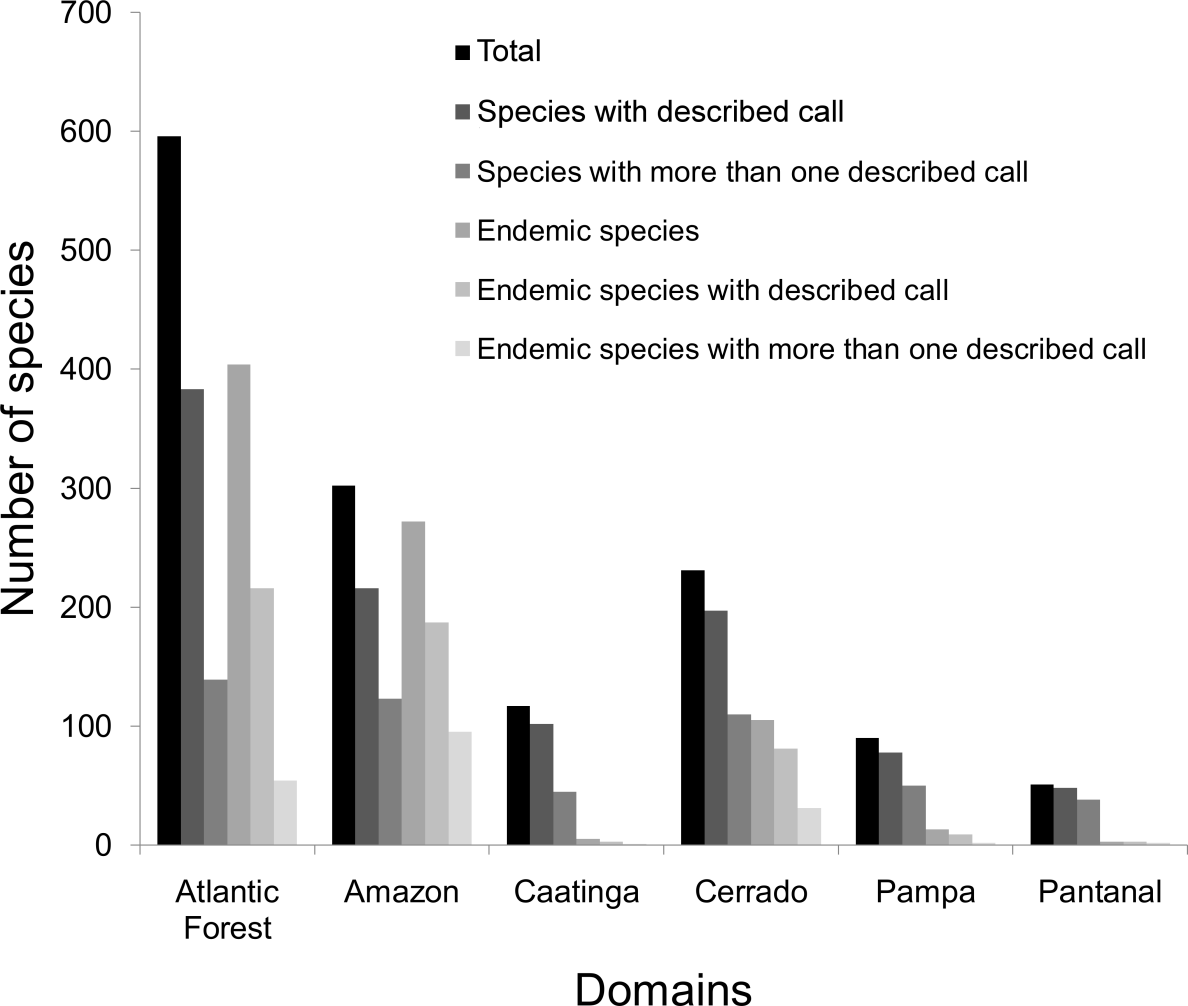
Number of anuran species (overall and endemic) in each Brazilian biome with one or more published descriptions of their advertisement call.

Among the six biomes presented in Brazil, the Atlantic Forest has the highest number of anuran species, followed by the Amazon and the Cerrado (Fig 4, S2 Table), whereas the level of endemism is maximum in the Amazon (90.1%), followed by the Atlantic Forest (67.2%) and the Cerrado (44.5%). Overall, biomes with higher diversity of amphibian anurans have lower percentage of species with their adverstisement calls described, namely, Atlantic Forest (65.1%), Amazon (71.5%), Cerrado (83.5%), Caatinga (87.2%), Pampa (83.9%), and Pantanal (90.6%). Among the three most diverse biomes, the highest percentage of endemic species with described calls corresponded to the Cerrado (77.1%), followed by the Amazon (68.7%) and the Atlantic Forest (54.9%), being more than 50% for all biomes. On the other hand, the proportion of species with calls described more than once ranges from more than 50% in the Pantanal to 13.4% in the Atlantic Forest (Fig 4).

### Call descriptions per categories of threat

**Fig 5:**
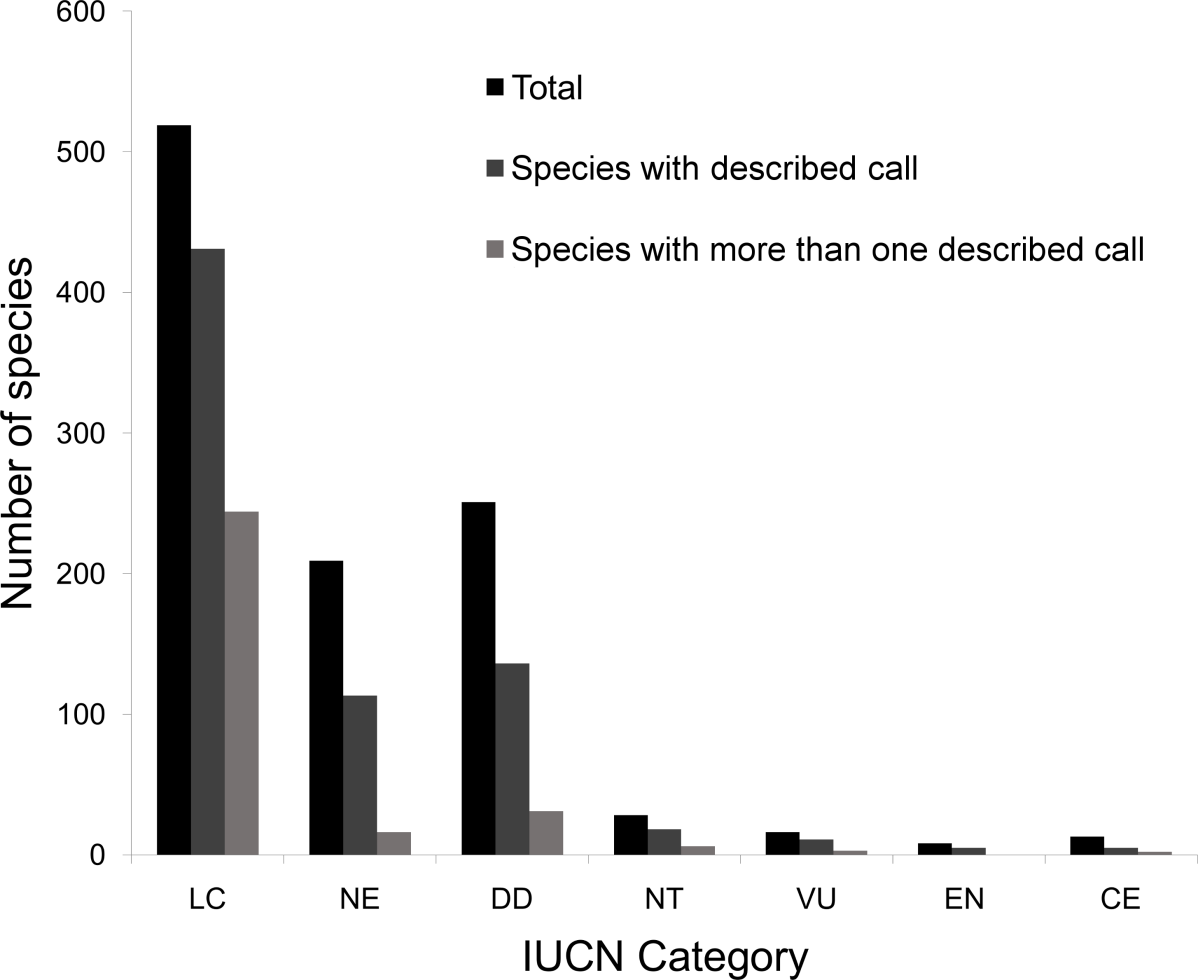
Number of Brazilian anuran species in each of the categories of threat of the IUCN red list with one or more published descriptions of their advertisement call.

Within the IUCN red list, 56.8% of the species included in threatened categories (critically endangered ─ CR, endangered ─ EN, and vulnerable ─ VU) have their advertisement call described, whereas 82.1% of the species in less-than-threatened categories (least concern ─ LC and near threatened ─ NT). The category CR (38.5%) has the lowest percentage of species with their calls described, while the remaining IUCN categories (including data deficient species ─ DD and not evaluated ─ NE) reach more than 54% (Fig 5). The call of the only extinct species (*Phyllomedusa fimbriata*) remains unknown (S1 Table). Concerning the Brazil Red Book of Threatened Species of Fauna, among the 46 species that are included in some threatened category (CR, EN and VU), 41.3% of them (19 species) do not yet have their advertisement call described (S1 Table). Only five threatened species included in the BRB (*Allobates goianus*, *Boana semiguttatus*, *Scinax duartei*, *Physalaemus maximus*, and *Proceratophrys moratoi*) have their calls described in more than one study.

### Publication of call descriptions

**Fig 6:**
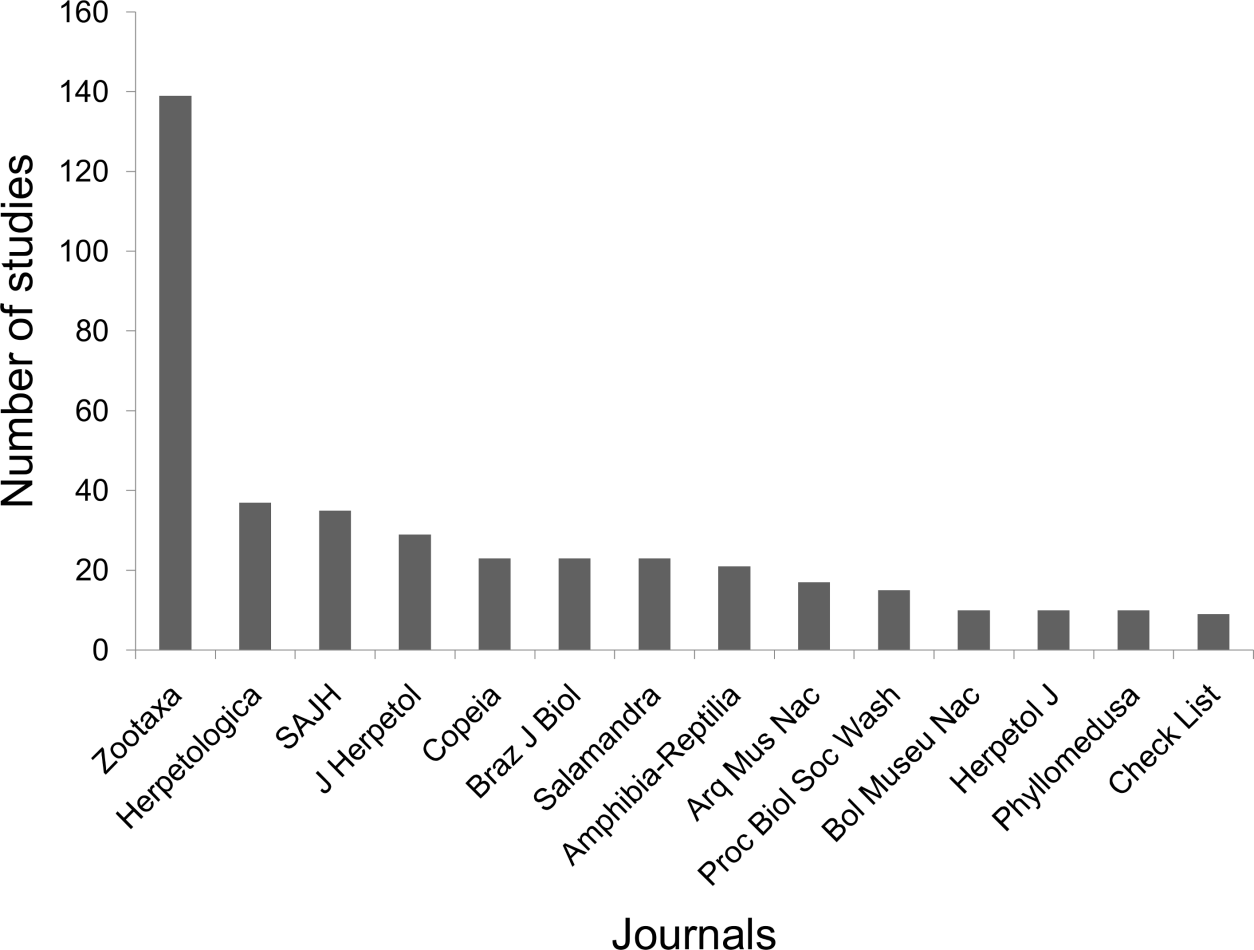
Scientific journals with publications about descriptions of advertisement calls of Brazilian anurans published between 1960 and 2016.

**Fig 7:**
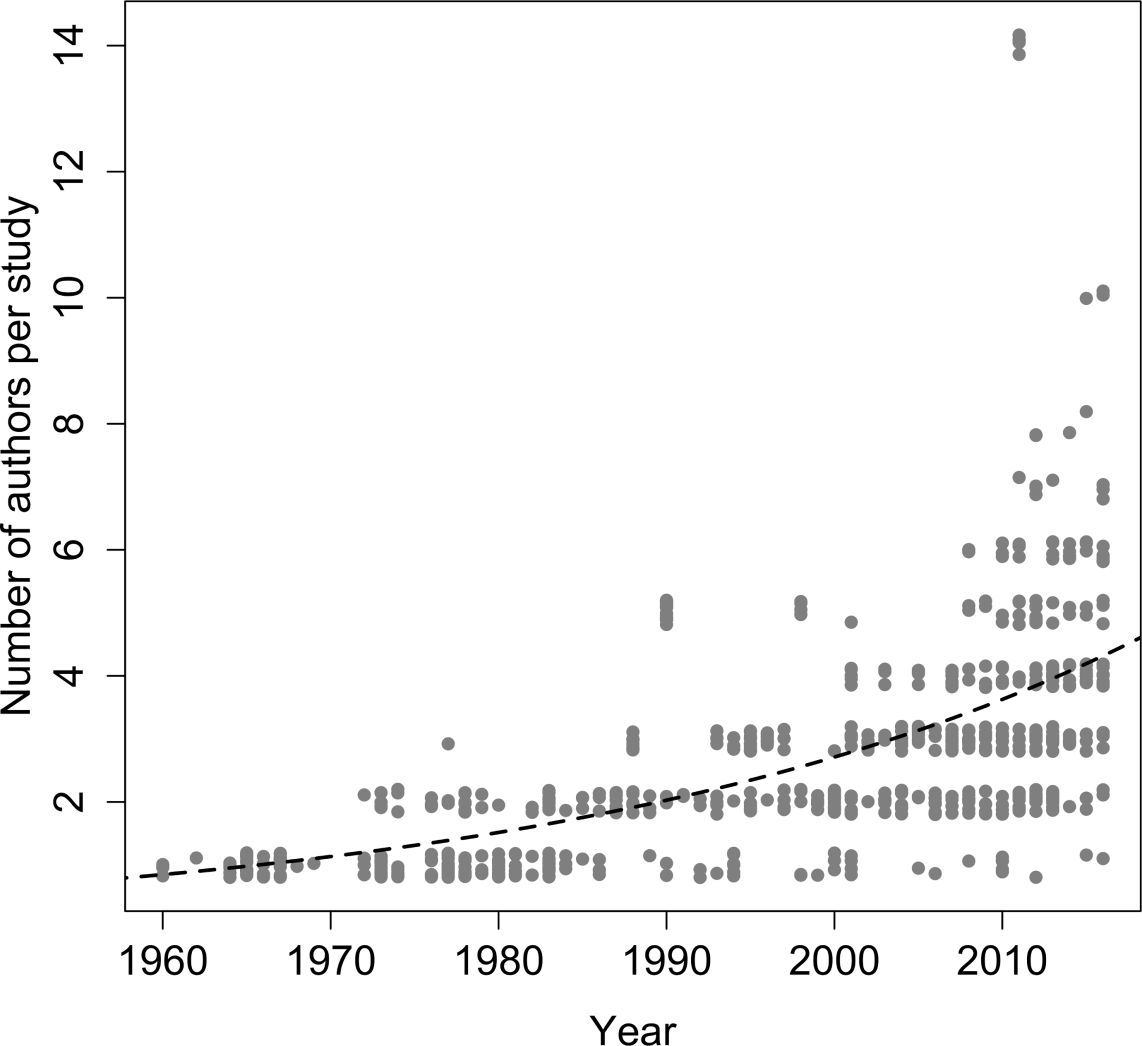
Evolution in the number of authors of studies with descriptions of advertisement calls of Brazilian anurans between 1960 and 2016. Regression function and line fitting the number of authors of each study and the year of publication.

**Fig 8:**
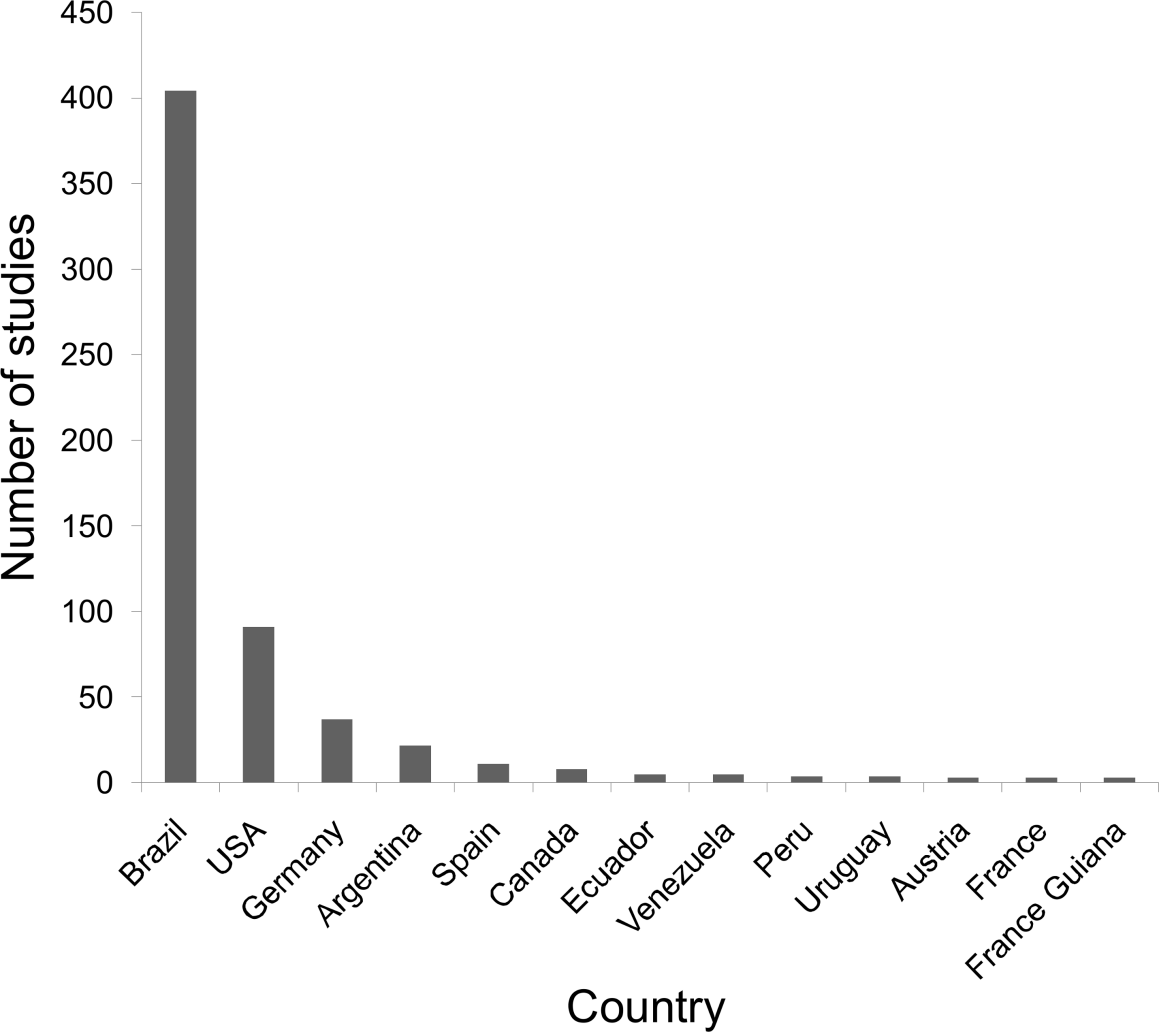
Number of studies with descriptions of advertisement calls of Brazilian anurans published per country (affiliated institution of the first author).

The advertisement calls of Brazilian anurans have been reported in a series of 99 journals and books. The journals with the largest number of publications are Zootaxa, followed by Herpetologica, Brazilian Journal of Biology (previously "Revista Brasileira de Biologia"), South American Journal of Herpetology, Journal of Herpetology, and Copeia (Fig 6). From the 607 studies including descriptions of advertisement calls published so far, 88 were single-authored (14.5%), 199 published by two authors (32.8%), 168 by three authors (27.7%), 73 by four authors (12.0%), and the remainder by five or more authors (13.0%). Over time, the number of authors per study has also increased significantly (estimate±SE = 0.029±0.002, z = 16.3, p<0.001, df = 717; Fig 7). As expected, first authors of most of the articles were affiliated with Brazilian institutions (403 studies, 66.4% of the total; Fig 8). The Universidade Estadual Paulista (9.1%), Universidade Federal do Rio de Janeiro (8.6%), Universidade de São Paulo (8.1%), Universidade Federal de Goiás (3.6%), and Universidade Federal de Uberlândia (3.6%) contributed with the greater proportion of studies. On the other hand, large number of studies described the calls of more than one species, highlighting the works of Lescure & Marty [54], with 72 call descriptions, Duellman [56], with 50, De Sá et al. [63], with 44, and Heyer et al. [53], with 36.

## Discussion

### General perspective

Despite of intense efforts to characterize advertisement calls of Brazilian anurans during the last six decades, which have resulted in the publication of 607 studies and 1,403 call descriptions, the bibliographical review indicated a remarkable gap in the knowledge of this key functional trait (only 68.8% of the described species have their advertisement call known). Moreover, significant biases in the current understanding of the advertisement calls were identified across families, biomes, and categories of threat. This lack of information about the natural history of the species may compromise the amount, quality, and types of research accomplish in this group, such as studies on behaviour [17,20], ecology [21], conservation [23], thermal biology [24] or the evolutionary relationships among taxa [26–28], since calls may contain a phylogenetic signal [22].

Natural history information is important not only to known the biology of species and communities, but also to identify cryptic species or to prioritize conservation efforts over taxa that could be more vulnerable than others [1,8]. Since sophisticated technological tools allow us to better examine the patterns of nature and to make more accurate predictions of the responses of organisms to potential futures, it seems that fewer researchers have devoted to studing natural history [1]. Fieldwork is laborious, costly, and time-consuming; besides, journals that published works on natural history show generally low scientific impact in official journal rankings, been restricted to specific areas of biology. The risk that new generations of biologists disregard the importance of the natural history as an relevant component of their professional training or their scientific careers [3], may complicate the reduction of knowledge gaps on basic functional traits of the species and also deteriorate research in general, since natural history can contribute to all phases of research, from the development of hypotheses and concepts to the interpretation and discussion of results.

Nevertheless, an exponential growing in the annual number of published call descriptions was observed in the period 1960–2016. On average, more than 10 descriptions of advertisement calls of Brazilian anurans were reported every year, with a rate above 30–40 descriptions per year in the last decade. The emergent interest in bioacoustics [23] and the opportunities provided by the expansion of audio digital recording and other technologies [64] are likely responsible for this particular pattern, opposite to that observed in other areas of natural history. Therefore, the growing attention paied to the anuran calls enables to forecast a progressive mitigation of this knowledge gap about Brazilian anurans in future decades.

New technologies, including portable recorders, automated recording systems and novel analyses software, have played an important role in the advance of studies on animal acoustic communication [65,66]. A constant improvement and miniaturization of the sound devices, together with a decrease in price, have contributed to make studies on acoustic signals more affordable, allowing a greater number of researches to be completed [66]. However, at the same time that the interest in bioacoustics has recently increased, standardarization in sound recording, analyses or terminology are still scarce [14,64].

### Knowledge gaps: Taxa

Our review pointed out that the advertisement calls of anuran families with restrict distribution and including many recently described species (e.g., Brachycephalidae—genera *Brachycephalus* and *Ischnocnema*) were less studied. The genera *Amazophrynella*, *Melanophryniscus*, *Dendrophryniscus* and *Oreophrynella* (Bufonidae), *Cycloramphus* (Cycloramphidae), and *Megaelosia* (Hylodidae) have scarce or no studies on their calls. Other genera, such as *Phasmahyla* and *Phrynomedusa* (Phyllomedusidae), show less than a half of their species with their advertisement calls described. Many of the genera mentioned above are endemic to Brazil, being most of their species ranked in some threatened category of the IUCN (see S1 Table) or classified as data deficient (DD) due to noticeable lack of basic knowledge about their biology, distribution, population size and trends. Overall, species with their calls undescribed are found in places of difficult access, are poorly known in terms of geographic distribution or general biology, have cryptic habit, inhabiting forest floor and those that live near streams (rheophilic habit), or have a high morphological similarity with other taxa, becoming more challenging to study (e.g., *Pristimantis* spp.—familiy Craugastoridae).

Although no specific statistical analyses were performed, it is noticed that anurans with large distribution areas (e.g., *Leptodactylus fuscus* and *Dendropsophus minutus*) show a larger number of call descriptions. Some of these species are currently considered a complex of species (e.g., *Leptodactylus fuscus* in [67] and *Dendropsophus minutus* in [68]), and information on their vocalizations may help to elucidate such taxonomic problems. Besides, widely distributed species are generally more generalist in relation to their habitat and more abundant, occurring in a broad variety of environments [13]. Since these species are easily found, they are widely studied, serving as suitable models for testing ecological hypotheses (e.g., [69]) and evaluating how anthropic activities can affect this group of organisms (e.g., [70,71]).

### Knowledge gaps: Metadata

The most common and serious problems with respect to call descriptions are associated with the lack of standardized methodological procedures of analysis [14,34], which difficult comparisons of call characteristics among studies and species. Specifically, studies are highly variable in the metadata recorded and call descriptions are often poorly documented. In general, the studies analyzed often lack a description of the general properties of the calls (e.g., type of call, number of notes per call, number of calls per call series, structure of notes or calls, arrangement in groups or in series, presence or absence of harmonics). Our review identied only six among the twenty-five analyzed metadata variables being presented in more than 75% of the studies. These variables are considered a quality standard in call descriptions and also to offer some important information on the natural history of the species. Detailed information on all recording conditions such as precise locality, date, time of recording, abiotic (e.g., air and/or water temperature, and relative humidity) and biotic conditions (calling behaviour, social context), and also the description of the recording gear (e.g., recorder, microphone), recording settings (e.g., sampling rate, bit depth, format), and all procedures conducted during analysis (e.g., software, filtering, spectral settings) are part of a call description [14,64] and are crucial information for comparisons among studies and species. Activity period, calling habitat and perch height can also be important features to guide future research [66].

In addition to provide an exhaustive description of methods, context, circunstances, terminology and numerical parameters used in the studies, at least the following acoustic parameters should be analyzed and described in detail: (1) duration of calls, notes and/or pulses; (2) duration of intervals between calls, notes and/or pulses; (3) repetition rate of calls and/or notes per time unit; (4) pulse number (when applicable); (5) dominant frequency of calls and/or notes; (6) fundamental frequency of calls and/or notes; (7) bandwidth of calls and/or notes (or approximate prevalent bandwidth), i.e., difference between the uppest and lowest frequencies; and (8) harmonics. Besides, when possible, efforts to include information about the absolute or relative amplitude (sound pressure level ─ SPL) of the calls and of the background noise in which the calling males are inserted, would be highly appreciated. The knowledge of sound levels allows an estimation of the maximum distance in which intraspecific communication can occur (due to degradation and attenuation of sound), of the energy spent during communication, and of the active space in programs of passive acoustic monitoring [72]. Furthermore, this variable enables to guide the choice of sound amplitude levels in playback tests and behavioral studies [73,74]. The sound level information was previously reported for some American anurans (e.g., [73,75,76]). However, for most species of tropical anurans, as demonstrated in this study, this call parameter is unknown.

### Knowledge gaps: Biome

Some studies suggest that biomes with seasonally dry climatic features are historically neglected by taxonomists and geographers, as is the case of Cerrado and Caatinga [77,78]. However, these biomes, together with Pantanal and Pampa, showed the highest percentage of species with their advertisement calls described. This is likely because of the relative lower diversity and endemism rate of these biomes in comparison with humid forest biomes, such as the Amazon and the Atlantic Forest, where more studies on anuran species have traditionally been conducted [54,56,79]. Cerrado was lately considered as a hotspot of biodiversity [80], which has promoted a recent interest and a greater number of studies aimed at this biome. This trend has also been reinforced by the increase in the number of universities and researchers located in the region. On the other hand, the fact that Pantanal was the biome in which more species calls were studied may be explained by an inherent positive bias from taxonomists, higher number of common species and easier to sample [81,82]. In general, biomes with a greater density of access routes are those with higher biological knowledge [83], while regions having difficult access typically show better conservation status and distinct species, and should therefore be considered as priority areas for biodiversity inventories [83]. Efforts must also be allocated according to endemism rate of each region or biome. Atlantic Forest, Caatinga, and Pampa were the less studied biomes in terms of the advertisement calls of their endemic species (S2 Table). The low number of studies describing advertisement calls of endemic species can indicate that these species are in general still poorly studied and likely many of them have no data on reproductive biology or population size. Nevertheless, it should be highlight that studies across biomes in widely distributed species may help to identify the existence of cryptic species, when pronounced differences in their advertisement calls are found along the species range [84].

### Knowledge gaps: Categories of threat

A considerable gap of knowledge on the advertisement calls of species classified within the IUCN threatened categories were found, and specifically of species in categories VU and CR, which less than 40% have their advertisement calls described. Contrary to expectations, the species that do not have their threatened status evaluated (20% of the total) and those with data deficient (24% of total), showed a higher rate of their calls described, i.e. 54%, than species in the threatened categories. The IUCN’s Red List is an important conservation tool, not only to estimate the threat status of species, but also to guide conservation strategies [85]. Species classified as threatened (CR, EN, or VU) are usually prioritized in conservation action plans [85] and, according to our review, they should also be prioritized in efforts for describing anuran calls and conducting bioacoustics studies. Data deficient species are those which data available during the assessment process is not adequate to determine threat category [86], and amphibians are the terrestrial vertebrate group with the highest proportion of DD species [86,87]. It is therefore necessary to give special attention to DD species which were described more than 50 years ago and those geographically restricte[86,87].

### Publication of call descriptions

The description of advertisement calls of Brazilian anurans has been published in a wide variety of scientific journals and books, although most of the publications were gathered in a few journals, particularly Zootaxa and herpetological journals. In recent years, the number of authors collaborating on publications featuring call descriptions has been increasing noticeably. Likely, several benefits have promoted these scientific collaborations [88,89], such as addressing increasingly complex biological questions, sharing costs in laboratory studies, or exchanging information and building networks in multi-taxa inventories, with researchers specialized in various taxonomic groups [90], which also stimulate collaborations in different research areas [89,91]. Also, articles with more number of authors tend to be publicated in higher-ranked journals and have usually more citations [90,91]. Considering the Brazilian scientific productivity, it is possible to observe an increase in number of authors per article that is similar to that of developed countries, however these publications remain less cited [90]. As Brazil is a country of large territorial size, with different biomes and restricted access to many areas, there are still abundant localities to be deeply explored, and it is possible to notice that regions close to large universities or protected areas are often more intensely studied [83,92].

### Further directions

It is important to emphasize that anurans may present a broad vocal repertoire, with calls other than the advertisement call, and most of the species do not yet have all call types described. The acoustic social interactions during reproductive activity are complex and the species may present different strategies to communicate, depending of the social and ecological contexts. In a recently study, Toledo et al. [16] reviewed the calling repertoire terminology of anurans and proposed a classification based in three categories: reproductive, aggressive, and defensive calls. The calls classified in the reproductive category (e.g., advertisement, courtship, amplectant, and release calls) are the most studied and commonly described vocalizations. Due to the importance of acoustic signals in the communication of anurans and the existence of a complex vocal repertoire for many species, it is necessary not only to describe the advertisement calls and other vocaizations, but also to use experimental approaches to elucidate the function of each type of call. Thus, a considerable sampling effort under different social, ecological and climatic condictions is required to study the vocal repertoire of anurans.

The development of acoustic methodologies for biodiversity appraisal has been substantial in the last years [25,93]. The use of automatic sound recorders for acoustic monitoring terrestrial and aquatic environments is a relatively recent technique and has been applied on a large scale in many recent studies [94]. Besides being an efficient and non-invasive technique, passive acoustic monitoring enables simultaneous sampling at several scales in time and space, with reduced observer biases and animal disturbances [64]. Although there are methods to circumvent the need of knowledge about all species occurring in habitats with high diversity [79,80], information about the species-specific calls provides a more powerful and cost effective tool for the study of communities [25], and for monitoring of populations over the years in the light of global change [24,95] or other major evolutionary trends such as effects of introduced species [96]. Due to the relevance of bioacoustics in anuran species delimitation, the deposit of recorded calls in sound libraries may also facilitate the taxonomic studies [16,28]. Just like specimens and genetic samples curated in museums and other collections, sound archives are important repositories of worldwide biodiversity, storing significant information on the species [14,97]. Thus, the deposit of sound archives in audiovisual collections accessible to researchers must become a common practice for assisting further investigations.

The Raunkiaeran shortfalls are tipically present in poorly known regions of developing countries, characterized by recent and wide human occupation and often by high rate of biodiversity [8,98]. Knowledge about diversity and natural history of the species is relevant to identify current trends and to support future studies. Anuran species arised millions of years ago and have expanded into a wide range of environments [13], but many species are currently experiencing dramatic declines and even local and global extinctions mainly due to the anthropic activities [99,100]. Since the advertisement call and other acoustic signals are often species-specific and contain phylogenetic signal, they can be used for delimiting and identifying anuran taxa. A complete taxonomy is at the core of threat status assessments, and hence the knowledge about advertisement calls may assist for the effective planning of conservation and future management of amphibian diversity [14]. The exponential growth in the number of species and call descriptions suggests promising scientific advance for the coming years. However, in many developing countries such as Brazil, the adoption of laws that promote deforestation and other impacts on ecosystems as well as the reduction of investments in education and research may severely jeopardize this advance and conservation strategies. The long-term success of the efforts to know and protect biodiversity depends on a large series of factors, such as research investment, the establishment of research networks, preservation of natural habitats or public engagement in science and conservation, among many others [101,102]. Natural history may significantly contribute to this aim as shown in the present study, and we emphasize the need that key actors encourage its role in the scientific training and academic career of future generations of biologists.

### Conclusion

The bibliographical review of the description of advertisement calls of anuran species of Brazil suggested the existence of Raunkiaeran shortfall on this key functional trait. This shortfall was highly variable across families and mainly focused on species classified as threatened taxa by the UICN and the BRB and species occurring in biomes with the highest anuran diversity and endemism rate. In summary, our scientometric analysis provides a general insight about Brazilian anuran species, supplying a detailed guide for orienting future efforts on the description and study of their acoustic signals, behavior, taxonomy and natural history.

## Supporting information

S1 Table Species list of Brazilian anurans and reference of the descriptions of the species and calls. (PDF)

S2 Table Number and percentage of Brazilian anuran species with described calls per each biome. (PDF)

S1 Text Collected information and metadata variables from each study (only presence or absence). (PDF)

S2 Text Complete list of references cited in the S1 Table. (PDF)
