# Supplementary material for: The advertisement calls of Brazilian anurans: Historical review, current knowledge and future directions
Source: PLoS One. 2018 Jan 30;13(1):e0191691. doi: 10.1371/journal.pone.0191691 (PMC5790252; doi:10.1371/journal.pone.0191691)
Supplement: S1 Table — (PDF) [file pone.0191691.s001.pdf]

S1 Table. Species list of Brazilian anurans and reference of the descriptions of the species and calls. Abreviations: CR = Critically Endangered; DD = Data Deficient; EN = Endangered; EX = Extinct; LC = Least Concern; NA = Not Evaluated; NT = Near Threatened; VU = Vulnerable; AF = Atlantic Forest; AM = Amazon; CA = Caatinga; CE = Cerrado; PM = Pampa; PN = Pantanal; FZ = Fonoteca Zoológica of the National Museum of Natural Sciences, Madrid, Spain; ML = Maculay Library of the Cornell Lab of Ornithology, New York, USA; JV = Fonoteca Neotropical Jacques Vielliard of the Campinas State University, Campinas, Brazil. In gray the first descriptions of advertisement calls.

Taxa	Family	IUCN	Description Reference	Call_reference	Biome	Sound Library
Allophryne ruthveni	Allophrynidae	LC	Gaige 1926	Caldwell & Hoogmoed 1998	AM, AF	FZ, ML, JV
Allophryne ruthveni	Allophrynidae	LC	Gaige 1926	Lescure & Marty 2000	AM, AF	FZ, ML, JV
Allophryne relicta	Allophrynidae	NE	Caramaschi et al. 2013	Caramaschi et al. 2013	AF	–
Limnomedusa macroglossa	Alsodidae	LC	Duméril & Bibron 1841	NA	AF, PM	FZ, JV
Allobates bacurau	Aromobatidae	NE	Simões 2016	Simões 2016	AM	–
Allobates brunneus	Aromobatidae	LC	Cope 1887	Lima et al. 2009	CE	FZ, JV
Allobates caeruleodactylus	Aromobatidae	DD	Lima & Caldwell 2001	Lima & Caldwell 2001	AM	–
Allobates conspicuus	Aromobatidae	DD	Morales 2002	NA	AM	–
Allobates crombiei	Aromobatidae	DD	Morales 2002	Lima et al. 2012	AM	–
Allobates femoralis	Aromobatidae	LC	Boulenger 1884	Schlüter 1980a	AM	FZ, ML, JV
Allobates femoralis	Aromobatidae	LC	Boulenger 1884	Zimmerman 1983	AM	FZ, ML, JV
Allobates femoralis	Aromobatidae	LC	Boulenger 1884	Zimmerman & Bogart 1984	AM	FZ, ML, JV
Allobates femoralis	Aromobatidae	LC	Boulenger 1884	Lescure & Marty 2000	AM	FZ, ML, JV
Allobates femoralis	Aromobatidae	LC	Boulenger 1884	Duellman 2005	AM	FZ, ML, JV
Allobates femoralis	Aromobatidae	LC	Boulenger 1884	Amézquita et al. 2009	AM	FZ, ML, JV
Allobates flaviventris	Aromobatidae	NE	Melo-Sampaio et al. 2013	Melo-Sampaio et al. 2013	AM	–
Allobates fuscellus	Aromobatidae	DD	Morales 2002	NA	AM	–

Taxa	Family	IUCN	Description Reference	Call_reference	Biome	Sound Library
Allobates gasconi	Aromobatidae	DD	Morales 2002	NA	AM	–
Allobates grillisimilis	Aromobatidae	NE	Simões et al. 2013a	Simões et al. 2013a	AM	–
Allobates goianus	Aromobatidae	DD	Bokermann 1975	Bastos et al. 2011b	CE	–
Allobates goianus	Aromobatidae	DD	Bokermann 1975	Carvalho et al. 2016	CE	–
Allobates hodli	Aromobatidae	NE	Simões et al. 2010	Simões et al. 2010	AM	–
Allobates magnussoni	Aromobatidae	NE	Lima et al. 2014a	Lima et al. 2014a	AM	–
Allobates marchesianus	Aromobatidae	LC	Melin 1941	Schlüter 1980a	AM	FZ, ML, JV
Allobates marchesianus	Aromobatidae	LC	Melin 1941	Caldwell et al. 2002b	AM	FZ, ML, JV
Allobates masniger	Aromobatidae	DD	Morales 2002	Tsuji-Nishikido et al. 2012	AM	–
Allobates myersi	Aromobatidae	LC	Pyburn 1981	Pyburn 1981	AM	–
Allobates myersi	Aromobatidae	LC	Pyburn 1981	Simões & Lima 2011	AM	–
Allobates nidicola	Aromobatidae	DD	Caldwell & Lima 2003	Tsuji-Nishikido et al. 2012	AM	–
Allobates nidicola	Aromobatidae	DD	Caldwell & Lima 2003	Tsuji-Nishikido et al. 2012	AM	–
Allobates olfersioides	Aromobatidae	VU	Lutz 1925a	NA	AF, CA	FZ, ML, JV
Allobates paleovarzensis	Aromobatidae	NT	Lima et al. 2010a	Lima et al. 2010a	AM	–
Allobates paleovarzensis	Aromobatidae	NT	Lima et al. 2010a	Kaefer & Lima 2012	AM	–
Allobates subfolionidificans	Aromobatidae	VU	Lima et al. 2007b	Lima et al. 2007b	AM	–
Allobates sumtuosus	Aromobatidae	DD	Morales 2002	Simões et al. 2013b	AM	–
Allobates tapajos	Aromobatidae	NE	Lima et al. 2015	Lima et al. 2015	AM	–
Allobates vanzolinius	Aromobatidae	DD	Morales 2002	NA	AM	–
Anomaloglossus apiau	Aromobatidae	NE	Fouquet et al. 2015b	Fouquet et al. 2015b	AM	–
Anomaloglossus baeobatrachus	Aromobatidae	DD	Boistel & Massari 1999	Lescure & Marty 2000	AM	FZ
Anomaloglossus roraima	Aromobatidae	DD	La Marca 1998	Kok et al. 2013	AM	–
Anomaloglossus stepheni	Aromobatidae	LC	Martins 1989	Martins 1989	AM	JV
Anomaloglossus tamacuarensis	Aromobatidae	DD	Myers & Donnelly 1997	Myers & Donnelly 1997	AM	–
Anomaloglossus tepequem	Aromobatidae	NE	Fouquet et al. 2015b	NA	AM	–
Brachycephalus albolineatus	Brachycephalidae	NE	Bornschein et al. 2016	NA	AF	–

Taxa	Family	IUCN	Description Reference	Call_reference	Biome	Sound Library
Brachycephalus alipioi	Brachycephalidae	DD	Pombal & Gasparini 2006	NA	AF	–
Brachycephalus atelopoide	Brachycephalidae	NE	Miranda-Ribeiro 1920e	NA	AF	–
Brachycephalus auroguttatus	Brachycephalidae	NE	Ribeiro et al. 2015	NA	AF	–
Brachycephalus boticario	Brachycephalidae	NE	Ribeiro et al. 2015	NA	AF	–
Brachycephalus brunneus	Brachycephalidae	DD	Ribeiro et al. 2005	NA	AF	–
Brachycephalus bufonoides	Brachycephalidae	NE	Miranda-Ribeiro 1920e	NA	AF	–
Brachycephalus crispus	Brachycephalidae	NE	Condez et al. 2014	Condez et al. 2014	AF	–
Brachycephalus didactylus	Brachycephalidae	LC	Izecksohn 1971	NA	AF	–
Brachycephalus ephippium	Brachycephalidae	LC	Spix 1824	Pombal et al. 1994	AF	FZ, JV
Brachycephalus ferruginus	Brachycephalidae	DD	Alves et al. 2006	NA	AF	–
Brachycephalus fuscolineatus	Brachycephalidae	NE	Ribeiro et al. 2015	NA	AF	–
Brachycephalus garbeanus	Brachycephalidae	NE	Miranda-Ribeiro 1920e	NA	AF	–
Brachycephalus guarani	Brachycephalidae	NE	Clemente-Carvalho et al. 2012	NA	AF	–
Brachycephalus hermogenesi	Brachycephalidae	LC	Giaretta & Sawaya 1998	Verdade et al. 2008	AF	JV
Brachycephalus izecksohni	Brachycephalidae	DD	Ribeiro et al. 2005	NA	AF	–
Brachycephalus leopardus	Brachycephalidae	NE	Ribeiro et al. 2015	NA	AF	–
Brachycephalus margaritatus	Brachycephalidae	NE	Pombal & Izecksohn 2011	NA	AF	–
Brachycephalus mariaeterezae	Brachycephalidae	NE	Ribeiro et al. 2015	NA	AF	–
Brachycephalus nodoterga	Brachycephalidae	DD	Miranda-Ribeiro 1920e	NA	AF	JV
Brachycephalus olivaceus	Brachycephalidae	NE	Ribeiro et al. 2015	NA	AF	–
Brachycephalus pernix	Brachycephalidae	DD	Pombal et al. 1998	NA	AF	–
Brachycephalus pitanga	Brachycephalidae	NE	Alves et al. 2009	Araújo et al. 2012	AF	JV
Brachycephalus pombali	Brachycephalidae	DD	Alves et al. 2006	NA	AF	–
Brachycephalus pulex	Brachycephalidae	NE	Napoli et al. 2011a	NA	AF	–
Brachycephalus quiririensis	Brachycephalidae	NE	Pie & Ribeiro 2015	NA	AF	–
Brachycephalus sulfuratus	Brachycephalidae	NE	Condez et al. 2016	Condez et al. 2016	AF	–
Brachycephalus toby	Brachycephalidae	NE	Haddad et al. 2010	NA	AF	–

Taxa	Family	IUCN	Description Reference	Call_reference	Biome	Sound Library
Brachycephalus tridactylus	Brachycephalidae	NE	Garey et al. 2012a	Garey et al. 2012a	AF	JV
Brachycephalus vertebralis	Brachycephalidae	DD	Pombal 2001	NA	AF	–
Brachycephalus verrucosus	Brachycephalidae	NE	Ribeiro et al. 2015	NA	AF	–
Ischnocnema abdita	Brachycephalidae	NE	Canedo & Pimenta 2010	NA	AF	JV
Ischnocnema bolbodactyla	Brachycephalidae	LC	Lutz 1925a	Pombal & Cruz 1999	AF	FZ, JV
Ischnocnema concolor	Brachycephalidae	NE	Targino et al. 2009	NA	AF	JV
Ischnocnema epipeda	Brachycephalidae	NT	Heyer 1984	NA	AF	–
Ischnocnema erythromera	Brachycephalidae	DD	Heyer 1984	NA	AF	–
Ischnocnema gehrti	Brachycephalidae	DD	Miranda-Ribeiro 1926	NA	AF	–
Ischnocnema gualteri	Brachycephalidae	LC	Lutz 1974	Heyer 1984	AF	ML
Ischnocnema guentheri	Brachycephalidae	LC	Steindachner 1864	Heyer 1984	AF	FZ, ML, JV
Ischnocnema guentheri	Brachycephalidae	LC	Steindachner 1864	Heyer et al. 1990	AF	FZ, ML, JV
Ischnocnema guentheri	Brachycephalidae	LC	Steindachner 1863	Pombal 2010	AF	FZ, ML, JV
Ischnocnema henselii	Brachycephalidae	LC	Peters 1870	Conte et al. 2010	AF	FZ, JV
Ischnocnema hoehnei	Brachycephalidae	LC	Lutz 1958	Oliveira et al. 2008	AF	–
Ischnocnema holti	Brachycephalidae	DD	Cochran 1948a	NA	AF	–
Ischnocnema izecksohni	Brachycephalidae	DD	Caramaschi & Kisteumacher 1989a	Taucce et al. 2012	AF	–
Ischnocnema juipoca	Brachycephalidae	LC	Sazima & Cardoso 1978	Sazima & Cardoso 1978	AF	FZ, JV
Ischnocnema juipoca	Brachycephalidae	LC	Sazima & Cardoso 1978	Haddad et al. 1988	AF	FZ, JV
Ischnocnema karst	Brachycephalidae	NE	Canedo et al. 2012	NA	AF	–
Ischnocnema lactea	Brachycephalidae	LC	Miranda-Ribeiro 1923b	NA	AF	–
Ischnocnema manezinho	Brachycephalidae	NT	Garcia 1996	Castanho & Haddad 2000	AF	FZ, JV
Ischnocnema melanopygia	Brachycephalidae	NE	Targino et al. 2009	NA	AF	JV
Ischnocnema nanahallux	Brachycephalidae	NE	Brusquetti et al. 2013	NA	AF	–
Ischnocnema nasuta	Brachycephalidae	LC	Lutz 1925b	Heyer 1984	AF	ML, JV
Ischnocnema nigriventris	Brachycephalidae	DD	Lutz 1925a	Berneck et al. 2013	AF	–
Ischnocnema octavioi	Brachycephalidae	LC	Bokermann 1965a	NA	AF	–

Taxa	Family	IUCN	Description Reference	Call_reference	Biome	Sound Library
Ischnocnema oea	Brachycephalidae	NT	Heyer 1984	Hepp & Canedo 2013	AF	–
Ischnocnema paranaensis	Brachycephalidae	DD	Langone & Segalla 1996	NA	AF	–
Ischnocnema parva	Brachycephalidae	LC	Girard 1853	Heyer et al. 1990	AF	ML, JV
Ischnocnema penaxavantinho	Brachycephalidae	DD	Giaretta et al. 2007a	Giaretta et al. 2007a	CE	–
Ischnocnema pusilla	Brachycephalidae	DD	Bokermann 1967l	NA	AF	–
Ischnocnema randorum	Brachycephalidae	DD	Heyer 1985a	Heyer et al. 1990	AF	FZ, ML, JV
Ischnocnema sambaqui	Brachycephalidae	DD	Castanho & Haddad 2000	Castanho & Haddad 2000	AF	–
Ischnocnema spanios	Brachycephalidae	DD	Heyer 1985a	NA	AF	JV
Ischnocnema surda	Brachycephalidae	NE	Canedo et al. 2010	NA	AF	–
Ischnocnema venancioi	Brachycephalidae	LC	Lutz 1958	NA	AF	–
Ischnocnema verrucosa	Brachycephalidae	DD	Reinhardt & Lütken 1862	NA	AF	FZ
Ischnocnema vizottoi	Brachycephalidae	NE	Martins & Haddad 2010	Martins & Haddad 2010	AF	–
Amazophrynella bokermanni	Bufonidae	LC	Izecksohn 1994a	NA	AM	–
Amazophrynella manaos	Bufonidae	NE	Rojas-Zamora et al. 2014	NA	AM	–
Amazophrynella minuta	Bufonidae	LC	Melin 1941	Duellman 1978a	AM	–
Amazophrynella minuta	Bufonidae	LC	Melin 1941	Lescure & Marty 2000	AM	–
Amazophrynella vote	Bufonidae	NE	Avila et al. 2012a	NA	AM	–
Atelopus flavescens	Bufonidae	VU	Duméril & Bibron 1841	Lescure 1981a	AM	FZ, JV
Atelopus flavescens	Bufonidae	VU	Duméril & Bibron 1841	Lescure & Marty 2000	AM	FZ, JV
Atelopus hoogmoedi	Bufonidae	NE	Lescure 1974	Lescure 1981a	AM	FZ
Atelopus spumarius	Bufonidae	VU	Cope 1871a	Lescure 1981a	AM	FZ, ML
Atelopus spumarius	Bufonidae	VU	Cope 1871a	Cocroft et al. 1990	AM	FZ, ML
Dendrophryniscus berthalutzae	Bufonidae	LC	Izecksohn 1994b	NA	AF	–
Dendrophryniscus brevipollicatus	Bufonidae	LC	Jiménez de la Espada 1870	NA	AF	JV
Dendrophryniscus carvalhoi	Bufonidae	EN	Izecksohn 1994b	NA	AF	–
Dendrophryniscus krausae	Bufonidae	DD	Cruz & Fusinatto 2008	NA	AF	–
Dendrophryniscus leucomystax	Bufonidae	LC	Izecksohn 1968	NA	AF	–

Taxa	Family	IUCN	Description Reference	Call_reference	Biome	Sound Library
Dendrophryniscus oreites	Bufonidae	NE	Recoder et al. 2010	NA	AF	–
Dendrophryniscus organensis	Bufonidae	NE	Carvalho-e-Silva et al. 2010	NA	AF	–
Dendrophryniscus proboscideus	Bufonidae	NE	Boulenger 1882	NA	AF, CA	–
Dendrophryniscus skuki	Bufonidae	NE	Caramaschi 2012	NA	AF	–
Dendrophryniscus stawiarskyi	Bufonidae	DD	Izecksohn 1994b	NA	AF	–
Frostius erythrophthalmus	Bufonidae	DD	Pimenta & Caramaschi 2007	Juncá et al. 2012c	AF, CA	–
Frostius pernambucensis	Bufonidae	LC	Bokermann 1962e	Juncá et al. 2012c	AF, CA	JV
Melanophryniscus admirabilis	Bufonidae	CR	Di Bernardo et al. 2006	NA	AF	–
Melanophryniscus alipioi	Bufonidae	DD	Langone et al. 2008	NA	AF, PM	–
Melanophryniscus atroluteus	Bufonidae	LC	Miranda-Ribeiro 1920e	Baldo & Basso 2004	AF, PM	–
Melanophryniscus atroluteus	Bufonidae	LC	Miranda-Ribeiro 1920e	Kwet et al. 2005	AF, PM	–
Melanophryniscus biancae	Bufonidae	NE	Bornschein et al. 2015	NA	AF	–
Melanophryniscus cambaraensis	Bufonidae	DD	Braun & Braun 1979	NA	AF, PM	FZ
Melanophryniscus devincenzii	Bufonidae	EN	Klappenbach 1968	NA	PM	–
Melanophryniscus dorsalis	Bufonidae	VU	Mertens 1933	Kwet et al. 2005	AF, PM	FZ
Melanophryniscus fulvoguttatus	Bufonidae	LC	Mertens 1937	NA	CE, PN	–
Melanophryniscus klappenbachi	Bufonidae	LC	Prigioni & Langone 2000	Kurth et al. 2013b	PM	–
Melanophryniscus macrogranulosus	Bufonidae	VU	Braun 1973	NA	AF	FZ
Melanophryniscus milanoi	Bufonidae	NE	Bornschein et al. 2015	NA	AF	–
Melanophryniscus montevidensis	Bufonidae	VU	Philippi 1902	Kwet et al. 2005	AF, PM	FZ
Melanophryniscus moreirae	Bufonidae	NT	Miranda-Ribeiro 1920e	NA	AF	JV
Melanophryniscus pachyrhynus	Bufonidae	DD	Miranda-Ribeiro 1920e	Caldart et al. 2013	AF, PM	–
Melanophryniscus peritus	Bufonidae	CR	Caramaschi & Cruz 2011	NA	AF	–
Melanophryniscus sanmartini	Bufonidae	NT	Klappenbach 1968	NA	PM	–
Melanophryniscus setiba	Bufonidae	NE	Peloso et al. 2012	NA	AF	–
Melanophryniscus simplex	Bufonidae	DD	Caramaschi & Cruz 2002	NA	AF, PM	–
Melanophryniscus spectabilis	Bufonidae	DD	Caramaschi & Cruz 2002	NA	AF	–

Taxa	Family	IUCN	Description Reference	Call_reference	Biome	Sound Library
Melanophryniscus tumifrons	Bufonidae	LC	Boulenger 1905	NA	AF, PM	FZ, JV
Melanophryniscus vilavelhensis	Bufonidae	NE	Steinback-Padilha 2009	NA	AF, PM	–
Melanophryniscus xanthostomus	Bufonidae	NE	Bornschein et al. 2015	NA	AF	–
Oreophrynella quelchii	Bufonidae	VU	Boulenger 1895b	NA	AM	–
Oreophrynella weiassipuensis	Bufonidae	DD	Señaris et al. 2005	NA	AM	–
Rhaebo ecuadorensis	Bufonidae	NE	Mueses-Cisneros et al. 2012	NA	AM	–
Rhaebo guttatus	Bufonidae	LC	Schneider 1799	Lescure & Marty 2000	AM, CE	FZ, ML, JV
Rhaebo guttatus	Bufonidae	LC	Schneider 1799	Duellman 2005	AM, CE	FZ, ML, JV
Rhinella abei	Bufonidae	LC	Baldissera et al. 2004	Batista et al. 2016	AF	FZ, JV
Rhinella achavali	Bufonidae	LC	Maneyro et al. 2004	NA	AF, PM	–
Rhinella acutirostris	Bufonidae	LC	Spix 1824	NA	AM	–
Rhinella arenarum	Bufonidae	LC	Hensel 1867	Salas et al. 1998	PM	FZ, JV
Rhinella azarai	Bufonidae	NE	Gallardo 1965	Guerra et al. 2011	AF, CE	–
Rhinella bergi	Bufonidae	LC	Céspedez 2000	Guerra et al. 2011	PM, PN	–
Rhinella casconi	Bufonidae	NE	Roberto et al. 2014	Roberto et al. 2014	AF	JV
Rhinella castaneotica	Bufonidae	LC	Caldwell 1991	Köhler & Lötters 1999a	AM	FZ
Rhinella ceratophrys	Bufonidae	LC	Boulenger 1882	NA	AM	ML
Rhinella cerradensis	Bufonidae	DD	Maciel et al. 2007	Maciel et al. 2007	CE	–
Rhinella crucifer	Bufonidae	LC	Wied-Neuwied 1821a	Heyer et al. 1990	AF, CE	FZ, ML, JV
Rhinella crucifer	Bufonidae	LC	Wied-Neuwied 1821a	Oliveira et al. 2014	AF, CE	FZ, ML, JV
Rhinella dapsilis	Bufonidae	LC	Myers & Carvalho 1945	NA	AM	ML
Rhinella dorbignyi	Bufonidae	LC	Duméril & Bibron 1841	Guerra et al. 2011	AF, PM	FZ
Rhinella fernandezae	Bufonidae	LC	Gallardo 1957	Salas et al. 1998	AF, PM	FZ, JV
Rhinella fernandezae	Bufonidae	LC	Gallardo 1957	Guerra et al. 2011	AF, PM	FZ, JV
Rhinella granulosa	Bufonidae	LC	Spix 1824	Zimmerman 1983	AF, CA, CE, PN	FZ, ML, JV
Rhinella granulosa	Bufonidae	LC	Spix 1824	Duellman 1997	AF, CA, CE, PN	FZ, ML, JV
Rhinella granulosa	Bufonidae	LC	Spix 1824	Lescure & Marty 2000	AF, CA, CE, PN	FZ, ML, JV

Taxa	Family	IUCN	Description Reference	Call_reference	Biome	Sound Library
Rhinella granulosa	Bufonidae	LC	Spix 1824	São-Pedro et al. 2011	AF, CA, CE, PN	FZ, ML, JV
Rhinella gildae	Bufonidae	NE	Vaz-Silva et al. 2015	NA	AM	–
Rhinella henseli	Bufonidae	LC	Lutz 1934	NA	AF	FZ
Rhinella hoogmoedi	Bufonidae	LC	Caramaschi & Pombal 2006	Roberto et al. 2011	AF, CA	–
Rhinella icterica	Bufonidae	LC	Spix 1824	Heyer et al. 1990	AF, CE, PM	FZ, ML, JV
Rhinella icterica	Bufonidae	LC	Spix 1824	Pombal 2010	AF, CE, PM	FZ, ML, JV
Rhinella inopina	Bufonidae	NE	Vaz-Silva et al. 2012	Andrade et al. 2015	CE	–
Rhinella jimi	Bufonidae	LC	Stevaux 2002	Garda et al. 2010	AF, CA	JV
Rhinella magnussoni	Bufonidae	LC	Lima et al. 2007a	Lima et al. 2007a	AM	–
Rhinella major	Bufonidae	NE	Muller & Helmich 1936	Köhler et al. 1997	CE	ML, JV
Rhinella major	Bufonidae	NE	Muller & Helmich 1936	Guerra et al. 2011	CE	ML, JV
Rhinella major	Bufonidae	NE	Muller & Helmich 1936	Bernardes et al. 2015	CE	ML, JV
Rhinella margaritifera	Bufonidae	LC	Laurenti 1768	De La Riva et al. 1996a	AM, CE	FZ, ML, JV
Rhinella margaritifera	Bufonidae	LC	Laurenti 1768	Köhler et al. 1997	AM, CE	FZ, ML, JV
Rhinella margaritifera	Bufonidae	LC	Laurenti 1768	Lescure & Marty 2000	AM, CE	FZ, ML, JV
Rhinella marina	Bufonidae	LC	Linnaeus 1758	Schlüter 1981	AM	FZ, ML, JV
Rhinella marina	Bufonidae	LC	Linnaeus 1758	Lescure & Marty 2000	AM	FZ, ML, JV
Rhinella marina	Bufonidae	LC	Linnaeus 1758	Bernal et al. 2004	AM	FZ, ML, JV
Rhinella marina	Bufonidae	LC	Linnaeus 1758	Duellman 2005	AM	FZ, ML, JV
Rhinella martyi	Bufonidae	LC	Fouquet et al. 2007	Fouquet et al. 2007	AM	–
Rhinella merianae	Bufonidae	NE	Gallardo 1965	Guerra et al. 2011	AM	–
Rhinella mirandaribeiroi	Bufonidae	NE	Gallardo 1965	Morais et al. 2012a	CE	–
Rhinella nattereri	Bufonidae	NE	Bokermann 1967e	NA	AM	–
Rhinella ocellata	Bufonidae	LC	Günther 1858	Caldwell & Shepard 2007	CE	JV
Rhinella ornata	Bufonidae	LC	Spix 1824	Heyer et al. 1990	AF, CE	JV
Rhinella ornata	Bufonidae	LC	Spix 1824	Pombal 2010	AF, CE	JV
Rhinella paraguayensis	Bufonidae	NE	Ávila et al. 2010	Ávila et al. 2010	PN	–

Taxa	Family	IUCN	Description Reference	Call_reference	Biome	Sound Library
Rhinella proboscidea	Bufonidae	LC	Spix 1824	Zimmerman & Bogart 1988	AM	–
Rhinella pygmaea	Bufonidae	LC	Myers & Carvalho 1952	Carvalho et al. 2013c	AF	JV
Rhinella roqueana	Bufonidae	LC	Melin 1941	NA	AM	–
Rhinella rubescens	Bufonidae	LC	Lutz 1925b	Haddad et al. 1988	AF, CA	JV
Rhinella rubescens	Bufonidae	LC	Lutz 1925b	Maciel et al. 2007	AF, CA	JV
Rhinella schneideri	Bufonidae	LC	Werner 1894	Köhler et al. 1997	CA, CE, PM, PN	FZ, ML, JV
Rhinella schneideri	Bufonidae	LC	Werner 1894	Silva et al. 2008	CA, CE, PM, PN	FZ, ML, JV
Rhinella scitula	Bufonidae	DD	Caramaschi & Niemeyer 2003b	NA	CE, PN	–
Rhinella sebbeni	Bufonidae	NE	Vaz-Silva et al. 2015	NA	CE	–
Rhinella veredas	Bufonidae	LC	Brandão et al. 2007	NA	CE	–
Teratohyla adenocheira	Centrolenidae	DD	Harvey & Noonan 2005	NA	AM	–
Teratohyla midas	Centrolenidae	LC	Lynch & Duellman 1973	Twomey et al. 2014	AM	ML
Vitreorana baliomma	Centrolenidae	NE	Pontes et al. 2014	NA	AF	–
Vitreorana eurygnatha	Centrolenidae	LC	Lutz 1925a	Heyer et al. 1990	AF, CA	FZ, ML
Vitreorana franciscana	Centrolenidae	NE	Santana et al. 2015	Santana et al. 2015	CE	–
Vitreorana parvula	Centrolenidae	NE	Boulenger 1895a	NA	AF	–
Vitreorana ritae	Centrolenidae	DD	Lutz & Kloss 1952	Zimmerman 1983	AM	ML
Vitreorana ritae	Centrolenidae	DD	Lutz & Kloss 1952	Zimmerman & Bogart 1984	AM	ML
Vitreorana ritae	Centrolenidae	DD	Lutz & Kloss 1952	Lescure & Marty 2000	AM	ML
Vitreorana uranoscopa	Centrolenidae	LC	Müller 1924a	Heyer et al. 1990	AF, CE	FZ, ML
Vitreorana uranoscopa	Centrolenidae	LC	Müller 1924a	Haga et al. 2014	AF, CE	FZ, ML
Vitreorana uranoscopa	Centrolenidae	LC	Müller 1924a	Zaracho 2014	AF, CE	FZ, ML
Hyalinobatrachium cappellei	Centrolenidae	NE	van Lidth de Jeude 1904	Myers & Donnelly 1997	AM	–
Hyalinobatrachium cappellei	Centrolenidae	NE	van Lidth de Jeude 1904	Señaris & Ayarzagüena 2005	AM	–
Hyalinobatrachium cappellei	Centrolenidae	NE	van Lidth de Jeude 1904	Castroviejo-Fischer et al. 2011	AM	–
Hyalinobatrachium carlesvilai	Centrolenidae	NE	Castroviejo-Fischer et al. 2009	Castroviejo-Fischer et al. 2009	AM	FZ
Hyalinobatrachium iaspidiense	Centrolenidae	DD	Ayarzaqüena 1992	Lescure & Marty 2000	AM	–

Taxa	Family	IUCN	Description Reference	Call_reference	Biome	Sound Library
Hyalinobatrachium iaspidiense	Centrolenidae	DD	Ayarzaqüena 1992	Señaris & Ayarzagüena 2005	AM	–
Hyalinobatrachium iaspidiense	Centrolenidae	DD	Ayarzaqüena 1992	Castroviejo-Fischer et al. 2011	AM	–
Hyalinobatrachium mondolfii	Centrolenidae	NE	Señaris & Ayarzagüena 2001	Señaris & Ayarzagüena 2001	AM	–
Hyalinobatrachium mondolfii	Centrolenidae	NE	Señaris & Ayarzagüena 2001	Castroviejo-Fischer et al. 2011	AM	–
Hyalinobatrachium munozorum	Centrolenidae	NE	Señaris & Ayarzagüena 2001	NA	AM	–
Ceratophrys aurita	Ceratophryidae	LC	Raddi 1823	NA	AF, CA	ML, JV
Ceratophrys cornuta	Ceratophryidae	LC	Linnaeus 1758	Schlüter 1980b	AM	FZ, ML, JV
Ceratophrys cornuta	Ceratophryidae	LC	Linnaeus 1758	Márquez et al. 1995	AM	FZ, ML, JV
Ceratophrys cornuta	Ceratophryidae	LC	Linnaeus 1758	Lescure & Marty 2000	AM	FZ, ML, JV
Ceratophrys cornuta	Ceratophryidae	LC	Linnaeus 1758	Duellman 2005	AM	FZ, ML, JV
Ceratophrys cranwelli	Ceratophryidae	LC	Barrio 1980	Salas et al. 1998	PM, PN	FZ
Ceratophrys cranwelli	Ceratophryidae	LC	Barrio 1980	Lescano 2011	PM, PN	FZ
Ceratophrys cranwelli	Ceratophryidae	LC	Barrio 1980	Valetti et al. 2013	PM, PN	FZ
Ceratophrys joazeirensis	Ceratophryidae	DD	Mercadal de Barrio 1986	Zaidan & Leite 2012	AF, CA	JV
Ceratophrys ornata	Ceratophryidae	NT	Bell 1843	Barrio 1980	PM	JV
Ceratophrys ornata	Ceratophryidae	NT	Bell 1843	Salas et al. 1998	PM	JV
Lepidobatrachus asper	Ceratophryidae	NT	Budgett 1899	Barrio 1968	AM	–
Lepidobatrachus asper	Ceratophryidae	NT	Budgett 1899	Lescano 2011	AM	–
Haddadus aramunha	Craugastoridae	DD	Cassimiro et al. 2008	Costa et al. 2014	AF, CA	JV
Haddadus binotatus	Craugastoridae	LC	Spix 1824	Carvalho & Martins 2012	AF, CA	JV
Haddadus binotatus	Craugastoridae	LC	Spix 1824	Dias et al. 2012	AF, CA	JV
Haddadus binotatus	Craugastoridae	LC	Spix 1824	Moura et al. 2012	AF, CA	JV
Haddadus plicifer	Craugastoridae	DD	Boulenger 1888c	NA	AF	–
Strabomantis sulcatus	Craugastoridae	LC	Cope 1874	NA	AM	–
Eleutherodactylus bilineatus	Craugastoridae	LC	Bokermann 1974	NA	AF	–
Barycholos ternetzi	Craugastoridae	LC	Miranda-Ribeiro 1937a	Guimarães et al. 2001	CE	FZ, JV
Barycholos ternetzi	Craugastoridae	LC	Miranda-Ribeiro 1937a	Lemes et al. 2012	CE	FZ, JV

Taxa	Family	IUCN	Description Reference	Call_reference	Biome	Sound Library
Euparkerella brasiliensis	Craugastoridae	LC	Parker 1926	Hepp & Carvalho-e-Silva 2011	AF	–
Euparkerella cochranae	Craugastoridae	LC	Izecksohn 1988	Hepp & Carvalho-e-Silva 2011	AF	–
Euparkerella cryptica	Craugastoridae	NE	Hepp et al. 2015	Hepp et al. 2015	AF	–
Euparkerella robusta	Craugastoridae	VU	Izecksohn 1988	Hepp et al. 2015	AF	–
Euparkerella tridactyla	Craugastoridae	VU	Izecksohn 1988	Hepp et al. 2015	AF	–
Holoaden bradei	Craugastoridae	CR	Lutz 1958	NA	AF	JV
Holoaden luederwaldti	Craugastoridae	DD	Miranda-Ribeiro 1920a	Martins 2010	AF	–
Holoaden pholeter	Craugastoridae	DD	Pombal et al. 2008	NA	AF	–
Holoaden suarezi	Craugastoridae	NE	Martins & Zaher 2013	NA	AF	–
Noblella myrmecoides	Craugastoridae	LC	Lynch 1976	NA	AM	–
Oreobates crepitans	Craugastoridae	DD	Bokermann 1965d	Strussman et al. 2011	CE	JV
Oreobates heterodactylus	Craugastoridae	DD	Miranda-Ribeiro 1937a	Padial & De la Riva 2005	CE	FZ
Oreobates quixensis	Craugastoridae	LC	Jiménez de la Espada 1872	Heyer & Gascon 1995	AM	FZ, ML, JV
Oreobates remotus	Craugastoridae	NE	Teixeira et al. 2012a	Teixeira et al. 2012a	CE	ML
Ceuthomantis cavernibardus	Craugastoridae	DD	Myers & Donnelly 1997	Myers & Donnelly 1997	AM	–
Pristimantis academicus	Craugastoridae	NE	Lehr et al. 2010	NA	AM	–
Pristimantis acuminatus	Craugastoridae	LC	Schreve 1935	NA	AM	–
Pristimantis altamazonicus	Craugastoridae	LC	Barbour & Dunn 1921	NA	AM	–
Pristimantis aureolineatus	Craugastoridae	LC	Guayasamin et al. 2006	McCracken & Forstner 2006	AM	–
Pristimantis aureoventris	Craugastoridae	EN	Kok et al. 2011	Kok et al. 2011	AM	–
Pristimantis buccinator	Craugastoridae	LC	Rodríguez 1994	Rodríguez 1994	AM	–
Pristimantis carvalhoi	Craugastoridae	LC	Lutz & Kloss 1952	NA	AM	–
Pristimantis chiastonotus	Craugastoridae	LC	Lynch & Hoogmoed 1977	Lynch & Hoogmoed 1977	AM	–
Pristimantis chiastonotus	Craugastoridae	LC	Lynch & Hoogmoed 1977	Lescure & Marty 2000	AM	–
Pristimantis conspicillatus	Craugastoridae	LC	Günther 1858	NA	AM	JV
Pristimantis delius	Craugastoridae	DD	Duellman & Mendelson 1995	NA	AM	–
Pristimantis diadematus	Craugastoridae	LC	Jiménez de la Espada 1875	NA	AM	–

Taxa	Family	IUCN	Description Reference	Call_reference	Biome	Sound Library
Pristimantis dundeei	Craugastoridae	DD	Heyer & Muñoz 1999	Heyer & Muñoz 1999	AM, CE	ML
Pristimantis dundeei	Craugastoridae	DD	Heyer & Muñoz 1999	Köhler 2000	AM, CE	ML
Pristimantis eurydactylus	Craugastoridae	LC	Hedges & Schlüter 1992	NA	AM	–
Pristimantis fenestratus	Craugastoridae	LC	Steindachner 1864	Zimmerman 1983	AM	FZ, ML, JV
Pristimantis fenestratus	Craugastoridae	LC	Steindachner 1864	Zimmerman & Bogart 1984	AM	FZ, ML, JV
Pristimantis fenestratus	Craugastoridae	LC	Steindachner 1864	Rodríguez 1994	AM	FZ, ML, JV
Pristimantis fenestratus	Craugastoridae	LC	Steindachner 1864	Márquez et al. 1995	AM	FZ, ML, JV
Pristimantis fenestratus	Craugastoridae	LC	Steindachner 1864	Heyer & Muñoz 1999	AM	FZ, ML, JV
Pristimantis fenestratus	Craugastoridae	LC	Steindachner 1864	Köhler 2000	AM	FZ, ML, JV
Pristimantis fenestratus	Craugastoridae	LC	Steindachner 1864	Duellman 2005	AM	FZ, ML, JV
Pristimantis fenestratus	Craugastoridae	LC	Steindachner 1864	Padial & de La Riva 2009	AM	FZ, ML, JV
Pristimantis gutturalis	Craugastoridae	LC	Hoogmoed et al. 1977	NA	AM	–
Pristimantis inguinalis	Craugastoridae	LC	Parker 1940	Lescure & Marty 2000	AM	–
Pristimantis lacrimosus	Craugastoridae	LC	Jiménez de la Espada 1875	Batallas & Brito 2014	AM	JV
Pristimantis lanthanites	Craugastoridae	LC	Lynch 1975	NA	AM	–
Pristimantis luscombei	Craugastoridae	DD	Duellman & Mendelson 1995	NA	AM	–
Pristimantis malkini	Craugastoridae	LC	Lynch 1980	NA	AM	–
Pristimantis marmoratus	Craugastoridae	LC	Boulenger 1900	Lescure & Marty 2000	AM	–
Pristimantis martiae	Craugastoridae	LC	Lynch 1974	NA	AM	–
Pristimantis memorans	Craugastoridae	DD	Myers & Donnelly 1997	Myers & Donnelly 1997	AM	–
Pristimantis ockendeni	Craugastoridae	LC	Boulenger 1912	NA	AM	JV
Pristimantis orcus	Craugastoridae	LC	Lehr et al. 2009	López-Rojas et al. 2013	AM	–
Pristimantis paulodutrai	Craugastoridae	LC	Bokermann 1974	Bokermann 1974	AF	JV
Pristimantis peruvianus	Craugastoridae	LC	Melin 1941	NA	AM	FZ, ML, JV
Pristimantis ramagii	Craugastoridae	LC	Boulenger 1888c	Heyer & Morato-de-Carvalho 2000	AF, CA	ML, JV
Pristimantis reichlei	Craugastoridae	NE	Padial & de La Riva 2009	Schlüter 1980b	AM	FZ, ML
Pristimantis reichlei	Craugastoridae	NE	Padial & de La Riva 2009	Rodríguez 1994	AM	FZ, ML

Taxa	Family	IUCN	Description Reference	Call_reference	Biome	Sound Library
Pristimantis reichlei	Craugastoridae	NE	Padial & de La Riva 2009	Duellman 2005	AM	FZ, ML
Pristimantis reichlei	Craugastoridae	NE	Padial & de La Riva 2009	Padial & de La Riva 2009	AM	FZ, ML
Pristimantis skydmainos	Craugastoridae	LC	Flores & Rodriguez 1997	Rodríguez 1994	AM	–
Pristimantis skydmainos	Craugastoridae	LC	Flores & Rodriguez 1997	Flores & Rodriguez 1997	AM	–
Pristimantis toftae	Craugastoridae	LC	Duellman 1978b	Márquez et al. 1995	AM	FZ, ML
Pristimantis toftae	Craugastoridae	LC	Duellman 1978b	Duellman 2005	AM	FZ, ML
Pristimantis variabilis	Craugastoridae	LC	Lynch 1968	NA	AM	–
Pristimantis ventrigranulosus	Craugastoridae	NE	Maciel et al. 2012	Maciel et al. 2012	CE	–
Pristimantis ventrimarmoratus	Craugastoridae	LC	Boulenger 1912	NA	AM	–
Pristimantis vilarsi	Craugastoridae	LC	Melin 1941	Heyer & Barrio-Amorós 2009	AM	ML
Pristimantis vinhai	Craugastoridae	LC	Bokermann 1974	NA	AF, CA	–
Pristimantis zeuctotylus	Craugastoridae	LC	Lynch & Hoogmoed 1977	Lynch & Hoogmoed 1977	AM	ML
Pristimantis zeuctotylus	Craugastoridae	LC	Lynch & Hoogmoed 1977	Lescure & Marty 2000	AM	ML
Pristimantis zimmermanae	Craugastoridae	LC	Heyer & Hardy 1991	Heyer & Hardy 1991	AM	–
Cycloramphus acangatan	Cycloramphidae	VU	Verdade & Rodrigues 2003	NA	AF	–
Cycloramphus asper	Cycloramphidae	DD	Werner 1899	NA	AF	–
Cycloramphus bandeirensis	Cycloramphidae	DD	Heyer 1983c	NA	AF	–
Cycloramphus bolitoglossus	Cycloramphidae	DD	Werner 1897	Lingnau et al. 2008b	AF	FZ
Cycloramphus boraceiensis	Cycloramphidae	LC	Heyer 1983c	Heyer & Mello 1979	AF	FZ, ML, JV
Cycloramphus boraceiensis	Cycloramphidae	LC	Heyer 1983c	Heyer et al. 1990	AF	FZ, ML, JV
Cycloramphus brasiliensis	Cycloramphidae	NT	Steindachner 1864	Heyer 1983c	AF	ML, JV
Cycloramphus carvalhoi	Cycloramphidae	DD	Heyer 1983c	NA	AF	–
Cycloramphus catarinensis	Cycloramphidae	DD	Heyer 1983c	NA	AF	–
Cycloramphus cedrensis	Cycloramphidae	DD	Heyer 1983b	Heyer 1983b	PM	ML
Cycloramphus diringshofeni	Cycloramphidae	DD	Bokermann 1957b	NA	PM	ML
Cycloramphus dubius	Cycloramphidae	LC	Miranda-Ribeiro 1920d	Giaretta & Cardoso 1995	AF	JV
Cycloramphus duseni	Cycloramphidae	DD	Andersson 1914	NA	AF	JV

Taxa	Family	IUCN	Description Reference	Call_reference	Biome	Sound Library
Cycloramphus eleutherodactylus	Cycloramphidae	DD	Miranda-Ribeiro 1920d	Brasileiro et al. 2007b	AF	JV
Cycloramphus faustoi	Cycloramphidae	CR	Brasileiro et al. 2007b	Brasileiro et al. 2007b	AF	–
Cycloramphus fuliginosus	Cycloramphidae	LC	Tschudi 1838	NA	AF	–
Cycloramphus granulosus	Cycloramphidae	DD	Lutz 1929a	NA	AF	JV
Cycloramphus izecksohni	Cycloramphidae	DD	Heyer 1983b	Heyer 1983c	AF	FZ, ML
Cycloramphus juimirim	Cycloramphidae	DD	Haddad & Sazima 1989	Haddad & Sazima 1989	AF	FZ
Cycloramphus lithomimeticus	Cycloramphidae	NE	Silva & Ouvernay 2012	NA	AF	–
Cycloramphus lutzorum	Cycloramphidae	DD	Heyer 1983c	Lima et al. 2010b	AF	–
Cycloramphus migueli	Cycloramphidae	DD	Heyer 1988	NA	AF	–
Cycloramphus mirandaribeiroi	Cycloramphidae	DD	Heyer 1983c	NA	AF	–
Cycloramphus ohausi	Cycloramphidae	DD	Wandolleck 1907	Heyer 1983c	AF	ML, JV
Cycloramphus organensis	Cycloramphidae	DD	Weber et al. 2011	NA	AF	–
Cycloramphus rhyakonastes	Cycloramphidae	LC	Heyer 1983c	Heyer 1983c	AF	ML
Cycloramphus semipalmatus	Cycloramphidae	NT	Miranda-Ribeiro 1920d	Heyer & Mello 1979	AF	FZ, ML, JV
Cycloramphus semipalmatus	Cycloramphidae	NT	Miranda-Ribeiro 1920d	Heyer et al. 1990	AF	FZ, ML, JV
Cycloramphus stejnegeri	Cycloramphidae	DD	Noble 1924	NA	AF	–
Cycloramphus valae	Cycloramphidae	DD	Heyer 1983c	Heyer 1983b	AF	ML
Thoropa lutzi	Cycloramphidae	EN	Cochran 1938	Nunes-de-Almeida et al. 2016b	AF	ML, JV
Thoropa megatympanum	Cycloramphidae	LC	Caramaschi & Sazima 1984	Nunes-de-Almeida et al. 2016b	CE	FZ, JV
Thoropa miliaris	Cycloramphidae	LC	Spix 1824	Heyer et al. 1990	AF, CA, CE	FZ, JV
Thoropa miliaris	Cycloramphidae	LC	Spix 1824	Nunes-de-Almeida et al. 2016b	AF, CA, CE	FZ, JV
Thoropa petropolitana	Cycloramphidae	VU	Wandolleck 1907	Nunes-de-Almeida et al. 2016b	AF	ML, JV
Thoropa saxatilis	Cycloramphidae	NT	Cocroft & Heyer 1988	NA	AF	–
Thoropa taophora	Cycloramphidae	NE	Miranda-Ribeiro 1923e	Nunes-de-Almeida et al. 2016b	AF	ML, JV
Zachaenus carvalhoi	Cycloramphidae	DD	Izecksohn 1983	Guimarães et al. 2013	AF	JV
Zachaenus parvulus	Cycloramphidae	LC	Girard 1853	NA	AF	JV
Ameerega berohoka	Dendrobatidae	LC	Vaz-Silva & Maciel 2011	Vaz-Silva & Maciel 2011	CE	JV

Taxa	Family	IUCN	Description Reference	Call_reference	Biome	Sound Library
Ameerega berohoka	Dendrobatidae	LC	Vaz-Silva & Maciel 2011	Andrade et al. 2014	CE	JV
Ameerega braccata	Dendrobatidae	LC	Steindachner 1864	Forti et al. 2010	CE, PN	FZ, JV
Ameerega flavopicta	Dendrobatidae	LC	Lutz 1925a	Haddad et al. 1988	CE	FZ, JV
Ameerega flavopicta	Dendrobatidae	LC	Lutz 1925a	Haddad & Martins 1994	CE	FZ, JV
Ameerega flavopicta	Dendrobatidae	LC	Lutz 1925a	Martins & Giaretta 2012a	CE	FZ, JV
Ameerega hahneli	Dendrobatidae	LC	Boulenger 1884	Haddad & Martins 1994	AF	FZ, JV
Ameerega hahneli	Dendrobatidae	LC	Boulenger 1884	Köhler & Lötters 1999a	AF	FZ, JV
Ameerega hahneli	Dendrobatidae	LC	Boulenger 1884	Lescure & Marty 2000	AF	FZ, JV
Ameerega hahneli	Dendrobatidae	LC	Boulenger 1884	Duellman 2005	AF	FZ, JV
Ameerega macero	Dendrobatidae	LC	Rodríguez & Myers 1993	Rodríguez & Myers 1993	AM	–
Ameerega petersi	Dendrobatidae	LC	Silverstone 1976	Schlüter 1980a	AM	–
Ameerega petersi	Dendrobatidae	LC	Silverstone 1976	Myers et al. 1998	AM	–
Ameerega picta	Dendrobatidae	LC	Tschudi 1838	Schlüter 1980a	CE, PN	FZ, ML, JV
Ameerega picta	Dendrobatidae	LC	Tschudi 1838	Haddad & Martins 1994	CE, PN	FZ, ML, JV
Ameerega picta	Dendrobatidae	LC	Tschudi 1838	Köhler 2000	CE, PN	FZ, ML, JV
Ameerega pulchripecta	Dendrobatidae	DD	Silverstone 1976	Costa-Campos et al. 2016	AM	–
Ameerega trivittata	Dendrobatidae	LC	Spix 1824	Schlüter 1980b	AM	FZ, ML, JV
Ameerega trivittata	Dendrobatidae	LC	Spix 1824	Roithmair 1994	AM	FZ, ML, JV
Adelphobates castaneoticus	Dendrobatidae	LC	Caldwell & Myers 1990	NA	AM	JV
Adelphobates galactonotus	Dendrobatidae	LC	Steindachner 1864	NA	CE	–
Adelphobates quinquevittatus	Dendrobatidae	LC	Steindachner 1864	NA	AM	ML
Dendrobates leucomelas	Dendrobatidae	LC	Steindachner 1864	Lötters et al. 2003	AM	–
Dendrobates tinctorius	Dendrobatidae	LC	Cuvier 1797	Lescure & Marty 2000	AM	–
Ranitomeya amazonica	Dendrobatidae	DD	Schulte 1999	Brown et al. 2011	AM	–
Ranitomeya cyanovittata	Dendrobatidae	NE	Perez-Peña et al. 2010	NA	AM	–
Ranitomeya defleri	Dendrobatidae	NE	Twomey & Brown 2009	Twomey & Brown 2009	AM	–
Ranitomeya flavovittata	Dendrobatidae	LC	Schulte 1999	Brown et al. 2011	AM	–

Taxa	Family	IUCN	Description Reference	Call_reference	Biome	Sound Library
Ranitomeya sirensis	Dendrobatidae	LC	Aichinger 1991	Brown et al. 2011	AM	ML
Ranitomeya toraro	Dendrobatidae	NE	Brown et al. 2011	NA	AM	–
Ranitomeya uakarii	Dendrobatidae	LC	Brown et al. 2006	Brown et al. 2006	AM	–
Ranitomeya uakarii	Dendrobatidae	LC	Brown et al. 2006	Brown et al. 2011	AM	–
Ranitomeya vanzolinii	Dendrobatidae	LC	Myers 1982	Brown et al. 2011	AM	–
Ranitomeya variabilis	Dendrobatidae	DD	Zimmermann & Zimmermann 1988	Brown et al. 2011	AM	–
Ranitomeya yavaricola	Dendrobatidae	NE	Perez-Peña et al. 2010	Perez-Peña et al. 2010	AM	–
Hyloxalus chlorocraspedus	Dendrobatidae	DD	Caldwell 2005	NA	AM	–
Adelophryne adiastola	Eleutherodactylidae	LC	Hoogmoed & Lescure 1984	Heyer 1977	AM	FZ, ML
Adelophryne baturitensis	Eleutherodactylidae	VU	Hoogmoed et al. 1994	NA	AF, CA	–
Adelophryne glandulata	Eleutherodactylidae	NE	Lourenço-de-Moraes et al. 2014	NA	AF	–
Adelophryne gutturosa	Eleutherodactylidae	LC	Hoogmoed & Lescure 1984	MacCulloch et al. 2008	AM	–
Adelophryne maranguapensis	Eleutherodactylidae	EN	Hoogmoed et al. 1994	Lima et al. 2014b	AF, CA	–
Adelophryne meridionalis	Eleutherodactylidae	NE	Santana et al. 2012a	NA	AF	–
Adelophryne mucronatus	Eleutherodactylidae	NE	Lourenço-de-Moraes et al. 2012b	Lourenço-de-Moraes et al. 2012b	AF	JV
Adelophryne pachydactyla	Eleutherodactylidae	DD	Hoogmoed et al. 1994	NA	AF, CA	–
Phyzelaphryne miriamae	Eleutherodactylidae	LC	Heyer 1977	Heyer 1977	AM	ML
Phyzelaphryne miriamae	Eleutherodactylidae	LC	Heyer 1977	Heyer & Gascon 1995	AM	ML
Fritziana fissilis	Hemiphractidae	LC	Miranda-Ribeiro 1920c	Duellman & Gray 1983	AF	ML, JV
Fritziana fissilis	Hemiphractidae	LC	Miranda-Ribeiro 1920c	Franz & Mello 2015	AF	ML, JV
Fritiziana goeldii	Hemiphractidae	NE	Boulenger 1895a	Sinsch & Juraske 2006	AF	JV
Fritziana ohausi	Hemiphractidae	LC	Wandolleck 1907	Heyer et al. 1990	AF	FZ, ML, JV
Fritziana tonimi	Hemiphractidae	NE	Walker et al. 2016	NA	AF	–
Fritziana ulei	Hemiphractidae	NE	Miranda-Ribeiro 1926	NA	AF	–
Gastrotheca albolineata	Hemiphractidae	LC	Lutz & Lutz 1939	Izecksohn & Carvalho-e-Silva 2008	AF	JV
Gastrotheca ernestoi	Hemiphractidae	DD	Miranda-Ribeiro 1920c	Izecksohn & Carvalho-e-Silva 2008	AF	–
Gastrotheca fissipes	Hemiphractidae	LC	Boulenger 1888c	Mendes et al. 2012	AF, CA	JV

Taxa	Family	IUCN	Description Reference	Call_reference	Biome	Sound Library
Gastrotheca flamma	Hemiphractidae	DD	Juncá & Nunes 2008	NA	AF, CA	–
Gastrotheca fulvorufa	Hemiphractidae	DD	Andersson 1911	Izecksohn & Carvalho-e-Silva 2008	AF	JV
Gastrotheca megacephala	Hemiphractidae	NE	Izecksohn et al. 2009	Izecksohn et al. 2009	AF	–
Gastrotheca microdiscus	Hemiphractidae	LC	Lönnberg & Andersson 1910	Antunes & Haddad 2009	AF	–
Gastrotheca prasina	Hemiphractidae	NE	Teixeira et al. 2012c	Teixeira et al. 2012c	AF	–
Gastrotheca pulchra	Hemiphractidae	DD	Caramaschi & Rodrigues 2007	Loebmann et al. 2008	AF	JV
Gastrotheca recava	Hemiphractidae	NE	Teixeira et al. 2012c	Teixeira et al. 2012c	AF	–
Hemiphractus helioi	Hemiphractidae	LC	Sheil & Mendelson 2001	NA	AM	–
Hemiphractus scutatus	Hemiphractidae	LC	Spix 1824	NA	AM	–
Stefania neblinae	Hemiphractidae	NE	Carvalho et al. 2010b	NA	AM	–
Stefania tamacuarina	Hemiphractidae	DD	Myers & Donnelly 1997	NA	AM	–
Hyla imitator	Hylidae	DD	Barbour & Dunn 1921	NA	AM	–
Calamita melanorabdotus	Hylidae	DD	Schneider 1799	NA	UN	–
Aparasphenodon arapapa	Hylidae	NE	Pimenta et al. 2009	Lourenço-de-Moraes et al. 2013	AF	JV
Aparasphenodon bokermanni	Hylidae	DD	Pombal 1993	NA	AF	–
Aparasphenodon brunoi	Hylidae	LC	Miranda-Ribeiro 1920g	NA	AF	–
Aparasphenodon venezolanus	Hylidae	LC	Mertens 1950a	NA	AM	–
Aparasphenodon pomba	Hylidae	NE	Assis et al. 2013	NA	AF	–
Aplastodiscus albofrenatus	Hylidae	LC	Lutz 1924	Bokermann 1967f	AF	JV
Aplastodiscus albofrenatus	Hylidae	LC	Lutz 1924	Heyer et al. 1990	AF	JV
Aplastodiscus albosignatus	Hylidae	LC	Lutz & Lutz 1938	Bokermann 1967f	AF	FZ, JV
Aplastodiscus albosignatus	Hylidae	LC	Lutz & Lutz 1938	Heyer et al. 1990	AF	FZ, JV
Aplastodiscus albosignatus	Hylidae	LC	Lutz & Lutz 1938	Abrunhosa et al. 2005	AF	FZ, JV
Aplastodiscus albosignatus	Hylidae	LC	Lutz & Lutz 1938	Abrunhosa et al. 2005	AF	FZ, JV
Aplastodiscus arildae	Hylidae	LC	Cruz & Peixoto 1987	Orrico et al. 2006	AF	FZ, ML, JV
Aplastodiscus arildae	Hylidae	LC	Cruz & Peixoto 1987	Carvalho et al. 2006	AF	FZ, ML, JV
Aplastodiscus arildae	Hylidae	LC	Cruz & Peixoto 1987	Zina & Haddad 2006	AF	FZ, ML, JV

Taxa	Family	IUCN	Description Reference	Call_reference	Biome	Sound Library
Aplastodiscus cavicola	Hylidae	NT	Cruz & Peixoto 1985	Abrunhosa et al. 2005	AF, CA	JV
Aplastodiscus cochranae	Hylidae	LC	Mertens 1952	Garcia et al. 2001a	AF	FZ, JV
Aplastodiscus ehrhardti	Hylidae	LC	Müller 1924a	Conte et al. 2005	AF	FZ, JV
Aplastodiscus eugenioi	Hylidae	NT	Carvalho-e-Silva & Carvalho-e-Silva 2005	Hartmann et al. 2004	AF	ML
Aplastodiscus eugenioi	Hylidae	NT	Carvalho-e-Silva & Carvalho-e-Silva 2005	Miranda et al. 2016	AF	ML
Aplastodiscus flumineus	Hylidae	DD	Cruz & Peixoto 1985	NA	AF	JV
Aplastodiscus ibirapitanga	Hylidae	LC	Cruz et al. 2003	Abrunhosa et al. 2005	AF, CA	–
Aplastodiscus leucopygius	Hylidae	LC	Cruz & Peixoto 1985	Heyer et al. 1990	AF, CE	FZ, ML, JV
Aplastodiscus leucopygius	Hylidae	LC	Cruz & Peixoto 1985	Abrunhosa et al. 2005	AF, CE	FZ, ML, JV
Aplastodiscus leucopygius	Hylidae	LC	Cruz & Peixoto 1985	Zina & Haddad 2006	AF, CE	FZ, ML, JV
Aplastodiscus musicus	Hylidae	DD	Lutz 1949	NA	AF	–
Aplastodiscus perviridis	Hylidae	LC	Lutz 1950	Bokermann 1967f	AF, CE, PM	FZ, ML, JV
Aplastodiscus perviridis	Hylidae	LC	Lutz 1950	Garcia et al. 2001a	AF, CE, PM	FZ, ML, JV
Aplastodiscus perviridis	Hylidae	LC	Lutz 1950	Kwet 2001	AF, CE, PM	FZ, ML, JV
Aplastodiscus perviridis	Hylidae	LC	Lutz 1950	Haddad et al. 2005	AF, CE, PM	FZ, ML, JV
Aplastodiscus sibilatus	Hylidae	DD	Cruz et al. 2003	Abrunhosa et al. 2005	AF, CA	–
Aplastodiscus weygoldti	Hylidae	NT	Cruz & Peixoto 1987	Orrico et al. 2006	AF, CA	JV
Bokermannohyla ahenea	Hylidae	DD	Napoli & Caramaschi 2004	NA	AF	–
Bokermannohyla alvarengai	Hylidae	LC	Bokermann 1956a	Sazima et al. 1977	CE	JV
Bokermannohyla astartea	Hylidae	LC	Bokermann 1967d	Heyer et al. 1990	AF	ML, JV
Bokermannohyla caramaschii	Hylidae	LC	Napoli 2005	NA	AF	–
Bokermannohyla carvalhoi	Hylidae	LC	Peixoto 1981a	Carvalho et al. 2012	AF	JV
Bokermannohyla capra	Hylidae	NE	Napoli & Pimenta 2009	Napoli & Pimenta 2009	AF, CA	–
Bokermannohyla circumdata	Hylidae	LC	Cope 1871b	Heyer et al. 1990	AF	FZ, ML, JV
Bokermannohyla circumdata	Hylidae	LC	Cope 1871b	Carvalho et al. 2012	AF	FZ, ML, JV
Bokermannohyla claresignata	Hylidae	DD	Lutz & Lutz 1939	NA	AF	–
Bokermannohyla clepsydra	Hylidae	DD	Lutz 1925b	Bokermann 1972a	AF	FZ, ML, JV

Taxa	Family	IUCN	Description Reference	Call_reference	Biome	Sound Library
Bokermannohyla diamantina	Hylidae	DD	Napoli & Juncá 2006	Napoli & Juncá 2006	AF, CA	JV
Bokermannohyla flavopicta	Hylidae	NE	Leite et al. 2012	Rocha et al. 2016b	CE	–
Bokermannohyla gouveai	Hylidae	DD	Peixoto & Cruz 1992	NA	AF	–
Bokermannohyla hylax	Hylidae	LC	Heyer 1985a	Heyer 1985a	AF	FZ, ML, JV
Bokermannohyla hylax	Hylidae	LC	Heyer 1985a	Heyer et al. 1990	AF	FZ, ML, JV
Bokermannohyla hylax	Hylidae	LC	Heyer 1985a	Carvalho et al. 2012	AF	FZ, ML, JV
Bokermannohyla ibitiguara	Hylidae	DD	Cardoso 1983	Cardoso 1983	CE	JV
Bokermannohyla ibitiguara	Hylidae	DD	Cardoso 1983	Nali & Prado 2014	CE	JV
Bokermannohyla ibitipoca	Hylidae	DD	Caramaschi & Cruz 2013	Napole & Caramaschi 2004	AF	JV
Bokermannohyla itapoty	Hylidae	LC	Lugli & Haddad 2006a	Lugli & Haddad 2006a	AF, CA	JV
Bokermannohyla izecksohni	Hylidae	CR	Jim & Caramaschi 1979	NA	AF, CE	JV
Bokermannohyla juiju	Hylidae	NE	Faivovich et al. 2009	Taucce et al. 2015	CA, CE	–
Bokermannohyla langei	Hylidae	DD	Bokermann 1965b	NA	AF	–
Bokermannohyla lucianae	Hylidae	DD	Napoli & Pimenta 2003	Napoli & Pimenta 2003	AF	–
Bokermannohyla luctuosa	Hylidae	LC	Pombal & Haddad 1993	Carvalho et al. 2012	AF	JV
Bokermannohyla martinsi	Hylidae	LC	Bokermann 1964a	Pinheiro et al. 2014	AF	JV
Bokermannohyla nanuzae	Hylidae	LC	Bokermann & Sazima 1973a	Bokermann & Sazima 1973a	AF, CE	ML, JV
Bokermannohyla nanuzae	Hylidae	LC	Bokermann & Sazima 1973a	Napole & Caramaschi 2004	AF, CE	ML, JV
Bokermannohyla nanuzae	Hylidae	LC	Bokermann & Sazima 1973a	Carvalho et al. 2012	AF, CE	ML, JV
Bokermannohyla nanuzae	Hylidae	LC	Bokermann & Sazima 1973a	Walker et al. 2015	AF, CE	ML, JV
Bokermannohyla napolii	Hylidae	NE	Carvalho et al. 2012	Carvalho et al. 2012	CE	–
Bokermannohyla oxente	Hylidae	LC	Lugli & Haddad 2006a	Lugli & Haddad 2006a	AF, CA	–
Bokermannohyla oxente	Hylidae	LC	Lugli & Haddad 2006a	Giaretta et al. 2016	AF, CA	–
Bokermannohyla pseudopseudis	Hylidae	LC	Miranda-Ribeiro 1937a	Eterovick & Brandão 2001	CE	FZ
Bokermannohyla pseudopseudis	Hylidae	LC	Miranda-Ribeiro 1937a	Carvalho et al. 2013a	CE	FZ
Bokermannohyla ravida	Hylidae	DD	Caramaschi et al. 2001	Pombal & Haddad 1993	CE	–
Bokermannohyla sagarana	Hylidae	NT	Leite et al. 2011	NA	CE	–

Taxa	Family	IUCN	Description Reference	Call_reference	Biome	Sound Library
Bokermannohyla sapiranga	Hylidae	NE	Brandão et al. 2012	Guimarães et al. 2001	CE	JV
Bokermannohyla sapiranga	Hylidae	NE	Brandão et al. 2012	Brandão et al. 2012	CE	JV
Bokermannohyla sapiranga	Hylidae	NE	Brandão et al. 2012	Carvalho et al. 2013a	CE	JV
Bokermannohyla sapiranga	Hylidae	NE	Brandão et al. 2012	Furtado et al. 2016	CE	JV
Bokermannohyla saxicola	Hylidae	LC	Bokermann 1964a	Bokermann 1964a	AF, CA	JV
Bokermannohyla saxicola	Hylidae	LC	Bokermann 1964a	Eterovick & Brandão 2001	AF, CA	JV
Bokermannohyla sazimai	Hylidae	DD	Cardoso & Andrade 1982	Cardoso & Andrade 1982	CE	JV
Bokermannohyla sazimai	Hylidae	DD	Cardoso & Andrade 1982	Carvalho et al. 2012		
Bokermannohyla sazimai	Hylidae	DD	Cardoso & Andrade 1982	Carvalho & Giaretta 2013a		
Bokermannohyla vulcaniae	Hylidae	VU	Vasconcelos & Giaretta 2003	Gaiga et al. 2013	AF	JV
Corythomantis galeata	Hylidae	NE	Pombal et al. 2012	NA	AM	–
Corythomantis greeningi	Hylidae	LC	Boulenger 1896	Juncá et al. 2008	CA, CE	–
Dendropsophus acreanus	Hylidae	LC	Bokermann 1964e	Márquez et al. 1993	AM	FZ, JV
Dendropsophus anataliasiasi	Hylidae	LC	Bokermann 1972b	Teixeira & Giaretta 2015	CE	FZ, ML, JV
Dendropsophus anceps	Hylidae	LC	Lutz 1929b	Bokermann 1967f	AF, CE	–
Dendropsophus anceps	Hylidae	LC	Lutz 1929b	Gomes & Martins 2006	AF, CE	FZ, JV
Dendropsophus anceps	Hylidae	LC	Lutz 1929b	Conte et al. 2010	AF, CE	FZ, JV
Dendropsophus araguaya	Hylidae	DD	Napoli & Caramaschi 1998	NA	CE	FZ, JV
Dendropsophus berthalutzae	Hylidae	LC	Bokermann 1962a	Forti et al. 2012	AF	–
Dendropsophus bifurcus	Hylidae	LC	Andersson 1945	Duellman 1974	AM	JV
Dendropsophus bifurcus	Hylidae	LC	Andersson 1945	Duellman 1978a	AM	FZ, ML
Dendropsophus bifurcus	Hylidae	LC	Andersson 1945	Márquez et al. 1993	AM	FZ, ML
Dendropsophus bifurcus	Hylidae	LC	Andersson 1945	Kaiser & Hammers 2008	AM	FZ, ML
Dendropsophus bipunctatus	Hylidae	LC	Spix 1824	Abrunhosa et al. 2001	AF, CA	FZ, ML
Dendropsophus bokermanni	Hylidae	LC	Goin 1960	Duellman & Crump 1974	AM	ML, JV
Dendropsophus bokermanni	Hylidae	LC	Goin 1960	Duellman 1978a	AM	FZ, ML, JV
Dendropsophus bokermanni	Hylidae	LC	Goin 1960	Duellman & Pyles 1983	AM	FZ, ML, JV

Taxa	Family	IUCN	Description Reference	Call_reference	Biome	Sound Library
Dendropsophus bokermanni	Hylidae	LC	Goin 1960	Duellman 2005	AM	FZ, ML, JV
Dendropsophus branneri	Hylidae	LC	Cochran 1948b	Nunes et al. 2007b	AF, CA, CE	FZ, ML, JV
Dendropsophus brevifrons	Hylidae	LC	Duellman & Crump 1974	Duellman & Crump 1974	AM	FZ, ML, JV
Dendropsophus brevifrons	Hylidae	LC	Duellman & Crump 1974	Duellman 1978a	AM	FZ, ML, JV
Dendropsophus brevifrons	Hylidae	LC	Duellman & Crump 1974	Schlüter 1979	AM	FZ, ML, JV
Dendropsophus brevifrons	Hylidae	LC	Duellman & Crump 1974	Duellman & Pyles 1983	AM	FZ, ML, JV
Dendropsophus brevifrons	Hylidae	LC	Duellman & Crump 1974	Lescure & Marty 2000	AM	FZ, ML, JV
Dendropsophus brevifrons	Hylidae	LC	Duellman & Crump 1974	Duellman 2005	AM	FZ, ML, JV
Dendropsophus cachimbo	Hylidae	DD	Napoli & Caramaschi 1999a	Teixeira & Giaretta 2015	AM	FZ, ML, JV
Dendropsophus cerradensis	Hylidae	DD	Napoli & Caramaschi 1998	NA	CE	–
Dendropsophus counani	Hylidae	NE	Fouquet et al. 2015a	Fouquet et al. 2015a	AM	–
Dendropsophus cruzi	Hylidae	LC	Pombal & Bastos 1998	Pombal & Bastos 1998	CE	–
Dendropsophus cruzi	Hylidae	LC	Pombal & Bastos 1998	Bastos et al. 2003	CE	FZ, JV
Dendropsophus cruzi	Hylidae	LC	Pombal & Bastos 1998	Tessarolo et al. 2016	CE	FZ, JV
Dendropsophus decipiens	Hylidae	LC	Lutz 1925b	Abrunhosa et al. 2001	AF	FZ, JV
Dendropsophus dutrai	Hylidae	DD	Gomes & Peixoto 1996	NA	AF, CA	JV
Dendropsophus elegans	Hylidae	LC	Wied-Neuwied 1824c	Bastos & Haddad 1995	AF, CA	–
Dendropsophus elegans	Hylidae	LC	Wied-Neuwied 1824c	Muniz et al. 2016	AF, CA	FZ, JV
Dendropsophus elianeae	Hylidae	LC	Napoli & Caramaschi 2000	Martins & Jim 2004	AF, CE, PN	FZ, JV
Dendropsophus elianeae	Hylidae	LC	Napoli & Caramaschi 2000	Silva et al. 2008	AF, CE, PN	–
Dendropsophus gaucheri	Hylidae	LC	Lescure & Marty 2000	Lescure & Marty 2000	AM	–
Dendropsophus giesleri	Hylidae	LC	Mertens 1950b	Heyer 1980	AF	–
Dendropsophus haddadi	Hylidae	LC	Bastos & Pombal 1996	Ruas et al. 2012a	AF	ML, JV
Dendropsophus haraldschultzi	Hylidae	LC	Bokermann 1962a	Hödl 1977	AM	JV
Dendropsophus jimi	Hylidae	LC	Napoli & Caramaschi 1999b	Martins & Jim 2004	CE	FZ
Dendropsophus koechlini	Hylidae	LC	Duellman & Trueb 1989	Duellman & Trueb 1989	AM	–
Dendropsophus koechlini	Hylidae	LC	Duellman & Trueb 1989	Duellman 2005	AM	FZ, ML

Taxa	Family	IUCN	Description Reference	Call_reference	Biome	Sound Library
Dendropsophus leali	Hylidae	LC	Bokermann 1964b	Márquez et al. 1993	AM	FZ, ML
Dendropsophus leali	Hylidae	LC	Bokermann 1964b	Duellman 2005	AM	FZ, ML, JV
Dendropsophus leucophyllatus	Hylidae	LC	Beireis 1783	Duellman 1974	AM, CE	FZ, ML, JV
Dendropsophus leucophyllatus	Hylidae	LC	Beireis 1783	Duellman 1978a	AM, CE	FZ, ML, JV
Dendropsophus leucophyllatus	Hylidae	LC	Beireis 1783	Duellman & Pyles 1983	AM, CE	FZ, ML, JV
Dendropsophus leucophyllatus	Hylidae	LC	Beireis 1783	Márquez et al. 1993	AM, CE	FZ, ML, JV
Dendropsophus leucophyllatus	Hylidae	LC	Beireis 1783	Lescure & Marty 2000	AM, CE	FZ, ML, JV
Dendropsophus leucophyllatus	Hylidae	LC	Beireis 1783	Duellman 2005	AM, CE	FZ, ML, JV
Dendropsophus leucophyllatus	Hylidae	LC	Beireis 1783	Kaiser & Hammers 2008	AM, CE	FZ, ML, JV
Dendropsophus limai	Hylidae	DD	Bokermann 1962a	NA	AF	FZ, ML, JV
Dendropsophus mapinguari	Hylidae	NE	Peloso et al. 2016	NA	AM	ML
Dendropsophus marmoratus	Hylidae	LC	Laurenti 1768	Duellman 1978a	AM	–
Dendropsophus marmoratus	Hylidae	LC	Laurenti 1768	Duellman & Pyles 1983	AM	FZ, ML
Dendropsophus marmoratus	Hylidae	LC	Laurenti 1768	Zimmerman 1983	AM	FZ, ML
Dendropsophus marmoratus	Hylidae	LC	Laurenti 1768	Zimmerman & Bogart 1984	AM	FZ, ML
Dendropsophus marmoratus	Hylidae	LC	Laurenti 1768	Duellman 2005	AM	FZ, ML
Dendropsophus melanargyreus	Hylidae	LC	Cope 1887	Márquez et al. 1993	AM, CE, PN	FZ, ML
Dendropsophus melanargyreus	Hylidae	LC	Cope 1887	Lescure & Marty 2000	AM, CE, PN	FZ, ML, JV
Dendropsophus meridianus	Hylidae	LC	Lutz 1954	Pombal & Bastos 1998	AF	FZ, ML, JV
Dendropsophus microcephalus	Hylidae	LC	Cope 1886	Schwartz & Wells 1984	AM, CA	JV
Dendropsophus microcephalus	Hylidae	LC	Cope 1886	Schwartz & Wells 1985	AM, CA	FZ, ML, JV
Dendropsophus microcephalus	Hylidae	LC	Cope 1886	Duellman 1997	AM, CA	FZ, ML, JV
Dendropsophus microcephalus	Hylidae	LC	Cope 1886	Kime et al. 2000	AM, CA	FZ, ML, JV
Dendropsophus microcephalus	Hylidae	LC	Cope 1886	Bernal et al. 2004	AM, CA	FZ, ML, JV
Dendropsophus microcephalus	Hylidae	LC	Cope 1886	Tárano 2010	AM, CA	FZ, ML, JV
Dendropsophus microps	Hylidae	LC	Peters 1872b	Heyer et al. 1990	AF, CE, PM	FZ, ML, JV
Dendropsophus microps	Hylidae	LC	Peters 1872b	Kwet 2001	CE, PM	FZ, ML, JV

Taxa	Family	IUCN	Description Reference	Call_reference	Biome	Sound Library
Dendropsophus microps	Hylidae	LC	Peters 1872b	Forti et al. 2015	CE, PM	FZ, ML, JV
Dendropsophus minimus	Hylidae	DD	Ahl 1933	NA	AM	FZ, ML, JV
Dendropsophus minusculus	Hylidae	LC	Rivero 1971	Duellman & Pyles 1983	AM	ML
Dendropsophus minusculus	Hylidae	LC	Rivero 1971	Duellman 1997	AM	FZ, ML, JV
Dendropsophus minusculus	Hylidae	LC	Rivero 1971	Lescure & Marty 2000	AM	FZ, ML, JV
Dendropsophus minusculus	Hylidae	LC	Rivero 1971	Tárano 2010	AM	FZ, ML, JV
Dendropsophus minutus	Hylidae	LC	Peters 1872b	Bokermann 1967f	AM, AF, CA, CE, PM, PN	FZ, ML, JV
Dendropsophus minutus	Hylidae	LC	Peters 1872b	Duellman 1978a	AM, AF, CA, CE, PM, PN	FZ, ML, JV
Dendropsophus minutus	Hylidae	LC	Peters 1872b	Duellman & Pyles 1983	AM, AF, CA, CE, PM, PN	FZ, ML, JV
Dendropsophus minutus	Hylidae	LC	Peters 1872b	Zimmerman 1983	AM, AF, CA, CE, PM, PN	FZ, ML, JV
Dendropsophus minutus	Hylidae	LC	Peters 1872b	Márquez et al. 1993	AM, AF, CA, CE, PM, PN	FZ, ML, JV
Dendropsophus minutus	Hylidae	LC	Peters 1872b	Cardoso & Haddad 1984	AM, AF, CA, CE, PM, PN	FZ, ML, JV
Dendropsophus minutus	Hylidae	LC	Peters 1872b	Haddad et al. 1988	AM, AF, CA, CE, PM, PN	FZ, ML, JV
Dendropsophus minutus	Hylidae	LC	Peters 1872b	Heyer et al. 1990	AM, AF, CA, CE, PM, PN	FZ, ML, JV
Dendropsophus minutus	Hylidae	LC	Peters 1872b	Duellman 1997	AM, AF, CA, CE, PM, PN	FZ, ML, JV
Dendropsophus minutus	Hylidae	LC	Peters 1872b	Lescure & Marty 2000	AM, AF, CA, CE, PM, PN	FZ, ML, JV
Dendropsophus minutus	Hylidae	LC	Peters 1872b	Köhler 2000	AM, AF, CA, CE, PM, PN	FZ, ML, JV
Dendropsophus minutus	Hylidae	LC	Peters 1872b	Kwet 2001	AM, AF, CA, CE, PM, PN	FZ, ML, JV
Dendropsophus minutus	Hylidae	LC	Peters 1872b	Silva et al. 2008	AM, AF, CA, CE, PM, PN	FZ, ML, JV
Dendropsophus minutus	Hylidae	LC	Peters 1872b	Pombal 2010	AM, AF, CA, CE, PM, PN	FZ, ML, JV
Dendropsophus minutus	Hylidae	LC	Peters 1872b	Morais et al. 2012b	AM, AF, CA, CE, PM, PN	FZ, ML, JV
Dendropsophus miyatai	Hylidae	LC	Vigle & Goberdhan-Vigle 1990	NA	AM	FZ, ML, JV
Dendropsophus nahdereri	Hylidae	LC	Lutz & Bokermann 1963	Conte et al. 2010	AF, PM	–
Dendropsophus nanus	Hylidae	LC	Boulenger 1889	Hödl 1977	AM, AF, CA, CE, PM, PN	FZ, JV
Dendropsophus nanus	Hylidae	LC	Boulenger 1889	Márquez et al. 1993	AM, AF, CA, CE, PM, PN	FZ, ML, JV
Dendropsophus nanus	Hylidae	LC	Boulenger 1889	Lescure & Marty 2000	AM, AF, CA, CE, PM, PN	FZ, ML, JV
Dendropsophus nanus	Hylidae	LC	Boulenger 1889	Bastos et al. 2003	AM, AF, CA, CE, PM, PN	FZ, ML, JV

Taxa	Family	IUCN	Description Reference	Call_reference	Biome	Sound Library
Dendropsophus nanus	Hylidae	LC	Boulenger 1889	Martins et al. 2006	AM, AF, CA, CE, PM, PN	FZ, ML, JV
Dendropsophus nanus	Hylidae	LC	Boulenger 1889	Silva et al. 2008	AM, AF, CA, CE, PM, PN	FZ, ML, JV
Dendropsophus nanus	Hylidae	LC	Boulenger 1889	Teixeira et al. 2016	AM, AF, CA, CE, PM, PN	FZ, ML, JV
Dendropsophus novaisi	Hylidae	DD	Bokermann 1968b	NA	AF, CA	FZ, ML, JV
Dendropsophus oliveirai	Hylidae	LC	Bokermann 1963d	Santana et al. 2011a	AF, CA	–
Dendropsophus ozzyi	Hylidae	NE	Orrico et al. 2014	Orrico et al. 2014	AM	JV
Dendropsophus parviceps	Hylidae	LC	Boulenger 1882	Duellman & Crump 1974	AM	–
Dendropsophus parviceps	Hylidae	LC	Boulenger 1882	Duellman 1978a	AM	FZ, ML, JV
Dendropsophus parviceps	Hylidae	LC	Boulenger 1882	Schlüter 1979	AM	FZ, ML, JV
Dendropsophus parviceps	Hylidae	LC	Boulenger 1882	Duellman & Pyles 1983	AM	FZ, ML, JV
Dendropsophus parviceps	Hylidae	LC	Boulenger 1882	Márquez et al. 1993	AM	FZ, ML, JV
Dendropsophus parviceps	Hylidae	LC	Boulenger 1882	Duellman 2005	AM	FZ, ML, JV
Dendropsophus pauiniensis	Hylidae	LC	Heyer 1977	NA	AM	FZ, ML, JV
Dendropsophus pseudomeridianus	Hylidae	LC	Cruz et al. 2000	Capranica 1965	AF	–
Dendropsophus rhea	Hylidae	DD	Napoli & Caramaschi 1999b	NA	CE	JV
Dendropsophus rhodopeplus	Hylidae	LC	Günther 1858	Duellman 1972c	AM	–
Dendropsophus rhodopeplus	Hylidae	LC	Günther 1858	Duellman 1978a	AM	FZ, ML, JV
Dendropsophus rhodopeplus	Hylidae	LC	Günther 1858	Schlüter 1979	AM	FZ, ML, JV
Dendropsophus rhodopeplus	Hylidae	LC	Günther 1858	Duellman & Pyles 1983	AM	FZ, ML, JV
Dendropsophus rhodopeplus	Hylidae	LC	Günther 1858	Márquez et al. 1993	AM	FZ, ML, JV
Dendropsophus rhodopeplus	Hylidae	LC	Günther 1858	Duellman 2005	AM	FZ, ML, JV
Dendropsophus riveroi	Hylidae	LC	Cochran & Goin 1970	Duellman 1978a	AM	FZ, ML, JV
Dendropsophus riveroi	Hylidae	LC	Cochran & Goin 1970	Márquez et al. 1993	AM	FZ, ML, JV
Dendropsophus rossalleni	Hylidae	LC	Goin 1959	Hödl 1977	AM	FZ, ML, JV
Dendropsophus rossalleni	Hylidae	LC	Goin 1959	Schlüter 1979	AM	ML
Dendropsophus rubicundulus	Hylidae	LC	Reinhardt & Lütken 1862	Cardoso & Vielliard 1985	AF, CE	ML
Dendropsophus rubicundulus	Hylidae	LC	Reinhardt & Lütken 1862	Teixeira & Giaretta 2015	AF, CE	FZ, ML, JV

Taxa	Family	IUCN	Description Reference	Call_reference	Biome	Sound Library
Dendropsophus ruschii	Hylidae	DD	Weygoldt & Peixoto 1987	Weygoldt & Peixoto 1987	AF	FZ, ML, JV
Dendropsophus sanborni	Hylidae	LC	Schmidt 1944	Barrio 1964a	AF, CE, PM	–
Dendropsophus sanborni	Hylidae	LC	Schmidt 1944	Martins & Jim 2003	AF, CE, PM	FZ, ML, JV
Dendropsophus sarayacuensis	Hylidae	LC	Schreve 1935	Duellman 1974	AM	FZ, ML, JV
Dendropsophus sarayacuensis	Hylidae	LC	Schreve 1935	Duellman 1978a	AM	FZ, ML, JV
Dendropsophus sarayacuensis	Hylidae	LC	Schreve 1935	Schlüter 1979	AM	FZ, ML, JV
Dendropsophus sarayacuensis	Hylidae	LC	Schreve 1935	Duellman & Pyles 1983	AM	FZ, ML, JV
Dendropsophus sarayacuensis	Hylidae	LC	Schreve 1935	Kaiser & Hammers 2008	AM	FZ, ML, JV
Dendropsophus schubarti	Hylidae	LC	Bokermann 1963a	NA	AM	FZ, ML, JV
Dendropsophus seniculus	Hylidae	LC	Cope 1868	Bokermann 1967f	AF	ML, JV
Dendropsophus seniculus	Hylidae	LC	Cope 1868	Hepp et al. 2012	AF	FZ, JV
Dendropsophus soaresi	Hylidae	LC	Caramaschi & Jim 1983c	Guimarães et al. 2001	AF, CA, CE	FZ, JV
Dendropsophus studerae	Hylidae	DD	Carvalho-e-Silva et al. 2003	Napoli et al. 2015	AF	JV
Dendropsophus timbeba	Hylidae	LC	Martins & Cardoso 1987	Martins & Cardoso 1987	AM	–
Dendropsophus timbeba	Hylidae	LC	Martins & Cardoso 1987	Duellman 2005	AM	ML, JV
Dendropsophus tintinnabulum	Hylidae	DD	Melin 1941	Teixeira & Giaretta 2016	AM	ML, JV
Dendropsophus triangulum	Hylidae	LC	Günther 1869	Duellman 1974	AM	–
Dendropsophus triangulum	Hylidae	LC	Günther 1869	Hödl 1977	AM	FZ, ML, JV
Dendropsophus triangulum	Hylidae	LC	Günther 1869	Duellman 1978a	AM	FZ, ML, JV
Dendropsophus triangulum	Hylidae	LC	Günther 1869	Duellman & Pyles 1983	AM	FZ, ML, JV
Dendropsophus triangulum	Hylidae	LC	Günther 1869	Kaiser & Hammers 2008	AM	FZ, ML, JV
Dendropsophus tritaeniatus	Hylidae	LC	Bokermann 1965d	Teixeira et al. 2013a	CE	FZ, ML, JV
Dendropsophus walfordi	Hylidae	LC	Bokermann 1962c	De La Riva et al. 1997	AM	FZ, JV
Dendropsophus werneri	Hylidae	LC	Cochran 1952	Lingnau et al. 2004	AF	FZ, ML, JV
Dendropsophus xapuriensis	Hylidae	LC	Martins & Cardoso 1987	Martins & Cardoso 1987	AM	FZ, ML, JV
Dryaderces inframaculatus	Hylidae	NE	Boulenger 1882	NA	AM	JV
Dryaderces pearsoni	Hylidae	LC	Gaige 1929	NA	AM	–

Taxa	Family	IUCN	Description Reference	Call_reference	Biome	Sound Library
Boana albomarginatus	Hylidae	LC	Spix 1824	Bokermann 1967f	AF, CA, CE	–
Boana albomarginatus	Hylidae	LC	Spix 1824	Giasson & Haddad 2006	AF, CA, CE	FZ, JV
Boana albopunctatus	Hylidae	LC	Spix 1824	Haddad et al. 1988	AF, CA, CE, PM, PN	FZ, JV
Boana albopunctatus	Hylidae	LC	Spix 1824	Heyer et al. 1990	AF, CA, CE, PM, PN	FZ, ML, JV
Boana albopunctatus	Hylidae	LC	Spix 1824	De La Riva et al. 1997	AF, CA, CE, PM, PN	FZ, ML, JV
Boana albopunctatus	Hylidae	LC	Spix 1824	Bastos et al. 2003	AF, CA, CE, PM, PN	FZ, ML, JV
Boana albopunctatus	Hylidae	LC	Spix 1824	Silva et al. 2008	AF, CA, CE, PM, PN	FZ, ML, JV
Boana albopunctatus	Hylidae	LC	Spix 1824	Vieira et al. 2016	AF, CA, CE, PM, PN	FZ, ML, JV
Boana albopunctatus	Hylidae	LC	Spix 1824	Furtado et al. 2016	AF, CA, CE, PM, PN	FZ, ML, JV
Boana atlanticus	Hylidae	LC	Caramaschi & Velosa 1996	Camurugi & Juncá 2013	AF	FZ, ML, JV
Boana atlanticus	Hylidae	LC	Caramaschi & Velosa 1996	Camurugi et al. 2015	AF	JV
Boana bandeirantes	Hylidae	NE	Caramaschi & Feio 1990	Pinheiro et al. 2012	AF	JV
Boana beckeri	Hylidae	DD	Caramaschi & Cruz 2004	Acioli & Toledo 2008	AF	JV
Boana beckeri	Hylidae	DD	Caramaschi & Cruz 2004	Martins et al. 2016	AF	JV
Boana benitezi	Hylidae	NE	Rivero 1961	Myers & Donnelly 1997	AM	JV
Boana bischoffi	Hylidae	LC	Boulenger 1887	Bokermann 1967f	AF, PM	ML, JV
Boana bischoffi	Hylidae	LC	Boulenger 1887	Heyer et al. 1990	AF, PM	FZ, ML, JV
Boana bischoffi	Hylidae	LC	Boulenger 1887	Kwet 2001	AF, PM	FZ, ML, JV
Boana bischoffi	Hylidae	LC	Boulenger 1887	Toledo et al. 2007a	AF, PM	FZ, ML, JV
Boana bischoffi	Hylidae	LC	Boulenger 1887	Pombal 2010	AF, PM	FZ, ML, JV
Boana boans	Hylidae	LC	Linnaeus 1758	Hödl 1977	AM	FZ, ML, JV
Boana boans	Hylidae	LC	Linnaeus 1758	Duellman 1978a	AM	FZ, ML, JV
Boana boans	Hylidae	LC	Linnaeus 1758	Schlüter 1979	AM	FZ, ML, JV
Boana boans	Hylidae	LC	Linnaeus 1758	Zimmerman 1983	AM	FZ, ML, JV
Boana boans	Hylidae	LC	Linnaeus 1758	Zimmerman & Bogart 1984	AM	FZ, ML, JV
Boana boans	Hylidae	LC	Linnaeus 1758	Lescure & Marty 2000	AM	FZ, ML, JV
Boana boans	Hylidae	LC	Linnaeus 1758	Duellman 2005	AM	FZ, ML, JV

Taxa	Family	IUCN	Description Reference	Call_reference	Biome	Sound Library
Boana botumirim	Hylidae	NE	Caramaschi et al. 2009b	Caramaschi et al. 2009b	CE	FZ, ML, JV
Boana buriti	Hylidae	DD	Caramaschi & Cruz 1999	NA	CE	–
Boana caingua	Hylidae	LC	Carrizo 1991	Batista et al. 2015	AF, CE, PM	–
Boana caipora	Hylidae	NE	Antunes et al. 2008	Antunes et al. 2008	AF	FZ, JV
Boana calcaratus	Hylidae	LC	Troschel 1848	Duellman 1973	AM	JV
Boana calcaratus	Hylidae	LC	Troschel 1848	Duellman 1978a	AM	FZ, ML, JV
Boana calcaratus	Hylidae	LC	Troschel 1848	Duellman & Pyles 1983	AM	FZ, ML, JV
Boana calcaratus	Hylidae	LC	Troschel 1848	Lescure & Marty 2000	AM	FZ, ML, JV
Boana calcaratus	Hylidae	LC	Troschel 1848	Duellman 2005	AM	FZ, ML, JV
Boana calcaratus	Hylidae	LC	Troschel 1848	Caminer & Ron 2014	AM	FZ, ML, JV
Boana cambui	Hylidae	NE	Pinheiro et al. 2016	Pinheiro et al. 2016	AF	FZ, ML, JV
Boana cinerascens	Hylidae	LC	Spix 1824	Duellman 1978a	AM	–
Boana cinerascens	Hylidae	LC	Spix 1824	Schlüter 1979	AM	FZ, ML, JV
Boana cinerascens	Hylidae	LC	Spix 1824	Duellman & Pyles 1983	AM	FZ, ML, JV
Boana cinerascens	Hylidae	LC	Spix 1824	Zimmerman 1983	AM	FZ, ML, JV
Boana cinerascens	Hylidae	LC	Spix 1824	Zimmerman & Bogart 1984	AM	FZ, ML, JV
Boana cinerascens	Hylidae	LC	Spix 1824	Cardoso & Vielliard 1990	AM	FZ, ML, JV
Boana cinerascens	Hylidae	LC	Spix 1824	De La Riva et al. 1997	AM	FZ, ML, JV
Boana cinerascens	Hylidae	LC	Spix 1824	Lescure & Marty 2000	AM	FZ, ML, JV
Boana cinerascens	Hylidae	LC	Spix 1824	Duellman 2005	AM	FZ, ML, JV
Boana cipoensis	Hylidae	NT	Lutz 1968a	Haddad et al. 1988	CE	FZ, ML, JV
Boana crepitans	Hylidae	LC	Wied-Neuwied 1824b	Kime et al. 2000	AM, AF, CA, CE	JV
Boana crepitans	Hylidae	LC	Wied-Neuwied 1824b	Bernal et al. 2004	AM, AF, CA, CE	FZ, ML, JV
Boana crepitans	Hylidae	LC	Wied-Neuwied 1824b	Casal & Juncá 2008	AM, AF, CA, CE	FZ, ML, JV
Boana crepitans	Hylidae	LC	Wied-Neuwied 1824b	Martins et al. 2009	AM, AF, CA, CE	FZ, ML, JV
Boana curupi	Hylidae	LC	Garcia et al. 2007	Garcia et al. 2007	AF	FZ, ML, JV
Boana cymbalum	Hylidae	CR	Bokermann 1963e	NA	AF	–

Taxa	Family	IUCN	Description Reference	Call_reference	Biome	Sound Library
Boana dentei	Hylidae	LC	Bokermann 1967i	Lescure & Marty 2000	AM	JV
Boana diabolica	Hylidae	NE	Fouquet et al. 2016	Fouquet et al. 2016	AM	–
Boana ericae	Hylidae	DD	Caramaschi & Cruz 2000	Garcia & Haddad 2008	CE	–
Boana exastis	Hylidae	DD	Caramaschi & Rodriguez 2003	Loebmann et al. 2008	AF, CA	FZ
Boana faber	Hylidae	LC	Wied-Neuwied 1821a	Martins & Haddad 1988	AF, CA, CE, PM	JV
Boana faber	Hylidae	LC	Wied-Neuwied 1821a	Kwet 2001	AF, CA, CE, PM	FZ, ML, JV
Boana faber	Hylidae	LC	Wied-Neuwied 1821a	Pombal 2010	AF, CA, CE, PM	FZ, ML, JV
Boana fasciatus	Hylidae	LC	Günther 1858	Duellman 1973	AM	FZ, ML, JV
Boana fasciatus	Hylidae	LC	Günther 1858	Duellman 1978a	AM	FZ, ML, JV
Boana fasciatus	Hylidae	LC	Günther 1858	Márquez et al. 1993	AM	FZ, ML, JV
Boana fasciatus	Hylidae	LC	Günther 1858	Lescure & Marty 2000	AM	FZ, ML, JV
Boana fasciatus	Hylidae	LC	Günther 1858	Duellman 2005	AM	FZ, ML, JV
Boana fasciatus	Hylidae	LC	Günther 1858	Caminer & Ron 2014	AM	FZ, ML, JV
Boana freicanecae	Hylidae	DD	Carnaval & Peixoto 2004	NA	AF	FZ, ML, JV
Boana geographicus	Hylidae	LC	Spix 1824	Duellman 1973	AM, AF, CE, PN	–
Boana geographicus	Hylidae	LC	Spix 1824	Duellman 1978a	AM, AF, CE, PN	FZ, ML, JV
Boana geographicus	Hylidae	LC	Spix 1824	Duellman & Pyles 1983	AM, AF, CE, PN	FZ, ML, JV
Boana geographicus	Hylidae	LC	Spix 1824	Zimmerman 1983	AM, AF, CE, PN	FZ, ML, JV
Boana geographicus	Hylidae	LC	Spix 1824	Zimmerman & Bogart 1984	AM, AF, CE, PN	FZ, ML, JV
Boana geographicus	Hylidae	LC	Spix 1824	Márquez et al. 1993	AM, AF, CE, PN	FZ, ML, JV
Boana geographicus	Hylidae	LC	Spix 1824	Lescure & Marty 2000	AM, AF, CE, PN	FZ, ML, JV
Boana geographicus	Hylidae	LC	Spix 1824	Duellman 2005	AM, AF, CE, PN	FZ, ML, JV
Boana goianus	Hylidae	LC	Lutz 1968a	Bastos et al. 2003	CE	FZ, ML, JV
Boana goianus	Hylidae	LC	Lutz 1968a	Menin et al. 2004	CE	FZ, JV
Boana goianus	Hylidae	LC	Lutz 1968a	Morais et al. 2015	CE	FZ, JV
Boana goianus	Hylidae	LC	Lutz 1968a	Furtado et al. 2016	CE	FZ, JV
Boana goianus	Hylidae	LC	Lutz 1968a	Signorelli et al. 2016	CE	FZ, JV

Taxa	Family	IUCN	Description Reference	Call_reference	Biome	Sound Library
Boana guentheri	Hylidae	LC	Günther 1869	NA	AF, PM	FZ, JV
Boana jaguariaivensis	Hylidae	NE	Caramaschi et al. 2010	NA	CE	FZ, JV
Boana joaquini	Hylidae	LC	Lutz 1968b	Garcia et al. 2003	AF, PM	–
Boana lanciformis	Hylidae	LC	Cope 1871b	Hödl 1977	AM	FZ, JV
Boana lanciformis	Hylidae	LC	Cope 1871b	Duellman 1978a	AM	FZ, ML, JV
Boana lanciformis	Hylidae	LC	Cope 1871b	Duellman & Pyles 1983	AM	FZ, ML, JV
Boana lanciformis	Hylidae	LC	Cope 1871b	Cardoso & Vielliard 1990	AM	FZ, ML, JV
Boana lanciformis	Hylidae	LC	Cope 1871b	Márquez et al. 1993	AM	FZ, ML, JV
Boana latistriatus	Hylidae	DD	Caramaschi & Cruz 2004	NA	AF	FZ, ML, JV
Boana leptolineatus	Hylidae	LC	Braun & Braun 1977a	Kwet 2001	AF, PM	JV
Boana leucocheilus	Hylidae	DD	Caramaschi & Niemeyer 2003a	Pansonato et al. 2011	AM	FZ, JV
Boana lundii	Hylidae	LC	Burmeister 1856	Bokermann & Sazima 1973a	CA, CE	–
Boana lundii	Hylidae	LC	Burmeister 1856	Guimarães et al. 2001	CA, CE	FZ, JV
Boana marginatus	Hylidae	LC	Boulenger 1887	Garcia et al. 2001b	AF	FZ, JV
Boana marginatus	Hylidae	LC	Boulenger 1887	Kwet 2001	AF	JV
Boana microderma	Hylidae	LC	Pyburn 1977	Pyburn 1977	AM	JV
Boana microderma	Hylidae	LC	Pyburn 1977	Cardoso & Vielliard 1990	AM	FZ, JV
Boana multifasciatus	Hylidae	LC	Günther 1859	Duellman & Pyles 1983	AM, CE	FZ, JV
Boana multifasciatus	Hylidae	LC	Günther 1859	Duellman 1997	AM, CE	FZ, ML, JV
Boana multifasciatus	Hylidae	LC	Günther 1859	Lescure & Marty 2000	AM, CE	FZ, ML, JV
Boana multifasciatus	Hylidae	LC	Günther 1859	Carvalho et al. 2010a	AM, CE	FZ, ML, JV
Boana nympha	Hylidae	LC	Faivovich et al. 2006	NA	AM	FZ, ML, JV
Boana ornatissimus	Hylidae	LC	Noble 1923	Lescure & Marty 2000	AM	–
Boana pardalis	Hylidae	LC	Spix 1824	Heyer et al. 1990	AF	–
Boana paranaiba	Hylidae	NE	Carvalho et al. 2010a	Guimarães et al. 2001	CE	FZ, ML, JV
Boana paranaiba	Hylidae	NE	Carvalho et al. 2010a	Carvalho et al. 2010a	CE	–
Boana paranaiba	Hylidae	NE	Carvalho et al. 2010a	Vieira et al. 2016	CE	–

Taxa	Family	IUCN	Description Reference	Call_reference	Biome	Sound Library
Boana phaeopleura	Hylidae	DD	Caramaschi & Cruz 2000	Pinheiro et al. 2012	CE	–
Boana poaju	Hylidae	NE	Garcia et al. 2008	Garcia et al. 2008	AF	–
Boana polytaenius	Hylidae	LC	Cope 1870	Bokermann 1967f	AF, CA	JV
Boana polytaenius	Hylidae	LC	Cope 1870	Heyer et al. 1990	AF, CA	FZ, ML, JV
Boana polytaenius	Hylidae	LC	Cope 1870	Pinheiro et al. 2012	AF, CA	FZ, ML, JV
Boana pombali	Hylidae	LC	Caramaschi et al. 2004	Caramaschi et al. 2004	AF, CA	FZ, ML, JV
Boana prasinus	Hylidae	LC	Burmeister 1856	Pombal 2010	AF, CE, PM	JV
Boana prasinus	Hylidae	LC	Burmeister 1856	Delgado & Haddad 2015	AF, CE, PM	FZ, JV
Boana pulchellus	Hylidae	LC	Duméril & Bibron 1841	Márquez et al. 1993	AF, PM	FZ, JV
Boana pulchellus	Hylidae	LC	Duméril & Bibron 1841	Kwet 2001	AF, PM	FZ, ML, JV
Boana punctatus	Hylidae	LC	Schneider 1799	Hödl 1977	AM, AF, CE, PN	FZ, ML, JV
Boana punctatus	Hylidae	LC	Schneider 1799	Duellman 1978a	AM, AF, CE, PN	FZ, ML, JV
Boana punctatus	Hylidae	LC	Schneider 1799	Duellman & Pyles 1983	AM, AF, CE, PN	FZ, ML, JV
Boana punctatus	Hylidae	LC	Schneider 1799	Márquez et al. 1993	AM, AF, CE, PN	FZ, ML, JV
Boana punctatus	Hylidae	LC	Schneider 1799	Cardoso & Vielliard 1990	AM, AF, CE, PN	FZ, ML, JV
Boana punctatus	Hylidae	LC	Schneider 1799	Lescure & Marty 2000	AM, AF, CE, PN	FZ, ML, JV
Boana punctatus	Hylidae	LC	Schneider 1799	Duellman 2005	AM, AF, CE, PN	FZ, ML, JV
Boana punctatus	Hylidae	LC	Schneider 1799	Brunetti e al. 2015	AM, AF, CE, PN	FZ, ML, JV
Boana raniceps	Hylidae	LC	Cope 1862b	Hödl 1977	AM, AF, CA, CE, PN	FZ, ML, JV
Boana raniceps	Hylidae	LC	Cope 1862b	Márquez et al. 1993	AM, AF, CA, CE, PN	FZ, ML, JV
Boana raniceps	Hylidae	LC	Cope 1862b	Lescure & Marty 2000	AM, AF, CA, CE, PN	FZ, ML, JV
Boana raniceps	Hylidae	LC	Cope 1862b	Guimarães & Bastos 2003	AM, AF, CA, CE, PN	FZ, ML, JV
Boana secedens	Hylidae	DD	Lutz 1963	NA	AF	FZ, ML, JV
Boana semiguttatus	Hylidae	LC	Lutz 1925b	Kwet 2001	AF	–
Boana semiguttatus	Hylidae	LC	Lutz 1925b	Garcia et al. 2007	AF	FZ, JV
Boana semilineatus	Hylidae	NE	Spix 1824	Lingnau & Bastos 2003	AF	FZ, JV
Boana stellae	Hylidae	LC	Kwet 2008	Kwet 2008	AF, PM	JV

Taxa	Family	IUCN	Description Reference	Call_reference	Biome	Sound Library
Boana stenocephalus	Hylidae	DD	Caramaschi & Cruz 1999	Martins et al. 2016	AF, CE	–
Boana tepuianus	Hylidae	NE	Barrio-Amoros & Brewer-Carias 2008	Barrio-Amoros & Brewer-Carias 2008	AM	–
Boana wavrini	Hylidae	LC	Parker 1936	Hoogmoed 1990	AM	–
Itapotihyla langsdorffii	Hylidae	LC	Duméril & Bibron 1841	NA	AF, CE	–
Julianus pinimus	Hylidae	DD	Bokermann & Sazima 1973b	Bokermann & Sazima 1973b	CE	FZ, JV
Julianus uruguayus	Hylidae	LC	Schmidt 1944	Kwet 2001	AF, PM	JV
Julianus uruguayus	Hylidae	LC	Schmidt 1944	Kolenc et al. 2003	AF, PM	FZ, JV
Lysapsus bolivianus	Hylidae	NE	Gallardo 1961b	Santana et al. 2013a	AM	FZ, JV
Lysapsus caraya	Hylidae	LC	Gallardo 1964	Bastos et al. 2011b	CE	–
Lysapsus caraya	Hylidae	LC	Gallardo 1964	Santana et al. 2013a	CE	–
Lysapsus laevis	Hylidae	LC	Parker 1935	Hödl 1977	AM	–
Lysapsus laevis	Hylidae	LC	Parker 1935	Santana et al. 2013a	AM	FZ, ML
Lysapsus limellum	Hylidae	LC	Cope 1862a	Santana et al. 2013a	PM, PN	FZ, ML
Ololygon agilis	Hylidae	LC	Cruz & Peixoto 1983	Nunes et al. 2007b	AF	FZ, ML, JV
Ololygon albicans	Hylidae	LC	Bokermann 1967a	Heyer 1980	AF	–
Ololygon albicans	Hylidae	LC	Bokermann 1967a	Pombal et al. 1995b	AF	JV
Ololygon alcatraz	Hylidae	CR	Lutz 1973	NA	AF	JV
Ololygon angrensis	Hylidae	LC	Lutz 1973	Garey et al. 2012b	AF	JV
Ololygon arduous	Hylidae	DD	Peixoto 2002	Pombal & Bastos 2003	AF	JV
Ololygon arduous	Hylidae	DD	Peixoto 2002	Lacerda et al. 2015	AF	–
Ololygon argyreornata	Hylidae	LC	Miranda-Ribeiro 1926	Pombal et al. 1995b	AF, CA	–
Ololygon ariadne	Hylidae	DD	Bokermann 1967a	NA	AF	FZ, JV
Ololygon aromothyella	Hylidae	DD	Faivovich 2005	Pereyra et al. 2012	AF, PM	–
Ololygon atrata	Hylidae	DD	Peixoto 1989	NA	AF	FZ, JV
Ololygon belloni	Hylidae	EN	Faivoivch et al. 2010	Peres & Simon 2011	AF	–
Ololygon berthae	Hylidae	LC	Barrio 1962	Barrio 1962	AF, PM	–
Ololygon berthae	Hylidae	LC	Barrio 1962	Barrio 1964b	AF, PM	FZ, JV

Taxa	Family	IUCN	Description Reference	Call_reference	Biome	Sound Library
Ololygon berthae	Hylidae	LC	Barrio 1962	Kwet 2001	AF, PM	FZ, JV
Ololygon berthae	Hylidae	LC	Barrio 1962	Pereyra et al. 2012	AF, PM	FZ, JV
Ololygon brieni	Hylidae	LC	De Witte 1930	NA	AF	FZ, JV
Ololygon canastrensis	Hylidae	DD	Cardoso & Haddad 1982	Cardoso & Haddad 1982	CE	–
Ololygon carnevallii	Hylidae	LC	Caramaschi & Kisteumacher 1989b	NA	AF	FZ, JV
Ololygon catharinae	Hylidae	LC	Boulenger 1888a	Heyer 1980	AF, PM	JV
Ololygon catharinae	Hylidae	LC	Boulenger 1888a	Kwet 2001	AF, PM	FZ, JV
Ololygon centralis	Hylidae	LC	Pombal & Bastos 1996	Pombal & Bastos 1996	CE	FZ, JV
Ololygon centralis	Hylidae	LC	Pombal & Bastos 1996	Bastos et al. 2011a	CE	JV
Ololygon cosenzai	Hylidae	NE	Lacerda et al. 2012	Lacerda et al. 2012	AF	JV
Ololygon faivovichi	Hylidae	CR	Brasileiro et al. 2007c	NA	AF	JV
Ololygon flavoguttata	Hylidae	LC	Lutz & Lutz 1939	NA	AF	–
Ololygon heyeri	Hylidae	DD	Peixoto & Weygoldt 1987	Peixoto & Weygoldt 1987	AF	–
Ololygon hiemalis	Hylidae	LC	Haddad & Pombal 1987	Haddad & Pombal 1987	AF, CE	–
Ololygon hiemalis	Hylidae	LC	Haddad & Pombal 1987	Bevier et al. 2008	AF, CE	JV
Ololygon humilis	Hylidae	LC	Lutz 1954	NA	AF	JV
Ololygon inesperata	Hylidae	NE	Silva & Alves-Silva 2011	Silva & Alves-Silva 2011	AF	JV
Ololygon inesperata	Hylidae	NE	Silva & Alves-Silva 2011	Pontes et al. 2013	AF	–
Ololygon jureia	Hylidae	DD	Pombal & Gordo 1991	NA	AF	–
Ololygon kautskyi	Hylidae	DD	Carvalho-e-Silva & Peixoto 1991	NA	AF	–
Ololygon littoralis	Hylidae	LC	Pombal & Gordo 1991	Garey et al. 2012b	AF	–
Ololygon littoreus	Hylidae	LC	Peixoto 1988a	Pontes et al. 2013	AF	JV
Ololygon longilinea	Hylidae	LC	Lutz 1968b	NA	AF, CE	–
Ololygon luizotavioi	Hylidae	LC	Caramaschi & Kisteumacher 1989b	Lourenço et al. 2009a	AF	–
Ololygon machadoi	Hylidae	LC	Bokermann & Sazima 1973b	Bokermann & Sazima 1973b	AF, CE	–
Ololygon melloi	Hylidae	DD	Peixoto 1989	NA	AF	JV
Ololygon muriciensis	Hylidae	CR	Cruz et al. 2011	NA	AF	–

Taxa	Family	IUCN	Description Reference	Call_reference	Biome	Sound Library
Ololygon obtriangulata	Hylidae	LC	Lutz 1968a	NA	AF	–
Ololygon peixotoi	Hylidae	CR	Brasileiro et al. 2007a	Brasileiro et al. 2007a	AF	JV
Ololygon perpusilla	Hylidae	CR	Lutz & Lutz 1939	Heyer et al. 1990	AF	–
Ololygon perpusilla	Hylidae	CR	Lutz & Lutz 1939	Pombal & Bastos 2003	AF	FZ, JV
Ololygon pombali	Hylidae	NE	Lourenço et al. 2013	NA	CE	FZ, JV
Ololygon ranki	Hylidae	DD	Andrade & Cardoso 1987	Andrade & Cardoso 1987	AF	–
Ololygon rizibilis	Hylidae	LC	Bokermann 1964d	Bokermann 1964d	AF, PM	JV
Ololygon rizibilis	Hylidae	LC	Bokermann 1964d	Pombal et al. 1995b	AF, PM	FZ, JV
Ololygon rizibilis	Hylidae	LC	Bokermann 1964d	Bastos & Haddad 1999	AF, PM	FZ, JV
Ololygon rizibilis	Hylidae	LC	Bokermann 1964d	Bevier et al. 2008	AF, PM	FZ, JV
Ololygon rizibilis	Hylidae	LC	Bokermann 1964d	Pombal 2010	AF, PM	FZ, JV
Ololygon skaios	Hylidae	NE	Pombal et al. 2010	Pombal et al. 2010	CE	FZ, JV
Ololygon skuki	Hylidae	EN	Lima et al. 2011	NA	AF	–
Ololygon strigilata	Hylidae	DD	Spix 1824	Mendes et al. 2013	AF, CA	–
Ololygon tripui	Hylidae	NE	Lourenço et al. 2009b	NA	AF	–
Ololygon tupinamba	Hylidae	NE	Silva & Alves-Silva 2008	NA	AF	JV
Ololygon v-signatus	Hylidae	LC	Lutz 1968a	Peixoto et al. 2016	AF	–
Osteocephalus buckleyi	Hylidae	LC	Boulenger 1882	Zimmerman & Bogart 1988	AM	JV
Osteocephalus buckleyi	Hylidae	LC	Boulenger 1882	Ron et al. 2012	AM	FZ, ML
Osteocephalus cabrerai	Hylidae	LC	Cochran & Goin 1970	NA	AM	FZ, ML
Osteocephalus camufatus	Hylidae	NE	Jungfer et al. 2016	NA	AM	FZ
Osteocephalus castaneicola	Hylidae	LC	Moravec et al. 2009a	NA	AM	–
Osteocephalus helenae	Hylidae	DD	Ruthven 1919	NA	AM	–
Osteocephalus leprieurii	Hylidae	LC	Duméril & Bibron 1841	Schlüter 1979	AM	–
Osteocephalus leprieurii	Hylidae	LC	Duméril & Bibron 1841	De La Riva et al. 1995	AM	FZ, ML
Osteocephalus leprieurii	Hylidae	LC	Duméril & Bibron 1841	Lescure & Marty 2000	AM	FZ, ML
Osteocephalus leprieurii	Hylidae	LC	Duméril & Bibron 1841	Jungfer & Hödl 2002	AM	FZ, ML

Taxa	Family	IUCN	Description Reference	Call_reference	Biome	Sound Library
Osteocephalus leprieurii	Hylidae	LC	Duméril & Bibron 1841	Duellman 2005	AM	FZ, ML
Osteocephalus oophagus	Hylidae	LC	Jungfer & Schiesari 1995	Zimmerman 1983	AM	FZ, ML
Osteocephalus oophagus	Hylidae	LC	Jungfer & Schiesari 1995	Zimmerman & Bogart 1984	AM	FZ
Osteocephalus oophagus	Hylidae	LC	Jungfer & Schiesari 1995	Zimmerman & Bogart 1988	AM	FZ
Osteocephalus oophagus	Hylidae	LC	Jungfer & Schiesari 1995	Lescure & Marty 2000	AM	FZ
Osteocephalus planiceps	Hylidae	LC	Cope 1874	Ron & Pramuk 1999	AM	FZ
Osteocephalus subtilis	Hylidae	LC	Martins & Cardoso 1987	Cardoso & Vielliard 1990	AM	FZ
Osteocephalus taurinus	Hylidae	LC	Steindachner 1862	Duellman & Lescure 1973	AM, CE	–
Osteocephalus taurinus	Hylidae	LC	Steindachner 1862	Schlüter 1979	AM, CE	FZ, ML, JV
Osteocephalus taurinus	Hylidae	LC	Steindachner 1862	Zimmerman 1983	AM, CE	FZ, ML, JV
Osteocephalus taurinus	Hylidae	LC	Steindachner 1862	Zimmerman & Bogart 1984	AM, CE	FZ, ML, JV
Osteocephalus taurinus	Hylidae	LC	Steindachner 1862	De La Riva et al. 1995	AM, CE	FZ, ML, JV
Osteocephalus taurinus	Hylidae	LC	Steindachner 1862	Lescure & Marty 2000	AM, CE	FZ, ML, JV
Osteocephalus taurinus	Hylidae	LC	Steindachner 1862	Duellman 2005	AM, CE	FZ, ML, JV
Osteocephalus vilarsi	Hylidae	NE	Melin 1941	NA	AM	FZ, ML, JV
Phyllodytes acuminatus	Hylidae	LC	Bokermann 1966g	Campos et al. 2014	AF	–
Phyllodytes brevirostris	Hylidae	DD	Peixoto & Cruz 1988	NA	AF	–
Phyllodytes edelmoi	Hylidae	DD	Peixoto et al. 2003	Lima et al. 2008	AF	–
Phyllodytes gyrinaethes	Hylidae	DD	Peixoto et al. 2003	Roberto & Ávila 2013	AF	JV
Phyllodytes kautskyi	Hylidae	LC	Peixoto & Cruz 1988	Simon & Gasparini 2003	AF	–
Phyllodytes luteolus	Hylidae	LC	Wied-Neuwied 1824a	Weygoldt 1981	AF, CA	JV
Phyllodytes maculosus	Hylidae	DD	Cruz et al. 2007b	NA	AF	FZ, JV
Phyllodytes melanomystax	Hylidae	LC	Cardoso & Andrade 1982	Nunes et al. 2007b	CE	–
Phyllodytes punctatus	Hylidae	DD	Caramaschi & Peixoto 2004	NA	AF	–
Phyllodytes tuberculosus	Hylidae	DD	Bokermann 1966g	Juncá et al. 2012b	AF, CA	JV
Phyllodytes wuchereri	Hylidae	DD	Peters 1873	Cruz et al. 2014	AF, CA	JV
Phyllodytes wuchereri	Hylidae	DD	Peters 1873	Magalhães et al. 2015b	AF, CA	JV

Taxa	Family	IUCN	Description Reference	Call_reference	Biome	Sound Library
Pseudis bolbodactyla	Hylidae	LC	Lutz 1925a	Guimarães et al. 2001	AF, CA, CE	FZ
Pseudis bolbodactyla	Hylidae	LC	Lutz 1925a	Vaz-Silva et al. 2007	AF, CA, CE	FZ
Pseudis cardosoi	Hylidae	LC	Kwet 2000	Kwet 2000	AF, PM	FZ, JV
Pseudis cardosoi	Hylidae	LC	Kwet 2000	Conte et al. 2010	AF, PM	FZ, JV
Pseudis fusca	Hylidae	LC	Garman 1883	Santana et al. 2016	AF, CA	–
Pseudis minuta	Hylidae	LC	Günther 1858	Kwet 2000	AF, PM	FZ, JV
Pseudis minuta	Hylidae	LC	Günther 1858	Zank et al. 2008	AF, PM	FZ, JV
Pseudis paradoxa	Hylidae	LC	Linnaeus 1758	Bosch et al. 1996	AM, AF	FZ, ML, JV
Pseudis paradoxa	Hylidae	LC	Linnaeus 1758	Lescure & Marty 2000	AM, AF	FZ, ML, JV
Pseudis paradoxa	Hylidae	LC	Linnaeus 1758	Duellman 2005	AM, AF	FZ, ML, JV
Pseudis paradoxa	Hylidae	LC	Linnaeus 1758	Tárano 2010	AM, AF	FZ, ML, JV
Pseudis platensis	Hylidae	DD	Gallardo 1961b	NA	CE, PN	–
Pseudis tocantins	Hylidae	LC	Caramaschi & Cruz 1998	Santana et al. 2016	CE	–
Scarthyla goinorum	Hylidae	LC	Bokermann 1962a	Duellman & Sá 1988	AM	FZ, ML, JV
Scarthyla goinorum	Hylidae	LC	Bokermann 1962a	Duellman 2005	AM	FZ, ML, JV
Scarthyla goinorum	Hylidae	LC	Bokermann 1962a	Barrio-Amorós et al. 2006	AM	FZ, ML, JV
Scinax acuminatus	Hylidae	LC	Cope 1862b	Magrini et al. 2011	CE, PN	–
Scinax alter	Hylidae	LC	Lutz 1973	Nunes et al. 2012	AF, CA	FZ, JV
Scinax auratus	Hylidae	LC	Wied-Neuwied 1821a	Nunes et al. 2007b	AF, CA	JV
Scinax auratus	Hylidae	LC	Wied-Neuwied 1821a	Bevier et al. 2008	AF, CA	JV
Scinax baumgardneri	Hylidae	DD	Rivero 1961	NA	AM	–
Scinax blairi	Hylidae	LC	Fouquette & Pyburn 1972	Fouquette & Pyburn 1972	AM	ML
Scinax boesemani	Hylidae	LC	Goin 1966	Hödl 1977	AM	FZ, ML, JV
Scinax boesemani	Hylidae	LC	Goin 1966	Duellman & Pyles 1983	AM	FZ, ML, JV
Scinax boesemani	Hylidae	LC	Goin 1966	Lescure & Marty 2000	AM	FZ, ML, JV
Scinax cabralensis	Hylidae	DD	Drummond et al. 2007	Drummond et al. 2007	AF, CE	–
Scinax caissara	Hylidae	NE	Lourenço et al. 2016	NA	AF	–

Taxa	Family	IUCN	Description Reference	Call_reference	Biome	Sound Library
Scinax caldarum	Hylidae	LC	Lutz 1968b	Magrini & Giaretta 2010	AF	–
Scinax camposseabrai	Hylidae	DD	Bokermann 1966f	Novaes & Zina 2016	CA	–
Scinax cardosoi	Hylidae	LC	Carvalho-e-Silva & Peixoto 1991	Carvalho et al. 2015a	AF	–
Scinax constrictus	Hylidae	LC	Lima et al. 2005	Lima et al. 2005	CE	–
Scinax constrictus	Hylidae	LC	Lima et al. 2005	Gambale et al. 2013	CE	–
Scinax cretatus	Hylidae	LC	Nunes & Pombal 2011	Nunes & Pombal 2011	AF, CA	JV
Scinax crospedospilus	Hylidae	LC	Lutz 1925b	Heyer et al. 1990	AF	FZ, ML, JV
Scinax crospedospilus	Hylidae	LC	Lutz 1925b	Bevier et al. 2008	AF	FZ, ML, JV
Scinax crospedospilus	Hylidae	LC	Lutz 1925b	Magrini et al. 2011	AF	FZ, ML, JV
Scinax cruentommus	Hylidae	LC	Duellman 1972a	Duellman 1972a	AM	FZ, JV
Scinax cruentommus	Hylidae	LC	Duellman 1972a	Duellman 1978a	AM	FZ, JV
Scinax cruentommus	Hylidae	LC	Duellman 1972a	Duellman & Pyles 1983	AM	FZ, JV
Scinax cruentommus	Hylidae	LC	Duellman 1972a	Lescure & Marty 2000	AM	FZ, JV
Scinax cruentommus	Hylidae	LC	Duellman 1972a	Carvalho et al. 2015b	AM	FZ, JV
Scinax curicica	Hylidae	DD	Pugliese et al. 2004	Pugliese et al. 2004	AF, CE	–
Scinax cuspidatus	Hylidae	LC	Lutz 1925b	Pombal et al. 1995b	AF	FZ, JV
Scinax dolloi	Hylidae	NE	Werner 1903	NA	AF	–
Scinax duartei	Hylidae	LC	Lutz 1951	Bokermann 1967h	AF	JV
Scinax duartei	Hylidae	LC	Lutz 1951	Magrini et al. 2011	AF	JV
Scinax eurydice	Hylidae	LC	Bokermann 1968b	Pombal et al. 1995b	AF, CA	FZ, JV
Scinax eurydice	Hylidae	LC	Bokermann 1968b	Magrini et al. 2011	AF, CA	FZ, JV
Scinax exiguus	Hylidae	LC	Duellman 1986	Duellman 1986	AM	FZ, ML
Scinax funereus	Hylidae	LC	Cope 1874	NA	AM	FZ, JV
Scinax fuscomarginatus	Hylidae	LC	Lutz 1925a	Duellman & Pyles 1983	AM, AF, CA, CE, PN	FZ, ML, JV
Scinax fuscomarginatus	Hylidae	LC	Lutz 1925a	De La Riva et al. 1994	AM, AF, CA, CE, PN	FZ, ML, JV
Scinax fuscomarginatus	Hylidae	LC	Lutz 1925a	Pombal et al. 1995b	AM, AF, CA, CE, PN	FZ, ML, JV
Scinax fuscomarginatus	Hylidae	LC	Lutz 1925a	Bastos et al. 2003	AM, AF, CA, CE, PN	FZ, ML, JV

Taxa	Family	IUCN	Description Reference	Call_reference	Biome	Sound Library
Scinax fuscomarginatus	Hylidae	LC	Lutz 1925a	Toledo & Haddad 2004	AM, AF, CA, CE, PN	FZ, ML, JV
Scinax fuscomarginatus	Hylidae	LC	Lutz 1925a	Silva et al. 2008	AM, AF, CA, CE, PN	FZ, ML, JV
Scinax fuscomarginatus	Hylidae	LC	Lutz 1925a	Pombal 2010	AM, AF, CA, CE, PN	FZ, ML, JV
Scinax fuscovarius	Hylidae	LC	Lutz 1925b	De La Riva et al. 1994	CE, PM, PN	FZ, ML, JV
Scinax fuscovarius	Hylidae	LC	Lutz 1925b	Pombal et al. 1995b	CE, PM, PN	FZ, ML, JV
Scinax fuscovarius	Hylidae	LC	Lutz 1925b	Köhler 2000	CE, PM, PN	FZ, ML, JV
Scinax fuscovarius	Hylidae	LC	Lutz 1925b	Kwet 2001	CE, PM, PN	FZ, ML, JV
Scinax fuscovarius	Hylidae	LC	Lutz 1925b	Silva et al. 2008	CE, PM, PN	FZ, ML, JV
Scinax fuscovarius	Hylidae	LC	Lutz 1925b	Bevier et al. 2008	CE, PM, PN	FZ, ML, JV
Scinax garbei	Hylidae	LC	Miranda-Ribeiro 1926	Duellman 1972b	AM	FZ, ML, JV
Scinax garbei	Hylidae	LC	Miranda-Ribeiro 1926	Duellman 1978a	AM	FZ, ML, JV
Scinax garbei	Hylidae	LC	Miranda-Ribeiro 1926	Duellman & Pyles 1983	AM	FZ, ML, JV
Scinax garbei	Hylidae	LC	Miranda-Ribeiro 1926	Zimmerman 1983	AM	FZ, ML, JV
Scinax garbei	Hylidae	LC	Miranda-Ribeiro 1926	Duellman & Wiens 1993	AM	FZ, ML, JV
Scinax garbei	Hylidae	LC	Miranda-Ribeiro 1926	Duellman 2005	AM	FZ, ML, JV
Scinax granulatus	Hylidae	LC	Peters 1871	Kwet 2001	AF, PM	FZ, JV
Scinax granulatus	Hylidae	LC	Peters 1871	Conte et al. 2010	AF, PM	FZ, JV
Scinax haddadorum	Hylidae	NE	Araujo-Vieira et al. 2016	NA	CE	–
Scinax hayii	Hylidae	LC	Barbour 1909	Heyer et al. 1990	AF	FZ, ML, JV
Scinax hayii	Hylidae	LC	Barbour 1909	Pombal et al. 1995b	AF	FZ, ML, JV
Scinax hayii	Hylidae	LC	Barbour 1909	Magrini et al. 2011	AF	FZ, ML, JV
Scinax iquitorum	Hylidae	LC	Moravec et al. 2009b	NA	AM	–
Scinax imbegue	Hylidae	NE	Nunes et al. 2012	Pombal et al. 1995b	AF, PM	JV
Scinax imbegue	Hylidae	NE	Nunes et al. 2012	Nunes et al. 2012	AF, PM	JV
Scinax juncae	Hylidae	NE	Nunes & Pombal 2010	Nunes & Pombal 2010	AF	–
Scinax lindsayi	Hylidae	LC	Pyburn 1992	Pyburn 1992	AM	–
Scinax madeirae	Hylidae	NE	Bokermann 1964b	Brusquetti et al. 2014	AM	–

Taxa	Family	IUCN	Description Reference	Call_reference	Biome	Sound Library
Scinax maracaya	Hylidae	DD	Cardoso & Sazima 1980	Cardoso & Sazima 1980	CE	JV
Scinax maracaya	Hylidae	DD	Cardoso & Sazima 1980	Haddad et al. 1988	CE	JV
Scinax maracaya	Hylidae	DD	Cardoso & Sazima 1980	Bang & Giaretta 2016	CE	JV
Scinax melanodactylus	Hylidae	NE	Lourenço et al. 2014	NA	AF	–
Scinax montivagus	Hylidae	NE	Juncá et al. 2015	Juncá et al. 2015	CE	–
Scinax nasicus	Hylidae	LC	Cope 1862b	De La Riva et al. 1994	AF, CE, PM	FZ, ML, JV
Scinax nebulosus	Hylidae	LC	Spix 1824	Hödl 1977	AM, AF, CA	FZ, ML, JV
Scinax nebulosus	Hylidae	LC	Spix 1824	Duellman & Pyles 1983	AM, AF, CA	FZ, ML, JV
Scinax nebulosus	Hylidae	LC	Spix 1824	De La Riva et al. 1994	AM, AF, CA	FZ, ML, JV
Scinax nebulosus	Hylidae	LC	Spix 1824	Lescure & Marty 2000	AM, AF, CA	FZ, ML, JV
Scinax pachycrus	Hylidae	LC	Miranda-Ribeiro 1937c	Carneiro et al. 2004	AF, CA	JV
Scinax pedromedinae	Hylidae	LC	Henle 1991	Duellman & Wiens 1993	AM	FZ, ML
Scinax pedromedinae	Hylidae	LC	Henle 1991	Duellman 2005	AM	FZ, ML
Scinax perereca	Hylidae	LC	Pombal et al. 1995a	Pombal et al. 1995b	AF, PM	FZ, JV
Scinax perereca	Hylidae	LC	Pombal et al. 1995a	Kwet 2001	AF, PM	FZ, JV
Scinax perereca	Hylidae	LC	Pombal et al. 1995a	Bevier et al. 2008	AF, PM	FZ, JV
Scinax perereca	Hylidae	LC	Pombal et al. 1995a	Magrini & Giaretta 2010	AF, PM	FZ, JV
Scinax proboscideus	Hylidae	LC	Brongersma 1933	Lescure & Marty 2000	AM	FZ
Scinax rogerioi	Hylidae	NE	Pugliese et al. 2009	Pugliese et al. 2009	CE	–
Scinax rossaferesae	Hylidae	NE	Conte et al. 2016	Conte et al. 2016	AF	–
Scinax rostratus	Hylidae	LC	Peters 1863	Duellman 1972b	AM	FZ, ML, JV
Scinax ruber	Hylidae	LC	Laurenti 1768	Duellman 1978a	AM, CA	FZ, ML, JV
Scinax ruber	Hylidae	LC	Laurenti 1768	Schlüter 1979	AM, CA	FZ, ML, JV
Scinax ruber	Hylidae	LC	Laurenti 1768	Duellman & Pyles 1983	AM, CA	FZ, ML, JV
Scinax ruber	Hylidae	LC	Laurenti 1768	Zimmerman 1983	AM, CA	FZ, ML, JV
Scinax ruber	Hylidae	LC	Laurenti 1768	Duellman & Wiens 1993	AM, CA	FZ, ML, JV
Scinax ruber	Hylidae	LC	Laurenti 1768	De La Riva et al. 1994	AM, CA	FZ, ML, JV

Taxa	Family	IUCN	Description Reference	Call_reference	Biome	Sound Library
Scinax ruber	Hylidae	LC	Laurenti 1768	Lescure & Marty 2000	AM, CA	FZ, ML, JV
Scinax ruber	Hylidae	LC	Laurenti 1768	Bernal et al. 2004	AM, CA	FZ, ML, JV
Scinax ruber	Hylidae	LC	Laurenti 1768	Duellman 2005	AM, CA	FZ, ML, JV
Scinax rupestris	Hylidae	NE	Araujo-Vieira et al. 2015a	Araujo-Vieira et al. 2015a	CE	–
Scinax satermawe	Hylidae	NE	Sturaro & Peloso 2014	NA	AM	–
Scinax similis	Hylidae	LC	Cochran 1952	Silva et al. 2008	AF, CE	FZ, JV
Scinax similis	Hylidae	LC	Cochran 1952	Bilates & Lack 2011	AF, CE	FZ, JV
Scinax squalirostris	Hylidae	LC	Lutz 1925b	Haddad et al. 1988	AF, CE, PM, PN	FZ, ML, JV
Scinax squalirostris	Hylidae	LC	Lutz 1925b	Pombal et al. 1995b	AF, CE, PM, PN	FZ, ML, JV
Scinax squalirostris	Hylidae	LC	Lutz 1925b	Kwet 2001	AF, CE, PM, PN	FZ, ML, JV
Scinax squalirostris	Hylidae	LC	Lutz 1925b	Faria et al. 2013	AF, CE, PM, PN	FZ, ML, JV
Scinax tigrinus	Hylidae	LC	Nunes et al. 2010b	Nunes et al. 2010b	CE	–
Scinax tigrinus	Hylidae	LC	Nunes et al. 2010b	Bang & Giaretta 2016	CE	–
Scinax tymbamirim	Hylidae	NE	Nunes et al. 2012	Bokermann 1967g	AF, PM	JV
Scinax tymbamirim	Hylidae	NE	Nunes et al. 2012	Kwet 2001	AF, PM	JV
Scinax trapicheiroi	Hylidae	NT	Lutz 1954	NA	AF	–
Scinax villasboasi	Hylidae	NE	Brusquetti et al. 2014	NA	AM	–
Scinax x-signatus	Hylidae	LC	Spix 1824	Tárano 2010	AF, CA, CE	JV
Sphaenorhynchus botocudo	Hylidae	NE	Caramaschi et al. 2009a	NA	AF	–
Sphaenorhynchus bromelicola	Hylidae	DD	Bokermann 1966c	NA	AF, CA	–
Sphaenorhynchus canga	Hylidae	NE	Araujo-Vieira et al. 2015b	Araujo-Vieira et al. 2015b	CE	–
Sphaenorhynchus caramaschii	Hylidae	LC	Toledo et al. 2007b	Toledo et al. 2007b	AF, CE, PM	JV
Sphaenorhynchus caramaschii	Hylidae	LC	Toledo et al. 2007b	Pombal 2010	AF, CE, PM	JV
Sphaenorhynchus carneus	Hylidae	LC	Cope 1868	Hödl 1977	AM	FZ, ML, JV
Sphaenorhynchus carneus	Hylidae	LC	Cope 1868	Duellman 1978a	AM	FZ, ML, JV
Sphaenorhynchus carneus	Hylidae	LC	Cope 1868	Duellman & Pyles 1983	AM	FZ, ML, JV
Sphaenorhynchus dorisae	Hylidae	LC	Goin 1957	Hödl 1977	AM	FZ, JV

Taxa	Family	IUCN	Description Reference	Call_reference	Biome	Sound Library
Sphaenorhynchus lacteus	Hylidae	LC	Daudin 1800	Duellman 1978a	AM	FZ, ML, JV
Sphaenorhynchus lacteus	Hylidae	LC	Daudin 1800	De La Riva et al. 1995	AM	FZ, ML, JV
Sphaenorhynchus lacteus	Hylidae	LC	Daudin 1800	Lescure & Marty 2000	AM	FZ, ML, JV
Sphaenorhynchus lacteus	Hylidae	LC	Daudin 1800	Duellman 2005	AM	FZ, ML, JV
Sphaenorhynchus mirim	Hylidae	DD	Caramaschi et al. 2009a	Lacerda et al. 2011	AF	–
Sphaenorhynchus orophilus	Hylidae	LC	Lutz & Lutz 1938	Heyer et al. 1990	AF	FZ, ML, JV
Sphaenorhynchus palustris	Hylidae	LC	Bokermann 1966c	Lacerda et al. 2013	AF	JV
Sphaenorhynchus pauloalvini	Hylidae	DD	Bokermann 1973	Bokermann 1973	AF	JV
Sphaenorhynchus planicola	Hylidae	LC	Lutz & Lutz 1938	NA	AF	JV
Sphaenorhynchus prasinus	Hylidae	LC	Bokermann 1973	Bokermann 1973	AF, CA	JV
Sphaenorhynchus surdus	Hylidae	LC	Cochran 1953	Toledo et al. 2007b	AF, PM	FZ, JV
Tepuihyla exophthalma	Hylidae	DD	Smith & Noonan 2001	NA	AM	–
Tepuihyla shushupe	Hylidae	NE	Ron et al. 2016	Ron et al. 2016	AM	–
Tepuihyla tuberculosa	Hylidae	LC	Boulenger 1882	Ron et al. 2016	AM	–
Trachycephalus atlas	Hylidae	LC	Bokermann 1966h	Santos-Silva et al. 2012	AF, CA, CE	–
Trachycephalus cunauaru	Hylidae	NE	Gordo et al. 2013	Gordo et al. 2013	AM	–
Trachycephalus coriaceus	Hylidae	LC	Peters 1867	Schlüter 1979	AM	FZ, ML
Trachycephalus coriaceus	Hylidae	LC	Peters 1867	De La Riva et al. 1995	AM	FZ, ML
Trachycephalus coriaceus	Hylidae	LC	Peters 1867	Lescure & Marty 2000	AM	FZ, ML
Trachycephalus dibernardoi	Hylidae	LC	Kwet & Solé 2008	Kwet & Solé 2008	AF, PM	FZ, JV
Trachycephalus dibernardoi	Hylidae	LC	Kwet & Solé 2008	Conte et al. 2010	AF, PM	FZ, JV
Trachycephalus hadroceps	Hylidae	LC	Duellman & Hoogmoed 1992	Lescure & Marty 2000	AM	–
Trachycephalus helioi	Hylidae	NE	Nunes et al. 2013	NA	AM	–
Trachycephalus imitatrix	Hylidae	LC	Miranda-Ribeiro 1926	NA	AF	–
Trachycephalus lepidus	Hylidae	DD	Pombal et al. 2003a	NA	AF	–
Trachycephalus mambaiensis	Hylidae	NE	Cintra et al. 2009	NA	CE	–
Trachycephalus mesophaeus	Hylidae	LC	Hensel 1867	Prado et al. 2003	AF, CA, PM	FZ

Taxa	Family	IUCN	Description Reference	Call_reference	Biome	Sound Library
Trachycephalus nigromaculatus	Hylidae	LC	Tschudi 1838	Abrunhosa et al. 2001	AF, CE	JV
Trachycephalus resinifictrix	Hylidae	LC	Goeldi 1907	Zimmerman 1983	AM	FZ, ML
Trachycephalus resinifictrix	Hylidae	LC	Goeldi 1907	Zimmerman & Höld 1983	AM	FZ, ML
Trachycephalus resinifictrix	Hylidae	LC	Goeldi 1907	Zimmerman & Bogart 1984	AM	FZ, ML
Trachycephalus resinifictrix	Hylidae	LC	Goeldi 1907	Lescure & Marty 2000	AM	FZ, ML
Trachycephalus typhonius	Hylidae	LC	Linnaeus 1758	Duellman 1978a	AM, AF, CA, CE, PM, PN	FZ, ML, JV
Trachycephalus typhonius	Hylidae	LC	Linnaeus 1758	Zimmerman 1983	AM, AF, CA, CE, PM, PN	FZ, ML, JV
Trachycephalus typhonius	Hylidae	LC	Linnaeus 1758	De La Riva et al. 1995	AM, AF, CA, CE, PM, PN	FZ, ML, JV
Trachycephalus typhonius	Hylidae	LC	Linnaeus 1758	Lescure & Marty 2000	AM, AF, CA, CE, PM, PN	FZ, ML, JV
Trachycephalus typhonius	Hylidae	LC	Linnaeus 1758	Guimarães et al. 2001	AM, AF, CA, CE, PM, PN	FZ, ML, JV
Trachycephalus typhonius	Hylidae	LC	Linnaeus 1758	Duellman 2005	AM, AF, CA, CE, PM, PN	FZ, ML, JV
Xenohyla eugenioi	Hylidae	DD	Caramaschi 1998	NA	AF, CA	–
Xenohyla truncata	Hylidae	NT	Izecksohn 1959	NA	AF	–
Callimedusa atelopoides	Phyllomedusidae	LC	Duellman et al. 1988	Duellman 2005	AM	FZ, ML
Callimedusa atelopoides	Phyllomedusidae	LC	Duellman et al. 1988	Duellman 2005	AM	FZ, ML
Callimedusa tomopterna	Phyllomedusidae	LC	Cope 1868	Zimmerman 1983	AM	FZ, ML
Callimedusa tomopterna	Phyllomedusidae	LC	Cope 1868	Zimmerman & Bogart 1984	AM	FZ, ML
Callimedusa tomopterna	Phyllomedusidae	LC	Cope 1868	Lescure & Marty 2000	AM	FZ, ML
Callimedusa tomopterna	Phyllomedusidae	LC	Cope 1868	Duellman 2005	AM	FZ, ML
Cruziohyla craspedopus	Phyllomedusidae	LC	Funkhouser 1957	Hoogmoed & Cadle 1991	AM	–
Cruziohyla craspedopus	Phyllomedusidae	LC	Funkhouser 1957	Duellman 2005	AM	–
Hylomantis aspera	Phyllomedusidae	LC	Peters 1873	Pimenta et al. 2007	AF	–
Hylomantis granulosa	Phyllomedusidae	LC	Cruz 1988	Vilela et al. 2014	AF, CA	–
Phasmahyla cochranae	Phyllomedusidae	LC	Bokermann 1966a	NA	AF	JV
Phasmahyla cruzi	Phyllomedusidae	NE	Carvalho-e-Silva et al. 2009	NA	AF	–
Phasmahyla exilis	Phyllomedusidae	LC	Cruz 1980	NA	AF	–
Phasmahyla guttata	Phyllomedusidae	LC	Lutz 1924	NA	AF	–

Taxa	Family	IUCN	Description Reference	Call_reference	Biome	Sound Library
Phasmahyla jandaia	Phyllomedusidae	LC	Bokermann & Sazima 1978	NA	AF, CE	–
Phasmahyla spectabilis	Phyllomedusidae	DD	Cruz et al. 2008b	Dias et al. 2011	AF	–
Phasmahyla timbo	Phyllomedusidae	DD	Cruz et al. 2008c	Cruz et al. 2008c	AF, CA	–
Phrynomedusa appendiculata	Phyllomedusidae	NT	Lutz 1925a	NA	AF	JV
Phrynomedusa bokermanni	Phyllomedusidae	DD	Cruz 1991	NA	AF	–
Phrynomedusa dryade	Phyllomedusidae	NE	Baêta et al. 2016	Baêta et al. 2016	AF	–
Phrynomedusa fimbriata	Phyllomedusidae	Extinct	Miranda-Ribeiro 1923a	NA	AF	–
Phrynomedusa marginata	Phyllomedusidae	LC	Izecksohn & Cruz 1976	NA	AF	ML
Phrynomedusa vanzolinii	Phyllomedusidae	DD	Cruz 1991	NA	AF	–
Pithecopus ayeaye	Phyllomedusidae	CR	Lutz 1966	Nali et al. 2015	AF, CE	JV
Pithecopus azurea	Phyllomedusidae	DD	Cope 1862b	Barrio 1976	CE, PN	FZ, JV
Pithecopus azurea	Phyllomedusidae	DD	Cope 1862b	Guimarães et al. 2001	CE, PN	FZ, JV
Pithecopus centralis	Phyllomedusidae	DD	Bokermann 1965d	Brandão et al. 2009	CE	JV
Pithecopus hypochondrialis	Phyllomedusidae	DD	Daudin 1800	Duellman & Pyles 1983	AM, CA, CE	FZ, ML, JV
Pithecopus hypochondrialis	Phyllomedusidae	DD	Daudin 1800	De La Riva et al. 1995	AM, CA, CE	FZ, ML, JV
Pithecopus hypochondrialis	Phyllomedusidae	DD	Daudin 1800	Duellman 1997	AM, CA, CE	FZ, ML, JV
Pithecopus hypochondrialis	Phyllomedusidae	DD	Daudin 1800	Lescure & Marty 2000	AM, CA, CE	FZ, ML, JV
Pithecopus megacephala	Phyllomedusidae	DD	Miranda-Ribeiro 1926	Giaretta et al. 2007b	CE	JV
Pithecopus nordestina	Phyllomedusidae	DD	Caramaschi 2006	Vilaça et al. 2011	AF, CA, CE	JV
Pithecopus oreades	Phyllomedusidae	DD	Brandão 2002	Giaretta et al. 2007b	CE	–
Pithecopus oreades	Phyllomedusidae	DD	Brandão 2002	Brandão & Alvares 2009	CE	–
Pithecopus palliata	Phyllomedusidae	LC	Peters 1873	Duellman 1978a	AM	FZ, ML, JV
Pithecopus palliata	Phyllomedusidae	LC	Peters 1873	Duellman & Pyles 1983	AM	FZ, ML, JV
Pithecopus palliata	Phyllomedusidae	LC	Peters 1873	Köhler & Lötters 1999a	AM	FZ, ML, JV
Pithecopus palliata	Phyllomedusidae	LC	Peters 1873	Duellman 2005	AM	FZ, ML, JV
Pithecopus rohdei	Phyllomedusidae	LC	Mertens 1926	Wogel et al. 2004a	AF	JV
Pithecopus rustica	Phyllomedusidae	NE	Bruschi et al. 2014	NA	PM	JV

Taxa	Family	IUCN	Description Reference	Call_reference	Biome	Sound Library
Phyllomedusa bahiana	Phyllomedusidae	DD	Lutz 1925a	Silva-Filho & Juncá 2006	AF, CA, CE	JV
Phyllomedusa bicolor	Phyllomedusidae	LC	Boddaert 1772	Zimmerman 1983	AM	FZ, ML, JV
Phyllomedusa bicolor	Phyllomedusidae	LC	Boddaert 1772	Zimmerman & Bogart 1984	AM	FZ, ML, JV
Phyllomedusa bicolor	Phyllomedusidae	LC	Boddaert 1772	Lescure & Marty 2000	AM	FZ, ML, JV
Phyllomedusa boliviana	Phyllomedusidae	LC	Boulenger 1902	Barrio 1976	AM, PN	FZ, JV
Phyllomedusa boliviana	Phyllomedusidae	LC	Boulenger 1902	Köhler 2000	AM, PN	FZ, JV
Phyllomedusa burmeisteri	Phyllomedusidae	DD	Boulenger 1882	Barrio 1976	AF, CE	ML, JV
Phyllomedusa burmeisteri	Phyllomedusidae	DD	Boulenger 1882	Abrunhosa & Wogel 2004	AF, CE	ML, JV
Phyllomedusa camba	Phyllomedusidae	LC	De la Riva 1999	De La Riva et al. 1995	AM	FZ, ML
Phyllomedusa camba	Phyllomedusidae	LC	De la Riva 1999	Duellman 2005	AM	FZ, ML
Phyllomedusa distincta	Phyllomedusidae	LC	Lutz 1950	Haddad et al. 1994	AF, CE, PM	FZ, ML, JV
Phyllomedusa distincta	Phyllomedusidae	LC	Lutz 1950	Pombal 2010	AF, CE, PM	FZ, ML, JV
Phyllomedusa iheringii	Phyllomedusidae	LC	Boulenger 1885	Kwet 1999	AF, PM	FZ, JV
Phyllomedusa sauvagii	Phyllomedusidae	LC	Boulenger 1882	Barrio 1976	CE, PN	FZ, JV
Phyllomedusa tarsius	Phyllomedusidae	LC	Cope 1868	Duellman 1978a	AM	FZ, ML, JV
Phyllomedusa tarsius	Phyllomedusidae	LC	Cope 1868	Schlüter 1979	AM	FZ, ML, JV
Phyllomedusa tarsius	Phyllomedusidae	LC	Cope 1868	Duellman & Pyles 1983	AM	FZ, ML, JV
Phyllomedusa tarsius	Phyllomedusidae	LC	Cope 1868	Zimmerman 1983	AM	FZ, ML, JV
Phyllomedusa tarsius	Phyllomedusidae	LC	Cope 1868	Zimmerman & Bogart 1984	AM	FZ, ML, JV
Phyllomedusa tetraploidea	Phyllomedusidae	LC	Pombal & Haddad 1992	Pombal & Haddad 1992	AF, PM	FZ, JV
Phyllomedusa tetraploidea	Phyllomedusidae	LC	Pombal & Haddad 1992	Haddad et al. 1994	AF, PM	FZ, JV
Phyllomedusa tetraploidea	Phyllomedusidae	LC	Pombal & Haddad 1992	Pombal 2010	AF, PM	FZ, JV
Phyllomedusa vaillantii	Phyllomedusidae	LC	Boulenger 1882	Duellman 1978a	AM	FZ, ML, JV
Phyllomedusa vaillantii	Phyllomedusidae	LC	Boulenger 1882	Schlüter 1979	AM	FZ, ML, JV
Phyllomedusa vaillantii	Phyllomedusidae	LC	Boulenger 1882	Zimmerman 1983	AM	FZ, ML, JV
Phyllomedusa vaillantii	Phyllomedusidae	LC	Boulenger 1882	Lescure & Marty 2000	AM	FZ, ML, JV
Phyllomedusa vaillantii	Phyllomedusidae	LC	Boulenger 1882	Duellman 2005	AM	FZ, ML, JV

Taxa	Family	IUCN	Description Reference	Call_reference	Biome	Sound Library
Crossodactylus aeneus	Hylodidae	DD	Müller 1924b	NA	AF	JV
Crossodactylus boulengeri	Hylodidae	NE	De Witte 1930	NA	AF	JV
Crossodactylus caramaschii	Hylodidae	LC	Bastos & Pombal 1995	Bastos & Pombal 1995	AF	FZ, JV
Crossodactylus cyclospinus	Hylodidae	DD	Nascimento et al. 2005	Nascimento et al. 2005	AF, CA	–
Crossodactylus dantei	Hylodidae	DD	Carcerelli & Caramaschi 1992	NA	AF	–
Crossodactylus díspar	Hylodidae	DD	Lutz 1925a	NA	AF	–
Crossodactylus franciscanus	Hylodidae	NE	Pimenta et al. 2015	Pimenta et al. 2008	AF	JV
Crossodactylus franciscanus	Hylodidae	NE	Pimenta et al. 2015	Pimenta et al. 2015	AF	JV
Crossodactylus gaudichaudii	Hylodidae	LC	Duméril & Bibron 1841	Weygoldt & Carvalho-e-Silva 1992	AF	JV
Crossodactylus grandis	Hylodidae	DD	Lutz 1951	NA	AF	–
Crossodactylus lutzorum	Hylodidae	DD	Carcerelli & Caramaschi 1992	NA	AF	–
Crossodactylus schmidti	Hylodidae	NT	Gallardo 1961a	Caldart et al. 2011	AF	–
Crossodactylus schmidti	Hylodidae	NT	Gallardo 1961a	Caldart et al. 2016	AF	–
Crossodactylus timbuhy	Hylodidae	NE	Pimenta et al. 2014	Weygoldt 1986	AF	–
Crossodactylus trachystomus	Hylodidae	DD	Reinhardt & Lütken 1862	Pimenta et al. 2008	AF, CE	JV
Crossodactylus werneri	Hylodidae	NE	Pimenta et al. 2014	NA	AF	–
Hylodes amnicola	Hylodidae	DD	Pombal et al. 2002	Pombal et al. 2002	AF	–
Hylodes asper	Hylodidae	LC	Müller 1924b	Haddad et al. 1999	AF	FZ, ML, JV
Hylodes babax	Hylodidae	DD	Heyer 1982	Heyer 1982	AF	ML, JV
Hylodes babax	Hylodidae	DD	Heyer 1982	Pirani et al. 2010	AF	ML, JV
Hylodes cardosoi	Hylodidae	LC	Lingnau et al. 2008a	Lingnau et al. 2008a	AF	FZ, JV
Hylodes cardosoi	Hylodidae	LC	Lingnau et al. 2008a	Forti & Castanho 2012	AF	FZ, JV
Hylodes charadranaetes	Hylodidae	DD	Heyer & Cocroft 1986	Heyer & Cocroft 1986	AF	ML, JV
Hylodes charadranaetes	Hylodidae	DD	Heyer & Cocroft 1986	Weber et al. 2007	AF	ML, JV
Hylodes dactylocinus	Hylodidae	DD	Pavan et al. 2001	Pavan et al. 2001	AF	–
Hylodes fredi	Hylodidae	DD	Canedo & Pombal 2007	Canedo & Pombal 2007	AF	ML
Hylodes glaber	Hylodidae	DD	Miranda-Ribeiro 1926	NA	AF	JV

Taxa	Family	IUCN	Description Reference	Call_reference	Biome	Sound Library
Hylodes heyeri	Hylodidae	DD	Haddad et al. 1996	Haddad et al. 1996	AF	FZ, JV
Hylodes heyeri	Hylodidae	DD	Haddad et al. 1996	Lingnau & Bastos 2007	AF	FZ, JV
Hylodes japi	Hylodidae	NE	Sá et al. 2015	Sá et al. 2015	AF	JV
Hylodes japi	Hylodidae	NE	Sá et al. 2015	Sá et al. 2016	AF	JV
Hylodes lateristrigatus	Hylodidae	LC	Baumann 1912	Bokermann 1964c	AF	ML, JV
Hylodes lateristrigatus	Hylodidae	LC	Baumann 1912	Heyer 1982	AF	ML, JV
Hylodes lateristrigatus	Hylodidae	LC	Baumann 1912	Heyer & Cocroft 1986	AF	ML, JV
Hylodes magalhaesi	Hylodidae	DD	Bokermann 1964c	Bokermann 1964c	AF	JV
Hylodes meridionalis	Hylodidae	LC	Mertens 1927	Lingnau et al. 2013	AF	FZ, JV
Hylodes mertensi	Hylodidae	DD	Bokermann 1956c	NA	AF	–
Hylodes nasus	Hylodidae	LC	Lichtenstein 1823	Wogel et al. 2004c	AF	JV
Hylodes ornatus	Hylodidae	LC	Bokermann 1967m	Bilate et al. 2012	AF	–
Hylodes otavioi	Hylodidae	DD	Sazima & Bokermann 1982	Sazima & Bokermann 1982	AF, CE	JV
Hylodes perere	Hylodidae	NE	Silva & Benmaman 2008	Silva & Benmaman 2008	AF	JV
Hylodes perplicatus	Hylodidae	LC	Miranda-Ribeiro 1926	Haddad et al. 2003	AF	FZ
Hylodes phyllodes	Hylodidae	LC	Heyer & Cocroft 1986	Heyer & Cocroft 1986	AF	FZ, ML, JV
Hylodes phyllodes	Hylodidae	LC	Heyer & Cocroft 1986	Heyer et al. 1990	AF	FZ, ML, JV
Hylodes phyllodes	Hylodidae	LC	Heyer & Cocroft 1986	Hartmann et al. 2006	AF	FZ, ML, JV
Hylodes pipilans	Hylodidae	DD	Canedo & Pombal 2007	Canedo & Pombal 2007	AF	JV
Hylodes regius	Hylodidae	DD	Gouvêa 1979	Heyer 1982	AF	ML
Hylodes sazimai	Hylodidae	DD	Haddad & Pombal 1995	Haddad & Pombal 1995	AF	FZ, JV
Hylodes uai	Hylodidae	DD	Nascimento et al. 2001a	Nascimento et al. 2001a	AF	–
Hylodes vanzolinii	Hylodidae	DD	Heyer 1982	NA	AF	–
Megaelosia apuana	Hylodidae	DD	Pombal et al. 2003b	NA	AF	–
Megaelosia bocainensis	Hylodidae	DD	Giaretta et al. 1993	NA	AF	–
Megaelosia boticariana	Hylodidae	DD	Giaretta & Aguiar 1998	NA	AF	–
Megaelosia goeldii	Hylodidae	LC	Baumann 1912	NA	AF	–

Taxa	Family	IUCN	Description Reference	Call_reference	Biome	Sound Library
Megaelosia jordanensis	Hylodidae	DD	Heyer 1983c	NA	AF	–
Megaelosia lutzae	Hylodidae	DD	Izecksohn & Gouvêa 1985	NA	AF	–
Megaelosia massarti	Hylodidae	DD	De Witte 1930	NA	AF	–
Edalorhina perezi	Leptodactylidae	LC	Jiménez de la Espada 1870	Schlüter 1980b	AM	FZ, ML
Edalorhina perezi	Leptodactylidae	LC	Jiménez de la Espada 1870	Duellman & Morales 1990	AM	FZ, ML
Edalorhina perezi	Leptodactylidae	LC	Jiménez de la Espada 1870	Schlüter 1990	AM	FZ, ML
Edalorhina perezi	Leptodactylidae	LC	Jiménez de la Espada 1870	Duellman 2005	AM	FZ, ML
Engystomops petersi	Leptodactylidae	LC	Jiménez de la Espada 1872	Schlüter 1980b	AM	FZ, ML, JV
Engystomops petersi	Leptodactylidae	LC	Jiménez de la Espada 1872	Márquez et al. 1995	AM	FZ, ML, JV
Engystomops petersi	Leptodactylidae	LC	Jiménez de la Espada 1872	Lescure & Marty 2000	AM	FZ, ML, JV
Engystomops petersi	Leptodactylidae	LC	Jiménez de la Espada 1872	Duellman 2005	AM	FZ, ML, JV
Engystomops petersi	Leptodactylidae	LC	Jiménez de la Espada 1872	Funk et al. 2008	AM	FZ, ML, JV
Engystomops petersi	Leptodactylidae	LC	Jiménez de la Espada 1872	Guerra & Ron 2008	AM	FZ, ML, JV
Engystomops freibergi	Leptodactylidae	LC	Donoso-Barros 1969	Funk et al. 2008	AM	–
Physalaemus aguirrei	Leptodactylidae	LC	Bokermann 1966b	Bokermann 1966b	AF	JV
Physalaemus aguirrei	Leptodactylidae	LC	Bokermann 1966b	Pimenta & Cruz 2004	AF	JV
Physalaemus albifrons	Leptodactylidae	LC	Spix 1824	Pederassi et al. 2015	CA, CE	JV
Physalaemus albonotatus	Leptodactylidae	LC	Steindachner 1864	Barrio 1965b	CE, PN	FZ, ML, JV
Physalaemus albonotatus	Leptodactylidae	LC	Steindachner 1864	Márquez et al. 1995	CE, PN	FZ, ML, JV
Physalaemus albonotatus	Leptodactylidae	LC	Steindachner 1864	Köhler 2000	CE, PN	FZ, ML, JV
Physalaemus angrensis	Leptodactylidae	DD	Weber et al. 2005b	Weber et al. 2005b	AF	–
Physalaemus atim	Leptodactylidae	NE	Brasileiro & Haddad 2015	Brasileiro & Haddad 2015	AF	–
Physalaemus atlanticus	Leptodactylidae	VU	Haddad & Sazima 2004	Haddad & Sazima 2004	AF	JV
Physalaemus atlanticus	Leptodactylidae	VU	Haddad & Sazima 2004	Weber et al. 2005b	AF	JV
Physalaemus barrioi	Leptodactylidae	DD	Bokermann 1967k	Bokermann 1967k	AF	JV
Physalaemus barrioi	Leptodactylidae	DD	Bokermann 1967k	Provete et al. 2012	AF	JV
Physalaemus biligonigerus	Leptodactylidae	LC	Cope 1861	Barrio 1965a	AF, PM	FZ, ML, JV

Taxa	Family	IUCN	Description Reference	Call_reference	Biome	Sound Library
Physalaemus biligonigerus	Leptodactylidae	LC	Cope 1861	Márquez et al. 1995	AF, PM	FZ, ML, JV
Physalaemus biligonigerus	Leptodactylidae	LC	Cope 1861	Salas et al. 1998	AF, PM	FZ, ML, JV
Physalaemus bokermanni	Leptodactylidae	DD	Cardoso & Haddad 1985	Cardoso & Haddad 1985	AF	JV
Physalaemus bokermanni	Leptodactylidae	DD	Cardoso & Haddad 1985	Weber et al. 2005b	AF	JV
Physalaemus bokermanni	Leptodactylidae	DD	Cardoso & Haddad 1985	Giaretta et al. 2009	AF	JV
Physalaemus caete	Leptodactylidae	DD	Pombal & Madureira 1997	NA	AF	–
Physalaemus camacan	Leptodactylidae	DD	Pimenta et al. 2005	Pimenta et al. 2005	AF	–
Physalaemus centralis	Leptodactylidae	LC	Bokermann 1962d	Padial & Köhler 2001	CA, CE, PN	FZ, ML, JV
Physalaemus centralis	Leptodactylidae	LC	Bokermann 1962d	Silva et al. 2008	CA, CE, PN	FZ, ML, JV
Physalaemus centralis	Leptodactylidae	LC	Bokermann 1962d	Vasconcelos et al. 2014	CA, CE, PN	FZ, ML, JV
Physalaemus cicada	Leptodactylidae	LC	Bokermann 1966e	NA	CA, CE	JV
Physalaemus crombiei	Leptodactylidae	LC	Heyer & Wolf 1989	Heyer & Wolf 1989	AF	FZ, JV
Physalaemus crombiei	Leptodactylidae	LC	Heyer & Wolf 1989	Weber et al. 2005b	AF	FZ, JV
Physalaemus cuvieri	Leptodactylidae	LC	Fitzinger 1826	Barrio 1965b	AF, CA, CE, PM, PN	FZ, ML, JV
Physalaemus cuvieri	Leptodactylidae	LC	Fitzinger 1826	Heyer et al. 1990	AF, CA, CE, PM, PN	FZ, ML, JV
Physalaemus cuvieri	Leptodactylidae	LC	Fitzinger 1826	Silva et al. 2008	AF, CA, CE, PM, PN	FZ, ML, JV
Physalaemus cuvieri	Leptodactylidae	LC	Fitzinger 1826	Pombal 2010	AF, CA, CE, PM, PN	FZ, ML, JV
Physalaemus cuvieri	Leptodactylidae	LC	Fitzinger 1826	Gambale & Bastos 2014	AF, CA, CE, PM, PN	FZ, ML, JV
Physalaemus deimaticus	Leptodactylidae	DD	Sazima & Caramaschi 1988	NA	CE	–
Physalaemus ephippifer	Leptodactylidae	LC	Steindachner 1864	Kaefer et al. 2011	AM	FZ, ML, JV
Physalaemus erikae	Leptodactylidae	LC	Cruz & Pimenta 2004	Cruz & Pimenta 2004	AF	JV
Physalaemus erythros	Leptodactylidae	DD	Caramaschi et al. 2003	Baêta et al. 2007b	AF	–
Physalaemus evangelistai	Leptodactylidae	DD	Bokermann 1967k	Bokermann 1967k	CE	ML, JV
Physalaemus feioi	Leptodactylidae	NE	Cassini et al. 2010	Cassini et al. 2010	AF	JV
Physalaemus gracilis	Leptodactylidae	LC	Boulenger 1883	Barrio 1965b	AF, CA, CE, PM	FZ, JV
Physalaemus henselii	Leptodactylidae	LC	Peters 1872a	Barrio 1965b	AF, PM	ML, JV
Physalaemus henselii	Leptodactylidae	LC	Peters 1872a	Maneyro et al. 2008	AF, PM	ML, JV

Taxa	Family	IUCN	Description Reference	Call_reference	Biome	Sound Library
Physalaemus insperatus	Leptodactylidae	DD	Cruz et al. 2008a	NA	AF	–
Physalaemus irroratus	Leptodactylidae	DD	Cruz et al. 2007d	Cruz et al. 2007d	AF	–
Physalaemus jordanensis	Leptodactylidae	DD	Bokermann 1967k	Bokermann 1967k	AF	JV
Physalaemus jordanensis	Leptodactylidae	DD	Bokermann 1967k	Giaretta et al. 2009	AF	JV
Physalaemus kroyeri	Leptodactylidae	LC	Reinhardt & Lütken 1862	Bokermann 1966e	AF, CA	JV
Physalaemus lateristriga	Leptodactylidae	NE	Steindachner 1864	Cassini et al. 2010	AF	JV
Physalaemus lisei	Leptodactylidae	LC	Braun & Braun 1977b	Morais & Kwet 2012	AF, PM	FZ, JV
Physalaemus maculiventris	Leptodactylidae	LC	Lutz 1925a	Heyer et al. 1990	AF	FZ, ML, JV
Physalaemus maculiventris	Leptodactylidae	LC	Lutz 1925a	Weber et al. 2005b	AF	FZ, ML, JV
Physalaemus marmoratus	Leptodactylidae	LC	Reinhardt & Lütken 1862	Giaretta & Menin 2004	AF, CE, PN	FZ, JV
Physalaemus maximus	Leptodactylidae	DD	Feio et al. 1999	Baêta et al. 2007a	AF	JV
Physalaemus maximus	Leptodactylidae	DD	Feio et al. 1999	Peres & Simon 2012	AF	JV
Physalaemus moreirae	Leptodactylidae	DD	Miranda-Ribeiro 1937a	Weber et al. 2005b	AF	ML, JV
Physalaemus moreirae	Leptodactylidae	DD	Miranda-Ribeiro 1937a	Giaretta et al. 2009	AF	ML, JV
Physalaemus nanus	Leptodactylidae	LC	Boulenger 1888b	Weber et al. 2005b	AF	FZ, JV
Physalaemus nattereri	Leptodactylidae	LC	Steindachner 1863	Bokermann 1966b	CE, PN	FZ, ML, JV
Physalaemus nattereri	Leptodactylidae	LC	Steindachner 1863	Márquez et al. 1995	CE, PN	FZ, ML, JV
Physalaemus nattereri	Leptodactylidae	LC	Steindachner 1863	Silva et al. 2008	CE, PN	FZ, ML, JV
Physalaemus obtectus	Leptodactylidae	DD	Bokermann 1966b	Bokermann 1966b	AF	JV
Physalaemus obtectus	Leptodactylidae	DD	Bokermann 1966b	Weber et al. 2005b	AF	JV
Physalaemus olfersii	Leptodactylidae	LC	Lichtenstein & Martens 1856	Bokermann 1966b	AF, CE	FZ, ML, JV
Physalaemus olfersii	Leptodactylidae	LC	Lichtenstein & Martens 1856	Heyer et al. 1990	AF, CE	FZ, ML, JV
Physalaemus olfersii	Leptodactylidae	LC	Lichtenstein & Martens 1856	Giaretta et al. 2009	AF, CE	FZ, ML, JV
Physalaemus olfersii	Leptodactylidae	LC	Lichtenstein & Martens 1856	Cassini et al. 2010	AF, CE	FZ, ML, JV
Physalaemus olfersii	Leptodactylidae	LC	Lichtenstein & Martens 1856	Pombal 2010	AF, CE	FZ, ML, JV
Physalaemus orophilus	Leptodactylidae	NE	Cassini et al. 2010	Cassini et al. 2010	AF, CA, CE	JV
Physalaemus riograndensis	Leptodactylidae	LC	Milstead 1960	Barrio 1965b	PM	FZ, ML, JV

Taxa	Family	IUCN	Description Reference	Call_reference	Biome	Sound Library
Physalaemus rupestris	Leptodactylidae	DD	Caramaschi et al. 1991	Nascimento et al. 2001b	AF	–
Physalaemus signifer	Leptodactylidae	LC	Girard 1853	Wogel et al. 2002	AF, CE	JV
Physalaemus signifer	Leptodactylidae	LC	Girard 1853	Weber et al. 2005b	AF, CE	JV
Physalaemus soaresi	Leptodactylidae	EN	Izecksohn 1965	Cassini et al. 2010	AF, CA	JV
Physalaemus spiniger	Leptodactylidae	LC	Miranda-Ribeiro 1926	Costa & Toledo 2013	AF	JV
Pleurodema alium	Leptodactylidae	NE	Maciel & Nunes 2010	NA	CE	–
Pleurodema bibroni	Leptodactylidae	NT	Tschudi 1838	Kolenc et al. 2009	PM	–
Pleurodema brachyops	Leptodactylidae	LC	Cope 1869	Tárano 2010	PM	FZ, ML, JV
Pleurodema diplolister	Leptodactylidae	LC	Peters 1870	Hödl 1992	CA, CE	JV
Pseudopaludicola ameghini	Leptodactylidae	NE	Cope 1887	Pansonato et al. 2013	CE, PN	JV
Pseudopaludicola atragula	Leptodactylidae	NE	Pansonato et al. 2014a	Pansonato et al. 2014a	CE	–
Pseudopaludicola boliviana	Leptodactylidae	LC	Parker 1927a	Márquez et al. 1995	CE	FZ, ML, JV
Pseudopaludicola boliviana	Leptodactylidae	LC	Parker 1927a	Duré et al. 2004	CE	FZ, ML, JV
Pseudopaludicola canga	Leptodactylidae	DD	Giaretta & Kokubum 2003	Giaretta & Kokubum 2003	CE	JV
Pseudopaludicola canga	Leptodactylidae	DD	Giaretta & Kokubum 2003	Pansonato et al. 2012	CE	JV
Pseudopaludicola canga	Leptodactylidae	DD	Giaretta & Kokubum 2003	Carvalho et al. 2015c	CE	JV
Pseudopaludicola ceratophyes	Leptodactylidae	LC	Rivero & Serna 1985	NA	AM	–
Pseudopaludicola facureae	Leptodactylidae	DD	Andrade & Carvalho 2013	Andrade & Carvalho 2013	CE	FZ, JV
Pseudopaludicola facureae	Leptodactylidae	DD	Andrade & Carvalho 2013	Carvalho et al. 2015c	CE	FZ, JV
Pseudopaludicola falcipes	Leptodactylidae	LC	Hensel 1867	Haddad & Cardoso 1987	AF, CE, PM	ML, JV
Pseudopaludicola giarettai	Leptodactylidae	NE	Carvalho 2012	Carvalho 2012	CE	JV
Pseudopaludicola hyleaustralis	Leptodactylidae	NE	Pansonato et al. 2012	Pansonato et al. 2012	CE	–
Pseudopaludicola hyleaustralis	Leptodactylidae	NE	Pansonato et al. 2012	Carvalho et al. 2015c	CE	–
Pseudopaludicola ibisoroca	Leptodactylidae	NE	Pansonato et al. 2016	Pansonato et al. 2016	CE	–
Pseudopaludicola jaredi	Leptodactylidae	NE	Andrade et al. 2016	Andrade et al. 2016	AF	JV
Pseudopaludicola mineira	Leptodactylidae	DD	Lobo 1994	Pereira & Nascimento 2004	CE	JV
Pseudopaludicola motorzinho	Leptodactylidae	NE	Pansonato et al. 2016	Pansonato et al. 2016	CE	–

Taxa	Family	IUCN	Description Reference	Call_reference	Biome	Sound Library
Pseudopaludicola murundu	Leptodactylidae	NE	Toledo et al. 2010a	Toledo et al. 2010a	CE	JV
Pseudopaludicola murundu	Leptodactylidae	NE	Toledo et al. 2010a	Pansonato et al. 2014b	CE	JV
Pseudopaludicola mystacalis	Leptodactylidae	LC	Cope 1887	Haddad & Cardoso 1987	CA, CE, PN	FZ, JV
Pseudopaludicola mystacalis	Leptodactylidae	LC	Cope 1887	Pansonato et al. 2013	CA, CE, PN	FZ, JV
Pseudopaludicola mystacalis	Leptodactylidae	LC	Cope 1887	Pansonato et al. 2014b	CA, CE, PN	FZ, JV
Pseudopaludicola parnaiba	Leptodactylidae	NE	Roberto et al. 2013	Roberto et al. 2013	CE	–
Pseudopaludicola parnaiba	Leptodactylidae	NE	Roberto et al. 2013	Carvalho et al. 2015c	CE	–
Pseudopaludicola pocoto	Leptodactylidae	NE	Magalhães et al. 2014	Magalhães et al. 2014	CA	JV
Pseudopaludicola saltica	Leptodactylidae	LC	Cope 1887	Haddad et al. 1988	CE, PN	JV
Pseudopaludicola saltica	Leptodactylidae	LC	Cope 1887	Pansonato et al. 2013	CE, PN	JV
Pseudopaludicola saltica	Leptodactylidae	LC	Cope 1887	Pansonato et al. 2014b	CE, PN	JV
Pseudopaludicola ternetzi	Leptodactylidae	NE	Miranda-Ribeiro 1937a	Cardozo & Toledo 2013	CE	JV
Adenomera ajurauna	Leptodactylidae	DD	Berneck et al. 2008	Berneck et al. 2008	AF	JV
Adenomera andreae	Leptodactylidae	LC	Müller 1923	Zimmerman 1983	AM	FZ, ML, JV
Adenomera andreae	Leptodactylidae	LC	Müller 1923	Zimmerman & Bogart 1984	AM	FZ, ML, JV
Adenomera andreae	Leptodactylidae	LC	Müller 1923	Márquez et al. 1995	AM	FZ, ML, JV
Adenomera andreae	Leptodactylidae	LC	Müller 1923	Lescure & Marty 2000	AM	FZ, ML, JV
Adenomera andreae	Leptodactylidae	LC	Müller 1923	Ângulo & Icochea 2003	AM	FZ, ML, JV
Adenomera araucaria	Leptodactylidae	LC	Kwet & Ângulo 2002	Kwet & Ângulo 2002	AF	FZ, JV
Adenomera araucaria	Leptodactylidae	LC	Kwet & Ângulo 2002	kwet 2007	AF	FZ, JV
Adenomera araucaria	Leptodactylidae	LC	Kwet & Ângulo 2002	Conte et al. 2010	AF	FZ, JV
Adenomera bokermanni	Leptodactylidae	LC	Heyer 1973	Kwet et al. 2009	AF, CE	FZ, JV
Adenomera cotuba	Leptodactylidae	NE	Carvalho & Giaretta 2013b	Carvalho & Giaretta 2013b	CE	–
Adenomera diptyx	Leptodactylidae	LC	Boettger 1885	Márquez et al. 1995	CE, PN	FZ, ML
Adenomera engelsi	Leptodactylidae	NE	Kwet et al. 2009	Kwet et al. 2009	AF	FZ, JV
Adenomera heyeri	Leptodactylidae	LC	Boistel et al. 2006	Boistel et al. 2006	AM	–
Adenomera hylaedactyla	Leptodactylidae	LC	Cope 1868	Straughan & Heyer 1976	AM, CE	FZ, ML, JV

Taxa	Family	IUCN	Description Reference	Call_reference	Biome	Sound Library
Adenomera hylaedactyla	Leptodactylidae	LC	Cope 1868	Schlüter 1980b	AM, CE	FZ, ML, JV
Adenomera hylaedactyla	Leptodactylidae	LC	Cope 1868	Zimmerman 1983	AM, CE	FZ, ML, JV
Adenomera hylaedactyla	Leptodactylidae	LC	Cope 1868	Schneider et al. 1988	AM, CE	FZ, ML, JV
Adenomera hylaedactyla	Leptodactylidae	LC	Cope 1868	Lescure & Marty 2000	AM, CE	FZ, ML, JV
Adenomera hylaedactyla	Leptodactylidae	LC	Cope 1868	Ângulo et al. 2003	AM, CE	FZ, ML, JV
Adenomera juikitam	Leptodactylidae	NE	Carvalho & Giaretta 2013b	Carvalho & Giaretta 2013b	CE	–
Adenomera marmorata	Leptodactylidae	LC	Steindachner 1867	Barrio 1965a	AF	FZ, ML, JV
Adenomera marmorata	Leptodactylidae	LC	Steindachner 1867	Heyer et al. 1990	AF	FZ, ML, JV
Adenomera marmorata	Leptodactylidae	LC	Steindachner 1867	Pombal 2010	AF	FZ, ML, JV
Adenomera martinezi	Leptodactylidae	LC	Bokermann 1956b	Carvalho & Giaretta 2013c	CE	JV
Adenomera nana	Leptodactylidae	LC	Müller 1922	Kwet et al. 2009	AF	FZ
Adenomera saci	Leptodactylidae	NE	Carvalho & Giaretta 2013c	Carvalho & Giaretta 2013c	CE	–
Adenomera thomei	Leptodactylidae	LC	Almeida & Ângulo 2006	Almeida & Ângulo 2006	AF	JV
Adenomera thomei	Leptodactylidae	LC	Almeida & Ângulo 2006	Ferrante et al. 2014	AF	JV
Hydrolaetare caparu	Leptodactylidae	DD	Jansen et al. 2007	Jansen et al. 2007	AM	–
Hydrolaetare dantasi	Leptodactylidae	LC	Bokermann 1959	Souza & Haddad 2003	AM	–
Hydrolaetare schmidti	Leptodactylidae	LC	Cochran & Goin 1959	Lescure & Marty 2000	AM	FZ
Leptodactylus bolivianus	Leptodactylidae	LC	Boulenger 1898	Fouquette 1960	AM	FZ, ML, JV
Leptodactylus bolivianus	Leptodactylidae	LC	Boulenger 1898	Straughan & Heyer 1976	AM	FZ, ML, JV
Leptodactylus bolivianus	Leptodactylidae	LC	Boulenger 1898	Heyer et al. 1990	AM	FZ, ML, JV
Leptodactylus bolivianus	Leptodactylidae	LC	Boulenger 1898	Márquez et al. 1995	AM	FZ, ML, JV
Leptodactylus bolivianus	Leptodactylidae	LC	Boulenger 1898	Tárano 2010	AM	FZ, ML, JV
Leptodactylus bolivianus	Leptodactylidae	LC	Boulenger 1898	Sá et al. 2014	AM	FZ, ML, JV
Leptodactylus bufonius	Leptodactylidae	LC	Boulenger 1894	Barrio 1965a	PN	FZ, ML, JV
Leptodactylus bufonius	Leptodactylidae	LC	Boulenger 1894	Straughan & Heyer 1976	PN	FZ, ML, JV
Leptodactylus bufonius	Leptodactylidae	LC	Boulenger 1894	Sá et al. 2014	PN	FZ, ML, JV
Leptodactylus caatingae	Leptodactylidae	LC	Heyer & Juncá 2003	Heyer & Juncá 2003	CA	–

Taxa	Family	IUCN	Description Reference	Call_reference	Biome	Sound Library
Leptodactylus caatingae	Leptodactylidae	LC	Heyer & Juncá 2003	Sá et al. 2014	CA	–
Leptodactylus camaquara	Leptodactylidae	DD	Sazima & Bokermann 1978	Sazima & Bokermann 1978	CE	JV
Leptodactylus camaquara	Leptodactylidae	DD	Sazima & Bokermann 1978	Carvalho et al. 2013b	CE	JV
Leptodactylus camaquara	Leptodactylidae	DD	Sazima & Bokermann 1978	Sá et al. 2014	CE	JV
Leptodactylus chaquensis	Leptodactylidae	LC	Cei 1950	Barrio 1965a	CE, PM, PN	FZ, ML
Leptodactylus chaquensis	Leptodactylidae	LC	Cei 1950	Heyer & Giaretta 2009	CE, PM, PN	FZ, ML
Leptodactylus chaquensis	Leptodactylidae	LC	Cei 1950	Sá et al. 2014	CE, PM, PN	FZ, ML
Leptodactylus cunicularius	Leptodactylidae	LC	Sazima & Bokermann 1978	Sazima & Bokermann 1978	CE	FZ, ML, JV
Leptodactylus cunicularius	Leptodactylidae	LC	Sazima & Bokermann 1978	Heyer et al. 2008	CE	FZ, ML, JV
Leptodactylus cunicularius	Leptodactylidae	LC	Sazima & Bokermann 1978	Sá et al. 2014	CE	FZ, ML, JV
Leptodactylus cupreus	Leptodactylidae	DD	Caramaschi et al. 2008	Caramaschi et al. 2008	AF	–
Leptodactylus cupreus	Leptodactylidae	DD	Caramaschi et al. 2008	Sá et al. 2014	AF	–
Leptodactylus didymus	Leptodactylidae	LC	Heyer et al. 1996	Heyer et al. 1996	AM	FZ, ML
Leptodactylus didymus	Leptodactylidae	LC	Heyer et al. 1996	Köhler & Lötters 1999a	AM	FZ, ML
Leptodactylus didymus	Leptodactylidae	LC	Heyer et al. 1996	Duellman 2005	AM	FZ, ML
Leptodactylus didymus	Leptodactylidae	LC	Heyer et al. 1996	Sá et al. 2014	AM	FZ, ML
Leptodactylus diedrus	Leptodactylidae	LC	Heyer 1994	Heyer 1998	AM	ML
Leptodactylus diedrus	Leptodactylidae	LC	Heyer 1994	Sá et al. 2014	AM	ML
Leptodactylus discodactylus	Leptodactylidae	LC	Boulenger 1884	Straughan & Heyer 1976	AM	ML, JV
Leptodactylus discodactylus	Leptodactylidae	LC	Boulenger 1884	Duellman 1978a	AM	ML, JV
Leptodactylus discodactylus	Leptodactylidae	LC	Boulenger 1884	Sá et al. 2014	AM	ML, JV
Leptodactylus elenae	Leptodactylidae	LC	Heyer 1978	Barrio 1965a	CE, PN	FZ, ML, JV
Leptodactylus elenae	Leptodactylidae	LC	Heyer 1978	Márquez et al. 1995	CE, PN	FZ, ML, JV
Leptodactylus elenae	Leptodactylidae	LC	Heyer 1978	Heyer et al. 1996	CE, PN	FZ, ML, JV
Leptodactylus elenae	Leptodactylidae	LC	Heyer 1978	Heyer & Heyer 2002	CE, PN	FZ, ML, JV
Leptodactylus elenae	Leptodactylidae	LC	Heyer 1978	Sá et al. 2014	CE, PN	FZ, ML, JV
Leptodactylus flavopictus	Leptodactylidae	LC	Lutz 1926	Sá et al. 2014	AF	–

Taxa	Family	IUCN	Description Reference	Call_reference	Biome	Sound Library
Leptodactylus furnarius	Leptodactylidae	LC	Sazima & Bokermann 1978	Sazima & Bokermann 1978	AF, CA, CE, PM	FZ, ML, JV
Leptodactylus furnarius	Leptodactylidae	LC	Sazima & Bokermann 1978	Giaretta & Kokubum 2004	AF, CA, CE, PM	FZ, ML, JV
Leptodactylus furnarius	Leptodactylidae	LC	Sazima & Bokermann 1978	Heyer & Heyer 2004	AF, CA, CE, PM	FZ, ML, JV
Leptodactylus furnarius	Leptodactylidae	LC	Sazima & Bokermann 1978	Silva et al. 2008	AF, CA, CE, PM	FZ, ML, JV
Leptodactylus furnarius	Leptodactylidae	LC	Sazima & Bokermann 1978	Sá et al. 2014	AF, CA, CE, PM	FZ, ML, JV
Leptodactylus fuscus	Leptodactylidae	LC	Schneider 1799	Barrio 1965a	AM, AF, CA, CE, PM, PN	FZ, ML, JV
Leptodactylus fuscus	Leptodactylidae	LC	Schneider 1799	Straughan & Heyer 1976	AM, AF, CA, CE, PM, PN	FZ, ML, JV
Leptodactylus fuscus	Leptodactylidae	LC	Schneider 1799	Heyer 1978	AM, AF, CA, CE, PM, PN	FZ, ML, JV
Leptodactylus fuscus	Leptodactylidae	LC	Schneider 1799	Martins 1988	AM, AF, CA, CE, PM, PN	FZ, ML, JV
Leptodactylus fuscus	Leptodactylidae	LC	Schneider 1799	Schneider et al. 1988	AM, AF, CA, CE, PM, PN	FZ, ML, JV
Leptodactylus fuscus	Leptodactylidae	LC	Schneider 1799	Heyer et al. 1990	AM, AF, CA, CE, PM, PN	FZ, ML, JV
Leptodactylus fuscus	Leptodactylidae	LC	Schneider 1799	Márquez et al. 1995	AM, AF, CA, CE, PM, PN	FZ, ML, JV
Leptodactylus fuscus	Leptodactylidae	LC	Schneider 1799	Lescure & Marty 2000	AM, AF, CA, CE, PM, PN	FZ, ML, JV
Leptodactylus fuscus	Leptodactylidae	LC	Schneider 1799	kwet et al. 2001	AM, AF, CA, CE, PM, PN	FZ, ML, JV
Leptodactylus fuscus	Leptodactylidae	LC	Schneider 1799	Heyer & Reid 2003	AM, AF, CA, CE, PM, PN	FZ, ML, JV
Leptodactylus fuscus	Leptodactylidae	LC	Schneider 1799	Bernal et al. 2004	AM, AF, CA, CE, PM, PN	FZ, ML, JV
Leptodactylus fuscus	Leptodactylidae	LC	Schneider 1799	Silva et al. 2008	AM, AF, CA, CE, PM, PN	FZ, ML, JV
Leptodactylus fuscus	Leptodactylidae	LC	Schneider 1799	Tárano 2010	AM, AF, CA, CE, PM, PN	FZ, ML, JV
Leptodactylus fuscus	Leptodactylidae	LC	Schneider 1799	Sá et al. 2014	AM, AF, CA, CE, PM, PN	FZ, ML, JV
Leptodactylus gracilis	Leptodactylidae	LC	Duméril & Bibron 1840	Barrio 1965a	PM	FZ, ML, JV
Leptodactylus gracilis	Leptodactylidae	LC	Duméril & Bibron 1840	Heyer 1978	PM	FZ, ML, JV
Leptodactylus gracilis	Leptodactylidae	LC	Duméril & Bibron 1840	Salas et al. 1998	PM	FZ, ML, JV
Leptodactylus gracilis	Leptodactylidae	LC	Duméril & Bibron 1840	Köhler & Lötters 1999b	PM	FZ, ML, JV
Leptodactylus gracilis	Leptodactylidae	LC	Duméril & Bibron 1840	Köhler 2000	PM	FZ, ML, JV
Leptodactylus gracilis	Leptodactylidae	LC	Duméril & Bibron 1840	kwet et al. 2001	PM	FZ, ML, JV
Leptodactylus gracilis	Leptodactylidae	LC	Duméril & Bibron 1840	Sá et al. 2014	PM	FZ, ML, JV
Leptodactylus guianensis	Leptodactylidae	NE	Heyer & Sá 2011	NA	AM	–

Taxa	Family	IUCN	Description Reference	Call_reference	Biome	Sound Library
Leptodactylus hylodes	Leptodactylidae	DD	Reinhardt & Lütken 1862	NA	AF	–
Leptodactylus jolyi	Leptodactylidae	DD	Sazima & Bokermann 1978	Sazima & Bokermann 1978	AF, CE	FZ, ML, JV
Leptodactylus jolyi	Leptodactylidae	DD	Sazima & Bokermann 1978	Giaretta & Costa 2007	AF, CE	FZ, ML, JV
Leptodactylus jolyi	Leptodactylidae	DD	Sazima & Bokermann 1978	Sá et al. 2014	AF, CE	FZ, ML, JV
Leptodactylus knudseni	Leptodactylidae	LC	Heyer 1972	Heyer 1979	AM	FZ, ML
Leptodactylus knudseni	Leptodactylidae	LC	Heyer 1972	Lescure & Marty 2000	AM	FZ, ML
Leptodactylus knudseni	Leptodactylidae	LC	Heyer 1972	Duellman 2005	AM	FZ, ML
Leptodactylus knudseni	Leptodactylidae	LC	Heyer 1972	Heyer 2005	AM	FZ, ML
Leptodactylus knudseni	Leptodactylidae	LC	Heyer 1972	Heyer & Heyer 2006a	AM	FZ, ML
Leptodactylus knudseni	Leptodactylidae	LC	Heyer 1972	Sá et al. 2014	AM	FZ, ML
Leptodactylus labyrinthicus	Leptodactylidae	LC	Spix 1824	Márquez et al. 1995	AM, AF, CA, CE, PM, PN	FZ, ML, JV
Leptodactylus labyrinthicus	Leptodactylidae	LC	Spix 1824	Heyer 2005	AM, AF, CA, CE, PM, PN	FZ, ML, JV
Leptodactylus labyrinthicus	Leptodactylidae	LC	Spix 1824	Zina & Haddad 2005	AM, AF, CA, CE, PM, PN	FZ, ML, JV
Leptodactylus labyrinthicus	Leptodactylidae	LC	Spix 1824	Silva et al. 2008	AM, AF, CA, CE, PM, PN	FZ, ML, JV
Leptodactylus labyrinthicus	Leptodactylidae	LC	Spix 1824	Sá et al. 2014	AM, AF, CA, CE, PM, PN	FZ, ML, JV
Leptodactylus laticeps	Leptodactylidae	NT	Boulenger 1918	Heyer 2006	PM	FZ
Leptodactylus latinasus	Leptodactylidae	LC	Jiménez de la Espada 1875	Barrio 1965a	CA, PM, PN	FZ, ML, JV
Leptodactylus latinasus	Leptodactylidae	LC	Jiménez de la Espada 1875	Heyer 1978	CA, PM, PN	FZ, ML, JV
Leptodactylus latinasus	Leptodactylidae	LC	Jiménez de la Espada 1875	Salas et al. 1998	CA, PM, PN	FZ, ML, JV
Leptodactylus latinasus	Leptodactylidae	LC	Jiménez de la Espada 1875	Heyer & Juncá 2003	CA, PM, PN	FZ, ML, JV
Leptodactylus latinasus	Leptodactylidae	LC	Jiménez de la Espada 1875	Sá et al. 2014	CA, PM, PN	FZ, ML, JV
Leptodactylus latrans	Leptodactylidae	LC	Steffen 1815	Salas et al. 1998	AF, CA, CE, PM	FZ, ML, JV
Leptodactylus latrans	Leptodactylidae	LC	Steffen 1815	Nunes & Juncá 2006	AF, CA, CE, PM	FZ, ML, JV
Leptodactylus latrans	Leptodactylidae	LC	Steffen 1815	Pombal 2010	AF, CA, CE, PM	FZ, ML, JV
Leptodactylus lauramiriamae	Leptodactylidae	DD	Heyer & Crombie 2005	NA	CE	–
Leptodactylus leptodactyloides	Leptodactylidae	LC	Andersson 1945	Heyer 1994	AM	FZ, ML
Leptodactylus leptodactyloides	Leptodactylidae	LC	Andersson 1945	Márquez et al. 1995	AM	FZ, ML

Taxa	Family	IUCN	Description Reference	Call_reference	Biome	Sound Library
Leptodactylus leptodactyloides	Leptodactylidae	LC	Andersson 1945	Lescure & Marty 2000	AM	FZ, ML
Leptodactylus leptodactyloides	Leptodactylidae	LC	Andersson 1945	Duellman 2005	AM	FZ, ML
Leptodactylus leptodactyloides	Leptodactylidae	LC	Andersson 1945	Sá et al. 2014	AM	FZ, ML
Lithodytes lineatus	Leptodactylidae	LC	Schneider 1799	Schlüter 1980b	AM, CE	FZ, ML, JV
Lithodytes lineatus	Leptodactylidae	LC	Schneider 1799	Márquez et al. 1995	AM, CE	FZ, ML, JV
Lithodytes lineatus	Leptodactylidae	LC	Schneider 1799	Duellman 2005	AM, CE	FZ, ML, JV
Leptodactylus longirostris	Leptodactylidae	LC	Boulenger 1882	Crombie & Heyer 1983	AM	FZ, ML
Leptodactylus longirostris	Leptodactylidae	LC	Boulenger 1882	Lescure & Marty 2000	AM	FZ, ML
Leptodactylus longirostris	Leptodactylidae	LC	Boulenger 1882	Sá et al. 2014	AM	FZ, ML
Leptodactylus macrosternum	Leptodactylidae	LC	Miranda-Ribeiro 1926	Tárano 2010	AM, CA	ML, JV
Leptodactylus marambaiae	Leptodactylidae	LC	Izecksohn 1976a	Heyer 1978	AF	–
Leptodactylus marambaiae	Leptodactylidae	LC	Izecksohn 1976a	Sá et al. 2014	AF	–
Leptodactylus myersi	Leptodactylidae	LC	Heyer 1995	Lescure & Marty 2000	AM	FZ
Leptodactylus myersi	Leptodactylidae	LC	Heyer 1995	Heyer 2005	AM	FZ
Leptodactylus myersi	Leptodactylidae	LC	Heyer 1995	Sá et al. 2014	AM	FZ
Leptodactylus mystaceus	Leptodactylidae	LC	Spix 1824	Duellman 1978a	AF, CA, CE	FZ, ML, JV
Leptodactylus mystaceus	Leptodactylidae	LC	Spix 1824	Heyer 1978	AF, CA, CE	FZ, ML, JV
Leptodactylus mystaceus	Leptodactylidae	LC	Spix 1824	Zimmerman 1983	AF, CA, CE	FZ, ML, JV
Leptodactylus mystaceus	Leptodactylidae	LC	Spix 1824	Zimmerman & Bogart 1984	AF, CA, CE	FZ, ML, JV
Leptodactylus mystaceus	Leptodactylidae	LC	Spix 1824	Márquez et al. 1995	AF, CA, CE	FZ, ML, JV
Leptodactylus mystaceus	Leptodactylidae	LC	Spix 1824	Heyer et al. 1996	AF, CA, CE	FZ, ML, JV
Leptodactylus mystaceus	Leptodactylidae	LC	Spix 1824	Lescure & Marty 2000	AF, CA, CE	FZ, ML, JV
Leptodactylus mystaceus	Leptodactylidae	LC	Spix 1824	Toledo et al. 2005	AF, CA, CE	FZ, ML, JV
Leptodactylus mystaceus	Leptodactylidae	LC	Spix 1824	Sá et al. 2014	AF, CA, CE	FZ, ML, JV
Leptodactylus mystacinus	Leptodactylidae	LC	Burmeister 1861	Fouquette 1960	AF, CE, PM, PN	FZ, ML, JV
Leptodactylus mystacinus	Leptodactylidae	LC	Burmeister 1861	Barrio 1965a	AF, CE, PM, PN	FZ, ML, JV
Leptodactylus mystacinus	Leptodactylidae	LC	Burmeister 1861	Straughan & Heyer 1976	AF, CE, PM, PN	FZ, ML, JV

Taxa	Family	IUCN	Description Reference	Call_reference	Biome	Sound Library
Leptodactylus mystacinus	Leptodactylidae	LC	Burmeister 1861	Salas et al. 1998	AF, CE, PM, PN	FZ, ML, JV
Leptodactylus mystacinus	Leptodactylidae	LC	Burmeister 1861	Abrunhosa et al. 2001	AF, CE, PM, PN	FZ, ML, JV
Leptodactylus mystacinus	Leptodactylidae	LC	Burmeister 1861	Oliveira-Filho & Giaretta 2008	AF, CE, PM, PN	FZ, ML, JV
Leptodactylus natalensis	Leptodactylidae	LC	Lutz 1930b	Heyer & Heyer 2006b	AF, CA	ML, JV
Leptodactylus natalensis	Leptodactylidae	LC	Lutz 1930b	Sá et al. 2014	AF, CA	ML, JV
Leptodactylus notoaktites	Leptodactylidae	LC	Heyer 1978	Heyer et al. 1996	AF, PM	FZ, ML, JV
Leptodactylus notoaktites	Leptodactylidae	LC	Heyer 1978	Sá et al. 2014	AF, PM	FZ, ML, JV
Leptodactylus ochraceus	Leptodactylidae	NE	Lutz & Lutz 1939	NA	AF	–
Leptodactylus oreomantis	Leptodactylidae	NE	Carvalho et al. 2013b	Carvalho et al. 2013b	CE	–
Leptodactylus oreomantis	Leptodactylidae	NE	Carvalho et al. 2013b	Sá et al. 2014	CE	–
Leptodactylus paraensis	Leptodactylidae	LC	Heyer 2005	NA	AM	–
Leptodactylus pentadactylus	Leptodactylidae	LC	Laurenti 1768	Fouquette 1960	AM	FZ, ML, JV
Leptodactylus pentadactylus	Leptodactylidae	LC	Laurenti 1768	Straughan & Heyer 1976	AM	FZ, ML, JV
Leptodactylus pentadactylus	Leptodactylidae	LC	Laurenti 1768	Duellman 1978a	AM	FZ, ML, JV
Leptodactylus pentadactylus	Leptodactylidae	LC	Laurenti 1768	Heyer 1979	AM	FZ, ML, JV
Leptodactylus pentadactylus	Leptodactylidae	LC	Laurenti 1768	Schlüter 1980b	AM	FZ, ML, JV
Leptodactylus pentadactylus	Leptodactylidae	LC	Laurenti 1768	Zimmerman 1983	AM	FZ, ML, JV
Leptodactylus pentadactylus	Leptodactylidae	LC	Laurenti 1768	Zimmerman & Bogart 1984	AM	FZ, ML, JV
Leptodactylus pentadactylus	Leptodactylidae	LC	Laurenti 1768	Schneider et al. 1988	AM	FZ, ML, JV
Leptodactylus pentadactylus	Leptodactylidae	LC	Laurenti 1768	Kime et al. 2000	AM	FZ, ML, JV
Leptodactylus pentadactylus	Leptodactylidae	LC	Laurenti 1768	Lescure & Marty 2000	AM	FZ, ML, JV
Leptodactylus pentadactylus	Leptodactylidae	LC	Laurenti 1768	Duellman 2005	AM	FZ, ML, JV
Leptodactylus pentadactylus	Leptodactylidae	LC	Laurenti 1768	Heyer 2005	AM	FZ, ML, JV
Leptodactylus pentadactylus	Leptodactylidae	LC	Laurenti 1768	Sá et al. 2014	AM	FZ, ML, JV
Leptodactylus petersii	Leptodactylidae	LC	Steindachner 1864	Heyer 1994	AM, CE	FZ, ML, JV
Leptodactylus petersii	Leptodactylidae	LC	Steindachner 1864	Lescure & Marty 2000	AM, CE	FZ, ML, JV
Leptodactylus petersii	Leptodactylidae	LC	Steindachner 1864	Duellman 2005	AM, CE	FZ, ML, JV

Taxa	Family	IUCN	Description Reference	Call_reference	Biome	Sound Library
Leptodactylus petersii	Leptodactylidae	LC	Steindachner 1864	Tárano 2010	AM, CE	FZ, ML, JV
Leptodactylus plaumanni	Leptodactylidae	LC	Ahl 1936	Barrio 1973	AF, CE, PM	FZ, ML, JV
Leptodactylus plaumanni	Leptodactylidae	LC	Ahl 1936	Heyer 1978	AF, CE, PM	FZ, ML, JV
Leptodactylus plaumanni	Leptodactylidae	LC	Ahl 1936	Péres & Heyer 1993	AF, CE, PM	FZ, ML, JV
Leptodactylus plaumanni	Leptodactylidae	LC	Ahl 1936	kwet et al. 2001	AF, CE, PM	FZ, ML, JV
Leptodactylus plaumanni	Leptodactylidae	LC	Ahl 1936	Sá et al. 2014	AF, CE, PM	FZ, ML, JV
Leptodactylus podicipinus	Leptodactylidae	LC	Cope 1862a	Barrio 1965a	AF, CA, CE, PM, PN	FZ, ML, JV
Leptodactylus podicipinus	Leptodactylidae	LC	Cope 1862a	Heyer 1994	AF, CA, CE, PM, PN	FZ, ML, JV
Leptodactylus podicipinus	Leptodactylidae	LC	Cope 1862a	Márquez et al. 1995	AF, CA, CE, PM, PN	FZ, ML, JV
Leptodactylus podicipinus	Leptodactylidae	LC	Cope 1862a	Guimarães et al. 2001	AF, CA, CE, PM, PN	FZ, ML, JV
Leptodactylus podicipinus	Leptodactylidae	LC	Cope 1862a	Silva et al. 2008	AF, CA, CE, PM, PN	FZ, ML, JV
Leptodactylus podicipinus	Leptodactylidae	LC	Cope 1862a	Sá et al. 2014	AF, CA, CE, PM, PN	FZ, ML, JV
Leptodactylus pustulatus	Leptodactylidae	LC	Peters 1870	Brandão & Heyer 2005	CE	FZ
Leptodactylus pustulatus	Leptodactylidae	LC	Peters 1870	Sá et al. 2014	CE	FZ
Leptodactylus rhodomystax	Leptodactylidae	LC	Boulenger 1884	Zimmerman & Bogart 1984	AM	FZ, ML, JV
Leptodactylus rhodomystax	Leptodactylidae	LC	Boulenger 1884	Lescure & Marty 2000	AM	FZ, ML, JV
Leptodactylus rhodomystax	Leptodactylidae	LC	Boulenger 1884	Sá et al. 2014	AM	FZ, ML, JV
Leptodactylus rhodonotus	Leptodactylidae	LC	Günther 1869	Köhler & Lötters 1999b	AM	FZ
Leptodactylus rhodonotus	Leptodactylidae	LC	Günther 1869	Köhler 2000	AM	FZ
Leptodactylus rhodonotus	Leptodactylidae	LC	Günther 1869	Duellman 2005	AM	FZ
Leptodactylus rhodonotus	Leptodactylidae	LC	Günther 1869	Sá et al. 2014	AM	FZ
Leptodactylus riveroi	Leptodactylidae	LC	Heyer & Pyburn 1983	Heyer & Pyburn 1983	AM	ML
Leptodactylus riveroi	Leptodactylidae	LC	Heyer & Pyburn 1983	Sá et al. 2014	AM	ML
Leptodactylus rugosus	Leptodactylidae	LC	Noble 1923	Heyer 1979	AM	FZ, ML, JV
Leptodactylus rugosus	Leptodactylidae	LC	Noble 1923	Duellman 1997	AM	FZ, ML, JV
Leptodactylus rugosus	Leptodactylidae	LC	Noble 1923	Heyer & Thompson 2000	AM	FZ, ML, JV
Leptodactylus rugosus	Leptodactylidae	LC	Noble 1923	Heyer & Barrio-Amorós 2009	AM	FZ, ML, JV

Taxa	Family	IUCN	Description Reference	Call_reference	Biome	Sound Library
Leptodactylus rugosus	Leptodactylidae	LC	Noble 1923	Sá et al. 2014	AM	FZ, ML, JV
Leptodactylus sabanensis	Leptodactylidae	LC	Heyer 1994	Heyer 1994	AM	ML
Leptodactylus sabanensis	Leptodactylidae	LC	Heyer 1994	Sá et al. 2014	AM	ML
Leptodactylus sertanejo	Leptodactylidae	LC	Giaretta & Costa 2007	Giaretta & Costa 2007	CE	–
Leptodactylus sertanejo	Leptodactylidae	LC	Giaretta & Costa 2007	Sá et al. 2014	CE	–
Leptodactylus spixi	Leptodactylidae	LC	Heyer 1983a	Bilate et al. 2007	AF	–
Leptodactylus spixi	Leptodactylidae	LC	Heyer 1983a	Sá et al. 2014	AF	–
Leptodactylus stenodema	Leptodactylidae	LC	Jiménez de la Espada 1875	Heyer 1979	AM	FZ, ML, JV
Leptodactylus stenodema	Leptodactylidae	LC	Jiménez de la Espada 1875	Zimmerman 1983	AM	FZ, ML, JV
Leptodactylus stenodema	Leptodactylidae	LC	Jiménez de la Espada 1875	Zimmerman & Bogart 1984	AM	FZ, ML, JV
Leptodactylus stenodema	Leptodactylidae	LC	Jiménez de la Espada 1875	Lescure & Marty 2000	AM	FZ, ML, JV
Leptodactylus stenodema	Leptodactylidae	LC	Jiménez de la Espada 1875	Sá et al. 2014	AM	FZ, ML, JV
Leptodactylus syphax	Leptodactylidae	LC	Bokermann 1969	Bokermann 1969	CA, CE, PN	FZ, ML, JV
Leptodactylus syphax	Leptodactylidae	LC	Bokermann 1969	Heyer 1979	CA, CE, PN	FZ, ML, JV
Leptodactylus syphax	Leptodactylidae	LC	Bokermann 1969	Cardoso & Heyer 1995	CA, CE, PN	FZ, ML, JV
Leptodactylus syphax	Leptodactylidae	LC	Bokermann 1969	Heyer et al. 2010	CA, CE, PN	FZ, ML, JV
Leptodactylus syphax	Leptodactylidae	LC	Bokermann 1969	Sá et al. 2014	CA, CE, PN	FZ, ML, JV
Leptodactylus tapiti	Leptodactylidae	DD	Sazima & Bokermann 1978	Brandão et al. 2013a	CE	–
Leptodactylus tapiti	Leptodactylidae	DD	Sazima & Bokermann 1978	Sá et al. 2014	CE	–
Leptodactylus troglodytes	Leptodactylidae	LC	Lutz 1926	Heyer 1978	AF, CA, CE	FZ, ML, JV
Leptodactylus troglodytes	Leptodactylidae	LC	Lutz 1926	Nunes & Juncá 2006	AF, CA, CE	FZ, ML, JV
Leptodactylus troglodytes	Leptodactylidae	LC	Lutz 1926	Sá et al. 2014	AF, CA, CE	FZ, ML, JV
Leptodactylus validus	Leptodactylidae	LC	Garman 1888	Heyer 1994	AM	FZ, ML
Leptodactylus validus	Leptodactylidae	LC	Garman 1888	Sá et al. 2014	AM	FZ, ML
Leptodactylus vastus	Leptodactylidae	LC	Lutz 1930b	Heyer 2005	AF, CA, CE	JV
Leptodactylus vastus	Leptodactylidae	LC	Lutz 1930b	Jansen & Schulze 2012	AF, CA, CE	JV
Leptodactylus vastus	Leptodactylidae	LC	Lutz 1930b	Sá et al. 2014	AF, CA, CE	JV

Taxa	Family	IUCN	Description Reference	Call_reference	Biome	Sound Library
Leptodactylus viridis	Leptodactylidae	DD	Jim & Spirandeli-Cruz 1973	Rocha et al. 2016a	AF	JV
Leptodactylus wagneri	Leptodactylidae	LC	Peters 1862	Straughan & Heyer 1976	AM	FZ, ML, JV
Leptodactylus wagneri	Leptodactylidae	LC	Peters 1862	Hödl 1977	AM	FZ, ML, JV
Leptodactylus wagneri	Leptodactylidae	LC	Peters 1862	Duellman 1978a	AM	FZ, ML, JV
Leptodactylus wagneri	Leptodactylidae	LC	Peters 1862	Schlüter 1980b	AM	FZ, ML, JV
Leptodactylus wagneri	Leptodactylidae	LC	Peters 1862	Zimmerman 1983	AM	FZ, ML, JV
Leptodactylus wagneri	Leptodactylidae	LC	Peters 1862	Zimmerman & Bogart 1984	AM	FZ, ML, JV
Leptodactylus wagneri	Leptodactylidae	LC	Peters 1862	Schneider et al. 1988	AM	FZ, ML, JV
Leptodactylus wagneri	Leptodactylidae	LC	Peters 1862	Cardoso & Vielliard 1990	AM	FZ, ML, JV
Crossodactylodes bokermanni	Leptodactylidae	NT	Reinhardt & Lütken 1862	NA	AF	–
Crossodactylodes itambe	Leptodactylidae	NE	Barata et al. 2013	NA	CE	–
Crossodactylodes izecksohni	Leptodactylidae	NT	Peixoto 1982a	NA	AF	–
Crossodactylodes pintoi	Leptodactylidae	DD	Cochran 1938	NA	AF	–
Crossodactylodes septentrionalis	Leptodactylidae	NE	Teixeira et al. 2013b	NA	AF	–
Paratelmatobius cardosoi	Leptodactylidae	DD	Pombal & Haddad 1999	Pombal & Haddad 1999	AF	FZ
Paratelmatobius gaigeae	Leptodactylidae	DD	Cochran 1938	Giaretta & Magrini 2013	AF	–
Paratelmatobius gaigeae	Leptodactylidae	DD	Cochran 1938	Domenico et al. 2014	AF	–
Paratelmatobius lutzii	Leptodactylidae	DD	Lutz & Carvalho 1958	NA	AF	–
Paratelmatobius mantiqueira	Leptodactylidae	DD	Pombal & Haddad 1999	NA	AF	–
Paratelmatobius poecilogaster	Leptodactylidae	DD	Giaretta & Castanho 1990	Giaretta & Castanho 1990	AF	FZ
Paratelmatobius yepiranga	Leptodactylidae	NE	Garcia et al. 2009	Garcia et al. 2009	AF	–
Rupirana cardosoi	Leptodactylidae	NT	Heyer 1999	Juncá & Lugli 2009	AF, CA, CE	–
Scythrophrys sawayae	Leptodactylidae	LC	Cochran 1953	NA	AF	JV
Adelastes hylonomus	Microhylidae	NT	Zweifel 1986	Zweifel 1986	AM	ML
Arcovomer passarellii	Microhylidae	LC	Carvalho 1954	Nelson 1973	AF	FZ, JV
Arcovomer passarellii	Microhylidae	LC	Carvalho 1954	Giaretta & Martins 2009	AF	FZ, JV
Chiasmocleis alagoanus	Microhylidae	NE	Cruz et al. 1999	NA	AF	–

Taxa	Family	IUCN	Description Reference	Call_reference	Biome	Sound Library
Chiasmocleis albopunctata	Microhylidae	LC	Boettger 1885	Nelson 1973	AM, AF, CE, PN	FZ, JV
Chiasmocleis albopunctata	Microhylidae	LC	Boettger 1885	De La Riva et al. 1996b	AM, AF, CE, PN	FZ, JV
Chiasmocleis albopunctata	Microhylidae	LC	Boettger 1885	Köhler 2000	AM, AF, CE, PN	FZ, JV
Chiasmocleis albopunctata	Microhylidae	LC	Boettger 1885	Oliveira-Filho & Giaretta 2006	AM, AF, CE, PN	FZ, JV
Chiasmocleis antenori	Microhylidae	LC	Walker 1973	NA	AM	–
Chiasmocleis atlantica	Microhylidae	LC	Cruz et al. 1997	Wogel et al. 2004b	AF, CE	–
Chiasmocleis avilapiresae	Microhylidae	LC	Peloso & Sturaro 2008	Barros et al. 2010	AM	–
Chiasmocleis bassleri	Microhylidae	LC	Dunn 1949	Santana et al. 2009	AM	FZ, JV
Chiasmocleis capixaba	Microhylidae	LC	Cruz et al. 1997	Wogel et al. 2004b	AF	–
Chiasmocleis carvalhoi	Microhylidae	LC	Nelson 1975	Hartmann et al. 2002	AF	FZ
Chiasmocleis carvalhoi	Microhylidae	LC	Nelson 1975	Wogel et al. 2004b	AF	FZ
Chiasmocleis centralis	Microhylidae	DD	Bokermann 1952	NA	CE	–
Chiasmocleis cordeiroi	Microhylidae	DD	Caramaschi & Pimenta 2003	Forlani et al. 2013	AF	–
Chiasmocleis crucis	Microhylidae	DD	Caramaschi & Pimenta 2003	Forlani et al. 2013	AF	–
Chiasmocleis gnoma	Microhylidae	DD	Canedo et al. 2004	NA	AF	–
Chiasmocleis haddadi	Microhylidae	NE	Peloso et al. 2014	Peloso et al. 2014	AM	JV
Chiasmocleis hudsoni	Microhylidae	LC	Parker 1940	Rodrigues et al. 2008	AM	ML, JV
Chiasmocleis hudsoni	Microhylidae	LC	Parker 1940	Peloso et al. 2014	AM	ML, JV
Chiasmocleis lacrimae	Microhylidae	NE	Peloso et al. 2014	NA	AF	–
Chiasmocleis leucosticta	Microhylidae	LC	Boulenger 1888a	Nelson 1973	AF	FZ, JV
Chiasmocleis mantiqueira	Microhylidae	DD	Cruz et al. 2007c	Santana et al. 2012b	AF	–
Chiasmocleis mehelyi	Microhylidae	DD	Caramaschi & Cruz 1997	Hartmann et al. 2002	AF, PN	FZ
Chiasmocleis papachibe	Microhylidae	NE	Peloso et al. 2014	NA	AM	–
Chiasmocleis quilombola	Microhylidae	NE	Tonini et al. 2014	NA	AF	–
Chiasmocleis royi	Microhylidae	NE	Peloso et al. 2014	Peloso et al. 2014	AM	ML
Chiasmocleis sapiranga	Microhylidae	DD	Cruz et al. 2007a	NA	AF	–
Chiasmocleis schubarti	Microhylidae	LC	Bokermann 1952	Nelson 1973	AF, CA	–

Taxa	Family	IUCN	Description Reference	Call_reference	Biome	Sound Library
Chiasmocleis shudikarensis	Microhylidae	LC	Dunn 1949	Zimmerman & Bogart 1988	AM	FZ, ML, JV
Chiasmocleis shudikarensis	Microhylidae	LC	Dunn 1949	Lescure & Marty 2000	AM	FZ, ML, JV
Chiasmocleis supercilialba	Microhylidae	NE	Morales & McDiarmid 2009	Morales & McDiarmid 2009	AM	–
Chiasmocleis tridactyla	Microhylidae	LC	Duellman & Mendelson 1995	NA	AM	–
Chiasmocleis ventrimaculata	Microhylidae	LC	Andersson 1945	Nelson 1973	AM	FZ, ML
Chiasmocleis ventrimaculata	Microhylidae	LC	Andersson 1945	Schlüter 1980a	AM	FZ, ML
Chiasmocleis ventrimaculata	Microhylidae	LC	Andersson 1945	Duellman 2005	AM	FZ, ML
Ctenophryne geayi	Microhylidae	LC	Mocquard 1904	Nelson 1973	AM	FZ, ML
Ctenophryne geayi	Microhylidae	LC	Mocquard 1904	Schlüter 1980a	AM	FZ, ML
Ctenophryne geayi	Microhylidae	LC	Mocquard 1904	Zweifel & Myers 1989	AM	FZ, ML
Ctenophryne geayi	Microhylidae	LC	Mocquard 1904	Duellman 1997	AM	FZ, ML
Ctenophryne geayi	Microhylidae	LC	Mocquard 1904	Duellman 2005	AM	FZ, ML
Dasypops schirchi	Microhylidae	VU	Miranda-Ribeiro 1924	Nelson 1973	AF	JV
Dermatonotus muelleri	Microhylidae	LC	Boettger 1885	Nelson 1973	CA, CE, PN	FZ, JV
Dermatonotus muelleri	Microhylidae	LC	Boettger 1885	Giaretta et al. 2013	CA, CE, PN	FZ, JV
Dermatonotus muelleri	Microhylidae	LC	Boettger 1885	Giaretta et al. 2015	CA, CE, PN	FZ, JV
Elachistocleis bicolor	Microhylidae	LC	Guérin-Menéville 1838	De La Riva et al. 1996b	AF, CE, PM, PN	FZ, ML, JV
Elachistocleis bicolor	Microhylidae	LC	Guérin-Menéville 1838	Kwet & Di-Bernardo 1998	AF, CE, PM, PN	FZ, ML, JV
Elachistocleis bicolor	Microhylidae	LC	Guérin-Menéville 1838	Silva et al. 2008	AF, CE, PM, PN	FZ, ML, JV
Elachistocleis bumbameuboi	Microhylidae	DD	Caramaschi 2010b	NA	AM	–
Elachistocleis carvalhoi	Microhylidae	LC	Caramaschi 2010b	NA	AM	–
Elachistocleis cesarii	Microhylidae	NE	Miranda-Ribeiro 1920f	Haddad et al. 1988	AF, CE	JV
Elachistocleis cesarii	Microhylidae	NE	Miranda-Ribeiro 1920f	Toledo et al. 2010b	AF, CE	JV
Elachistocleis helianneae	Microhylidae	LC	Caramaschi 2010b	Fonseca et al. 2012	AM	–
Elachistocleis erythrogaster	Microhylidae	NT	Kwet & Di-Bernardo 1998	Kwet & Di-Bernardo 1998	AF, CE, PM	FZ
Elachistocleis magnus	Microhylidae	NE	Toledo 2010	NA	AM, CE	–
Elachistocleis matogrosso	Microhylidae	LC	Caramaschi 2010b	NA	CE	–

Taxa	Family	IUCN	Description Reference	Call_reference	Biome	Sound Library
Elachistocleis muiraquitan	Microhylidae	NE	Nunes-de-Almeida & Toledo 2012	Nunes-de-Almeida & Toledo 2012	AM	JV
Elachistocleis ovalis	Microhylidae	LC	Schneider 1799	Nelson 1973	AM	FZ, ML
Elachistocleis ovalis	Microhylidae	LC	Schneider 1799	De La Riva et al. 1996b	AM	FZ, ML
Elachistocleis ovalis	Microhylidae	LC	Schneider 1799	Köhler 2000	AM	FZ, ML
Elachistocleis ovalis	Microhylidae	LC	Schneider 1799	Lescure & Marty 2000	AM	FZ, ML
Elachistocleis piauiensis	Microhylidae	LC	Caramaschi & Jim 1983a	Nunes et al. 2010a	CA, CE	–
Elachistocleis piauiensis	Microhylidae	LC	Caramaschi & Jim 1983a	Toledo et al. 2010b	CA, CE	–
Elachistocleis surumu	Microhylidae	DD	Caramaschi 2010b	NA	AM	–
Hamptophryne alios	Microhylidae	DD	Wild 1995	NA	AM	–
Hamptophryne boliviana	Microhylidae	LC	Parker 1927b	Nelson 1973	AM	FZ, ML, JV
Hamptophryne boliviana	Microhylidae	LC	Parker 1927b	De La Riva et al. 1996b	AM	FZ, ML, JV
Hamptophryne boliviana	Microhylidae	LC	Parker 1927b	Schlüter 1980a	AM	FZ, ML, JV
Hamptophryne boliviana	Microhylidae	LC	Parker 1927b	Lescure & Marty 2000	AM	FZ, ML, JV
Hamptophryne boliviana	Microhylidae	LC	Parker 1927b	Duellman 2005	AM	FZ, ML, JV
Myersiella microps	Microhylidae	LC	Duméril & Bibron 1841	Hartmann et al. 2002	AF	FZ
Stereocyclops histrio	Microhylidae	DD	Carvalho 1954	NA	AF	–
Stereocyclops incrassatus	Microhylidae	LC	Cope 1870	Nelson 1973	AF	JV
Stereocyclops palmipes	Microhylidae	NE	Caramaschi et al. 2012	NA	AF	–
Stereocyclops parkeri	Microhylidae	LC	Wettstein 1934	NA	AF	–
Otophryne pyburni	Microhylidae	LC	Campbell & Clarke 1998	Lescure & Marty 2000	AM	FZ, ML
Otophryne pyburni	Microhylidae	LC	Campbell & Clarke 1998	MacCulloch et al. 2015	AM	FZ, ML
Synapturanus mirandaribeiroi	Microhylidae	LC	Gallardo 1965	Zimmerman 1983	AM	FZ
Synapturanus mirandaribeiroi	Microhylidae	LC	Gallardo 1965	Lescure & Marty 2000	AM	FZ
Synapturanus mirandaribeiroi	Microhylidae	LC	Gallardo 1965	Menin et al. 2007	AM	FZ
Synapturanus salseri	Microhylidae	LC	Pyburn 1975	Zimmerman 1983	AM	ML, JV
Synapturanus salseri	Microhylidae	LC	Pyburn 1975	Zimmerman & Bogart 1984	AM	ML, JV
Synapturanus salseri	Microhylidae	LC	Pyburn 1975	Menin et al. 2007	AM	ML, JV

Taxa	Family	IUCN	Description Reference	Call_reference	Biome	Sound Library
Macrogenioglottus alipioi	Odontophrynidae	LC	Carvalho 1946	Abravaya & Jackson 1978	AF	FZ, JV
Odontophrynus americanus	Odontophrynidae	LC	Duméril & Bibron 1841	Márquez et al. 1995	AF, CE, PM, PN	FZ, JV
Odontophrynus americanus	Odontophrynidae	LC	Duméril & Bibron 1841	Salas et al. 1998	AF, CE, PM, PN	FZ, JV
Odontophrynus americanus	Odontophrynidae	LC	Duméril & Bibron 1841	Köhler 2000	AF, CE, PM, PN	FZ, JV
Odontophrynus carvalhoi	Odontophrynidae	LC	Duméril & Bibron 1841	Caramaschi & Napoli 2012	AF, CA	JV
Odontophrynus cultripes	Odontophrynidae	LC	Reinhardt & Lütken 1862	Caramaschi & Napoli 2012	AF, CE	JV
Odontophrynus lavillai	Odontophrynidae	LC	Cei 1985	Köhler 2000	PN	FZ
Odontophrynus lavillai	Odontophrynidae	LC	Cei 1985	Rosset & Baldo 2014	PN	FZ
Odontophrynus maisuma	Odontophrynidae	NE	Rosset 2008	Borteiro et al. 2010	AF, PM	–
Odontophrynus monachus	Odontophrynidae	NE	Caramaschi & Napoli 2012	Caramaschi & Napoli 2012	CE	JV
Odontophrynus salvatori	Odontophrynidae	DD	Caramaschi 1996	Bastos et al. 2011a	CE	–
Proceratophrys appendiculata	Odontophrynidae	LC	Günther 1873	Dias et al. 2003	AF	–
Proceratophrys aridus	Odontophrynidae	NE	Cruz et al. 2012	NA	CA	–
Proceratophrys avelinoi	Odontophrynidae	LC	Mercadal del Barrio & Barrio 1993	Kwet & Baldo 2003	AF	–
Proceratophrys avelinoi	Odontophrynidae	LC	Mercadal del Barrio & Barrio 1993	Lima 2007	AF	–
Proceratophrys bagnoi	Odontophrynidae	NE	Brandão et al. 2013b	NA	CE	–
Proceratophrys bigibbosa	Odontophrynidae	NT	Peters 1872a	Kwet & Faivovich 2001	AF	FZ, JV
Proceratophrys belzebul	Odontophrynidae	NE	Dias et al. 2013	NA	AF	–
Proceratophrys boiei	Odontophrynidae	LC	Wied-Neuwied 1824c	Heyer et al. 1990	AF, CE	FZ, ML, JV
Proceratophrys boiei	Odontophrynidae	LC	Wied-Neuwied 1824c	Pombal 2010	AF, CE	FZ, ML, JV
Proceratophrys branti	Odontophrynidae	NE	Brandão et al. 2013b	NA	CE	JV
Proceratophrys brauni	Odontophrynidae	LC	Kwet & Faivovich 2001	Kwet & Faivovich 2001	AF	FZ, JV
Proceratophrys caramaschii	Odontophrynidae	NE	Cruz et al. 2012	Nunes et al. 2015	AF	–
Proceratophrys carranca	Odontophrynidae	NE	Godinho et al. 2013	Godinho et al. 2013	CE	–
Proceratophrys concavitympanum	Odontophrynidae	DD	Giaretta et al. 2000	Santana et al. 2010	CE	–
Proceratophrys cristiceps	Odontophrynidae	LC	Müller 1883	Nunes & Juncá 2006	AF, CA	JV
Proceratophrys cururu	Odontophrynidae	DD	Eterovick & Sazima 1998	Eterovick & Sazima 1998	AF, CE	JV

Taxa	Family	IUCN	Description Reference	Call_reference	Biome	Sound Library
Proceratophrys dibernardoi	Odontophrynidae	NE	Brandão et al. 2013b	Ferreira et al. 2016	CE	–
Proceratophrys gladius	Odontophrynidae	NE	Mângia et al. 2014	NA	AF	–
Proceratophrys goyana	Odontophrynidae	LC	Miranda-Ribeiro 1937b	Martins & Giaretta 2013	CE	–
Proceratophrys huntingtoni	Odontophrynidae	NE	Ávila et al. 2012b	Ávila et al. 2012b	CE	–
Proceratophrys itamari	Odontophrynidae	NE	Mângia et al. 2014	Mângia et al. 2014	AF	JV
Proceratophrys izecksohni	Odontophrynidae	NE	Dias et al. 2013	NA	AF	–
Proceratophrys laticeps	Odontophrynidae	LC	Izecksohn & Peixoto 1981	NA	AF	–
Proceratophrys mantiqueira	Odontophrynidae	NE	Mângia et al. 2014	Mângia et al. 2010	AF	JV
Proceratophrys melanopogon	Odontophrynidae	LC	Miranda-Ribeiro 1926	Mângia et al. 2010	AF	JV
Proceratophrys minuta	Odontophrynidae	NE	Napoli et al. 2011b	NA	AF	–
Proceratophrys moehringi	Odontophrynidae	DD	Weygoldt & Peixoto 1985	Weygoldt & Peixoto 1985	AF	–
Proceratophrys moratoi	Odontophrynidae	CR	Jim & Caramaschi 1980	Brasileiro et al. 2008	CE	JV
Proceratophrys moratoi	Odontophrynidae	CR	Jim & Caramaschi 1980	Martins & Giaretta 2012b	CE	JV
Proceratophrys moratoi	Odontophrynidae	CR	Jim & Caramaschi 1980	Forti et al. 2016	CE	JV
Proceratophrys palustris	Odontophrynidae	DD	Giaretta & Sazima 1993	Martins & Giaretta 2012b	AF	JV
Proceratophrys paviotii	Odontophrynidae	DD	Cruz et al. 2005	Cruz et al. 2005	AF	–
Proceratophrys phyllostomus	Odontophrynidae	DD	Izecksohn et al. 1998	NA	AF	–
Proceratophrys pombali	Odontophrynidae	NE	Mângia et al. 2014	Malagoli et al. 2016	AF	–
Proceratophrys redacta	Odontophrynidae	NE	Teixeira et al. 2012b	NA	CA	–
Proceratophrys renalis	Odontophrynidae	NE	Miranda-Ribeiro 1920b	Santana et al. 2011b	AF, CA	JV
Proceratophrys rotundipalpebra	Odontophrynidae	NE	Martins & Giaretta 2013	Martins & Giaretta 2013	CE	–
Proceratophrys rondonae	Odontophrynidae	NE	Prado & Pombal 2008	NA	AM	–
Proceratophrys sanctaritae	Odontophrynidae	NE	Cruz & Napoli 2010	Cruz & Napoli 2010	AF	–
Proceratophrys schirchi	Odontophrynidae	LC	Miranda-Ribeiro 1937b	NA	AF, CA	–
Proceratophrys strussmannae	Odontophrynidae	NE	Ávila et al. 2011	NA	CE	–
Proceratophrys subguttata	Odontophrynidae	LC	Izecksohn et al. 1998	NA	AF	–
Proceratophrys tupinamba	Odontophrynidae	NE	Prado & Pombal 2008	NA	AF	–

Taxa	Family	IUCN	Description Reference	Call_reference	Biome	Sound Library
Proceratophrys vielliardi	Odontophrynidae	DD	Martins & Giaretta 2011	Martins & Giaretta 2011	CE	–
Pipa arrabali	Pipidae	LC	Izecksohn 1976b	NA	AM	ML
Pipa carvalhoi	Pipidae	LC	Miranda-Ribeiro 1937c	Weygoldt 1976	AF, CA	–
Pipa carvalhoi	Pipidae	LC	Miranda-Ribeiro 1937c	Bünten et al. 1992	AF, CA	–
Pipa pipa	Pipidae	LC	Linnaeus 1758	NA	AF, CE	JV
Pipa snethlageae	Pipidae	LC	Müller 1914	NA	AM	–
Lithobates catesbeianus	Ranidae	LC	Shaw 1802	Caram et al. 2014	AF, CE, PM	FZ, ML
Lithobates catesbeianus	Ranidae	LC	Shaw 1802	Bee & Gerhardt 2001	AF, CE, PM	FZ, ML
Lithobates catesbeianus	Ranidae	LC	Shaw 1802	Simmons 2004	AF, CE, PM	FZ, ML
Lithobates catesbeianus	Ranidae	LC	Shaw 1802	Llusia et al. 2013	AF, CE, PM	FZ, ML
Lithobates palmipes	Ranidae	LC	Spix 1824	Greding 1976	AF, AM, CE	FZ, ML, JV

6
