# Supplementary material for: The advertisement calls of Brazilian anurans: Historical review, current knowledge and future directions
Source: PLoS One. 2018 Jan 30;13(1):e0191691. doi: 10.1371/journal.pone.0191691 (PMC5790252; doi:10.1371/journal.pone.0191691)
Supplement: S2 Table — (PDF) [file pone.0191691.s002.pdf]

S2 Table. Number and percentage of Brazilian anuran species with described calls per each biome. Species = Total number of species; Calls = Species with described calls; Calls >1 = Species with more than one description of their calls; Endemic Spp = Endemic species; ECalls = Endemic species with described calls; ECalls >1 = Endemic species with more than one description of their calls. Percentages in the first three columns refer to Species (the total number of species), while percentages in the two last columns refer to Endemic Spp (number of endemic species).

**Biomes**	**Species**	**Calls**	**Calls >1**	**Endemic Spp**	**ECalls**	**ECall >1**
Atlantic Forest	601	391<br>65.1%	139<br>23.1%	404<br>67.2%	222<br>54.9%	54<br>13.4%
Amazon	302	216<br>71.5%	123<br>40.7%	272<br>90.1%	187<br>68.7%	95<br>34.9%
Caatinga	117	102<br>87.2%	45<br>38.5%	5<br>4.3%	3<br>60.0%	1<br>20.0%
Cerrado	236	197<br>83.5%	110<br>46.6%	105<br>44.5%	81<br>77.1%	31<br>29.5%
Pampa	93	78<br>83.9%	50<br>53.8%	13<br>14.0%	9<br>69.2%	2<br>15.4%
Pantanal	53	48<br>90.6%	38<br>71.7%	3<br>5.7%	3<br>100%	2<br>66.7%
