# Supplementary material for: The advertisement calls of Brazilian anurans: Historical review, current knowledge and future directions
Source: PLoS One. 2018 Jan 30;13(1):e0191691. doi: 10.1371/journal.pone.0191691 (PMC5790252; doi:10.1371/journal.pone.0191691)
Supplement: S1 Text — (PDF) [file pone.0191691.s003.pdf]

S1 Text. Collected information and metadata variables from each study (only presence or absence).

(i) Locality: Specific site (with coordinates), state or county where the species call was recorded. Works with only country information were not considered.

(ii) Date: Days, months or years when the species call was recorded.

(iii) Water temperature; (iv) Air temperature; and (v) Relative Humidity: data obtained directly from the environment in which the species was recorded. Climatological data or data unrelated to the recorded calls were not considered.

(vi) Activity period: the period of calling activity described in the methodology, results and/or discussion. The period could be the hour or the period of day (day, twilight or night).

(vii) Habitat: the type of substract that the species typically uses to vocalize (soil, litter, leaf, bush, bromeliad, creek, pond, etc.).

(viii) Perch height: the height at which the species was perched while calling, described in the methodology, results and/or discussion. When there was information of the species calling on the ground, the perch height was considered as present.

(ix) Distance to the nearest calling male: the nearest distance between two conspecific calling individuals.

(x) Number of recorded individuals: the number of individuals included in the description of the call.

(xi) Recorder: the specific device used to record the calls described in the study.

(xii) Software: the software used to analyze the calls described in the study.

(xiii) Microphone distance: the distance at which the microphone was positioned in relation to the recorded individual.

(xiv) Voucher specimen: the number of the collected specimen associated to the species or call description and deposited in a collection. It could be different from the individual that emitted the recorded calls used for the description.

(xv) Voucher recording: the number of the recorded calls that were described in the study and deposited in a sound collection.

(xvi) Sonogram (or spectrogram): a visual representation of spectrum of frequencies of the recorded call across the time.

(xvii) Oscilogram: a visual representation of the sound wave of the recorded call across the time.

(xviii) Power spectrum: a visual representation of a time series of a recorded call that describes the distribution of power into frequency components which composing that signal.

(xix) Call duration: the time between the onset and the end of a call. The call was considered as a set of sounds constituted by either a single note (simple call) or a series of identical or group of different notes (composite call) emitted in a defined period of time.

(xx) Note duration: the time between the onset and the end of a note. The note was considered as a temporally uninterrupted sound element composing the call and could be made up of a pulses series.

(xxi) Pulse number: the number of pulsed in a note. The pulse was considered as sounds of short duration produced by a single energy impulses released in the temporal spectrum of a note.

(xxii) Call rate: the number of calls emitted in a defined period of time.

(xxiii) Call frequency: the frequency band with more energy in the call.

(xxiv) Sound pressure level (SPL): the effective pressure caused by a sound wave relative to a reference pressure.

(xxv) Harmonics presence: the presence or absence of harmonics in the described call.
