# Supplementary material for: The advertisement calls of Brazilian anurans: Historical review, current knowledge and future directions
Source: PLoS One. 2018 Jan 30;13(1):e0191691. doi: 10.1371/journal.pone.0191691 (PMC5790252; doi:10.1371/journal.pone.0191691)
Supplement: S2 Text — (PDF) [file pone.0191691.s004.pdf]

S2 Text. Complete list of references cited in the S1 Table.

Abravaya, P.; Jackson, J. F. 1978. Reproduction in *Macrogenioglottus alipioi* Carvalho (Anura, Leptodactylidae). Contributions in Science, Natural History Museum of Los Angeles County 298: 1–9.

Abreu, R. O.; Juncá, F. A.; Souza, I. C. A.; Napoli, M. F. 2015. The tadpole of *Dendropsophus branneri* (Cochran, 1948) (Amphibia, Anura, Hylidae). Zootaxa 3946 (2): 296–300.

Abrunhosa, P.A.; Wogel, H. 2004. Breeding behavior of the leaf-frog *Phyllomedusa burmeisteri* (Anura: Hylidae). Amphibia-Reptilia 25: 125–135.

Abrunhosa, P.A.; Wogel, H.; Pombal, J.P.,Jr. 2001. Vocalização de quatro espécies de anuros do Estado do Rio de Janeiro, Sudeste do Brasil (Amphibia, Hylidae, Leptodactylidae). Boletim do Museu Nacional 472: 1–12.

Abrunhosa, P.A.; Pimenta, B.V.S.; Cruz, C.A.G.; Haddad, C.F.B. 2005. Advertisement Calls of Species of the *Hyla albosignata* Group (Amphibia, Anura, Hylidae). Arquivos do Museu Nacional 63: 275–282.

Acioli, E.C.S.; Toledo, L.F. 2008. Amphibia, Anura, Hylidae, *Hypsiboas beckeri*: filling gap and description of its advertisement call. Check List 4: 182–184.

Ahl, E. 1933. Über einige neu Frösche aus Brasilien. Zoologischer Anzeiger 104: 25–30.

Ahl, E. 1936. Zweif neue Froscharten der Gattung *Leptodactylus* aus Südamerika. Veröffentlichungen aus dem Deutschen Kolonial- und Übersee-Museum in Bremen 1: 389–392.

Aichinger, M. 1991. A new species of poison-dart frog (Anura: Dendrobatidae) from the Serranía de Sira, Peru. Herpetologica 47: 1–5.

Almeida, A. P.; Ângulo, A. 2006. A new species of *Leptodactylus* (Anura: Leptodactylidae) from the state of Espírito Santo, Brazil, with remarks on the systematics of associated populations. Zootaxa 1334: 1─25.

Alves, A. C. R.; Carvalho-e-Silva, S. P. 1999. Descrição da larva de *Scinax similis* (Cochran) com notas comparativas sobre o grupo "ruber" no sudeste do Brasil (Amphibia, Anura, Hylidae). Revista Brasileira de Zoologia 16(2): 507─512.

Alves, A. C. R.; Silva, S. 2002. Descriptions of the tadpoles of *Scinax alter* and *Scinax cuspidatus* (Anura: Hylidae). Journal of Herpetology 36: 133─137.

Alves, A. C. R.; Gomes, M. R.; Carvalho-e-Silva, S. P. 2004. Description of the tadpole of *Scinax auratus* (Weid-Neuwied) (Anura, Hylidae). Revista Brasileira de Zoologia 21: 315─317.

Alves, A. C. R.; Ribeiro, L. F.; Haddad, C. F. B.; Reis, S. F. dos. 2006. Two new species of *Brachycephalus* (Anura: Brachycephalidae) from the Atlantic Forest in Paraná state, southern Brazil. Herpetologica 62: 221–233.

Alves, A. C. R.; Sawaya, R. J.; Reis, S. F. dos; Haddad, C. F. B. 2009. New species of *Brachycephalus* (Anura: Brachycephalidae) from the Atlantic Rain Forest in São Paulo state, southeastern Brazil. Journal of Herpetology 43: 212–219.

Amézquita, A.; Lima, A. P.; Jehle, R.; Castellanos, L.; Ramos, O.; Crawford, A. J.; Gasser, H.; Hödl, W. 2009. Calls, colours, shape, and genes: a multi-trait approach to

the study of geographic variation in the Amazonian frog *Allobates femoralis*. Biological Journal of the Linnean Society 98: 826–838.

Andersson, L. G. 1911. A new *Leptodactylus* and a new Nototrema from Brazil. Arkiv för Zoologi. Stockholm 7(17): 1–6.

Andersson, L. G. 1914. A new *Telmatobius* and new teiidoid lizards from South America. Arkiv för Zoologi. Stockholm 9(3): 1–12.

Andersson, L. G. 1945. Batrachians from East Ecuador, collected 1937, 1938 by Wm. Clarke-Macintyre and Rolf Blomberg. Arkiv för Zoologi. Stockholm 37A(2): 1–88.

Andrade, G. V.; Cardoso, A. J. 1987. Reconhecimento do grupo rizibilis: descrição de uma nova espécie de *Hyla* (Amphibia, Anura). Revista Brasileira de Zoologia 3: 433─440.

Andrade, G. V.; Cardoso, A. J. 1991. Descrição de larvas e biologia de quatro espécies de *Hyla* (Amphibia, Anura). Revista Brasileira de Biologia 51: 391─402.

Andrade, F. S. de; Carvalho, T. R. de. 2013. A new species of *Pseudopaludicola* Miranda-Ribeiro (Leiuperinae: Leptodactylidae: Anura) from the Cerrado of southeastern Brazil. Zootaxa 3608: 389–397.

Andrade, F. S.; Haga, I. A.; Martins, F. A. M.; Giaretta, A. A. 2014. On advertisement call of the poison frog *Ameerega berohoka* (Dendrobatidae, Anura) from the Brazilian Cerrado. Zootaxa 3838: 392 396.

Andrade, S.P.de; Rocha, C. F.; Victor, E.P.,Jr.; Vaz-Silva, W. 2015. Advertisement call of *Rhinella inopina* Vaz-Silva, Valdujo & Pombal, 2012 (Anura: Bufonidae) from the type-locality, northeastern Goiás State, Central Brazil. Zootaxa 3931: 448–450.

66 Andrade, F. S. de; Magalhães, F. de M.; Nunes-de-Almeida, C. H. L.; Veiga-
67 Menoncello, A. C. P.; Santana, D. J.; Garda, A. A.; Loebmann, D.; Recco-Pimentel, ,
68 S.; Giaretta, , A. ; Toledo, L. F. 2016. A new species of long-legged *Pseudopaludicola*
69 from northeastern Brazil (Anura, Leptodactylidae, Leiuperinae). Salamandra 52: 107–
70 124.

71 Ângulo, A.; Icochea, J. 2003. *Adenomera* cf. *andreae* (NCN): vocalization.
72 Herpetological Review 34: 47–48.

73 Ângulo, A.; Cocroft, R.B.; Reichle, S. 2003. Species identity in the genus *Adenomera*
74 (Anura: Leptodactylidae) in southeastern Peru. Herpetologica 59: 490–504.

75 Antunes, A. P.; Haddad, C. F. B. 2009. Ferreiros da Mata Atlântica. Scientific
76 American Brasil 2009: 69–73.

77 Antunes, A. P.; Faivovich, J.; Haddad, C. F. B. 2008. A new species of *Hypsiboas* from
78 the Atlantic Forest of southeastern Brazil (Amphibia: Anura: Hylidae). Copeia 2008:
79 170–190.

80 Araújo, C. B; Guerra, T. J.; Amatuzzi, M. C. O.; Campos, L. A. 2012. Advertisement
81 and territorial calls of *Brachycephalus pitanga* (Anura: Brachycephalidae). Zootaxa
82 3302: 66–67.

83 Araújo Olívia G. S.; Haddad, C. F. B.; Silva, H. R. da; Pugener Lourdes, A. 2016. A
84 simplified table for staging embryos of the pipid frog *Pipa arrabali*. Anais da Academia
85 Brasileira de Ciências 88(3): 1875─1887.

86 Araujo-Vieira, K.; Brandão, R. A.; Faria, D. C. do C. 2015a. A new species of rock-
87 dwelling Scinax Wagler (Anura: Hylidae) from Chapada dos Veadeiros, central Brazil.
88 Zootaxa 3915: 52–66.

89 Araujo-Vieira, K.; Lacerda, J. V. A. de; Pezzuti, T. L.; Leite, F. S. F.; Assis, C. L. de;
90 Cruz, C. A. G. 2015b. A new species of Hatchet-faced Treefrog *Sphaenorhynchus*
91 Tschudi (Anura: Hylidae) from Quadrilátero Ferrífero, Minas Gerais, southeastern
92 Brazil. Zootaxa 4059: 96–114.

93 Araújo-Vieira, K.; Tacioli, A.; Faivovich, J.; Orrico, V. G. D.; Grant, T. 2015c. The
94 tadpole of *Sphaenorhynchus caramaschii*, with comments on larval morphology of
95 *Sphaenorhynchus* (Anura: Hylidae). Zootaxa 3904 (2): 270–282.

96 Araujo-Vieira, K.; Valdujo, P. H.; Faivovich, J. 2016. A new species of *Scinax* Wagler
97 (Anura: Hylidae) from Mato Grosso, Brazil. Zootaxa 4061: 261–273.

98 Assis, C. L. de; Santana, D. J.; Silva, F. A. da; Quintela, F. M.; Feio, R. N. 2013. A new
99 and possibly critically endangered species of casque-headed tree frog *Aparasphenodon*
100 Miranda-Ribeiro, 1920 (Anura, Hylidae) from southeastern Brazil . Zootaxa 3716: 583–
101 591.

102 Ávila, R. W.; Pansonato, A.; Strüssmann, C. 2010a. A new species of the *Rhinella*
103 *margaritifera* group (Anura: Bufonidae) from Brazilian Pantanal. Zootaxa 2339: 57–89.

104 Ávila, R. W.; Kawashita-Ribeiro, R. A.; Morais, D. H. 2011. A new species of
105 *Proceratophrys* (Anura: Cycloramphidae) from western Brazil. Zootaxa 2890: 20–28.

106 Ávila, R. W.; Carvalho, V. T. de; Gordo, M.; Kawashita-Ribeiro, R. A.; Morais, D. H.
107 2012a. A new species of *Amazophrynella* (Anura: Bufonidae) from southern Amazonia.
108 Zootaxa 3484: 65–74.

109 Ávila, R. W.; Pansonato, A.; Strüssmann, C. 2012b. A new species of *Proceratophrys*
110 (Anura: Cycloramphidae) from midwestern Brazil. Journal of Herpetology 46: 466–472.

111 Ayarzagüena, J. 1992. Los centrolenidos de la Guayana Venezolana. Publicaciones de
112 la Asociación de Amigos de Doñana. Sevilla 1: 1–46.

113 Baêta, D., Lourenço, A. C. C., Pezzuti, T. L. and Pires, M. R. S. 2007a. The tadpole,
114 advertisement call, and geographic distribution of *Physalaemus maximus* Feio, Pombal
115 and Caramaschi, 1999 (Amphibia, Anura, Leiuperidae). Arquivos do Museu Nacional
116 65:27-32.

117 Baêta, D.; Lourenco, A. C. C.; Nascimento, L. B. 2007b. Tadpole and advertisement
118 call of *Physalaemus erythros* Caramaschi, Feio and Guimaraes-Neto, 2003 (Amphibia,
119 Anura, Leiuperidae). Zootaxa 1623: 39─46.

120 Baêta, D.; Giasson, L. O. M.; Pombal, Jr., J. P.; Haddad, C. F. B. 2016. Review of the
121 rare genus *Phrynomedusa* Miranda-Ribeiro, 1923 (Anura: Phyllomedusidae) with
122 description of a new species. Herpetological Monographs 30: 49–78.

123 Baldissera, Jr., F. A.; Caramaschi, U.; Haddad, C. F. B. 2004. Review of the *Bufo*
124 *crucifer* species group, with descriptions of two new related species (Amphibia, Anura,
125 Bufonidae). Arquivos do Museu Nacional. Rio de Janeiro 62: 255–282.

126 Baldo, D.; Basso, N. G. 2004. A new species of *Melanophryniscus* Gallardo, 1961
127 (Anura: Bufonidae), with comments on the species of the genus reported for Misiones,
128 Northeastern Argentina. Journal of Herpetology 38: 393 403.

129 Baldo, D.; Maneyro, R.; Laufer, G. 2010. The tadpole of *Melanophryniscus atroluteus*
130 (Miranda Ribeiro, 1902) (Anura: Bufonidae) from Argentina and Uruguay. Zootaxa
131 2615: 66–68.

132 Bang, D. L.; Giaretta, A. A. 2016. Redescription of the advertisement calls of *Scinax*
133 *tigrinus* and *Scinax maracaya* (Anura: Hylidae) and an evaluation of their differential
134 diagnosis. Revista Brasileira de Biociências 14: 181─186.

135 Barata, I. M.; Santos, M. T. T.; Leite, F. S. F.; Garcia, P. C. de A. 2013. A new species
136 of *Crossodactylodes* (Anura: Leptodactylidae) from Minas Gerais, Brazil: first record of
137 genus within the Espinhaço Mountain Range . Zootaxa 3731: 552–560.

138 Barbour, T.; Dunn, E. R. 1921. Herpetological novelties. Proceedings of the Biological
139 Society of Washington 34: 157–162.

140 Barbour, T. 1909. Some new South American cold-blooded vertebrates. Proceedings of
141 the New England Zoölogical Club. Cambridge, Massachusetts 4: 47–52.

142 Barreto, G. S.; Ramos, J. C.; Mercês, E. A.; Napoli, M. F.; Garda, A. A.; Juncá, F. A.
143 2015. External morphology and oral cavity of the tadpole of *Trachycephalus atlas*
144 Bokermann, 1966 (Amphibia, Anura, Hylidae). Zootaxa 3980 (4): 597–600.

145 Barrio, A. 1945. Contribución al estudio de la etología y reproducción del batracio
146 *Pseudopaludicola falcipes*. Revista Argentina de Zoogeografía 5: 37–43.

147 Barrio, A. 1953. Sistemática, morfología y reproducción de *Physalaemus henselii*
148 (Peters) y *Pseudopaludicola falcipes* (Hensel) (Anura, Leptodactylidae). Physis 20:
149 379─389.

150 Barrio, A. 1962. Los Hylidae de Punta Lara, Provincia de Buenos Aires. Observaciones
151 sistemáticas, ecológicas y análisis espectrográfico del canto. Physis 23: 129–142.

152 Barrio, A. 1964a. Importancia, significación y análisis del canto de batracios anuros.
153 Publicaciones en Conmemoración del Cincuentenario del Museo Provincial de Ciencias
154 Naturales "Florentino Ameghino", Santa Fe: 51-79.

155 Barrio, A. 1964b. Relaciones morfológicas, eto-ecológicas y zoogeográficas entre
156 *Physalaemus henseli* (Peters) y *P. fernandezae* (Müller) (Anura, Leptodactylidae). Acta
157 Zoológica Lilloana 20: 284─305.

158 Barrio, A. 1965a. Afinidades del canto nupcial de las especies cavícolas del género
159 *Leptodactylus* (Anura-Leptodactylidae). Physis 25: 401–410.

160 Barrio, A. 1965b. El género *Physalaemus* (Anura, Leptodactylidae) en la Argentina.
161 Physis 25: 421 448.

162 Barrio, A. 1968. Revisión del género *Lepidobatrachus* Budgett (Anura,
163 Ceratophrynidae). Physis 28: 95–166.

164 Barrio, A. 1973. *Leptodactylus geminus* una nueva especie del grupo fuscus (Anura,
165 Leptodactylidae). Physis 32: 199–206.

166 Barrio, A. 1976. Estudio cariotipico y analisis audioespectrografico de los cantos de las
167 especies de *Phyllomedusa* (Anura, Hylidae) que habitan en la Argentina. Physis 35:
168 65 74.

169 Barrio, A. 1980. Una nueva especie de *Ceratophrys* (Anura: Ceratophryidae) del
170 Dominio Chaqueno. Physis. Buenos Aires 39: 21–30.

171 Barrio-Amorós, C. L.; Brewer-Carias, C. 2008. Herpetological results of the 2002
172 expedition to Sarisariñama, a tepui in Venezuelan Guayana, with the description of five
173 new species. Zootaxa 1942: 1–68.

174 Barrio-Amorós, C. L.; Díaz De A. P.; Mueses-Cisneros, J. J.; Infante, E.; Chacón, A.
175 2006. Hyla vigilans Solano, 1971, a second species for the genus *Scarthyla*,
176 redescription and distribution in Venezuela and Colombia. Zootaxa 1349: 1 18.

177 Barros, F. B.; Peloso, P. L. V.; Vicente, L.; Pereira, H. M.; Sturaro, M. J. 2010. The
178 advertisement call of the narrow-mouthed frog *Chiasmocleis avilapiresae* Peloso and
179 Sturaro, 2008 (Amphibia, Anura, Microhylidae). Zootaxa 2657: 66–68.

180 Bastos, R. P.; Haddad, C. F. B. 1995. Vocalizations and acoustic interactions in *Hyla*
181 *elegans* (Anura, Hylidae) during the reproductive activity. Naturalia 20: 165 176.

182 Bastos, R. P.; Haddad, C. F. B. 1999. Atividade reprodutiva de *Scinax rizibilis*
183 (Bokermann) (Anura, Hylidae) na Floresta Atlântica, sudeste do Brasil. Revista
184 Brasileira de Zoologia 16: 409 421.

185 Bastos, R. P.; Pombal, Jr., J. P. 1995. New species of *Crossodactylus* (Anura:
186 Leptodactylidae) from the Atlantic Rain Forest of southeastern Brazil. Copeia 1995:
187 436–439.

188 Bastos, R. P.; Pombal, Jr., J. P. 1996. A new species of *Hyla* (Anura: Hylidae) from
189 eastern Brazil. Amphibia-Reptilia 17: 325–331.

190 Bastos, R.P.; Bueno, M. A. F.; Dutra, S. L.; Lima, L. P. 2003. Padrões de vocalização
191 de anúncio em cinco espécies de Hylidae (Amphibia: Anura) do Brasil Central.
192 Comunicação do Museu de Ciências e Tecnologia da PUCRS 16: 39 51.

193 Bastos, R. P.; Alcantara, M. B. de; Morais, A. R.; Lingnau, R.; Signorelli, L. 2011a.
194 Vocal behavior and conspecific call response in *Scinax centralis* (Anura, Hylidae).
195 Herpetological Journal 21: 43 50.

196 Bastos, R. P.; Signorelli, L.; Morais, A. R.; Costa, T. B.; Lima, L. P.; Pombal, J. P.
197 2011b. Advertisement Calls of Three Anuran Species (Amphibia) from the Cerrado,
198 Central Brazil. South American Journal of Herpetology 6: 67 72.

199 Batallas, D. R.; Brito, J. M. 2014. Nueva especie de rana del genero *Pristimantis* del
200 grupo lacrimosus (Amphibia, Craugastoridae) del Parque Nacional Sangay, Ecuador.
201 Papéis Avulsos de Zoologia 54: 51 62.

202 Batista, V. G.; Gambale, P. G.; Lourenço-de-Moraes, R.; Campos, R. M.; Bastos, R. P.
203 2015. Vocalizations of two species of the *Hypsiboas pulchellus* group (Anura: Hylidae)
204 with comments on this species group. North-Western Journal of Zoology 253 261.

205 Batista, V. G.; Ramalho, W. P.; Amaral, D. F.; Maciel, N.; Bastos, R. P. 2016. The
206 advertisement and aggressive calls of *Rhinella abei* (Baldissera, Caramaschi, and
207 Haddad, 2004) (Anura: Bufonidae) from Campo Largo, Paraná, Brazil. Zootaxa 4107:
208 595─597.

209 Baumann, F. 1912. Brasilianische Batrachier des Berner Naturhistorischen Museums
210 nebst Untersuchenugen über die geographische Verbreitung der Batrachier in Brasilien.
211 Zoologische Jahrbücher. Abteilung für Systematik, Geographie und Biologie der Tiere.
212 Jena 33: 87–172.

213 Bee, M. A.; Gerhardt, H. C. 2001. Neighbour–stranger discrimination by territorial male
214 bullfrogs (*Rana catesbeiana*). I. Acoustic basis. Animal Behaviour 62: 1129–1140.

215 Beireis, G. C. 1783. Beschreibung eines bisher unbekannt gewesenen amerikanischen
216 Froschen, welcher sich in der Naturaliensammlung des Herrn Hofraths Beireis in
217 Helmstädt befindet. Schriften der Berlinischen Gesellschaft Naturforschender Freunde
218 4: 178–182.

219 Bell, T. 1843. Reptiles. Darwin, C. ed., The Zoology of the Voyage of the H.M.S.
220 Beagle, Under the Command of Captain Fitzroy, R.N., During the Years 1832 to 1836.
221 Volume 5: 1–51. London, Smith, Elder and Co..

222 Bernal, M. H.; Montealegre, D. P.; Páez, C. A. 2004. Estudio de la vocalización de trece
223 especies de anuros del municipio de Ibagué, Colombia. Revista de la Academia
224 Colombiana de Ciencias 28: 385–390.

225 Bernardes, C. S.; Carvalho, T. R.; Giaretta, A. A. 2015. Advertisement call of *Rhinella*
226 *major* (Anura: Bufonidae) from the lower Amazonas River basin with comments on
227 intraspecific variation. Zootaxa 4012: 375 378.

228 Bernardo-Silva, J. S.; Santos, R. R.; Both, C. 2010. The tadpole of *Melanophryniscus*
229 *cambaraensis* Braun & Braun, 1979 (Anura: Bufonidae). Zootaxa 2569: 67─68.

230 Berneck, B. v. M.; Costa, C. O. R. da; Garcia, P. C. de A. 2008. A new species of
231 *Leptodactylus* (Anura: Leptodactylidae) from the Atlantic forest of São Paulo state,
232 Brazil. Zootaxa 1795: 46–56.

233 Berneck, B. V. M.; Targino, M.; Garcia, P. C. de A. 2013. Rediscovery and re-
234 description of *Ischnocnema nigriventris* (Lutz, 1925) (Anura: Terrarana:
235 Brachycephalidae). Zootaxa 3692: 131–142.

236 Bertoluci, J.; Xavier, V.; Cassimiro, J. 2003. Description of the tadpole of *Hyla hylax*
237 Heyer, 1985 (Anura, Hylidae) with notes on its ecology. Amphibia-Reptilia 24:
238 509─514.

239 Bertoluci, J. A.; Leite, F. S.; Eisemberg, C. C.; Canelas, M. A. S. 2007. Description of
240 the tadpole of *Scinax luizotavioi* from the Atlantic rainforest of southeastern Brazil.
241 Herpetological Journal 17: 14─18.

242 Bevier, C. R.; Gomes, F. R.; Navas, C. A. 2008. Variation in call structure and calling
243 behavior in treefrogs of the genus *Scinax*. South American Journal of Herpetology 3:
244 196 206.

245 Bilate, M.; Wogel, H.; Weber, L. N.; Abrunhosa, P. A. 2006. Vocalizações e girino de
246 *Leptodactylus spixi* Heyer, 1983 (Amphibia, Anura, Leptodactylidae). Arquivos do
247 Museu Nacional 64: 235─245.

248 Bilate, M.; Nogueira-Costa, P.; Weber, L. N. 2012. The tadpole of the hylodid frog
249 *Hylodes ornatus* (Bokermann, 1967), including chondrocranium description, and
250 advertisement call. Zootaxa 3249: 60–66.

251 Bilate, M.; Lack, E. 2011. The advertisement call of *Scinax similis* (Cochran, 1952)
252 (Amphibia, Anura). South American Journal of Herpetology 6: 54 58.

253 Blotto, B. L.; Pereyra, M. O.; Baldo, D. 2014. The tadpole of *Rhinella azarai* (Gallardo,
254 1965) with comments on larval morphology in the *Rhinella granulosa* Species Group
255 (Anura: Bufonidae). Journal of Herpetology 48(3): 434─438.

256 Boddaert, P. 1772. Brief ... aan ... Johannes Oosterdyk Schacht naar het Leven
257 Vervaardige Afbeelding, van den Twee-koleurigen Kikvorsch, Uit des Vezameling van
258 ... Johannes Albertus Schlosser /Epistola ad ... Johannem Oosterdyk Schacht ... de Rana
259 bicolore, Descripta Atque Accuratissima Icone Illustrata Ex museo ... Johannis Alberti
260 Schlosseri. Amstelodami: M. Magérum.

261 Boettger, O. 1885. Liste von Reptilien und Batrachiern aus Paraguay. Zeitschrift für
262 Naturwissenschaften. Halle 58: 213–248.

263 Boistel, R.; Massary, J.-C. de. 1999. Les amphibiens vénéneux de la famille des
264 dendrobatidés. Le Courier de la Nature 176: 34–39.

265 Boistel, R.; Massary, J.-C. de; Ângulo, A. 2006. Description of a new species of the
266 genus *Adenomera* (Amphibia, Anura, Leptodactylidae) from French Guiana. Acta
267 Herpetologica 1: 1–14.

268 Bokermann, W. C. A.; Sazima, I. 1973a. Anfíbios da Serra do Cipó, Minas Gerais,
269 Brasil. I—Espécies novas de *Hyla* (Anura, Hylidae). Revista Brasileira de Biologia 33:
270 329–336.

271 Bokermann, W. C. A.; Sazima, I. 1973b. Anfíbios da Serra do Cipó, Minas Gerais,
272 Brasil. II— Duas espécies novas de *Hyla* (Anura, Hylidae). Revista Brasileira de
273 Biologia 33: 521–528.

Bokermann, W. C. A.; Sazima, I. 1978. Anfíbios da Serra do Cipó, Minas Gerais, Brasil. 4: Descrição de *Phyllomedusa jandaia* sp. n. (Anura, Hylidae). Revista Brasileira de Biologia 38: 927–930.

Bokermann, W. C. A. 1952. Microhylidae de coleçao do Departamento de Zoologia (Amphibia-Anura). Papeis Avulsos de Zoologia. São Paulo 10: 271–292.

Bokermann, W. C. A. 1956a. Sôbre uma espécie de *Hyla* do estado de Minas Gerais, Brasil. Papeis Avulsos de Zoologia. São Paulo 12: 357–362.

Bokermann, W. C. A. 1956b. Sôbre una nueva especie de *Leptodactylus* del Brasil (Amphibia). Neotropica. La Plata 2: 37–40.

Bokermann, W. C. A. 1956c. Una nueva especies del genero *Elosia* del sudeste del Brasil (Amphibia Salientia, Leptodactylidae). Neotropica. La Plata 2: 81–84.

Bokermann, W. C. A. 1957a. Notas sobre a biologia de *Leptodactylus flavopictus* Lutz, 1926. Revista Brasileira de Biologia 17: 495─500.

Bokermann, W. C. A. 1957b. Sôbre uma nova espécie de *Cycloramphus* do Estado de Santa Catarina, Brasil. Revista Brasileira de Biologia 17: 249–252.

Bokermann, W. C. A. 1959. Una nueva especies de *Leptodactylus* de la region Amazonica (Amphibia, Salientia, Leptodactylidae). Neotropica. La Plata 5: 5–8.

Bokermann, W. C. A. 1962a. Cuatro nuevos hylidos del Brasil. Neotropica. La Plata 8: 81–92.

Bokermann, W. C. A. 1962b. Observações biológicas sobre *Physalaemus cuvieri* Fitz., 1826 (Amphibia, Salientia). Revista Brasileira de Biologia 22: 391─399.

295 Bokermann, W. C. A. 1962c. Nova especie de *Hyla* de Rondônia, Brasil (Amphibia,
296 Salientia). Atas da Sociedade de Biologia do Rio de Janeiro 6: 52–55.

297 Bokermann, W. C. A. 1962d. Sôbre uma pequena coleção de anfibios do Brasil Central,
298 com de descrição de una especie nova de *Physalaemus* (Amphibia, Salientia). Revista
299 Brasileira de Biologia 22: 213–219.

300 Bokermann, W. C. A. 1962e. Una nueva especies de *Atelopus* del nordeste de Brasil
301 (Amphibia, Salientia, Brachycephalidae). Neotropica. La Plata 8: 42–44.

302 Bokermann, W. C. A. 1963a. Duas espécies novas de *Hyla* de Rondônia, Brasil. Revista
303 Brasileira de Biologia 23: 247–250.

304 Bokermann, W. C. A. 1963b. Girinos de anfíbios brasileiros - I (Amphibia, Salientia).
305 Anais da Academis Brasileira de Ciências 35: 465─474.

306 Bokermann, W. C. A. 1963c. Girinos de anfíbios brasileiros - 2 (Amphibia, Salientia).
307 Revista Brasileira de Biologia 23: 349─353.

308 Bokermann, W. C. A. 1963d. Nova especie de *Hyla* da Bahia, Brasil. Atas da Sociedade
309 de Biologia do Rio de Janeiro 7: 6–8.

310 Bokermann, W. C. A. 1963e. Una nueva especie de *Hyla* del sudeste Brasileño.
311 Neotropica. La Plata 9: 27–30.

312 Bokermann, W. C. A. 1964a. Dos nuevas especies de Hyla de Minas Gerais y notas
313 sobre *Hyla alvarengai* Bok. (Amphibia, Salientia, Hylidae). Neotropica. La Plata 10:
314 67–76.

315 Bokermann, W. C. A. 1964b. Dos nuevas especies de *Hyla* de Rondonia, Brasil
316 (Amphibia, Salientia, Hylidae). Neotropica. La Plata 10: 3–6.

317 Bokermann, W. C. A. 1964c. Una nueva especie de *Elosia* de la Serra de Mantiqueira,
318 Brasil. Neotropica 10: 102–107.

319 Bokermann, W. C. A. 1964d. Uma nova especie de *Hyla* da Serra do Mar em São Paulo
320 (Amphibia, Salientia). Revista Brasileira de Biologia 24: 429–434.

321 Bokermann, W. C. A. 1964e. Notes on treefrogs of the *Hyla marmorata* group with
322 description of a new species (Amphibia, Hylidae). Senckenbergiana Biologica 45: 243–
323 254.

324 Bokermann, W. C. A. 1965a. A new *Eleutherodactylus* from southeastern Brazil.
325 Copeia 1965: 440–441.

326 Bokermann, W. C. A. 1965b. *Hyla langei*, a new frog from Paraná, southern Brasil.
327 Journal of the Ohio Herpetological Society 5: 49–51.

328 Bokermann, W. C. A. 1965c. Notas sobre as espécies de *Thoropa* Fitzinger (Amphibia,
329 Anura). Anais Academia Brasileira de Ciências 37: 525─537.

330 Bokermann, W. C. A. 1965d. Tres novos batraquios da região central de Mato Grosso,
331 Brasil. Revista Brasileira de Biologia 25: 257–264.

332 Bokermann, W. C. A. 1966a. A new *Phyllomedusa* from southeastern Brasil.
333 Herpetologica 22: 293─297.

334 Bokermann, W. C. A. 1966b. Dos nuevas especies de *Physalameus* de Espirito Santo,
335 Brasil (Amphibia, Leptodactylidae). Physis 26: 193–202.

336 Bokermann, W. C. A. 1966c. Dos nuevas especies de *Sphaenorhynchus* (Amphibia,
337 Hylidae). Revista Brasileira de Biologia 26: 15–21.

Bokermann, W. C. A. 1966d. Notas sobre Hylidae do Espírito Santo (Amphibia, Salientia). Revista Brasileira de Biologia 26: 29─37.

Bokermann, W. C. A. 1966e. Notas sobre três espécies de *Physalaemus* de Maracas, Bahia (Amphibia, Leptodactylidae). Revista Brasileira de Biologia 26: 253–259.

Bokermann, W. C. A. 1966f. Lista Anotada das Localidades Tipo de Anfíbios Brasileiros. São Paulo: Servicio de Documentacão, Universidade Rural São Paulo.

Bokermann, W. C. A. 1966g. O gênero *Phyllodytes* Wagler, 1830 (Anura, Hylidae). Anais da Academia Brasileira de Ciências 38(2): 335─344.

Bokermann, W. C. A. 1966h. Una nueva especie de *Trachycephalus* de Bahia, Brasil (Amphibia, Hylidae). Neotropica. La Plata 12: 120–124.

Bokermann, W. C. A. 1967a. Dos nuevas especies de *Hyla* del grupo catharinae. Neotropica. La Plata 13: 61–66.

Bokermann, W. C. A. 1967b. Girinos de anfíbios brasileiros - 3. Sobre um girino gigante de *Pseudis paradoxa* (Amphibia, Pseudidae). Revista Brasileira de Biologia 27: 208─212.

Bokermann, W. C. A. 1967c. Girinos de anfíbios brasileiros - 4. Revista Brasileira de Biologia 27: 363─367.

Bokermann, W. C. A. 1967d. *Hyla astartea*, nova espécie da Serra do Mar em São Paul (Amphibia, Hylidae). Revista Brasileira de Biologia 27: 157–158.

Bokermann, W. C. A. 1967e. Notas sôbre a distribuição de *Bufo granulosus* Spix, 1824 na Amazônia e descrição de uma subespécie nova (Amphibia, Bufonidae). Lent, H. ed.,

359 Atas do Simposio Sôbre a Biota Amazônica, Volume 5 (Zoologia): 103–109. Rio de
360 Janeiro, Brazil, Conselho Nacional de Pesquisas.

361 Bokermann, W. C. A. 1967f. Notas sobre cantos nupciais de anfíbios brasileiros: I.
362 (Anura). Anais da Academia Brasileira de Ciências 39: 438 443.

363 Bokermann, W. C. A. 1967g. Notas sobre cantos nupciais de anfíbios brasileiros
364 (Anura). III. Anais da Academia Brasileira de Ciências 39: 491–493.

365 Bokermann, W. C. A. 1967h. Notas sobre *Hyla duartei* B. Lutz (Anura, Hylidae). Anais
366 da Academia Brasileira de Ciências 39: 437─440.

367 Bokermann, W. C. A. 1967i. Nova espéce de *Hyla* do Amapá (Amphibia, Hylidae).
368 Revista Brasileira de Biologia 27: 109–112.

369 Bokermann, W. C. A. 1967j. Observações sobre *Melanophryniscus moreirae* (Mir.
370 Rib.) (Amphibia - Brachycephalidae). Anais da Academis Brasileira de Ciências 39:
371 301─306.

372 Bokermann, W. C. A. 1967k. Três novas especies de *Physalaemus* do sudeste brasileiro
373 (Amphibia, Leptodactylidae). Revista Brasileira de Biologia 27: 135–143.

374 Bokermann, W. C. A. 1967l. Una nueva especie de *Eleutherodactylus* del sudeste
375 brasileño (Amphibia, Leptodactylidae). Neotropica. La Plata 13: 1–3.

376 Bokermann, W. C. A. 1967m. Una nueva especie de *Elosia* de Itatiaia, Brasil
377 (Amphibia, Leptodactylidae). Neotropica. La Plata 13: 135–137.

378 Bokermann, W. C. A. 1968a. Observações sobre *Hyla pardalis* Spix (Anura, Hylidae).
379 Revista Brasileira de Biologia 28: 1─6.

Bokermann, W. C. A. 1968b. Three new *Hyla* from the Plateau of Maracás, central Bahia, Brazil. Journal of Herpetology 1: 25–31.

Bokermann, W. C. A. 1969. Uma nova espécie de *Leptodactylus* de Mato Grosso (Anura, Leptodactylidae). Revista Brasileira de Biologia 29: 3–16.

Bokermann, W. C. A. 1972a. Notas sobre *Hyla clepsydra* A. Lutz (Anura, Hylidae). Revista Brasileira de Biologia 32: 291─295.

Bokermann, W. C. A. 1972b. Uma nova espécie de *Hyla* de Goiás, Brasil (Anura, Hylidae). Revista Brasileira de Biologia 32: 593–594.

Bokermann, W. C. A. 1973. Duas novas especies de *Sphaenorynchus* da Bahia (Anura, Hylidae). Revista Brasileira de Biologia 33: 589–594.

Bokermann, W. C. A. 1974. Três espécies novas de *Eleutherodactylus* do sudeste da Bahia, Brasil (Anura, Leptodactylidae). Revista Brasileira de Biologia 34: 11–18.

Bokermann, W. C. A. 1975. Uma nova espécie de *Colostethus* do Brasil Central (Anura, Dendrobatidae). Iheringia 46: 13–18.

Bokermann, W. C. A. 1978. Anfíbios da Serra do Cipó, Minas Gerais, Brasil. 4. Descrição de *Phyllomedusa jandaia* sp.n. (Anura, Hylidae). Revista Brasileira de Biologia 38: 927─930.

Bornschein, M. R.; Firkowski, C. R.; Baldo, D.; Ribeiro, L. F.; Belmonte-Lopes, R.; Corrêa, L.; Morato, S. A. A.; Pie, M. R. 2015. Three new species of phytotelm-breeding *Melanophryniscus* from the Atlantic rainforest of southern Brazil (Anura: Bufonidae). PLoS (Public Library of Science) One 10 (12): e0142791: 1–35.

401 Bornschein, M. R., L. F. Ribeiro, D. C. Blackburn, E. L. Stanley, and M. R. Pie. 2016.
402 A new species of *Brachycephalus* (Anura: Brachycephalidae) from Santa Catarina,
403 southern Brazil. PeerJ : 1–19.

404 Borteiro, C.; Kolenc, F. 2007. Redescription of the tadpoles of three species of frogs
405 from Uruguay (Amphibia: Anura: Leiuperidae and Leptodactylidae), with notes on
406 natural history. Zootaxa 1638: 1–20.

407 Borteiro, C.; Kolenc, F.;Tedros, M.; Prigioni, C. 2006. The tadpole of *Chaunus*
408 *dorbignyi* (Duméril and Bibron) (Anura, Bufonidae). Zootaxa 1308: 49–62.

409 Borteiro, C.; Kolenc, F.; Pereyra, M. O.; Rosset, S. D.; Baldo, D. 2010. A diploid
410 surrounded by polyploids: tadpole description, natural history and cytogenetics of
411 *Odontophrynus maisuma* Rosset from Uruguay (Anura: Cycloramphidae). Zootaxa
412 2611: 1–15.

413 Bosch, J.; De la Riva, I.; Márquez, R. 1996. The calling behavior of *Lysapsus limellus*
414 and *Pseudis paradoxa* (Amphibia:Anura:Pseudidae). Folia Zoologica 45: 49 55.

415 Both, C. A.; Kwet, A.; Solé, M. 2006. The tadpole of *Physalaemus lisei* Braun and
416 Braun, 1977 (Anura, Leptodactylidae) from southern Brazil. Alytes 24: 109─116.

417 Both, C.; Kwet, A.; Solé, M. 2007. The tadpole of *Hypsiboas leptolineatus* (Braun and
418 Braun, 1977), a species in the *Hypsiboas polytaenius* clade (Anura; Hylidae). Brazilian
419 Journal of Biology 67(2): 309─312.

420 Boulenger, G. A. 1882. Catalogue of the Batrachia Salientia s. Ecaudata in the
421 Collection of the British Museum. Second Edition. London: Taylor and Francis.

422 Boulenger, G. A. 1883. Notes on little-known species of frogs. Annals and Magazine of
423 Natural History, Series 5, 11: 16–19.

424 Boulenger, G. A. 1884. On a collection of frogs from Yurimaguas, Huallaga River,
425 Northern Peru. Proceedings of the Zoological Society of London 1883: 635–638.

426 Boulenger, G. A. 1885. Second list of reptiles and batrachians from the Province Rio
427 Grande do Sul, Brazil, sent to the Natural History Museum by Dr. H. von Ihering.
428 Annals and Magazine of Natural History, Series 5, 16: 85–88.

429 Boulenger, G. A. 1887. Descriptions of new or little-known South-American frogs of
430 the genera *Paludicola* and *Hyla*. Annals and Magazine of Natural History, Series 5, 20:
431 295–300.

432 Boulenger, G. A. 1888a. A list of batrachians from the Province Santa Catharina, Brazil.
433 Annals and Magazine of Natural History, Series 6, 1: 415–417.

434 Boulenger, G. A. 1888b. Descriptions of new Brazilian batrachians. Annals and
435 Magazine of Natural History, Series 6, 1: 187–189.

436 Boulenger, G. A. 1888c. On some reptiles and batrachians from Iguarasse, Pernambuco.
437 Annals and Magazine of Natural History, Series 6, 2: 40–43.

438 Boulenger, G. A. 1889. On a collection of batrachians made by Prof. Charles
439 Spegazzini at Colonia Resistencia, South Chaco, Argentine Republic. Annali del Museo
440 Civico di Storia Naturale di Genova. Serie 2, 7: 246–249.

441 Boulenger, G. A. 1894. List of reptiles and batrachians collected by Dr. J. Bohls near
442 Asuncion, Paraguay. Annals and Magazine of Natural History, Series 6, 13: 342–348.

443 Boulenger, G. A. 1895a. Third report on additions to the batrachian collection in the
444 Natural-History Museum. Proceedings of the Zoological Society of London 1894: 640–
445 646.

446 Boulenger, G. A. 1895b. Correction to p. 521 ('Annals,' June 1895). Annals and
447 Magazine of Natural History, Series 6, 16: 125.

448 Boulenger, G. A. 1896. Descriptions of new batrachians in the British Museum. Annals
449 and Magazine of Natural History, Series 6, 17: 401–406.

450 Boulenger, G. A. 1898. A list of the reptiles and batrachians collected by the late Prof.
451 L. Balzan in Bolivia. Annali del Museo Civico di Storia Naturale di Genova. Serie 2,
452 19: 128–133.

453 Boulenger, G. A. 1900. Batrachians. In E. R. Lankester, Report on a collection made by
454 Messrs. F. V. McConnell and J. J. Quelch at Mount Roraima in British Guiana.
455 Transactions of the Linnean Society of London. 2nd series, Zoology 8: 55–56.

456 Boulenger, G. A. 1902. Descriptions of new batrachians and reptiles from the Andes of
457 Peru and Bolivia. Annals and Magazine of Natural History, Series 7, 10: 394–402.

458 Boulenger, G. A. 1905. Descriptions of new tailless batrachians in the collection of the
459 British Museum. Annals and Magazine of Natural History, Series 7, 16: 180–184.

460 Boulenger, G. A. 1912. Descriptions of new batrachians from the Andes of South
461 America, preserved in the British Museum. Annals and Magazine of Natural History,
462 Series 8, 10: 185–191.

463 Boulenger, G. A. 1918. Descriptions of new South American batrachians. Annals and
464 Magazine of Natural History, Series 9, 2: 427–433.

Brandão, R. A.; Álvares, G. F. 2009. Remarks on "A new *Phyllomedusa* Wagler (Anura, Hylidae) with reticulated pattern on flanks from Southeastern Brazil". Zootaxa 2044: 61 64.

Brandão, R. A.; Batista, C. G. 2000. Descrição do girino de *Odontophrynus salvatori* (Anura, Leptodactylidae). Iheríngia 89: 165─170.

Brandão, R. A.; Heyer, W. R. 2005. The complex calls of *Leptodactylus pustulatus* (Amphibia, Anura, Leptodactylidae).Amphibia-Reptilia 26: 566 570.

Brandão, R. A. 2002. A new species of *Phyllomedusa* Wagler, 1830 (Anura: Hylidae) from central Brazil. Journal of Herpetology 36: 571–578.

Brandão, R. A.; Maciel, N. M.; Sebben, A. 2007. A new species of *Chaunus* from central Brazil (Anura: Bufonidae). Journal of Herpetology 41: 309–316.

Brandão, R. A.; Álvares, G. F. R.; Crema, A.; Zernini, G. J. 2009. Natural history of *Phyllomedusa centralis* Bokermann 1965 (Anura: Hylidae: Phyllomedusinae): tadpole and calls. South American Journal of Herpetology 4: 61─68.

Brandão, R. A.; Magalhães, R. F. de; Garda, A. A.; Campos, L. A.; Sebben, A.; Maciel, A. O. 2012. A new species of *Bokermannohyla* (Anura: Hylidae) from highlands of central Brazil. Zootaxa 3527: 28–42.

Brandão, R. A.; Álvares, G. F. R.; De Sá, R. O. 2013. The advertisement call of the poorly known *Leptodactylus tapiti* (Anura, Leptodactylidae). Zootaxa 3616: 284 286.

Brandão, R. A.; Caramaschi, U.; Vaz-Silva, W.; Campos, L. A. 2013. Three new species of *Proceratophrys* Miranda-Ribeiro 1920 from Brazilian Cerrado (Anura, Odontophrynidae) . Zootaxa 3750: 321–347.

487 Brasileiro, C. A.; Haddad, C. F. B. 2015. A new species of *Physalaemus* from central
488 Brazil (Anura: Leptodactylidae). Herpetologica 71: 280–288.

489 Brasileiro, C. A.; Haddad, C. F. B.; Sawaya, R. J.; Martins, M. 2007a. A new and
490 threatened species of *Scinax* (Anura: Hylidae) from Queimada Grande Island,
491 southeastern Brazil. Zootaxa 1391: 47–55.

492 Brasileiro, C. A.; Haddad, C. F. B.; Sawaya, R. J.; Sazima, I. 2007b. A new and
493 threatened island-dwelling species of *Cycloramphus* (Anura: Cycloramphidae) from
494 southeastern Brazil. Herpetologica 63: 501–510.

495 Brasileiro, C. A.; Oyamaguchi, H. M.; Haddad, C. F. B. 2007c. A new island species of
496 *Scinax* (Anura; Hylidae) from southeastern Brazil. Journal of Herpetology 41: 271–275.

497 Brasileiro, C. A.; Martins, I. A.; Jim, J. 2008. Amphibia, Anura, Cycloramphidae,
498 *Odontophrynus moratoi*: Distribution extension and advertisement call. Check List 4:
499 382–385.

500 Braun, P. C.; Braun, C. A. S. 1977a. Nova espécie de *Hyla* do estado do Rio Grande do
501 Sul, Brasil (Anura, Hylidae). Revista Brasileira de Biologia 37: 853–857.

502 Braun, P. C.; Braun, C. A. S. 1977b. Nova espécie de *Physalaemus* do estado do Rio
503 Grande do Sul, Brasil (Anura, Leptodactylidae). Revista Brasileira de Biologia 37: 867–
504 871.

505 Braun, P. C.; Braun, C. A. S. 1979. Nova espécie de *Melanophryniscus* Gallardo, 1961
506 do Estado do Rio Grande do Sul, Brasil (Anura, Bufonidae). Iheringia. Série Zoologia
507 54: 7–17.

508 Braun, P. C. 1973. Nova espécie do genero *Melanophryniscus* Gallardo, 1961 do Estado
509 do Rio Grande do Sul, Brasil (Anura, Brachycephalidae). Iheringia. Série Zoologia 44:
510 3–13.

511 Breder Jr., C. M. 1946. Amphibians and reptiles of the Rio Chucubaque drainage,
512 Darien, Panama, with notes on their life histories and habitats. Bulletin of the American
513 Museum of Natural History 86: 381─435.

514 Brongersma, L. D. 1933. Ein neuer Laubfrosch aus Surinam. Zoologischer Anzeiger
515 103: 267–270.

516 Brown, J. L.; Schulte, R.; Summers, K. 2006. A new species of *Dendrobates* (Anura:
517 Dendrobatidae) from the Amazonian lowlands of Perú. Zootaxa 1152: 45–58.

518 Brown, J. L.; Twomey, E.; Amezquita, A.; Barbosa-de-Souza, M.; Caldwell, J. P.;
519 Lötters, S.; von May, R.; Melo-Sampaio, P. R.; Mejia-Vargas, D.; Perez-Peña, P.;
520 Pepper, M.; Poelman, E.H.; Sanchez-Rodriguez, M.; Summers, K. 2011. A taxonomic
521 revision of the Neotropical poison frog genus *Ranitomeya* (Amphibia: Dendrobatidae).
522 Zootaxa 3083: 1 120.

523 Brunetti, A. E.; Taboada, C.; Faivovich, J. 2015. Extended vocal repertoire in
524 *Hypsiboas punctatus* (Anura: Hylidae). Journal of Herpetology 49: 46–52.

525 Bruschi, D. P.; Lucas, E. M.; Garcia, P. C. de A.; Recco-Pimentel, S. M. 2014.
526 Molecular and morphological evidence reveals a new species in the *Phyllomedusa*
527 *hypochondrialis* group (Hylidae, Phyllomedusinae) from the Atlantic Forest of the
528 highlands of southern Brazil. Public Library of Science (PLoS) One 9 (8: e105608): 1–
529 13.

530 Brusquetti, F.; Thomé, M. T. C.; Canedo, C.; Condez, T. H.; Haddad, C. F. B. 2013. A
531 new species of *Ischnocnema parva* species series (Anura, Brachycephalidae) from
532 northern state of Rio de Janeiro, Brazil. Herpetologica 69: 175–185.

533 Brusquetti, F.; Jansen, M.; Barrio-Amorós, C. L.; Segalla, M. V.; Haddad, C. F. B.
534 2014. Taxonomic review of *Scinax fuscomarginatus* (Lutz, 1925) and related species
535 (Anura; Hylidae). Zoological Journal of the Linnean Society 171: 783–821.

536 Budgett, J. S. 1899. Notes on the batrachians of Paraguayan Chaco, with observations
537 upon their breeding habits and development, especially with regard to *Phyllomedusa*
538 *hypochondrialis* Cope. Also a description of a new genus. Quarterly Journal of
539 Microscopical Science. London 42: 305–333.

540 Bünten, G.; Heuwinkel, H.; Greven, H. 1992. Zur Hydroakustik von *Pipa carvalhoi*
541 (Miranda-Ribeiro, 1937). Salamandra 28: 72 85.

542 Burmeister, H. 1856. Erläuterungen zur Fauna Brasiliens, enthaltend Abbildungen und
543 ausführliche Beschreibungen neuer oder ungenügend bekannter Thier-Arten. Berlin:
544 Georg Reimer.

545 Burmeister, H. 1861. Reise durch die La Plata-Staaten mit besonderer Rücksicht auf die
546 Physische Beschaffenheit und den Culturzustand der Argentinische Republik.
547 Ausgefuhrt in den Jahren 1857, 1858, 1859 un 1860. Volume 2. Halle: H. W. Schmidt.

548 Caldart, V. M.; Iop, S.; Cechin, S. Z. 2011. Vocalizations of *Crossodactylus schmidti*
549 Gallardo, 1961 (Anura, Hylodidae): advertisement call and aggressive call. North-
550 Western Journal of Zoology 7: 118-124.

551 Caldart, A. M.; Santos, T. G.; Maneyro, R. 2013. The advertisement and release calls of
552 *Melanophryniscus pachyrhynus* (Miranda-Ribeiro, 1920) from the central region of Rio
553 Grande do Sul, southern Brazil. Acta Herpetologica 8: 115 122.

554 Caldart, V. M.; Iop, S.; Lingnau, R.; Cechin, S. Z. 2016. Communication in a noisy
555 environment: short-term acoustic adjustments and the underlying acoustic niche of a
556 Neotropical stream-breeding frog. Acta Ethologica 19: 151.

557 Caldwell, J. P.; Hoogmoed, M. S. 1998. Allophrynidae, Allophryne, A. ruthveni.
558 Catalogue of American Amphibians and Reptiles 666: 1 3.

559 Caldwell, J. P.; Lima, A. P. 2003. A new Amazonian species of *Colostethus* (Anura:
560 Dendrobatidae) with a nidicolous tadpole. Herpetologica 59: 219–234.

561 Caldwell, J. P.; Myers, C. W. 1990. A new poison frog from Amazonian Brazil, with
562 further revision of the quinquevittatus group of *Dendrobates*. American Museum
563 Novitates 2988: 1–21.

564 Caldwell, J. P.; Shepard, D. B. 2007. Calling site fidelity and call structure of a
565 neotropical toad, *Rhinella ocellata* (Anura: Bufonidae). Journal of Herpetology 41:
566 611 621.

567 Caldwell, J. P. 1991. A new species of toad in the genus *Bufo* from Para, Brazil, with an
568 unusual breeding site. Papeis Avulsos de Zoologia. São Paulo 37: 389–400.

569 Caldwell, J. P. 2005. A new amazonian species of *Cryptophyllobates* (Anura:
570 Dendrobatidae). Herpetologica 61: 449─461.

571 Caldwell, J. P.; Lima, A. P.; Biavati, G. M. 2002a. Descriptions of the tadpoles of
572 *Colostethus marchesianus* and *Colostethus caeruleodactylus* (Anura: Dendrobatidae)
573 from their type localities. Copeia 2002: 166─172.

574 Caldwell, J. P.; Lima, A. P.; Keller, C. 2002b. Redescription of *Colostethus*
575 *marchesianus* (Melin, 1941) from its type locality. Copeia 2002: 157–165.

576 Caminer, M. A.; Ron, S. R. 2014. Systematics of treefrogs of the *Hypsiboas calcaratus*
577 and *Hypsiboas fasciatus* species complex (Anura, Hylidae) with the description of four
578 new species. ZooKeys 370: 1–68.

579 Campbell, J. A.; Clarke, B. T. 1998. A review of frogs of the genus *Otophryne*
580 (Microhylidae) with the description of a new species. Herpetologica 54: 301–317.

581 Campos, T. F.; Lima, M. G. de; Nascimento, A. C. do; Santos, E. M. dos. 2014. Larval
582 morphology and advertisement call of *Phyllodytes acuminatus* Bokermann, 1966
583 (Anura: Hylidae) from Northeastern Brazil . Zootaxa 3779: 93–100.

584 Camurugi, F.; Juncá, F. A. 2013. Reproductive biology of *Hypsiboas atlanticus* (Anura:
585 Hylidae). Herpetology Notes 6: 489 495.

586 Camurugi, F.; Mercês, E. A.; Nunes, I.; Juncá, F. A. 2013. The tadpole of *Scinax*
587 *strigilatus* (Spix, 1824) (Anura: Hylidae). Zootaxa 3686 (4): 497–499.

588 Camurugi, F.; Röhr, D. L.; Juncá, F. A. 2015. Differences in advertisement calls and
589 vocal Behavior in *Hypsiboas atlanticus* (Anura: Hylidae) among Microhabitats.
590 Herpetologica 71: 243 251.

591 Candioti, M. F. V. 2005. Morphology and feeding in tadpoles of *Ceratophrys cranwelli*
592 (Anura: Leptodactylidae). ActaZoologica (Stockholm) 86: 1─11.

593 Candioti, M. F. V., Brusquetti, F. and Netto, F. 2007. Morphological characterization of
594 *Leptodactylus elenae* tadpoles (Anura: Leptodactylidae: *L. fuscus* group), from central
595 Paraguay. Zootaxa 1435: 1─17.

596 Canedo, C.; Pimenta, B. V. S. 2010. New species of *Ischnocnema* (Anura,
597 Brachycephalidae) from the Atlantic rainforest of the state of Espérito Santo, Brazil.
598 South American Journal of Herpetology 5: 199–206.

599 Canedo, C.; Pombal, Jr., J. P. 2007. Two new species of torrent frog of the genus
600 *Hylodes* (Anura, Hylodidae) with nuptial thumb tubercles. Herpetologica 63: 224–235.

601 Canedo, C.; Dixo, M.; Pombal, Jr., J. P. 2004. A new species of *Chiasmocleis* Mehely,
602 1904 (Anura, Microhylidae) from the Atlantic rainforest of Bahia, Brazil. Herpetologica
603 60: 495–501.

604 Canedo, C.; Pimenta, B. V. S.; Leite, F. S. F.; Caramaschi, U. 2010. New species of
605 *Ischnocnema* (Anura: Brachycephalidae) from the state of Minas Gerais, southeastern
606 Brazil, with comments on the *I. verrucosa* species series. Copeia 2010: 629–634.

607 Canedo, C.; Targino, M.; Leite, F. S. F.; Haddad, C. F. B. 2012. A new species of
608 *Ischnocnema* (Anura) from the São Francisco Basin karst region, Brazil. Herpetologica
609 68: 393–400.

610 Cannatella, D. C. 1983. Synonymy and distribution of *Phyllomedusa boliviana*
611 Boulenger (Anura: Hylidae). Proceedings of the Biological Society of Washington 96:
612 59–66.

613 Capranica, R. R. 1965. The evoked vocal response of the Bullfrog: a study of
614 communication by sound (MIT Press, Cambridge, MA).

615 Caram, J.; Luna-Dias, C.; Hepp, F. S. F. dos S., Neto; Carvalho-e-Silva, S. P. de. 2014.
616 The advertisement call of *Dendropsophus pseudomeridianus* (Cruz, Caramaschi &
617 Dias) (Anura: Hylidae) . Zootaxa 3784: 294–296.

618 Caramaschi, U.; Cruz, C. A. G. 1997. Redescription of *Chiasmocleis albopunctata*
619 (Boettger) and description of a new species of *Chiasmocleis* (Anura: Microhylidae).
620 Herpetologica 53: 259–268.

621 Caramaschi, U.; Cruz, C. A. G. 1998. Notas taxonômicas sobre *Pseudis fusca* Garman e
622 *P. bolboactyla* A. Lutz, com a descriçao de uma nova espécie correlata (Anura,
623 Pseudidae). Revista Brasileira de Biologia 15(4): 929─944.

624 Caramaschi, U.; Cruz, C. A. G. 1999. Duas espécies novas do grupo de *Hyla polytaenia*
625 Cope, 1870 do Estado de Minas Gerais, Brasil. Boletim do Museu Nacional. Nova
626 Serie, Zoologia. Rio de Janeiro 403: 1–10.

627 Caramaschi, U.; Cruz, C. A. G. 2000. Duas espécies novas de *Hyla* Laurenti, 1768 do
628 Estado de Goiás, Brasil (Amphibia, Anura, Hylidae). Boletim do Museu Nacional.
629 Nova Serie, Zoologia. Rio de Janeiro 422: 1–12.

630 Caramaschi, U.; Cruz, C. A. G. 2002. Taxonomic status of *Atelopus pachyrhynus*
631 Miranda-Ribeiro, 1920, redescription of *Melanophryniscus tumifrons* (Boulenger,
632 1905), and descriptions of two new species of *Melanophryniscus* from the state of Santa
633 Catarina, Brazil (Amphibia, Anura, Bufonidae). Arquivos do Museu Nacional. Rio de
634 Janeiro 60: 303–314.

635 Caramaschi, U.; Cruz, C. A. G. 2004. Duas novas espécies de Hyla do grupo de *H.*
636 *polytaenia* Cope, 1870 do sudeste do Brasil (Amphibia, Anura, Hylidae). Arquivos do
637 Museu Nacional. Rio de Janeiro 62: 247–254.

638 Caramaschi, U.; Cruz, C. A. G. 2011. A new, possibly threatened species of
639 *Melanophryniscus* Gallardo, 1961 from the state of Minas Gerais, southeastern Brazil
640 (Amphibia, Anura, Bufonidae). Boletim do Museu Nacional. Nova Serie, Zoologia. Rio
641 de Janeiro 528: 1–9.

642 Caramaschi, U.; Cruz, C. A. G. 2013. A new species of the *Hypsiboas polytaenius* clade
643 from southeastern Brazil (Anura: Hylidae). South American Journal of Herpetology 8:
644 121-126.

645 Caramaschi, U.; Feio, R. N. 1990. A new species of *Hyla* (Anura, Hylidae) from
646 southern Minas Gerais, Brazil. Copeia 1990: 542–546.

647 Caramaschi, U.; Jim, J. 1983a. A new microhylid frog, genus *Elachistocleis* (Amphibia,
648 Anura), from northeastern Brasil. Herpetologica 39: 390–394.

649 Caramaschi, U.; Jim, J. 1983b. Obserações sobre hábitos e desenvolvimento dos girinos
650 de *Phyllomedusa vaillanti* (Amphibia, Anura, Hylidae). Revista Brasileira de Biologia
651 43: 261─268.

652 Caramaschi, U.; Jim, J. 1983c. Uma nova especie de *Hyla* do grupo marmorata do
653 nordeste Brasileiro (Amphibia, Anura, Hylidae). Revista Brasileira de Biologia 43:
654 195–198.

655 Caramaschi, U.; Kisteumacher, G. 1989a. A new species of *Eleutherodactylus* (Anura:
656 Leptodactylidae) from Minas Gerais, southeastern Brazil. Herpetologica 44: 423–426.

657 Caramaschi, U.; Kisteumacher, G. 1989b. Duas novas especies de *Ololygon* Fitzinger,
658 1843, do sudeste do Brasil (amphibia, Anura, Hylidae). Boletim do Museu Nacional.
659 Nova Serie, Zoologia. Rio de Janeiro 327: 1–15.

660 Caramaschi, U.; Kisteumacher, G. 1989c. O girino de *Crossodactylus trachystomus*
661 (Reinhardt e Luetken, 1862) (Anura, Leptodactylidae). Revista Brasileira de Biologia
662 49: 237.

663 Caramaschi, U.; Napoli, M. F. 2012. Taxonomic revision of the *Odontophrynus*
664 *cultripes* species group, with description of a new related species (Anura,
665 Cycloramphidae). Zootaxa 3155: 1–20.

666 Caramaschi, U.; Niemeyer, H. 2003a. New species of the *Hyla albopunctata* group from
667 central Brazil (Amphibia, Anura, Hylidae). Boletim do Museu Nacional. Nova Serie,
668 Zoologia. Rio de Janeiro 504: 1–8.

669 Caramaschi, U.; Niemeyer, H. 2003b. Nova espécie do complexo de *Bufo margaritifer*
670 (Laurenti, 1768) do estado de Mato Grosso do Sul (Amphibia, Anura, Bufonidae).
671 Boletim do Museu Nacional 501: 1─16.

672 Caramaschi, U.; Niemeyer, H. 2004. Descrição do girino de *Lysapsus laevis* (Parker),
673 com notas sobre o ambiente, hábitos e desenvolvimento (Anura, Hylidae, Pseudinae).
674 Revista Brasileira de Zoologia 21: 449─452.

675 Caramaschi, U.; Peixoto, O. L. 2004. A new species of *Phyllodyes* (Anura: Hylidae)
676 from the state of Sergipe, northeastern Brazil. Amphibia-Reptilia 25: 1–8.

677 Caramaschi, U.; Pimenta, B. V. S. 2003. Duas novas espécies de *Chiasmocleis* Méhelÿ,
678 1904, da Mata Atlântica do Sul da Bahia, Brasil (Amphibia, Anura, Microhylidae).
679 Arquivos do Museu Nacional. Rio de Janeiro 61: 195–202.

680 Caramaschi, U.; Pombal, Jr., J. P. 2006. A new species of *Rhinella* Fitzinger, 1826 from
681 the Atlantic Rain Forest, eastern Brazil (Amphibia, Anura, Bufonidae). Papéis Avulsos
682 de Zoologia. São Paulo 46: 251–259.

683 Caramaschi, U.; Rodrigues, M. T. 2007. Taxonomic status of the species of *Gastrotheca*
684 Fitzinger, 1843 (Amphibia, Anura, Amphignathodontidae) of the Atlantic rain forest of
685 eastern Brazil, with description of a new species. Boletim do Museu Nacional. Nova
686 Serie, Zoologia. Rio de Janeiro 525: 1–19.

687 Caramaschi, U.; Rodrigues, M. T. 2003. A new large treefrog species, genus *Hyla*
688 Laurenti, 1768, from southern Bahia, Brazil (Amphiibia, Anura, Hylidae. Arquivos do
689 Museu Nacional. Rio de Janeiro 61: 255–260.

690 Caramaschi, U.; Sazima, I. 1984. Uma nova espécie de *Thoropa* da Serra do Cipó,
691 Minas Gerais, Brasil (Amphibia, Leptodactylidae). Revista Brasileira de Zoologia 2:
692 139–146.

693 Caramaschi, U.; Sazima, I. 1985. Uma nova espécie de *Crossodactylus* da Serra do
694 Cipó, Minas Gerais, Brasil (Amphibia, Leptodactylidae). Revista Brasileira de Zoologia
695 3: 43─49.

696 Caramaschi, U.; Velosa, A. 1996. Nova especie de *Hyla* Laurenti, 1768 do leste
697 brasileiro (Amphibia, Anura, Hylidae). Boletim do Museu Nacional. Nova Serie,
698 Zoologia. Rio de Janeiro 365: 1–7.

699 Caramaschi, U. 1979. O girino de *Odontophrynus carvalhoi* Savage and Cei, 1965
700 (Amphibia, Anura, Ceratophrydidae). Revista Brasileira de Biologia 39: 169─171

701 Caramaschi, U. 1996. Nova espécie de *Odontophrynus* Reinhardt & Lütken, 1862 do
702 Brasil Central (Amphibia, Anura, Leptodactylidae). Boletim do Museu Nacional. Nova
703 Serie, Zoologia. Rio de Janeiro 367: 1–8.

704 Caramaschi, U. 1998. Description of a second species of the genus *Xenohyla* (Anura:
705 Hylidae). Amphibia-Reptilia 19: 377–384.

706 Caramaschi, U. 2006. Redefinição do grupo de *Phyllomedusa hypochondrialis*, com
707 redescrição de *P. megacephala* (Miranda-Ribeiro, 1926), revalidação de *P. azurea*
708 Cope, 1862 e descrição de uma nova espécie (Amphibia, Anura, Hylidae). Arquivos do
709 Museu Nacional. Rio de Janeiro 64: 159–179.

710 Caramaschi, U. 2010a. Descrição do girino de *Sphaenorhynchus surdus* (Cichran, 1953)
711 (Anura, Hylidae). Boletim do Museu de Biologia Melo Leitão 27: 67─74

712 Caramaschi, U. 2010b. Notes on the taxonomic status of *Elachistocleis ovalis*
713 (Schneider, 1799) and description of five new species of *Elachistocleis* Parker, 1927
714 (Amphibia, Anura, Microhylidae). Boletim do Museu Nacional. Nova Serie, Zoologia.
715 Rio de Janeiro 527: 1–30.

716 Caramaschi, U. 2012. The generic position of *Rhinella skuki* Caramaschi, 2012 (Anura,
717 Bufonidae). Zootaxa 3527: 88.

718 Caramaschi, U.; Jim, J.; Carvalho, C. M. 1980. Observações sobre *Aplastodiscus*
719 *perviridis* A. Lutz (Amphibia, Anura, Hylidae). Revista Brasileira de Biologia 40:
720 405─408.

721 Caramaschi, U.; Carcerelli, L. C.; Feio, R. N. 1991. A new species of *Physalameus*
722 (Anura: Leptodactylidae) from Minas Gerais, southeastern Brazil. Herpetologica 47:
723 148–151.

724 Caramaschi, U.; da Silva, H. R.; Britto-Pereira, M. C. 1992. A new species of
725 *Phyllodytes* (Anura, Hylidae) from Southern Bahia, Brazil. Copeia 1992: 187─191.

726 Caramaschi, U.; Silva, H. R. da; Britto-Pereira, M. C. de. 1992. A new species of
727 *Phyllodytes* (Anura, Hylidae) from southern Bahia, Brazil. Copeia 1992: 187–191.

728 Caramaschi, U.; Napoli, M. F.; Bernardes, A. T. 2001. Nova espécie do grupo do *Hyla*
729 *circumdata* (Cope, 1870) do Estado de Minas Gerais, Brasil (Amphibia, Anura,
730 Hylidae). Boletim do Museu Nacional. Nova Serie, Zoologia. Rio de Janeiro 457: 1–11.

731 Caramaschi, U.; Feio, R. N.; Guimarães, Neto, A. S. 2003. A new, brightly colored
732 species of *Physalaemus* (Anura: Leptodactylidae) from Minas Gerias, southeastern
733 Brazil. Herpetologica 59: 519–524.

734 Caramaschi, U.; Pimenta, B. V. S.; Feio, R. N. 2004. Nova especie do grupo de *Hyla*
735 *geographica* Spix, 1824 da floresta Atlântica, Brasil (Amphibia, Anura, Hylidae).
736 Boletim do Museu Nacional 518: 1–14.

737 Caramaschi, U.; Feio, R. N.; São Pedro, V. de A. 2008. A new species of *Leptodactylus*
738 Fitzinger (Anura, Leptodactylidae) from Serra do Brigadeiro, state of Minas Gerais,
739 southeastern Brazil. Zootaxa 1861: 44–54.

740 Caramaschi, U.; Almeida, A. de P.; Gasparini, J. L. 2009a. Description of two new
741 species of *Sphaenorhynchus* (Anura, Hylidae) from the state of Espírito Santo,
742 southeastern Brazil. Zootaxa 2115: 34–46.

743 Caramaschi, U.; Cruz, C. A. G.; Nascimento, L. B. 2009b. A new species
744 of *Hypsiboas* of the *H. polytaenius* clade from southeastern Brazil (Anura: Hylidae).
745 South American Journal of Herpetology 4: 210–216.

746 Caramaschi, U.; Cruz, C. A. G.; Segalla, M. V. 2010. A new species of *Hypsiboas* of
747 the *H. polytaenius* clade from the state of Paraná, southern Brazil (Anura: Hylidae).
748 South American Journal of Herpetology 5: 169–174.

749 Caramaschi, U.; Salles, R. de O. L.; Cruz, C. A. G. 2012. A new species of
750 *Stereocyclops* Cope (Anura, Microhylidae) from southeastern Brazil. Zootaxa 3583: 83–
751 88.

752 Caramaschi, U.; Orrico, V. G. D.; Faivovich, J.; Dias, I. R.; Solé, M. 2013. A new
753 species of *Allophryne* (Anura: Allophrynidae) from the Atlantic Rain Forest Biome of
754 eastern Brazil. Herpetologica 69: 480–491.

755 Carcerelli, L. C.; Caramaschi, U. 1992. Ocorrência do gênero *Crossodactylus* Duméril
756 & Bibron, 1841 no nordeste brasileiro, com descrição de duas espécies novas
757 (Amphibia, Anura, Leptodactylidae). Revista Brasileira de Biologia 52: 415–422.

758 Cardoso, A. J.; Andrade, G. V. 1982. Nova espécie de *Hyla* do Parque Nacional Serra
759 da Canastra (Anura, Hylidae). Revista Brasileira de Biologia 42: 589─593.

760 Cardoso, A. J.; Haddad, C. F. B. 1982. Nova espécie de *Hyla* da Serra da Canastra
761 (Amphibia, Anura, Hylidae). Revista Brasileira de Biologia 42: 499–503.

762 Cardoso, A. J.; Haddad, C. F. B. 1984. Variabilidade acústica em diferentes populações
763 e interações agressivas de *Hyla minuta* (Amphibia, Anura). Ciência e Cultura 36:
764 1393 1399.

765 Cardoso, A. J.; Haddad, C. F. B. 1985. Nova espécie de *Physalaemus* do grupo
766 signiferus (Amphibia, Anura, Leptodactylidae). Revista Brasileira de Biologia 45: 33–
767 37.

768 Cardoso, A. J.; Haddad. C. F. B. 1990. Redescrição e biologia de *Paratelmatobius*
769 *gaigeae* (Anura, Leptodactylidae). Papéis Avulos de Zoologia 37: 125─132.

770 Cardoso, A. J.; Heyer, W. R. 1995. Advertisement, aggressive, and possible seismic
771 signals of the frog *Leptodactylus syphax* (Amphibia, Leptodactylidae). Alytes 13:
772 67 76.

773 Cardoso, A. J.; Sazima, I. 1977. Batracofagia na fase adulta e larvária da rã pimenta.
774 *Leptdactylus labyrinthicus* (Spix, 1824) – Anura, Leptodactylidae. Ciência e Cultura 29:
775 1130─1132.

776 Cardoso, A. J.; Sazima, I. 1980. Nova espécie de *Hyla* do sudeste brasileiro (Amphibia,
777 Anura, Hylidae). Revista Brasileira de Biologia 40: 75–79.

778 Cardoso, A. J.; Vielliard, J. M. E. 1985. Caracterização bio-acústica da população
779 topotípica de *Hyla rubicundula* (Amphibia, Anura). Revista Brasileira de Zoologia 2:
780 423 426.

781 Cardoso, A. J.; Vielliard, J.M.E. 1990. Vocalização de anfíbios anuros de um ambiente
782 aberto, em Cruzeiro do Sul, Estado do Acre. Revista Brasileira de Biologia. 50:
783 229 242.

784 Cardoso, A. J. 1983. Descrição e biologia de uma nova espécie de *Hyla* Laurenti, 1768
785 (Amphibia, Anura, Hylidae). Iheringia 62: 37–45.

786 Cardozo, D.; Toledo, L. F. 2013. Taxonomic status of *Pseudopaludicola*
787 *riopiedadensis* Mercadal de Barrio and Barrio, 1994 (Anura, Leptodactylidae,
788 Leiuperinae). Zootaxa 3734: 571–582.

789 Carnaval, A. C. O. Q.; Peixoto, O. L. 2004. A new species of *Hyla* from northeastern
790 Brazil (Amphibia, Anura, Hylidae). Herpetologica 60: 387–395.

791 Carneiro, M. C. L.; Magalhães, P. S.; Juncá, F. A. 2004. Descrição do girino e
792 vocalização de *Scinax pachycrus* (Miranda-Ribeiro, 1937) (Amphibia, Anura, Hylidae).
793 Arquivos do Museu Nacional 62: 241 246.

794 Carrizo, G. R. 1991. Sobre los hílidos de Misiones, Argentina, con la descripción de una
795 nueva especie *Hyla caingua* n. sp.. Cuadernos de Herpetología 5: 32–39.

796 Carvalho, T. R. de; Giaretta, A. A. 2013a. A reappraisal of the geographic distribution
797 of *Bokermannohyla sazimai* (Anura: Hylidae) through morphological and bioacoustic
798 approaches. Phyllomedusa 12: 33–45.

799 Carvalho, T. R. de; Giaretta, A. A. 2013b. Bioacoustics reveals two new syntopic
800 species of *Adenomera* Steindachner (Anura: Leptodactylidae: Leptodactylinae) in the
801 Cerrado of central Brazil . Zootaxa 3731: 533–551.

802 Carvalho, T. R. de; Giaretta, A. A. 2013c. Taxonomic circumscription of *Adenomera*
803 *martinezi* (Bokermann, 1956) (Anura: Leptodactylidae: Leptodactylinae) with the
804 recognition of a new cryptic taxon through a bioacoustic approach . Zootaxa 3701: 207–
805 237.

arvalho, T. R. de; Martins, L. B. 2012. Advertisement call of *Haddadus binotatus* (Spix, 1824) (Anura: Terrarana: Craugastoridae) from three localities in the State of Rio de Janeiro, with comments on its bioacoustic variability. Herpetology Notes 5: 419 422.

Carvalho, A. L. de. 1946. Um novo gênero de ceratofridídeo do sudeste baiano. Boletim do Museu Nacional. Nova Serie, Zoologia. Rio de Janeiro 73: 1–18.

Carvalho, A. L. de. 1949. Notas sobre os hábitos de *Dendrophryniscus brevipollicatus* Espada (Amphibia, Anura). Revista Brasileira de Biologia 9: 223─227

Carvalho, A. L. de. 1954. A preliminary synopsis of the genera of American microhylid frogs. Occasional Papers of the Museum of Zoology, University of Michigan 555: 1–19.

Carvalho, T. R. de. 2012. A new species of *Pseudopaludicola* Miranda-Ribeiro (Leiuperinae: Leptodactylidae: Anura) from the Cerrado of southeastern Brazil with a distinctive advertisement call pattern. Zootaxa 3328: 47–54.

Carvalho, R. R. Jr.; Galdino, C. A. B.; Nascimento, L. B. 2006. Notes on the courtship behavior of *Aplastodiscus arildae* (Cruz & Peixoto, 1985) at na urban forest fragment in southeastern Brazil (Amphibia, Anura, Hylidae). Arquivos do Museu Nacional 64: 247 254.

Carvalho, T. R. de; Giaretta, A. A.; Facure, K. G. 2010a. A new species of *Hypsiboas* Wagler (Anura: Hylidae) closely related to *H. multifasciatus* Günther from southeastern Brazil. Zootaxa 2521: 37–52.

Carvalho, V. T. de; MacCulloch, R. D.; Bonora, L.; Vogt, R. C. 2010b. New Species of *Stefania* (Anura: Cryptobatrachidae) from northern Amazonas, Brazil. Journal of Herpetology 44: 229–235.

828 Carvalho, T. R. de; Giaretta, A. A.; Magrini, L. 2012. A new species of the
829 *Bokermannohyla circumdata* group (Anura: Hylidae) from southeastern Brazil, with
830 bioacoustic data on seven species of the genus. Zootaxa 3321: 37–55.

831 Carvalho, T. R. de; Giaretta, A. A.; Teixeira, B. F. V.; Martins, L. B. 2013b. New
832 bioacoustic and distributional data on *Bokermannohyla sapiranga* Brandão et al., 2012
833 (Anura: Hylidae): revisiting its diagnosis in comparison with *B. pseudopseudis*
834 (Miranda-Ribeiro, 1937). Zootaxa 3746: 383–392.

835 Carvalho, T. R. de; Leite, F. S. F.; Pezzuti, T. L. 2013a. A new species of *Leptodactylus*
836 Fitzinger (Anura, Leptodactylidae, Leptodactylinae) from montane rock fields of the
837 Chapada Diamantina, northeastern Brazil . Zootaxa 3701: 349–364.

838 Carvalho, T. R. de; Tolentino, V. C. M.; Giaretta, A. A. 2013c. Advertisement call of
839 *Rhinella pygmaea* (Myers and Carvalho, 1952) (Anura: Bufonidae) from the northern
840 State of Rio de Janeiro. Herpetology Notes 6: 229 231.

841 Carvalho, T. R. de; Martins, L. B.; Giaretta, A. A. 2015a. The complex vocalization of
842 *Scinax cardosoi* (Anura: Hylidae), with comments on advertisement calls in the S. ruber
843 Clade. Phyllomedusa 14: 127 137.

844 Carvalho, T. R. de; Teixeira, B. F. V.; Duellman, W. E.; Giaretta, A. A. 2015b. *Scinax*
845 *cruentommus* (Anura: Hylidae) in the upper Rio Negro drainage, Amazonas state,
846 Brazil, with the redescription of its advertisement call. Phyllomedusa 14: 139 146.

847 Carvalho, T. R. de; Teixeira, B. F. V.; Martins, L. B.; Giaretta, A. A. 2015c.
848 Intraspecific variation and new distributional records for *Pseudopaludicola* species
849 (Anura, Leptodactylidae, Leiuperinae) with trilled advertisement call pattern: diagnostic

850 characters revisited and taxonomic implications. North-Western Journal of Zoology 11:
851 262-273.

852 Carvalho, T. R. de; Martins, L. B.; Giaretta, A. A. 2016. A new account for the
853 endangered Cerrado Rocket Frog *Allobates goianus* (Bokermann, 1975) (Anura:
854 Aromobatidae), with comments on taxonomy and conservation. Acta Herpetologica 11:
855 21 30.

856 Carvalho-e-Silva, S. P.; Carnaval, A. C. O. Q. 1997. Observations on the biology of
857 *Scinax flavoguttatus* (Lutz et Lutz) and description of its tadpoles (Amphibia: Anura:
858 Hylidae). Revue Française de Aquariologie 24: 59─64.

859 Carvalho-e-Silva, A. M. P. T. and Carvalho-e-Silva, S. P. 1994a. Donnés sur la biologie
860 et description des larves de *Bufo pygmaeus* Myers et Carvalho (Amphibia, Anura,
861 Bufonidae). Revue Française de Aquariologie 21:53-56.

862 Carvalho-e-Silva, S. P.; Carvalho-e-Silva, A. M. P. T. 1994. Descrição das larvas de
863 *Ololygon albicans* e de *Ololygon trapicheiroi* com considerações sobre sua biologia
864 (Amphibia, Anura, Hylidae). Revista Brasileira de Biologia 54:55-62.

865 Carvalho-e-Silva, S. P.; Carvalho-e-Silva, A. M. P. T. 1998. Aspects of the biology and
866 description of the larvae of *Scinax argyreornatus* and *Scinax humilis* (Amphibia: Anura:
867 Hylidae). Revue Française de Aquariologie 25: 47─52.

868 Carvalho-e-Silva, A. M. P. T. de; Carvalho-e-Silva, S. P. de. 2005. New species of the
869 *Hyla albofrenata* group, from the states of Rio de Janeiro and São Paulo, Brazil (Anura,
870 Hylidae). Journal of Herpetology 39: 73–81.

871 Carvalho-e-Silva, S. P.; Peixoto, O. L. 1991. Duas novas espécies de *Ololygon* para os
872 Estados do Rio de Janeiro e Espírito Santo (Amphibia, Anura, Hylidae). Revista
873 Brasileira de Biologia 51: 263–270.

874 Carvalho-e-Silva, S. P.; Gomes, M. R.; Peixoto, O. L.1995. Descrição dos girinos de
875 *Scinax angrensis* (B. Lutz, 1973) e de *Scinax kautskyi* (Carvalho e Silva e Peixoto,
876 1991) (Amphibia, Anura, Hylidae). Revista Brasileira de Biologia 55: 61─65.

877 Carvalho-e-Silva, S. P.; Pinto, A. L. C.; Carvalho-e-Silva, A. M. P. T. 2002. Aspectos
878 da reprodução, da vocalização e da larva de *Phrynohyas mesophaea* Hensel (Amphibia,
879 Anura, Hylidae). Revista Aquarium 35: 19─24.

880 Carvalho-e-Silva, S. P.; Carvalho-e-Silva, A. M. P. T.; Izecksohn, E. 2003. Nova
881 espécie de *Hyla* Laurenti do grupo de *H. microcephala* Cope (Amphibia, Anura,
882 Hylidae) do nordeste do Brasil. Revista Brasileira de Zoologia 20: 553─558.

883 Carvalho-e-Silva, A. M. P. T. de; Silva, G. R. da; Carvalho-e-Silva, S. P. de. 2009. A
884 new species of *Phasmahyla* Cruz, 1990 from the Atlantic Forest in the state of Rio de
885 Janeiro, Brazil (Amphibia, Hylidae, Phyllomedusinae). Zootaxa 2120: 15–26.

886 Carvalho-e-Silva, A. M. P. T. de; Mongin, M. M.; Izecksohn, E.; Carvalho-e-Silva, S.
887 P. de. 2010. A new species of *Dendrophryniscus* Jiménez–de–la–Espada from the
888 Parque Nacional da Serra dos Órgãos, Teresópolis, state of Rio de Janeiro, Brazil
889 (Amphibia, Anura, Bufonidae). Zootaxa 2632: 46–52.

890 Casal, F. S. C.; Juncá, F. A. 2008. Girino e canto de anúncio de *Hypsiboas crepitans*
891 (Amphibia: Anura: Hylidae) do estado da Bahia, Brasil, e considerações taxonômicas.
892 Boletim do Museu Paraense Emílio Goeldi 3: 217 224.

893 Cascon, P.; Peixoto, O.L. 1985. Observações sobre a larva de *Leptodactylus troglodytes*
894 (Amphibia, Anura, Leptodactylidae). Revista Brasileira de Biologia 45: 361─4.

895 Cassimiro, J.; Verdade, V. K.; Rodrigues, M. T. 2008. A large and enigmatic new
896 eleutherodactyline frog (Anura, Strabomantidae) from Serra do Sincorá, Espinhaço
897 range, northeastern Brazil. Zootaxa 1761: 59–68.

898 Cassini, C. S.; Cruz, C. A. G.; Caramaschi, U. 2010. Taxonomic review of *Physalaemus*
899 *olfersii* (Lichtenstein & Martens, 1856) with revalidation of *Physalaemus lateristriga*
900 (Steindachner, 1864) and description of two new related species (Anura: Leiuperidae).
901 Zootaxa 2491: 1–33.

902 Castanho, L. M.; Haddad, C. F. B. 2000. New species of *Eleutherodactylus* (Amphibia:
903 Leptodactylidae) from Guaraqueçaba, Atlantic forest of Brazil. Copeia 2000: 777–781.

904 Castroviejo-Fischer, S.; Padial, J. M.; Chaparro, J. C.; Aguayo-Vedia, C. R.; De la Riva,
905 I. 2009. A new species of *Hyalinobatrachium* (Anura: Centrolenidae) from the
906 Amazonian slopes of the central Andes, with comments on the diversity of the genus in
907 the area. Zootaxa 2143: 24–44.

908 Castroviejo-Fisher, S.; Vilà, C.; Ayarzagüena, J.; Blanc, M.; Ernst, R. 2011. Species
909 diversity of *Hyalinobatrachium* glassfrogs (Amphibia: Centrolenidae) from the Guiana
910 Shield, with the description of two new species. Zootaxa 3132: 1–55.

911 Cei, J. M. 1949. Costumbres nupciales y reproduccion de un batracio caracteristico
912 chaqueño. Acta Zoologica Lilloana 8: 105─110.

913 Cei, J. M. 1950. *Leptodactylus chaquensis* n. sp. y el valor sistematico real de la especie
914 Linneana *Leptodactylus ocellatus* en la Argentina. Acta Zoologica Lilloana 9: 395–423.

915 Cei, J. M. 1968. Notes on the tadpoles and breeding ecology of *Lepidobatrachus*
916 (Amphibia: Ceratophryidae). Herpetologica 24(2): 141─146.

917 Cei, J. M. 1980. Amphibians of Argentina. Monitore Zoologico Italiano N.S.
918 Monografia 1─609.

919 Cei, J. M. 1985. Un nuevo y peculiar *Odontophrynus* de la Sierra de Guasayán,
920 Santiago del Estero, Argentina (Anura: Leptodactylidae). Cuadernos de Herpetología 1:
921 1–13.

922 Céspedez, J. A. 2000. Una nueva especie de *Bufo* del grupo granulosus (Anura:
923 Bufonidae) del Nordeste Argentino. FACENA. Facultad de Ciencias Exactas y
924 Naturales y Agrimensura, Universidad Nacional del Nordeste, Corrientes, Argentina 15:
925 72–91.

926 Cintra, C. E. D.; Silva, H. L. R. da; Silva, Jr., N. J. da; Garcia, P. C. de A.; Zaher, H.
927 2009. A new species of *Trachycephalus* (Amphibia, Anura, Hylidae) from the state of
928 Goiás, Brazil. Zootaxa 1975: 58–68.

929 Clemente-Carvalho, R. B. G., A. A. Giaretta, T. H. Condez, C. F. B. Haddad, and S. F.
930 dos Reis. 2012. A new species of miniaturized toadlet, genus *Brachycephalus* (Anura:
931 Brachycephalidae), from the Atlantic Forest of southeastern Brazil. Herpetologica 68:
932 365–374.

933 Cochran, D. M.; Goin, C. J. 1959. A new frog of the genus *Limnomedusa* from
934 Colombia. Copeia 1959: 208–210.

935 Cochran, D. M.; Goin, C. J. 1970. Frogs of Colombia. Bulletin of the United States
936 National Museum 288: 1–655.

937 Cochran, D. M. 1938. Diagnoses of new frogs from Brazil. Proceedings of the
938 Biological Society of Washington 51: 41–42.

939 Cochran, D. M. 1948a. A new subspecies of frog from Itatiaya, Brazil. American
940 Museum Novitates 1375: 1–3.

941 Cochran, D. M. 1948b. A new subspecies of tree frog from Pernambuco, Brazil. Journal
942 of the Washington Academy of Sciences 38: 316–318.

943 Cochran, D. M. 1952. Two Brazilian frogs: *Hyla werneri*, n. nom., and *Hyla similis* n.
944 sp. Journal of the Washington Academy of Sciences 42: 50–53.

945 Cochran, D. M. 1953. Three new Brazilian frogs. Herpetologica 8: 111–115.

946 Cocroft, R. B.; McDiarmid, R. W.; Jaslow, A. P.; Ruiz-Carranza, P. M. 1990.
947 Vocalizations of eight species of *Atelopus* (Anura: Bufonidae) with comments on
948 communication in the genus. Copeia 1990: 631–643.

949 Condez, T. H.; Clemente-Carvalho, R. B. G.; Haddad, C. F. B.; Reis, S. F. dos. 2014. A
950 new species of *Brachycephalus* (Anura: Brachycephalidae) from the highlands of the
951 Atlantic Forest, southeastern Brazil. Herpetologica 70: 89–99.

952 Condez, T. H.; Monteiro, J. P. de C.; Comitti, E. J.; Garcia, P. C. de A.; Amaral, I. B.;
953 Haddad, C. F. B. 2016. A new species of flea-toad (Anura: Brachycephalidae) from
954 southern Atlantic Forest, Brazil. Zootaxa 4083: 40–56.

955 Conte, C. E.; Lingnau, R.; Kwet, A. 2005. Description of the advertisement call of *Hyla*
956 *ehrhardtii* Müller, 1924 and new distribution records (Anura: Hylidae). Salamandra 41:
957 147–151.

Conte, C. E.; Nomura, F.; Rossa-Feres, D. C.; d'Heursel, A.; Haddad, C. F. B. 2007. The tadpole of *Scinax catharinae* (Anura: Hylidae) with description of the internal oral morphology, and a review of the tadpoles from the Scinax catharinae group. Amphibia-Reptilia 28: 177─192.

Conte, C.E.; Nomura, F.; Machado, R. A., Kwet, A.; Lingnau, R.; Rossa-Feres, D.C. 2010. New records in the geographic distribution range of the anurans of the Araucaria Forest and considerations on their vocalizations. Biota Neotrop. 10(2): 201 224.

Conte, C. E., K. Araujo-Vieira, L. B. Crivellari, and B. v. M. Berneck. 2016. A new species of *Scinax* Wagler (Anura: Hylidae) from Paraná, southern Brazil . Zootaxa 4193: 245–265.

Cope, E. D. 1861. Descriptions of new species of the reptilian genera *Hyperolius*, *Liuperus* and *Tropidodipsas*. Proceedings of the Academy of Natural Sciences of Philadelphia 12: 517–518.

Cope, E. D. 1862a. On some new and little known American Anura. Proceedings of the Academy of Natural Sciences of Philadelphia 14: 151–159.

Cope, E. D. 1862b. Catalogues of the reptiles obtained during the explorations of the Parana, Paraguay, Vermejo and Uraguay Rivers, by Capt. Thos. J. Page, U.S.N.; and of those procured by Lieut. N. Michler, U.S. Top. Eng., Commander of the expedition conducting the survey of the Atrato River. Proceedings of the Academy of Natural Sciences of Philadelphia 14: 346–359.

Cope, E. D. 1868. An examination of the Reptilia and Batrachia obtained by the Orton Expedition to Equador and the Upper Amazon, with notes on other species. Proceedings of the Academy of Natural Sciences of Philadelphia 20: 96–140.

981 Cope, E. D. 1869. Sixth contribution to the herpetology of tropical America.
982 Proceedings of the Academy of Natural Sciences of Philadelphia 20: 305–312.

983 Cope, E. D. 1870. Seventh contribution to the herpetology of tropical America.
984 Proceedings of the American Philosophical Society 11: 147–169.

985 Cope, E. D. 1871a. Ninth contribution to the herpetology of tropical America.
986 Proceedings of the Academy of Natural Sciences of Philadelphia 23: 200–224.

987 Cope, E. D. 1871b. Eighth contribution to the herpetology of tropical America.
988 Proceedings of the American Philosophical Society 11: 553–559.

989 Cope, E. D. 1874. On some Batrachia and Nematognathi brought from the upper
990 Amazon by Prof. Orton. Proceedings of the Academy of Natural Sciences of
991 Philadelphia 26: 120–137.

992 Cope, E. D. 1886. Thirteenth contribution to the herpetology of tropical America.
993 Proceedings of the American Philosophical Society 23: 271–287.

994 Cope, E. D. 1887. Synopsis of the Batrachia and Reptilia obtained by H. H. Smith, in
995 the Province of Mato Grosso, Brazil. Proceedings of the American Philosophical
996 Society 24: 44–60.

997 Costa, T. R. N.; Toledo, L. F. 2013. *Physalaemus spiniger* (Anura: Leiuperinae): A frog
998 with an extensive vocal repertoire. Journal of Herpetology 47: 530–538.

999 Costa, R. C.; Facure, K. G.; Giaretta, A. A. 2006. Courtship, vocalization, and tadpole
1000 description of *Epipedobates flavopictus* (Anura: Dendrobatidae) in southern Goiás,
1001 Brazil. Biota Neotropica 6: 1─9.

1002 Costa, T. R. N.; Lingnau, R.; Toledo. L. F. 2009. The tadpole of the Brazilian torrent
1003 frog *Hylodes heyeri* (Anura; Hylodidae). Zootaxa 2222: 66–68.

1004 Costa, P. N.; Lourenço, A. C. C.; Almeida-Santos, P.; Sluys, M. V. 2010a. The tadpole
1005 of *Bokermannohyla gouveai* (Peixoto and Cruz, 1992). Zootaxa 2418: 61─64.

1006 Costa, P. N.; Silva-Soares, T.; Weber, L. N.; Carvalho-e-Silva, A. M. P. T. 2010b.
1007 Redescription of tadpole of the hylodid frog *Hylodes asper* (Müller, 1924). Zootaxa
1008 2521: 65–68.

1009 Costa, P. N.; Siqueira, C. C.; Vrcibradic, D.; Weber, L. N.; Rocha, C. F. D. 2010c. The
1010 tadpole of the hylodid frog *Hylodes charadranaetes* Heyer and Cocroft, 1986. Zootaxa
1011 2680: 65–68.

1012 Costa, T. B.; Laranjeiras, D. O.; Röhr, D. L.; Magalhães, F. de M.; Juncá, F. A.; Garda,
1013 A. A. 2014. The advertisement call of *Haddadus aramunha* (Cassimiro, Verdade &
1014 Rodrigues, 2008) (Anura, Craugastoridae). Zootaxa 3784: 94–96.

1015 Costa-Campos, C. E.; Lima, A. L. P.; Amézquita, A. 2016. The advertisement call of
1016 *Ameerega pulchripecta* (Silverstone, 1976) (Anura, Dendrobatidae). Zootaxa 4136:
1017 387–389.

1018 Cocroft, R. B.; Heyer, W. R. 1988. Notes on the frog genus *Thoropa* (Amphibia:
1019 Leptodactylidae) with a description of a new species (*Thoropa saxatilis*). Proceedings
1020 of the Biological Society of Washington 101: 209–220.

1021 Crombie, R. I.; Heyer, W. R. 1983. *Leptodactylus longirostris* (Anura:
1022 Leptodactylidae): Advertisement call, tadpole, ecological and distributional notes.
1023 Revista Brasileira de Biologia 43: 291–296.

1024 Cruz, C. A. G.; Dias, A. G. 1991. Girinos de grupo "microcephala" do Estado do Rio de
1025 Janeiro (Amphibia, Anura, Hylidae). Revista Brasileira de Zoologia 7: 679─683.

1026 Cruz, C. A. G.; Fusinatto, L. A. 2008. A new species of *Dendrophryniscus* Jiménez de
1027 la Espada, 1871 (Amphibia, Anura, Bufonidae) from the Atlantic Rain Forest of Rio
1028 Grande do Sul, Brazil. South American Journal of Herpetology 3: 22–26.

1029 Cruz, C. A. G.; Napoli, M. F. 2010. A new species of smooth horned frog, genus
1030 *Proceratophrys* Miranda-Ribeiro (Amphibia: Anura: Cycloramphidae), from the
1031 Atlantic Rainforest of eastern Bahia, Brazil. Zootaxa 2660: 57–67.

1032 Cruz, C. A. G.; Peixoto, O. L. 1978. Notas sobre o girino de *Dasypops schirchi*
1033 Miranda-Ribeiro (Amphibia, Anura, Microhylidae). Revista Brasileira de Biologia 38:
1034 297─299.

1035 Cruz, C. A. G.; Peixoto, O. L. 1980. Notas sobre o girino de *Sphaenorhynchus*
1036 *orophilus* (Lutz and Lutz, 1938) (Amphibia, Anura, Hylidae). Revista Brasileira de
1037 Biologia 40: 383─386.

1038 Cruz, C. A. G.; Peixoto, O. L. 1982. Sobre a biologia de *Atelopus pernambucensis*
1039 Bokermnn, 1962 (Amphibia, Anura, Bufonidae). Revista Brasileira de Biologia 42:
1040 627─629.

1041 Cruz, C. A. G.; Peixoto, O. L. 1983. Uma nova especie de *Hyla* do estado do Espirito
1042 Santo, Brasil (Amphibia, Anura, Hylidae). Revista Brasileira de Biologia 42: 721–724.

1043 Cruz, C. A. G.; Peixoto, O. L. 1985. Especies verdes de *Hyla*: o complexo
1044 "Albosignata" (Amphibia, Anura, Hylidae). Arquivos de Universidade Federal Rural do
1045 Rio de Janeiro 7: 31–47.

1046 Cruz, C. A. G.; Peixoto, O. L. 1987. Especies verdes de *Hyla*: o complexo
1047 "Albofrenata" (Amphibia, Anura, Hylidae). Arquivos de Universidade Federal Rural do
1048 Rio de Janeiro 8: 59–70.

1049 Cruz, C. A. G.; Pimenta, B. V. S. 2004. New species of *Physalaemus* Fitzinger, 1826
1050 from southern Bahia, Brazil (Anura, Leptodactylidae). Journal of Herpetology 38: 480–
1051 486.

1052 Cruz, C. A. G. 1973. Observações sobre o girino de *Sphaenorhynchus* planicola (Lutz
1053 and Lutz, 1938). (Amphibia, Anura, Hylidae). Arquivos da Universidade Federal Rural
1054 do Rio de Janeiro 3: 83─86.

1055 Cruz, C. A. G. 1980. Descrição de uma nova espécies de Phyllomedusinae do estado do
1056 Espírito Santo, Brasil (Amphibia, Anura, Hylidae). Revista Brasileira de Biologia 40:
1057 683–687.

1058 Cruz, C. A. G. 1982. Conceituação de grupos de espécies de Phyllomedusinae
1059 brasileiras com base em caracteres larvários (Amphibia, Anura, Hylidae). Arquivos da
1060 Universidade Federal Rural do Rio de Janeiro 5: 147─171.

1061 Cruz, C. A. G. 1988. Sobre *Phyllomedusa aspera* e a descricão de uma especie nova
1062 desse genero (Amphibia, Anura, Hylidae). Arquivos de Universidade Federal Rural do
1063 Rio de Janeiro 11: 39–44.

1064 Cruz, C. A. G. 1991. Descriçao de duas espécies novas de Phyllomedusinae do sudeste
1065 Brasileiro (Amphibia, Anura, Hylidae). Revista Brasileira de Biologia 51: 271–275.

1066 Cruz, C. A. G.; Caramaschi, U.; Izecksohn,. E. 1997. The genus *Chiasmocleis* Méhely,
1067 1904 (Anura, Microhylidae) in the Atlantic Rain Forest of Brazil, with description of
1068 three new species. Alytes. Paris 15: 49–71.

1069 Cruz, C. A. G.; Caramaschi, U.; Freire, E. M. X. 1999. Occurrence of the genus
1070 *Chiasmocleis* (Anura: Microhylidae) in the State of Alagoas, north-eastern Brazil, with
1071 a description of a new species. Journal of Zoology. London 249: 123–126.

1072 Cruz, C. A. G.; Caramaschi, U.; Dias, A. G. 2000. Espécie nova de *Hyla* Laurenti, 1768
1073 do estado do Rio de Janeiro, Brasil (Amphibia, Anura, Hylidae). Boletim do Museu
1074 Nacional 434: 1-8.

1075 Cruz, C. A. G.; Pimenta, B. V. S.; Silvano, D. L. 2003. Duas novas espécies
1076 pertencentes ao complexo de *Hyla albosignata* Lutz & Lutz, 1938, do leste do Brasil
1077 (Amphibia, Anura, Hylidae). Boletim do Museu Nacional. Nova Serie, Zoologia. Rio de
1078 Janeiro 503: 1–13.

1079 Cruz, C. A. G.; Prado, G. M.; Izecksohn, E. 2005. Nova espécie de *Proceratophrys*
1080 Miranda-Ribeiro, 1920 do sudest do Brasil (Amphibia, Anura, Leptodactylidae).
1081 Arquivos do Museu Nacional 63: 289–295.

1082 Cruz, C. A. G.; Caramaschi, U.; Napoli, M. F. 2007a. A new species of *Chiasmocleis*
1083 (Anura, Microhylidae) from the Atlantic Rain Forest of northeastern Bahia, Brazil.
1084 South American Journal of Herpetology 2: 47–51.

1085 Cruz, C. A. G.; Feio, R. N.; Cardoso, M. C. S. 2007b. Description of a new species of
1086 *Phyllodytes* Wagler, 1830 (Anura, Hylidae) from the Atlantic Rain Forest of the states
1087 of Minas Gerais and Bahia, Brazil. Arquivos do Museu Nacional. Rio de Janeiro 64:
1088 321–324.

1089 Cruz, C. A. G.; Feio, R. N.; Cassini, C. S. 2007c. Nova espécie de *Chiasmocleis*
1090 Méhelÿ, 1904 (Amphibia, Anura, Microhylidae) da Serra da Maniqueira, Estado do
1091 Minas Gerais, Brasil. Arquivos do Museu Nacional. Rio de Janeiro 65: 33–38.

1092 Cruz, C. A. G.; Nascimento, L. B.; Feio, R. N. 2007d. A new species of the genus
1093 *Physalaemus* Fitzinger, 1826 (Anura, Leiuperidae) from southeastern Brazil. Amphibia-
1094 Reptilia 28: 457─465.

1095 Cruz, C. A. G.; Cassini, C. S.; Caramaschi, U. 2008a. A new species of the genus
1096 *Physalaemus* Fitzinger, 1826 (Anura, Leiuperidae) from southern Brazil. South
1097 American Journal of Herpetology 3: 239–243.

1098 Cruz, C. A. G.; Feio, R. N.; Nascimento, L. B. 2008b. A new species of *Phasmahyla*
1099 Cruz, 1990 (Anura: Hylidae) from the Atlantic rain forest of the states of Minas Gerais
1100 and Bahia, Brazil. Amphibia-Reptilia 29: 311–318.

1101 Cruz, C. A. G.; Napoli, M. F.; Fonseca, P. M. 2008c. A new species of *Phasmahyla*
1102 Cruz, 1990 (Anura: Hylidae) from the state of Bahia, Brazil. South American Journal of
1103 Herpetology 3: 187–195.

1104 Cruz, C. A. G.; Nunes, I.; Lima, M. G. de. 2011. A new Scinax Wagler belonging to the
1105 *S. catharinae* clade (Anura: Hylidae) from the state of Alagoas, northeastern Brazil.
1106 Zootaxa 3096: 18–26.

1107 Cruz, C. A. G.; Nunes, I.; Juncá, F. A. 2012. Redescription of *Proceratophrys cristiceps*
1108 (Müller, 1883) (Amphibia, Anura, Odontophrynidae), with description of two new
1109 species without eyelid appendages from northeastern Brazil. South American Journal of
1110 Herpetology 7: 110–122.

Cruz, D.; Marciano, E., Jr.; Napoli, M. F. 2014. Advertisement and courtship calls of *Phyllodytes wuchereri* (Peters, 1873) (Anura: Hylidae). Zootaxa 3774: 97–100.

Cuvier, G. L. C. F. D. 1797 "An. VI". Tableau Élémentaire de l'Histoire Naturelle des Animaux. Paris: Baudoin.

Da Silva, H. R.; Ouvernay, D. 2012. A new species of stream-dwelling frog of the genus *Cycloramphus* (Anura, Cycloramphidae) from the State of Rio de Janeiro, Brazil. Zootaxa 3407: 49–60.

Daudin, F.-M. 1800. Histoire Naturelle des Quadrupèdes Ovipaires. Livraison 2. Paris: Marchant et Cie.

De Abreu, R. O.; Napoli, M. F.; Trevisan, C. C.; Camardelli, M.; Dória, T. A. F.; Silva, L. M. 2015. The tadpole of *Scinax melanodactylus* (Lourenço, Luna & Pombal Jr, 2014)(Amphibia, Anura, Hylidae). Zootaxa, 3981(3): 430─436.

De la Riva, I. 1999. A new *Phyllomedusa* from southwestern Amazonia (Amphibia: Anura: Hylidae). Revista Española de Herpetología 13: 123–131.

De La Riva, I.; Márquez, R.; Bosch, J. 1994. Advertisement calls of Bolivian species of *Scinax* (Amphibia, Anura, Hylidae). Bijdragen tot de Dierkunde 64: 75–85.

De La Riva, I.; Márquez, R.; Bosch, J. 1995. Advertisement Calls of Eight Bolivian Hylids (Amphibia, Anura). Journal of Herpetology 29(1): 113 118.

De La Riva, I.; Bosch, J.; Marquez, R. 1996. Advertisement calls of two bolivian toads (Anura: Bufonidae: Bufo). Herpetological Journal 6: 55–61.

1131 De La Riva, I.; Márquez, R.; Bosch, J. 1996. Advertisement calls of four microhylid
1132 frogs from Bolivia (Amphibia, Anura). The American Midland Naturalist Journal
1133 136(2): 418 422.

1134 De La Riva, I.; Márquez, R.; Bosch, J. 1997. Description of the advertisement calls of
1135 some South American Hyidae (Amphibia, Anura): taxonomic and methodological
1136 consequences. Bonner zoologische Beiträge 47(1-2): 175 185.

1137 De Oliveira, F. F. R.; Nascimento, L. B.; Eterovick, P. C.; Sazima, I. 2013. Description
1138 of the tadpole and redescription of the advertisement call of *Physalaemus evangelistai*
1139 (Anura, Leiuperidae), with notes on its natural history. Journal of Herpetology 47:
1140 539─543.

1141 De Sá, R. O.; Gerhau, A. 1983. Observaciones sobre la biología de *Phyllomedusa*
1142 *iheringi* Boulenger, 1885 (Anura, Hylidae). Boletín de la Sociedad Zoológica del
1143 Uruguay 1: 44─49.

1144 De Sá, R. O.; Langone, J. A. 2002. The tadpole of *Proceratophyrs avelinoi*
1145 (Anura:Leptodactylidae). Journal of Herpetology 36: 490─494.

1146 De Sá, R. O. 1995. *Hyla albopunctata*. Catalogue of American amphibians and reptiles
1147 602: 1─5.

1148 De Sá, R. O. 1995. *Hyla multifasciata*. Catalogue of American amphibians and reptiles
1149 624: 1─4.

1150 De Sá, R. O.; Wassersug, R.; Kehr, A. I. 1997. Description of tadpoles of three species
1151 of *Scinax* (Anura: Hylidae). Herpetological Journal 7: 13─17.

De Sá, R. O.; Brandão, R.; Guimarães, L. D. 2007. Description of the tadpole of *Leptodactylus pustulatus* Peters, 1870 (Anura: Leptodactylidae). Zootaxa 1523: 49─58.

De Sá F. P.; Canedo C.; Lyra, M. L.; Haddad, C. F. B. 2015. A new species of *Hylodes* (Anura, Hylodidae) and its secretive underwater breeding behavior. Herpetologica 71: 58─71.

De Witte, G. F. 1930. Liste des reptiles et batraciens récoltés au Brésil par la Mission Massart (1922–23) et description de sept nouvelles espèces. Massart, J. ed., Une Mission Biologique Belge au Brésil (août 1922–mai 1923) par Jean Massart, Raymond Bouillene, Paul Ledoux, Paul Brien, et Albert Navez. Volume 2: 213–230.

Delgado, D. B.; Haddad, C. F. B. 2015. Calling activity and vocal repertoire of *Hypsiboas prasinus* (Anura, Hylidae), a treefrog from the Atlantic Forest of Brazil. Herpetologica 71: 88–95.

Di-Bernardo, M.; Maneyro, R.; Grillo, H. 2006. New species of *Melanophryniscus* (Anura: Bufonidae) from Rio Grande do Sul, southern Brazil. Journal of Herpetology 40: 261–266.

Dias, I. R.; Lourenço-de-Moraes, R.; Solé, M. 2012. Description of the advertisement call and morphometry of *Haddadus binotatus* (Spix, 1824) from a population from southern Bahia, Brazil. North-Western Journal of Zoology 8: 107–111.

Dias, I. R.; Rödder, D.; Weinsheimer, F.; Kwet, A.; Solé, M. 2011. Description of the advertisement call of *Phasmahyla spectabilis* Cruz, Feio & Nascimento, 2008 (Anura: Phyllomedusinae) with comments on its distribution and reproduction. Zootaxa 2767: 59 64.

1174 Dias, P. H. dos S.; Amaro, R. C.; Carvalho-e-Silva, A. M. P. T. de; Rodrigues, M. T.
1175 2013. Two new species of *Proceratophrys* Miranda-Ribeiro, 1920 (Anura;
1176 Odontophrynidae) from the Atlantic forest, with taxonomic remarks on the genus.
1177 Zootaxa 3682: 277–304.

1178 Dias, P. H. dos S.; Hepp, F. S. F. dos S.; Carvalho-e-Silva, A. M. P. T.; Carvalho-e-
1179 Silva, S. P. 2013. Breeding biology and advertisement call of the horned leaf-frog,
1180 *Proceratophrys appendiculata* (Amphibia: Anura: Odontophrynidae). Zoologia. 30:
1181 388–396.

1182 Dixon, J. R.; Staton, M. A. 1976. Some aspects of the biology of *Leptodactylus*
1183 *macrosternum* Miranda-Ribeiro (Anura: Leptodactylidae) of the Venezuelen Llanos.
1184 Herpetology 32: 227─232.

1185 Dixon, J. R.; Mercolli, C.; Yanosky, A. A. 1995. Some aspects of the ecology of
1186 *Pseudis paradoxa* from northeastern Argentina. Herpetological Review 26: 185─186.

1187 Domenico, E. A.; Haddad, C. F. B.; Zaher, H. 2014. Natural history of *Paratelmatobius*
1188 *gaigeae* (Amphibia, Anura, Leptodactylidae): description of the tadpole and
1189 advertisement call. Journal of Herpetology 48(3): 430 433.

1190 Donoso-Barros, R. 1969. Un nuevo anuro de Bolivia, *Eupemphix freibergi* nov. sp.
1191 Boletin de la Sociedad de Biología de Concepción 41: 183–187.

1192 Drummond, L. de O.; Baêta, D.; Pires, M. R. S. 2007. A new species of *Scinax* (Anura,
1193 Hylidae) of the S. ruber clade from Minas Gerais, Brazil. Zootaxa 1612: 45–63.

1194 Duellman, W. E.; Crump, M. L. 1974. Speciation in frogs of the *Hyla parviceps* group
1195 in the upper Amazon Basin. Occasional Papers of the Museum of Natural History 23:
1196 1–40.

1197 Duellman, W. E.; Gray, P. 1983. Developmental biology and systematics of the egg-
1198 brooding hylid frogs, genera *Flectonotus* and *Fritziana*. Herpetologica 39(4): 333–359.

1199 Duellman, W. E.; Hoogmoed, M. S. 1992. Some hylid frogs from the Guiana
1200 Highlands, northeastern South America: New species, distributional records, and a
1201 generic reallocation. Occasional Papers of the Museum of Natural History, University
1202 of Kansas 147: 1–21.

1203 Duellman, W. E.; Lescure, J. 1973. Life history and ecology of the hylid frog
1204 *Osteocephalus taurinus*, with observations on larval behavior. Occasional Papers of the
1205 Museum of Natural History 13: 1 12.

1206 Duellman, W. E.; Lizana, M. 1994. Biology of a sit-and-wait predator, the leptodactylid
1207 frog *Ceratophrys ornate*. Herpetologica 50: 51─64.

1208 Duellman, W. E.; Lynch, J. D. 1969. Descriptions of Atelopus tadpoles and their
1209 relevance to atelopodid classification. Herpetologica 25: 231─240.

1210 Duellman, W. E.; Mendelson, III, J. R. 1995. Amphibians and reptiles from northern
1211 Departamento Loreto, Peru: Taxonomy and biogeography. University of Kansas
1212 Science Bulletin 55: 329–376.

1213 Duellman, W. E.; Morales, V. R. 1990. Variation, distribution, and life history of
1214 *Edalorhina perezi* (Amphibia, Anura, Leptodactylidae). Studies on Neotropical Fauna
1215 and Environment 25(1): 19 30.

1216 Duellman, W. E.; Pyles, R. A. 1983. Acoustic resource partitioning in anuran
1217 communities. Copeia 3: 639 649.

1218 Duellman, W. E.; Sá, R. O. 1988. A new genus and species of South American hylid
1219 frog with a highly modified tadpole. Tropical Zoology 1: 117–136.

1220 Duellman, W. E.; Trueb, L. 1989. Two new treefrogs of the *Hyla parviceps* group from
1221 the Amazon Basin in southern Peru. Herpetologica 45: 1–10.

1222 Duellman, W. E.; Veloso A. 1977. Phylogeny of *Pleurodema* (Anura: Leptodactylidae):
1223 A biogeographic model. Occasional Papers of the Museum of Natural History 64: 1─46.

1224 Duellman, W. E.; Wiens, J. J. 1993. Hylid frogs of the genus *Scinax* Wagler, 1830, in
1225 Amazonian Ecuador and Peru. Occasional Papers of the Museum of Natural History
1226 153: 1–57.

1227 Duellman, W. E. 1970. The hylids frogs of Middle America. Monograph of the
1228 Museum of Natural History 1:1-753.

1229 Duellman, W. E. 1972a. A new species of *Hyla* from Amazonian Ecuador. Copeia
1230 1972: 265–271.

1231 Duellman, W. E. 1972b. South American frogs of the *Hyla rostrata* group (Amphibia,
1232 Anura, Hylidae). Zoologische Mededelingen 47: 177–192.

1233 Duellman, W. E. 1972c. The systematic status and life history of *Hyla rhodopepla*
1234 Günther. Herpetologica 28: 369–375.

1235 Duellman, W. E. 1973. Frogs of the *Hyla geographica* Group. Copeia 3: 515 33.

1236 Duellman, W. E. 1974. A reassessment of the taxonomic status of some neotropical
1237 hylid frogs. Occasional Papers of the Museum of Natural History 27: 1–27.

1238 Duellman, W. E. 1978a. The biology of an equatorial herpetofauna in Amazonian
1239 Ecuador. Miscellaneous Publications of the University of Kansas 65: 1 352.

1240 Duellman, W. E. 1978b. Three new species of *Eleutherodactylus* from Amazonian Perú
1241 (Amphibia: Anura: Leptodactylidae). Herpetologica 34: 264–270.

1242 Duellman, W. E. 1986. Two new species of *Ololygon* (Anura: Hylidae) from the
1243 Venezuelan Guyana. Copeia 1986: 864–870.

1244 Duellman, W. E. 1997. Amphibians of La Escalera region, southeastern Venezuela:
1245 Taxonomy, ecology, and biogeography. Scientific Papers, Natural History Museum 2:
1246 1–52.

1247 Duellman, W. E. 2005. Cusco Amazonico, The lives of amphibians and reptiles in an
1248 Amazonian rainforest. New York: Comstock Publishing Associates - Cornell University
1249 Press. 433 p.

1250 Duellman, W. E.; Cadle, J. E.; Cannatella, D. C. 1988. A new species of terrestrial
1251 *Phyllomedusa* (Anura: Hylidae) from southern Peru. Herpetologica 44: 91–95.

1252 Duméril, A. M. C.; Bibron, G. 1840. Plate 13. d'Orbigny, A., and G. Bibron eds.,
1253 Preprint of plate from "Voyage dans l'Amérique Méridionale (Le Brésil, La République
1254 Orientale de l'Uruguay, La République Argentine, La Patagonie, La Republique du
1255 Chili, La Republique de Bolivia, La République du Pérou), exécuté pendant las annees
1256 1826, 1827, 1828, 1829, 1830, 1831, 1832 et 1833. volume 5, Part 1": Paris and
1257 Strasbourg, M. le Ministre de l'Instruction Publique.

1258 Duméril, A. M. C.; Bibron, G. 1841. Erpétologie Générale ou Histoire Naturelle
1259 Complète des Reptiles. Volume 8. Paris: Librarie Enclyclopedique de Roret.

1260 Dunn, E. R. 1949. Notes on South American frogs of the family Microhylidae.
1261 American Museum Novitates 1419: 1–21.

1262 Duré, I. M.; Schaefer, E. F.; Hamman, M. I.; Kehr, A. I. 2004. Consideraciones
1263 ecológicas sobre la dieta, reproducción y parasitismo de Pseudopaludicola boliviana
1264 (Anura, Leptodactylidae) de Corrientes, Argentina. Phyllomedusa 3(2): 121 131.

1265 Edwards, S. R. 1974. Taxonomic notes on South American dendrobatid frogs of the
1266 genus *Colostethus*. Occasional Papers of the Museum of Natural History, University of
1267 Kansas 30: 1─14.

1268 Emerson, S. B. 1988. The giant tadpole of *Pseudis paradoxa*. Biological Journal of the
1269 Linnean Society 34: 93─104.

1270 Eterovick, P. C.; Brandão, R. A. 2001. A description of the tadpoles and advertisement
1271 calls of members of the *Hyla pseudopseudis* Group. Journal of Herpetology 35:
1272 442─450.

1273 Eterovick, P. C.; Sazima, I. 1998. New species of *Proceratophrys* (Anura:
1274 Leptodactylidae) from southeastern Brazil. Copeia 1998: 159–164.

1275 Eterovick, P. C.; Sazima, I. 1999. Description of the tadpole of *Bufo rufus* with notes on
1276 its aggregative behavior. Journal of Herpetology 23: 711─713.

1277 Eterovick, P. C.;. Sazima, I. 2000. Description of the tadpole of *Leptodactylus syphax*,
1278 with a comparison of morphological and ecological characters of tadpoles and adults of

1279 the species in the *L. pentadactylus* group (Anura, Leptodactylidae). Amphibia-Reptilia
1280 21: 341─350.

1281 Eterovick, P. C.; Barros, I. S.; Sazima, I. 2002. Tadpoles of two species in the *Hyla*
1282 *polytaeni*a species Group and comparison with other tadpoles of *Hyla polytaenia* and
1283 *Hyla pulchella* Groups (Anura, Hylidae). Journal of Herpetology 36: 512─515.

1284 Fabrezi, M.; Vera, R. 1997. Caracterización morfológica de larvas de anuros del
1285 Noroeste Argentino. Cuadernos de Herpetología 11: 37─49.

1286 Fabrezi, M.; Quinzio, S. I.; Goldberg, J. 2009. Giant tadpole and delayed
1287 metamorphosis of *Pseudis platensis* Gallardo, 1961 (Anura, Hylidae). Journal of
1288 Herpetology 43(2): 228─243.

1289 Faivovich, J.; Gasparini, J. L.; Haddad, C. F. B. 2010. A New Species of the *Scinax*
1290 *perpusillus* Group (Anura: Hylidae) from Espírito Santo, Brazil. Copeia 2010: 97–102.

1291 Faivovich, J. 1996. La larva de *Hyla semiguttata* A. Lutz, 1925 (Anura, Hylidae).
1292 Cuadernos de Herpetología 9: 61–67.

1293 Faivovich, J. 1998. Comments on the larvae of Argentine species of the genus
1294 *Crossodactylus* (Leptodactylidae, Hylodinae). Alytes 16: 61─67.

1295 Faivovich, J. 2005. A new species of *Scinax* (Anura: Hylidae) from Misiones,
1296 Argentina. Herpetologica 61: 69–77.

1297 Faivovich, J.; Moravec, J.; Cisneros-Heredia, D. F.; Köhler, J. 2006. A new species of
1298 the *Hypsiboas benitezi* group from the western Amazon Basin (Amphibia: Anura:
1299 Hylidae). Herpetologica 62: 96–108.

1300 Faivovich, J., L. Lugli; Lourenço, A. C. C.; Haddad, C. F. B. 2009. A new species of
1301 the *Bokermannohyla martinsi* group from central Bahia, Brazil with comments on
1302 *Bokermannohyla* (Anura: Hylidae). Herpetologica 65: 303–310.

1303 Faria, D. C. D., Signorelli, L.; Morais, A. R.; Bastos, R. P.; Maciel, N. M. 2013.
1304 Geographic structure and acoustic variation in populations of *Scinax squalirostris* (A.
1305 Lutz, 1925) (Anura: Hylidae). North-Western Journal of Zoology 9: 329 336.

1306 Fatorelli, P.; Costa, P. N.; Laia, R. C.; Almeida-Santos, M.; Van Sluys, M.; Rocha, C. F.
1307 D. 2010. Description, microhabitat and temporal distribution of the tadpole of
1308 *Proceratophrys tupinamba* Prado and Pombal, 2008. Zootaxa 2684: 57–62.

1309 Fehlberg, B. H. B.; Natali, F.; Pezzuti, T. L.; Garcia, P. C. A. 2012. The tadpole of
1310 *Rhinella abei* (Baldissera, Caramaschi, and Haddad, 2004). Zootaxa 3559: 37–3.

1311 Feio, R. N.; Pombal, Jr., J. P.; Caramaschi, U. 1999. New *Physalaemus* (Anura:
1312 Leptodactylidae) from the Atlantic forest of Minas Gerais, Brazil. Copeia 1999: 141–
1313 143.

1314 Fernández, K.; Fernández, M. 1921. Sobre la biología y reproducción de algunos
1315 batrachios argentinos I. Cystignathidae. Annales Sociedade Científica Argentina 91:
1316 97─140.

1317 Fernández, K. M. 1921. Sobre la biologia y reproduction de algunos batracios
1318 argentinos. I Cystignathidae. Anales de la Sociedad Científica Argentina 91: 97─139.

1319 Fernández, K. M. 1927. Sobre la biología y reproducción de batracios argentinos.
1320 Segunda Parte. Boletín de la Academia Nacional de Ciencias 29: 271–290.

1321 Ferrante L.; Angulo A.; Sacramento M. 2014. Notes on range extension and geographic
1322 variation of calls in *Adenomera thomei* (Anura: Leptodactylidae). Check List 10(6):
1323 1560–1562.

1324 Ferrerira, C. M. M.; Sugai, J. L. M. M.; Souza, F. L.; Bastos, R. P.; Vaz-Silva, W.;
1325 Andrade, S. P.; Morais, A. R. 2016. The advertisement call and geographic distribution
1326 of *Proceratophrys dibernardoi* Brandão, Caramaschi, Vaz-Silva & Campos 2013
1327 (Anura, Odontophrynidae). Zootaxa 4205: 480─482.

1328 Fitzinger, L. J. F. J. 1826. Neue Classification der Reptilien nach ihren Natürlichen
1329 Verwandtschaften nebst einer Verwandtschafts-Tafel und einem Verzeichnisse der
1330 Reptilien-Sammlung des K. K. Zoologisch Museum's zu Wien. Wien: J. G. Heubner.

1331 Flores, G.; Rodríguez, L. O. 1997. Two new species of the *Eleutherodactylus*
1332 *conspicillatus* group (Anura: Leptodactylidae) from Perú. Copeia 1997: 388–394.

1333 Fonseca, E. M. da; Lanna, F. M.; Sant'Anna, A. C.; Pereira, E. A.; Santos, F. F.; Neves,
1334 M. de O.; Mângia, S. 2012. The advertisement call of *Elachistocleis*
1335 *helianneae* Caramaschi, 2010 (Anura: Microhylidae). Zootaxa 3559: 58–60.

1336 Ford, P. R.; Scott, N. J. 1996. Descriptions of *Bufo* tadpoles from the southwestern coast
1337 of Jalisco, Mexico. Journal of Herpetology 30: 253–257.

1338 Forlani, M. C.; Mendes, C. V. de M.; Dias, I. R.; Ruas, D. S.; Tonini, J. F. R.; Sá, R. O.
1339 de. 2013. The advertisement calls and distribution of two sympatric species of
1340 *Chiasmocleis* (Méhely 1904) (Anura, Microhylidae, Gastrophryninae) from the Atlantic
1341 Forest. South American Journal of Herpetology 8: 46 51.

1342 Forti, L.R.; Castanho, L.M. 2012. Behavioural repertoire and a new geographical record
1343 of the torrent frog *Hylodes cardosoi* (Anura: Hylodidae). Herpetological Bulletin 121:
1344 17–22.

1345 Forti, L. R.; Strüssmann, C.; Mott, T. 2010. Acoustic communication and vocalization
1346 microhabitat in *Ameerega braccata* (Steindachner, 1864) (Anura, Dendrobatidae) from
1347 Midwestern Brazil. Brazilian Journal of Biology 70(1): 211 216.

1348 Forti, L. R.; Martins, F. A. M.; Bertoluci, J. 2012. Advertisement call and geographical
1349 variation in call features of *Dendropsophus berthalutzae* (Anura: Hylidae) from the
1350 Atlantic Rainforest of southeastern Brazil. Zootaxa 3310: 66–68.

1351 Forti, L. R.; Márquez, R.; Bertoluci, J. 2015. Advertisement call of *Dendropsophus*
1352 *microps* (Anura: Hylidae) from two populations from southeastern Brazil. Zoologia
1353 32: 187–194.

1354 Forti, L. R.; Costa, W. P.; Martins, L. B.; Nunes-De-Almeida, C. H. L.; Toledo, L. F.
1355 2016. Advertisement call and genetic structure conservatism: good news for an
1356 endangered Neotropical frog. PEERJ 4, p. e2014.

1357 Fouquet, A.; Gaucher, P.; Blanc, M.; Vélez-Rodriguez, C. M. 2007. Description of two
1358 new species of *Rhinella* (Anura: Bufonidae) from the lowlands of the Guiana Shield.
1359 Zootaxa 1663: 17–32.

1360 Fouquet, A.; Orrico, V. G. D.; Ernst, R.; Blanc, M.; Martinez, Q.; Vacher, J.-P.;
1361 Rodrigues, M. T.; Ouboter, P. E.; Jairam, R.; Ron, S. R. 2015a. A new *Dendropsophus*
1362 Fitzinger, 1843 (Anura: Hylidae) of the parviceps group from the lowlands of the
1363 Guiana Shield. Zootaxa 4052: 39–64.

1364 Fouquet, A.; Souza, S. M.; Nunes, P. M. S.; Kok, P. J. R.; Curcio, F. F.; Carvalho, C.
1365 M. de; Grant, T.; Rodrigues, M. T. 2015b. Two new endangered species of
1366 *Anomaloglossus* (Anura: Aromobatidae) from Roraima State, northern Brazil. Zootaxa
1367 3926: 191–210.

1368 Fouquet, A.; Martinez, Q.; Zeidler, L.; Courtois, E. A.; Gaucher, P.; Blanc, M.; Lima, J.
1369 D.; Souza, S. M.; Rodrigues, M. T.; Lima, J. D.; Souza, S. M.; Rodrigues, M. T.; Kok,
1370 P. J. R. 2016. Cryptic diversity in the *Hypsiboas semilineatus* species group (Amphibia,
1371 Anura) with the description of a new species from the eastern Guiana Shield. Zootaxa
1372 4084: 79–104.

1373 Fouquette, M. J., Jr., and W. F. Pyburn. 1972. A new Colombian treefrog of the *Hyla*
1374 *rubra* complex. Herpetologica 28: 176–181.

1375 Fouquette, M. J.; Jr. 1960. Call structure in frogs of the family Leptodactylidae. Texas
1376 Journal of Science: 12(3/4): 201 215

1377 Francioni, E.; Carcerelli, L. C. 1993. Descrição do girino de *Crossodactylus*
1378 *gaudichaudii* Duméril and Bibron, 1841 (Anura, Leptodactylidae). Memórias do
1379 Instituto Butantan 55: 63─67.

1380 Fouquette, M. J., Jr.; Pyburn, W. F. 1972. A new Colombian treefrog of the *Hyla rubra*
1381 complex. Herpetologica 28: 176–181.

1382 Franz, I.; Mello, M. H. 2015. *Fritziana* aff. *fissilis* (Miranda-Ribeiro, 1920) (Anura,
1383 Hemiphractidae): the first hemiphractid for the state of Rio Grande do Sul, southern
1384 Brazil. Check List 11: 1–4.

1385 Funk, W. C.; Angulo, A.; Caldwell, J. P.; Ryan, M. J.; Cannatella, D. C. 2008.
1386 Comparison of morphology and calls of two cryptic species of *Physalaemus* (Anura:
1387 Leiuperidae). Herpetologica 64: 290–304.

1388 Funkhouser, A. 1957. A review of the neotropical tree-frogs of the genus *Phyllomedusa*.
1389 Occasional Papers of the Natural History Museum of Stanford University 5: 1–90.

1390 Souza, R. F.; Santos, S. P.; Dias, T. M.; Bastos, R. P.; Nomura, F. 2016. Vocal
1391 Repertoire during reproductive and aggressive contexts of three Brazilian tree frogs:
1392 *Bokermanohyla sapiranga*, *Hypsiboas albopunctatus* and *Hypsiboas goianus* (Anura:
1393 Hylidae). South American Journal of Herpetology 11: 136─147.

1394 Gaiga, R.; Loiola, C.; Mângia, S.; Pirani, R. M. 2013. Advertisement call and tadpoles
1395 of *Bokermannohyla vulcaniae* (Vasconcelos and Giaretta, 2003) (Amphibia: Anura:
1396 Hylidae). South American Journal of Herpetology 8: 127–131.

1397 Gaige, H. T. 1926. A new frog from British Guiana. Occasional Papers of the Museum
1398 of Zoology, University of Michigan 176: 1–3.

1399 Gaige, H. T. 1929. Three new tree-frogs from Panama and Bolivia. Occasional Papers
1400 of the Museum of Zoology, University of Michigan 207: 1–6.

1401 Gallardo, J. M. 1957. Las subespecies argentinas de *Bufo granulosus* Spix. Revista del
1402 Museo Argentino de Ciencias Naturales "Bernardino Rivadavia". Zoología 3: 337–374.

1403 Gallardo, J. M. 1961a. Anfibios anuros de Misiones con la descripcion de una nueva
1404 especie de *Crossodactylus*. Neotropica. La Plata 7: 33–38.

1405 Gallardo, J. M. 1961b. On the species of Pseudidae (Amphibia, Anura). Bulletin of the
1406 Museum of Comparative Zoology. Cambridge, Massachusetts 125: 111– 134.

1407 Gallardo, J. M. 1964. Una nueva forma de Pseudidae (Amphibia, Anura) y algunos
1408 consideraciones sobre las especies Argentinas de esta familia. Acta Zoologica Lilloana
1409 20: 193–209.

1410 Gallardo, J. M. 1965. The species *Bufo granulosus* Spix (Salientia: Bufonidae) and its
1411 geographic variation. Bulletin of the Museum of Comparative Zoology. Cambridge,
1412 Massachusetts 134: 107–138.

1413 Gambale, P. G.; Bastos, R.P. 2014. Vocal repertoire and bioacoustic analyses in
1414 *Physalaemus cuvieri* (Anura, Leptodactylidae) in southern Brazil. Herpetological
1415 Journal 24: 31.

1416 Gambale, P. G.; Signorelli, L.; Bastos, R. P. 2014. Individual variation in the
1417 advertisement calls of a Neotropical treefrog (*Scinax constrictus*). Amphibia-Reptilia
1418 35: 271 281.

1419 Garcia, P. C. A.; Haddad, C. F. B. 2008. Vocalizations and comments on the
1420 relationships of *Hypsiboas ericae* (Amphibia, Hylidae). Iheringia 98(1): 161 166.

1421 Garcia, P. C. A. 1996. Nova especie de *Eleutherodactylus* Dumeril & Bibron, 1891 do
1422 estado de Santa Catarina, Brasil (Amphibia: Anura: Leptodactylidae). Biociencias.
1423 Porto Alegre 4: 57–68.

1424 Garcia, P. C. A.; Caramaschi, U.; Kwet, A. 2001a. O status taxonômico de *Hyla*
1425 *cochranae* Mertens e caracterização de *Aplastodiscus* A. Lutz (Anura, Hylidae). Revista
1426 Brasileira de Zoologia 18: 1197─1218.

Garcia, P. C. A.; Vinciprova, G.; Haddad, C. F. B. 2001b. Vocalização, girinos, distribuição geográfica e novos comentários sobre *Hyla marginata* Boulenger, 1887 (Anura, Hylidae, Hylinae). Boletim do Museo Nacional 460: 1─19.

Garcia, P. C. A.; Vinciprova, G.; Haddad, C. F. B. 2003. The taxonomic status of *Hyla pulchella joaquini* (Anura: Hylidae) with description of its tadpole and vocalization. Herpetologica 59: 350–363.

Garcia, P. C. A.; Faivovich, J.; Haddad, C. F. B. 2007. Redescription of *Hypsiboas semiguttatus*, with the description of a new species of the *Hypsiboas pulchellus* group. Copeia 2007: 933–951.

Garcia, P. C. A.; Peixoto, O. L.; Haddad, C. F. B. 2008. A new species of *Hypsiboas* (Anura: Hylidae) from the Atlantic Forest of Santa Catarina, southern Brazil, with comments on its conservation status. South American Journal of Herpetology 3: 27–35.

Garcia, P. C. A.; Berneck, B. V. M.; Costa, C. O. R. 2009. A new species of *Paratelmatobius* (Amphibia, Anura, Leptodactylidae) from Atlantic Rain Forest of southeastern Brazil. South American Journal of Herpetology 4: 217–224.

Garda, A. A.; Pedro, V. de A. S.; Lion, M. B. 2010. The advertisement and release calls of *Rhinella jimi* (Anura, Bufonidae). South American Journal of Herpetology 5(2): 151 156.

Garey, M. V.; Lima, A. M. X.; Hartmann, M. T.; Haddad, C. F. B. 2012. A new npecies of miniaturized yoadlet, genus *Brachycephalus* (Anura: Brachycephalidae), from southern Brazil. Herpetologica 68: 266–271.

1448 Garey, M. V.; Costa, T. R. N.; Lima, A. M. X.; Toledo, L. F.; Hartmann, M. T. 2012.
1449 Advertisement call of *Scinax littoralis* and *S. angrensis* (Amphibia: Anura: Hylidae),
1450 with notes on the reproductive activity of S. littoralis. Acta Herpetologica 7: 297–308.

1451 Garman, S. 1883. A species of Pseudis from the Rio Arassuahy, Brazil. Science
1452 Observer: A Journal for Scientists 4: 47.

1453 Garman, S. 1888. West Indian Batrachia in the Museum of Comparative Zoology.
1454 Bulletin of the Essex Institute. Salem, Massachusetts 19: 13–16.

1455 Garrido-Yrigaray, R. R. 1989. Descripción de la larva de *Melanophysniscus stelzneri*
1456 *montevidensis* (Phili, 1902) (Anura: Bufonidae) Boletim de la Sociedade Zoologia del
1457 Uruguay 5: 7─8.

1458 Gascon, C. 1989. The tadpole of *Atelopus pulcher* Boulenger (Anura, Bufonidae) from
1459 Manaus, Amazonas. Revista Brasileira de Zoologia 6: 235─239.

1460 Giaretta, A. A.; Aguiar, Jr., O. 1998. A new species of *Megaelosia* from the
1461 Mantiqueira Range, southeastern Brazil. Journal of Herpetology 32: 80–83.

1462 Giaretta, A. A.; Cardoso, A. J. 1995. Reproductive behavior of *Cycloramphus dubius*
1463 Miranda-Ribeiro (Amphibia, Anura, Leptodactylidae). Revista Brasileira de Zoologia
1464 12(2): 233 237.

1465 Giaretta, A. A.; Castanho, L. M. 1990. Nova especie de *Paratelmatobius* (Amphibia,
1466 Anura, Leptodactylidae) da Serra do Mar, Brasil. Papeis Avulsos de Zoologia 37: 133–
1467 139.

Giaretta, A. A.; Costa, H. C. M. 2007. A redescription of *Leptodactylus jolyi* Sazima and Bokermann (Anura, Leptodactylidae) and the recognition of a new closely related species. Zootaxa 1608: 1–10.

Giaretta, A. A.; Kokubum, M. N. C. 2003. A new species of *Pseudopaludicola* (Anura, Leptodactylidae) from northern Brazil. Zootaxa 383: 1–8.

Giaretta, A. A.; Kokubum, M. N. C. 2004. Reproductive ecology of *Leptodactylus furnarius* Sazima & Bokermann, 1978 a frog that lay eggs in underground chambers. Herpetozoa 16(3/4): 115–126.

Giaretta, A. A.; Magrini, L. 2013. Calls of *Paratelmatobius gaigeae* (Cochran 1938) (Anura, Leptodactylidae). Herpetology Notes 6: 171 176.

Giaretta, A. A.; Martins, L. 2009. Notes on the call and behavior of *Arcovomer passarellii* (Anura: Microhylidae). Herpetology Notes 2: 91 93.

Giaretta, A. A.; Menin, M. 2004. Reproduction, phenology and mortality sources of a species of *Physalaemus* (Anura: Leptodactylidae). Journal of Natural History 38: 1711–1722.

Giaretta, A. A.; Sawaya, R. J. 1998. Second species of *Psyllophryne* (Anura: Brachycephalidae). Copeia 1998: 985–987.

Giaretta, A. A.; Sazima, I. 1993. Nova espécie de *Proceratophrys* Mir. Rib. do sul de Minas Gerais, Brasil (Amphibia, Anura, Leptodactylidae). Revista Brasileira de Biologia 53: 13–19.

1488 Giaretta, A. A.; Bokermann, W. C. A.; Haddad, C. F. B. 1993. A review of the genus
1489 *Megaelosia* (Anura: Leptodactylidae) with a description of a new species. Journal of
1490 Herpetology 27: 276–285.

1491 Giaretta, A. A.; Bernarde, P. S.; Kokubum, M. C. N. 2000. A new species of
1492 *Proceratophrys* (Anura: Leptodactylidae) form the Amazon Rain Forest. Journal of
1493 Herpetology 34: 173─178.

1494 Giaretta, A. A.; Toffoli, D.; Oliveira, L. E. 2007. A new species of *Ischnocnema*
1495 (Anura: Eleutherodactylinae) from open areas of the Cerrado Biome in southeastern
1496 Brazil. Zootaxa 1666: 53–51.

1497 Giaretta, A. A.; Oliveira, Filho, J. C.; Kokubum, M. N. C. 2007. A
1498 new *Phyllomedusa* Wagler (Anura, Hylidae) with reticulated pattern on flanks from
1499 southeastern Brazil. Zootaxa 1614: 31–41.

1500 Giaretta, A. A.; Martins, L. B.; Santos, M. P. 2009. Further notes on the taxonomy of
1501 four species of *Physalaemus* (Anura, Leiuperidae) from the Atlantic Forest of
1502 southeastern Brazil. Zootaxa 2266: 51–60.

1503 Giaretta, A. A.; Andrade, F. S.; Haga, I. A.; Bernardes, C. S. 2013. On the
1504 advertisement call of *Dermatonotus muelleri* (Boettger, 1885) (Anura, Microhylidae).
1505 Zootaxa 3700: 593–596.

1506 Giaretta, A. A.; Herche, P. Vo, J.; Tang, J. N.; Gridi-Papp, M. 2015. Reinterpreting
1507 features of the advertisement call of *Dermatonotus muelleri* (Boettger, 1885; Anura,
1508 Microhylidae) . Zootaxa 3972: 595–598.

1509 Giaretta, A.; Brandão, T. F.; Martins, L. B. 2016 . On the advertisement call of
1510 *Bokermannohyla oxente* Lugli and Haddad, 2006 (Anura, Hylidae). Neotropical
1511 Biodiversity 2: 159─162.

1512 Giasson, L. O. M.; Haddad, C. F. B. 2006. Social Interactions in *Hypsiboas*
1513 *albomarginatus* (Anura: Hylidae) and the significance of acoustic and visual signals.
1514 Journal of Herpetology 40(2): 171 180.

1515 Girard, C. 1853. Descriptions of new species of reptiles, collected by the U.S. Exploring
1516 Expedition, under the command of Capt. Charles Wilkes, U.S.N. Second part—
1517 including the species of batrachians, exotic to North America. Proceedings of the
1518 Academy of Natural Sciences of Philadelphia 6: 420–424.

1519 Godinho, L. B.; Moura, M. R.; Lacerda, J. V. A.; Feio, R. N. 2013. A new species of
1520 *Proceratophrys* (Anura: Odontophrynidae) from the middle São Francisco River,
1521 southeastern Brazil. Salamandra 49(2): 63–73.

1522 Goeldi, E. A. 1907. Description of *Hyla resinifictrix* Goeldi, a new Amazonian tree frog
1523 peculiar for its breeding habits. Proceedings of the Zoological Society of London 1907:
1524 135–140.

1525 Goin, C. J. 1957. Status of the frog genus *Sphoenohyla* with a synopsis of the species.
1526 Caldasia. Bogotá 8: 11–31.

1527 Goin, C. J. 1959. A synonym and a homonym in the frog genus *Hyla*. Copeia 1959:
1528 340–341.

1529 Goin, C. J. 1960. Description of a new frog of the genus *Hyla* from northwestern Brazil.
1530 Annals and Magazine of Natural History, Series 13, 2: 721–724.

1531 Goin, C. J. 1966. Description of a new frog of the genus *Hyla* from Suriname.
1532 Zoologische Mededelingen. Leiden 41: 229–232.

1533 Gomes, F. B. R.; Martins, I. A. 2006. Amphibia, Anura, Hylidae, *Dendropsophus*
1534 *anceps* (Lutz, 1929): filling gap, geographic distribution map and vocalization. Check
1535 List 2(3): 22–25.

1536 Gomes, M. R.; Peixoto, O. L. 1991a. Larvas de *Hyla* do grupo "leucophyllata" com a
1537 descrição da de *H. elegans* Wied, 1824 e notas sobre a variação do pradrão de colorido
1538 do adulto nesta espécie (Anura, Hylidae). Revista Brasileira de Biologia 51: 257─262.

1539 Gomes, M. R.; Peixoto, O. L. 1991b. Considerações sobre os girinos de *Hyla senicula*
1540 (Cope, 1868) e *Hyla soaresi* (Caramaschi e Jim, 1983) (Amphibia, Anura, Hylidae).
1541 Acta Biologica Leopoldensia 13: 5─18.

1542 Gomes, M. R.; Peixoto, O. L. 1996. Nova especie de *Hyla* do group marmorata de
1543 Sergipe, nordeste do Brasil (Amphibia, Anura, Hylidae). Iheringia. Série Zoologia 80: 3
1544 3–38.

1545 Gomes, M. R.; Peixoto, O. L. 2002. O girino de *Hyla leucopygia* Cruz and Peixoto,
1546 1984 (Amphibia, Anura, Hylidae). Boletim do Museu de Biologia Mello Leitão 13:
1547 17─25.

1548 Gomes, F. B. R.; Provete, D. B.; Martins, I. A. 2010. The tadpole of *Physalaemus*
1549 *jordanensis* Bokermann, 1967 (Anura, Leiuperidae) from Campos do Jordão, Serra da
1550 Mantiqueira, Southeastern Brazil. Zootaxa 2327: 65─68.

1551 Gomes, J. R.; Ferreira, C. P.; Weber, L. N. 2011. The tadpole of *Physalaemus angrensis*
1552 Weber, Gonzaga and Carvalho-e-Silva, 2005 (Amphibia, Anura, Leiperidae). South
1553 American Journal of Herpetology 6(3): 229─233.

1554 Gomes, F. B. R.; Provete, D. B.; Martins, I. A. 2012. Description of the tadpole of
1555 *Hylodes magalhaesi* (Bokermann, 1964) (Anura: Hylodidae). Journal of Herpetology
1556 46(4): 614─619.

1557 Gomes, M. R.; Alves, A. C. R.; Peixoto, O. L. 2014. The tadpole of *Scinax nebulosus*
1558 (Amphibia, Anura, Hylidae). Iheringia 104(2): 184─188.

1559 Gordo, M.; Toledo, L. F.; Suárez, P.; Kawashita-Ribeiro, R. A.; Ávila, R. W.; Morais,
1560 D. H.; Nunes, I. 2013. A new species of Milk Frog of the genus *Trachycephalus*
1561 Tschudi (Anura, Hylidae) from the Amazonian rainforest. Herpetologica 69: 466–479.

1562 Gouvêa, É. 1979. Uma nova espécie de elosiineo da Serra do Itatiaia (Amphibia, Anura,
1563 Leptodactylidae). Revista Brasileira de Biologia 39: 855–859.

1564 Greding, Jr., E. J. 1976. Call of the tropical American frog *Rana palmipes* Spix
1565 (Amphibia, Anura, Ranidae). Journal of Herpetology 10(3): 263 264.

1566 Griffiths, I.; Carvalho, A. L. 1965. On the validity of employing larval characters as
1567 major phyletic indices in Amphibia, Salientia. Revista Brasileira de Biologia 25:
1568 113─121.

1569 Grillitsch, B. 1992. Notes on the tadpole of *Phrynohyas resinifictrix* (Goeldi, 1907).
1570 Buccopharyngeal and external morphology of a tree hole dwelling larva (Anura,
1571 Hylidae). Herpetozoa 5: 51─66.

1572 Grosso, J. R. 2015. Tadpole morphology of *Leptodactylus plaumanni* (Anura:
1573 Leptodactylidae), with comments on the phylogenetic significance of larval characters
1574 in *Leptodactylus*. Cuadernos de Herpetología 29(2): 117─129.

1575 Guayasamin, J. M.; Ron, S. R.; Cisneros-Heredia, D. F.; Lamar, W. W.; McCracken, S.
1576 F. 2006. A new species of frog of the *Eleutherodactylus lacrimosus* assemblage
1577 (Leptodactylidae) from the western Amazon Basin, with comments on the utility of
1578 canopy surveys in lowland rainforest. Herpetologica 62: 191–202.

1579 Guérin-Méneville, F.-É. 1838. Iconographie du Règne Animal de G. Cuvier ou
1580 Représentation d'Après Nature de l'une des Espèces les plus Remarquables et Souvent
1581 non Envore Figurees, de Chaque Genre d'Animaux, avec un Texte Descriptiff mis au
1582 Courant de La Science. Volume 3 (Part—Reptiles). Paris: J. B. Ballière.

1583 Guerra, M. A.; Ron, S. R. 2008. Mate choice and courtship signal differentiation
1584 promotes speciation in an Amazonian frog. Behavioral Ecology 19(6): 1128 1135.

1585 Guerra, C.; Baldo, D.; Rosset, S.; Borteiro, C.; Kolenc, F. 2011. Advertisement and
1586 release calls in Neotropical toas of the *Rhinella granulosa* group and evidence of natural
1587 hybridization between *R. bergi* and *R. ma*jor (Anura: Bufonidae). Zootaxa 3092: 26 42.

1588 Guimarães, L. D. A.; Bastos, R. P. 2003. Vocalizações e interações acústicas em *Hyla*
1589 *raniceps* (Anura, Hylidae) durante a atividade reprodutiva. Iheringia 93(2): 149 158.

1590 Guimarães, L. D. A.; Lima, L. P.; Juliano, R. F.; Bastos, R. P. 2001. Vocalizações de
1591 espécies de anuros (Amphibia) no Brasil central. Boletim do Museu Nacional 474:
1592 1 14.

1593 Guimarães, C. da S.; Lacerda, J. V. A.; Feio, R. N. 2013. Advertisement call of
1594 *Zachaenus carvalhoi* Izecksohn, 1982 (Anura: Cycloramphidae) from southeastern
1595 Brazil. Zootaxa 3718: 398–400.

1596 Guimarães, C. S.; Peixoto, M. A. A.; Lacerda, J. V. A.; Feio, R. N. 2014. The tadpole of
1597 *Scinax cosenzai* (Anura: Hylidae). Salamandra 50(2): 99–104.

1598 Günther, A. C. L. G. 1858. Neue Batrachier in der Sammlung des britischen Museums.
1599 Archiv für Naturgeschichte. Berlin 24: 319–328.

1600 Günther, A. C. L. G. 1859. Catalogue of the Batrachia Salientia in the Collection of the
1601 British Museum. London: Taylor and Francis.

1602 Günther, A. C. L. G. 1869. First account of species of tailless batrachians added to the
1603 collection of the British Museum. Proceedings of the Zoological Society of London
1604 1868: 478–490.

1605 Günther, A. C. L. G. 1873. Contribution to our knowledge of *Ceratophrys* and
1606 *Megalophrys*. Annals and Magazine of Natural History, Series 4, 11: 417–419.

1607 Haddad, C. F. B.; Cardoso, A. J. 1987. Taxonomia de três especies de Pseudopaludicola
1608 (Anura, Leptodactylidae). Papeis Avulsos de Zoologia 36(24): 287 300.

1609 Haddad, C. F. B.; Martins, M. 1994. Four species of Brazilian poison frogs related to
1610 *Epipedobates pictus* (Dendrobatidae): Taxonomy and natural history. Herpetologica 50:
1611 282–295.

1612 Haddad, C. F. B.; Pombal, Jr., J. P. 1987. *Hyla hiemalis*, nova espécie do grupo rizibilis
1613 do Estado de São Paulo (Amphibia, Anura, Hylidae). Revista Brasileira de Biologia 47:
1614 127–132.

Haddad, C. F. B.; Pombal, Jr., J. P. 1995. A new species of *Hylodes* from southeastern Brazil (Amphibia: Leptodactylidae). Herpetologica 51: 279–286.

Haddad, C. F. B.; Pombal, Jr., J. P. 1998. Redescription of *Physalaemus* spiniger (Anura: Leptodactylidae) and description of two new reproductive modes. Journal of Herpetology 32: 557─565.

Haddad, C. F. B.; Sazima, I. 1989. A new species of *Cycloramphus* from southeastern Brazil (Amphibia: Leptodactylidae). Herpetologica 45: 425–429.

Haddad, C. F. B.; Sazima, I. 2004. A new species of *Physalaemus* (Amphibia; Leptodactylidae) from the Atlantic forest in southeastern Brazil. Zootaxa 479: 1─12.

Haddad, C. F. B.; Andrade, G. V.; Cardoso, A. J. 1988. Anfíbios anuros no Parque Nacional da Serra da Canastra, estado de Minas Gerais. Brasil Florestal 64: 9 20.

Haddad, C. F. B.; Pombal, Jr., J. P.; Batistic, R. F. 1994. Natural hybridization between diploid and tetraploid species of leaf-frogs, genus *Phyllomedusa* (Amphibia). Journal of Herpetology 28(4): 425 430.

Haddad, C. F. B.; Pombal, Jr., J. P.; Bastos, R. P. 1996. New species of *Hylodes* from the Atlantic forest of Brazil (Amphibia: Leptodactylidae). Copeia 1996: 965–969.

Haddad, C. F. B.; Giaretta, A. A.. 1999. Visual and acoustic communication in the Brazilian torrent frog, *Hylodes asper* (Anura: Leptodactylidae). Herpetologica 55(3): 324–333.

Haddad, C. F. B.; Garcia, P. C. A.; Pombal Jr., J. P. 2003. Redescrição de *Hylodes perplicatus* (Miranda-Ribeiro, 1926) (Amphibia, Anura, Leptodactylidae). Arquivos do Museu Nacional 61: 245─254.

1637 Haddad, C. F. B.; Faivovich, J.; Garcia, P. C. A. 2005. The specialized reproductive
1638 mode of the treefrog *Aplastodiscus perviridis* (Anura : Hylidae). Amphibia-Reptilia 26
1639 (2005): 87 92.

1640 Haddad, C. F. B.; Alves, A. C. R.; Clemente-Carvalho, R. B. G.; Reis, S. F. dos. 2010.
1641 A new species of *Brachycephalus* from the Atlantic Rain Forest in São Paulo state,
1642 southeastern Brazil (Amphibia: Anura: Brachycephalidae). Copeia 2010: 410–420.

1643 Haga, I. A.; Andrade, F. S.; Toscano, N. P.; Kwet, A.; Giaretta, A. A. 2014.
1644 Advertisement call and habitat of *Vitreorana uranoscopa* (Anura: Centrolenidae) in
1645 Brazil. Salamandra 50(4): 236 240.

1646 Hartmann, M.T.; Hartmann, P.; Haddad, C. F. B. 2002. Advertisement calls of
1647 *Chiasmocleis carvalhoi*, *Chiasmocleis mehelyi*, and *Myersiella microps*. Journal of
1648 Herpetology 36: 509 511.

1649 Hartmann, M. T.; Hartmann, P. A.; Haddad, C. F. B. 2004. Visual signaling and
1650 reproductive biology in a nocturnal treefrog, genus *Hyla* (Anura: Hylidae). Amphibia
1651 Reptilia 25: 395 406.

1652 Hartmann, M. T.; Hartmann, P. A.; Haddad, C. F. B. 2006. Repertório vocal de *Hylodes*
1653 *phyllodes* (Amphibia, Anura, Hylodidae). Papeis Avulsos de Zoologia 46(17): 203 209.

1654 Harvey, M. B.; Noonan, B. P. 2005. Bolivian glass frogs (Anura: Centrolenidae) with a
1655 descrition of a new species from Amazonia. Proceedings of the Biological Society of
1656 Washington 118: 428–441.

Heatwole, H.; Solano, H.; Heatwole, A. 1965. Notes on amphibians from the Venezuelan Guayanas with description of two new forms. Acta Biologica Venezuelica 4: 349─364.

Hedges, S. B.; Schlüter, A. 1992. *Eleutherodactylus eurydactylus*, a new species of frog from central Amazonian Perú (Anura: Leptodactylidae). Copeia 1992: 1002–1006.

Henle, K. 1991. *Ololygon pedromedinae* sp. nov., ein neuer Knickzehenlaubfrosch (Hylidae) aus Peru. Salamandra 27: 76–82.

Hensel, R. 1867. Beiträge zur Kenntnis der Wirbelthiere Südbrasilens. Archiv für Naturgeschichte. Berlin 33: 120–162.

Hepp, F. S. F. S.; Canedo, C. 2013. Advertisement and aggressive calls of *Ischnocnema oea* (Heyer, 1984) (Anura, Brachycephalidae). Zootaxa 3710: 197–199.

Hepp, F. S. F. S.; Carvalho-e-Silva, S. P. 2011. Description and comparison of advertisement calls of *Euparkerella brasiliensis* (Parker, 1926) and E. cochrane Izecksohn, 1998 (Amphibia: Anura: Strabomantidae). Herpetology Notes 4: 45 51.

Hepp, F. S. F. S.; Luna-Dias, C.; Neto, L. P. G.; Carvalho-e-Silva, S. P. 2012. Redescription of the advertisement call of *Dendropsophus seniculus* (Cope, 1868) and the consequences for the acoustic traits of the *Dendropsophus marmoratus* species group (Amphibia: Anura: Dendropsophini). South American Journal of Herpetology 7: 165–171.

Hepp, F. S. F. S.; Carvalho-e-Silva, S. P.; Carvalho-e-Silva, M. P. T.; Folly, M. 2015. A fifth species of the genus Euparkerella (Griffths, 1959), the advertisement calls of *E. robusta* Izecksohn, 1988 and E. tridactyla Izecksohn, 1988, and a key for the

Euparkerella species (Anura: Brachycephaloidea: Craugastoridae) . Zootaxa 3973: 251–270.

Hero, J.M.; Mijares-Urrutia, A. 1995. The tadpole of *Scinax rostrata*. Journal of Herpetology 29(2): 307─311.

Hero, J. M. 1990. An ilustrated key to tadpoles occuring in Central Amazon rainforest, Manaus, Amazonas, Brasil. Amazoniana 11: 201─262.

Heyer, W. R.; Barrio-Amorós, C. L. 2009. The advertisement calls of two sympatric frogs, *Leptodactylus lithonaetes* (Amphibia: Anura: Leptodactylidae) and *Pristimantis vilarsi* (Amphibia: Anura: Strabomantidae). Proceedings of the Biological Society of Washington 122(3): 282 291.

Heyer, W. R.; Cocroft, R. B. 1986. Descriptions of two new species of *Hylodes* from the Atlantic forests of Brazil (Amphibia: Leptodactylidae). Proceedings of the Biological Society of Washington 99: 100–109.

Heyer, W. R.; Crombie R. I. 1979. Natural history notes on *Craspedoglossa stejnegeri* and *Thoropa petropolitana* (Amphibia: Salientia, Leptodactylidae). Journal of the Washington Academy of Sciences 69: 17─20.

Heyer, W. R.; Crombie, R. I. 2005. *Leptodactylus lauramiriamae*, a distinctive new species of frog (Amphibia: Anura: Leptodactylidae) from Rondônia, Brazil. Proceedings of the Biological Society of Washington 118: 590–595.

Heyer, W. R.; Gascon, C. 1995. Collection notes and call characteristics for *Ischnocnema quixensis* and *Phyzelaphryne miriamae* (Amphibia: Anura; Leptodactylidae). Journal of Herpetology 29: 304–307.

1701 Heyer, W. R.; Giaretta, A. A. 2009. Advertisement calls, notes on natural history, and
1702 distribution of *Leptodactylus chaquensis* (Amphibia: Anura: Leptodactylidae) in Brasil.
1703 Proceedings of the Biological Society of Washington 122(3): 292 305.

1704 Heyer, W. R.; Hardy, L. M. 1991. A new species of frog of the *Eleutherodactylus*
1705 *lacrimosus* assembly from Amazonia, South America (Amphibia: Anura:
1706 Leptodactylidae). Proceedings of the Biological Society of Washington 104: 436–447.

1707 Heyer, W. R.; Heyer, M. M. 2002. *Leptodactylus elenae*. Catalogue of American
1708 Amphibians and Reptiles 742: 1–5.

1709 Heyer, W. R.; Heyer, M. M. 2004. *Leptodactylus furnarius*. Catalogue of American
1710 Amphibians and Reptiles 785: 1–5.

1711 Heyer, W. R.; Heyer, M. M. 2006a. *Leptodactylus knudseni*. Catalogue of American
1712 Amphibians and Reptiles 807: 1–12.

1713 Heyer, W. R.; Heyer, M. M. 2006b. *Leptodactylus natalensis*. Catalogue of American
1714 Amphibians and Reptiles 808: 1 5.

1715 Heyer, W. R.; Juncá, F. A. 2003. *Leptodactylus caatingae*, a new species of frog from
1716 eastern Brazil (Amphibia: Anura: Leptodactylidae). Proceedings of the Biological
1717 Society of Washington 116: 317–329.

1718 Heyer, W. R.; Mello, C. C. 1979. Descriptions of the advertising calls of *Cycloramphus*
1719 *asper* and *Cycloramphus dubius* (Amphibia: Leptodactylidae). Papéis Avulsos de
1720 Zoologia 32(15): 193 200.

1721 Heyer, W. R.; Morato-de-Carvalho, C. 2000. The enigmatic advertisement call of
1722 *Eleutherodactylus ramagii* (Amphibia: Anura: Leptodactylidae). Amphibia-Reptilia
1723 21(1): 117 121.

1724 Heyer, W. R.; Muñoz, A. M. 1999. Validation of *Eleutherodactylus crepitans*
1725 Bokermann, 1965, notes on the types and type locality of *Telatrema heterodactylum*
1726 Miranda-Ribeiro, 1937, and a description of a new species of *Eleutherodactylus* from
1727 Mato Grosso, Brazil (Amphibia: Anura: Leptodactylidae). Proceedings of the Biological
1728 Society of Washington 112: 1–18.

1729 Heyer, W. R.; Pyburn, W. F. 1983. *Leptodactylus riveroi*, a new frog species from
1730 Amazonia, South America. (Anura: Leptodactylidae). Proceedings of the Biological
1731 Society of Washington 96: 560–566.

1732 Heyer, W. R.; Reid, Y. R. 2003. Does advertisement call variation coincide with genetic
1733 variation in the genetically diverse frog taxon currently known as *Leptodactylus fuscus*
1734 (Amphibia: Leptodactylidae)? Anais da Academia Brasileira de Ciências 75(1): 39 54.

1735 Heyer, W. R.; Sá, R. O. 2011. Variation, systematics, and relationships of the
1736 *Leptodactylus bolivianus* complex (Amphibia: Anura: Leptodactylidae). Smithsonian
1737 Contributions to Zoology 635: i–vii, 1–58.

1738 Heyer, W. R.; Silverstone, P. A. 1969. The larva of *Leptodactylus hylaedactylus*
1739 (Amphibia, Leptodactylidae). Fieldiana: Zoology 51: 141─145.

1740 Heyer, W. R.; Thompson, A. S. 2000. *Leptodactylus rugosus*. Catalogue of American
1741 Amphibians and Reptiles 708: 1–5.

1742 Heyer, W. R.; Wolf, A. J. 1989. *Physalaemus crombiei* (Amphibia: Leptodactylidae), a
1743 new frog species from Espírito Santo, Brazil, with comment on the *P. signifer* group.
1744 Proceedings of the Biological Society of Washington 102: 500–506.

1745 Heyer, W. R. 1969. Studies on frogs of the genus *Leptodactylus* (Amphibia,
1746 Leptodactylidae).V. Taxonomic notes on *L. latinasus*, *rhodonotus*, *romani*, and
1747 *wuchereri*. Herpetologica 25: 1─8.

1748 Heyer, W. R. 1970. Studies on the genus *Leptodactylus* (Amphibia, Leptodactylidae). II
1749 Diagnosis and distribution of the *Leptodactylus* of Costa Rica. Revista de Biología
1750 Tropical 16: 171─205.

1751 Heyer, W. R. 1972. The status of *Leptodactylus pumilio* Boulenger (Amphibia,
1752 Leptodactylidae) and the description of a new species of *Leptodactylus* from Ecuador.
1753 Contributions in Science. Natural History Museum of Los Angeles County 231: 1–8.

1754 Heyer, W. R. 1973. Systematics of the marmoratus group of the frog genus
1755 *Leptodactylus* (Amphibia, Leptodactylidae). Contributions in Science. Natural History
1756 Museum of Los Angeles County 251: 1–50.

1757 Heyer, W. R. 1977. Taxonomie notes on frogs from the Madeira and Purus rivers,
1758 Brasil. Papeis Avulsos de Zoologia 31(8): 141 162,.

1759 Heyer, W. R. 1978. Systematics of the fuscus group of the frog genus *Leptodactylus*
1760 (Amphibia, Leptodactylidae). Bulletin of the Natural History Museum of Los Angeles
1761 County 29: 1–85.

1762 Heyer, W. R. 1979. Systematics of the pentadactylus species group of the frog genus
1763 *Leptodactylus* (Amphibia: Leptodactylidae). Smithsonian Contributions to Zoology
1764 301: 1–43.

1765 Heyer, W. R. 1980. The calls and taxonomic positions of *Hyla giesleri* and *Ololygon*
1766 *opalina* (Amphibia: Anura: Hylidae). Proceedings of the Biological Society of
1767 Washington 93: 655–661.

1768 Heyer, W. R. 1982. Two new species of the frog genus *Hylodes* from Caparaó, Minas
1769 Gerais, Brasil (Amphibia: Leptodactylidae). Proceedings of the Biological Society of
1770 Washington 95: 377–385.

1771 Heyer, W. R. 1983a. Clarification of the names *Rana mystacea* Spix, 1824,
1772 *Leptodactylus amazonicus* Heyer, 1978, and a description of a new species,
1773 *Leptodactylus spixi* (Amphibia: Leptodactylidae). Proceedings of the Biological Society
1774 of Washington 96: 270–272.

1775 Heyer, W. R. 1983b. Notes on the frog genus *Cycloramphus* (Amphibia:
1776 Leptodactylidae), with descriptions of two new species. Proceedings of the Biological
1777 Society of Washington 96: 548–559.

1778 Heyer, W. R. 1983c. Variation and systematics of frogs of the genus *Cycloramphus*
1779 (Amphibia, Leptodactylidae). Arquivos de Zoologia 30: 235–339.

1780 Heyer, W. R. 1984. Variation, systematics, and zoogeography of *Eleutherodactylus*
1781 *guentheri* and closely related species (Amhibia: Anura: Leptodactylidae). Smithsonian
1782 Contributions to Zoology 402: 1–42.

1783 Heyer, W. R. 1985a. New species of frogs from Boracéia, São Paulo, Brazil.
1784 Proceedings of the Biological Society of Washington 98: 657–671.

1785 Heyer, W. R. 1985b. Taxonomic and natural history notes on frogs of the genus
1786 *Centrolenella* (Amphibia: Centrolenidae) from southeastern Brazil and adjacent
1787 Argentina. Papéis Avulsos de Zoologia 36: 1─21.

1788 Heyer, W. R. 1988. A notable collection of *Cycloramphus* (Amphibia: Leptodactylidae)
1789 from Bahia, Brazil, with a description of a new species (Cycloramphus migueli).
1790 Proceedings of the Biological Society of Washington 101: 151–154.

1791 Heyer, W. R. 1994. Variation within the *Leptodactylus podicipinus–wagneri* complex
1792 of frogs (Amphibia: Leptodactylidae). Smithsonian Contributions to Zoology 546: 1–
1793 124.

1794 Heyer, W. R. 1995. South American rocky habitat *Leptodactylus* (Amphibia: Anura:
1795 Leptodactylidae) with description of two new species. Proceedings of the Biological
1796 Society of Washington 108: 695–716.

1797 Heyer, W. R. 1998. The relationships of *Leptodactylus diedrus* (Anura,
1798 Leptodactylidae). Alytes 16: 1 24.

1799 Heyer, W. R. 1999. A new genus and species of frog from Bahia, Brazil (Amphibia:
1800 Anura: Leptodactylidae) with comments on the zoogeography of the Brazilian campos
1801 rupestres. Proceedings of the Biological Society of Washington 112: 19–30.

1802 Heyer, W. R. 2005. Variation and taxonomic clarification of the large species of the
1803 *Leptodactylus pentadactylus* species group (Amphibia: Leptodactylidae) from Middle
1804 America, northern South America, and Amazonia. Arquivos de Zoologia 37: 269–348.

1805 Heyer, R. H. 2006. The advertisement call of *Leptodactylus laticeps* (Amphibia, Anura,
1806 Leptodactylidae): predatory aural luring? Herpetological Natural History 9(2): 189 194.

1807 Heyer, W. R.; Rand, A. S.; Cruz, C. A. G.; Peixoto, O. L.; Nelson, C. E. 1990. Frogs of
1808 Boracéia. Arquivos de Zoologia 31: 231─410.

1809 Heyer, W. R.; García-Lopez, J. M.; Cardoso, A. J. 1996. Advertisement call variation in
1810 the *Leptodactylus mystaceus* species complex (Amphibia: Leptodactylidae) with a
1811 description of a new sibling species. Amphibia-Reptilia 17: 7–31.

1812 Heyer W. R.; Heyer M. M.; Sá R. O. 2008. *Leptodactylus cunicularius*. Catalogue of
1813 American Amphibians and Reptiles 845: 1–5.

1814 Heyer W. R.; Heyer M. M.; Sá, R. O. 2010. *Leptodactylus syphax*. Catalogue of
1815 American Amphibians and Reptiles 868: 1–9.

1816 Hödl, W. 1977. Call Differences and calling site segregation in anuran species from
1817 Central Amazonian Floating Meadows. Oecologia 28: 351 363.

1818 Hödl, W. 1992. Reproductive behavior in the neotropical foam-nesting frog *Pleurodema*
1819 *diplolistris* (Leptodactylidae). Amphibia-Reptilia 13: 263 274.

1820 Hoogmoed, M. S.; Cadle, J. E. 1991. Natural history and distribution of *Agalychnis*
1821 *craspedopus* (Funkhouser, 1957) (Amphibia: Anura: Hylidae). Zoologische
1822 Mededelingen 65: 129–142.

1823 Hoogmoed, M. S.; Lescure, J. 1984. A new genus and two new species of minute
1824 leptodactylid frogs from northern South America, with comments upon *Phyzelaphryne*
1825 (Amphibia: Anura: Leptodactylidae). Zoologische Mededelingen. Leiden 58: 85–115.

Hoogmoed, M. S. 1990. Resurrection of Hyla wavrini Parker (Amphibia: Anura: Hylidae), a gladiator frog from northern South America. Zoologische Mededelingen 64: 71–93.

Hoogmoed, M. S.; Lynch, J. D.; Lescure, J. 1977. A new species of *Eleutherodactylus* from Guiana (Leptodactylidae, Anura). Zoologische Mededelingen. Leiden 51: 33–41.

Hoogmoed, M. S.; Borges, D. M.; Cascon, P. 1994. Three new species of the genus *Adelophryne* (Amphibia: Anura: Leptodactylidae) from northeastern Brazil, with remarks on the other species of the genus. Zoologische Mededelingen. Leiden 68: 271–300.

Iop, S.; Lipinski, V. M.; Madalozzo, B.; Maragno, F. P.; Cechin, S. Z.; Santos, T. G. D. 2015. Re-description of the external morphology of *Phyllomedusa iheringii* Boulenger, 1885 larvae (Anura: Hylidae), with comments on the external morphology of tadpoles of the *P. burmeisteri* group. Acta Herpetologica 10: 67─75.

Izecksohn, E.; Carvalho-e-Silva, S. P. 2008. As espécies de *Gastrotheca* Fitzinger na Serra dos Órgãos, Estado do Rio de Janeiro, Brasil (Amphibia: Anura: Amphignathodontidae). Revista Brasileira de Zoologia 25(1): 100 110.

Izecksohn, E.; Cruz, C. A. G. 1972. Notas sobre os girinos de *Dendrophryniscus leucomystax* Izecksohn e *D. brevipollicatus* Espada (Amphibia, Anura, Bufonidae). Arquivo da Universidade Federal Rural do Rio de Janeiro 2: 63─9.

Izecksohn, E.; Cruz, C. A. G. 1976. Nova espécie de *Phyllomedusinae* do Estado do Espírito Santo, Brasil (Amphibia, Anura, Hylidae). Revista Brasileira de Biologia 36: 257–261.

Izecksohn, E.; Gouvêa, E. 1985. Nova espécie de *Megaelosia*, de Itatiaia, Estado do Rio de Janeiro (Amphibia, Anura, Leptodactylidae). Arquivos da Universidade Federal Rural do Rio de Janeiro 8: 17─22.

Izecksohn, E.; Peixoto, O. L. 1981. Nova espécie de *Proceratophrys*, da hiléia Bahiana, Brasil (Amphibia, Anura, Leptodactylidae). Revista Brasileira de Biologia 41: 19–24.

Izecksohn, E. 1959. Uma nova espécies de "Hylidae" da Baixada Fluminense, Estado do Rio de Janeiro, Brasil. Revista Brasileira de Biologia 19: 259–264.

Izecksohn, E. 1965. Uma nova espécie de *Physalaemus* Fitzinger do Estado do Rio de Janeiro (Amphibia, Anura). Revista Brasileira de Biologia 25: 165–168.

Izecksohn, E. 1968. Nova espécie de *Dendrophryniscus* do Estado do Rio de Janeiro (Amphibia, Salientia). Revista Brasileira de Biologia 28: 357–362.

Izecksohn, E. 1971. Nôvo gênero e nova espécie de Brachycephalidae do Estado do Rio de Janeiro, Brasil (Amphibia, Anura). Boletim do Museu Nacional. Nova Serie, Zoologia. Rio de Janeiro 280: 1–12.

Izecksohn, E. 1976a. Uma nova espécie de *Leptodactylus* do Estado do Rio de Janeiro, Brasil (Amphibia, Anura, Leptodactylidae). Revista Brasileira de Biologia 36: 527–530.

Izecksohn, E. 1976b. Uma nova espécie de *Pipa* do Estado do Amazonas, Brasil (Amphibia, Anura, Pipidae). Revista Brasileira de Biologia 36: 507–510.

Izecksohn, E. 1983. Uma nova especie de *Zachaenus* Cope, do Estado do Espirito Santo, Brasil (Amphibia, Anura, Leptodactylidae). Arquivos de Universidade Federal Rural do Rio de Janeiro 5: 7–11.

1869 Izecksohn, E. 1988. Algumas consideraçoes sôbre o genero *Euparkerella*, coma
1870 discrição de tres novas especies (Amphibia, Anura, Leptodactylidae). Revista Brasileira
1871 de Biologia 48: 59–74.

1872 Izecksohn, E. 1994a. Nova espécie de *Dendrophryniscus* da região Amazônica
1873 (Amphibia, Anura, Bufonidae). Revista Brasileira de Zoologia 10: 407–412.

1874 Izecksohn, E. 1994b. Três novas espécies de *Dendrophryniscus* Jiménez de la Espada
1875 das regiões sudeste e sul do Brasil (Amphibia, Anura, Bufonidae). Revista Brasileira de
1876 Zoologia 10: 473–488.

1877 Izecksohn, E.; Cruz, C. A. G.; Peixoto, O. L. 1979. Notas sobre o girino de
1878 *Proceratophrys boiei* (Weid) (Amphibia, Anura, Leptodactylidae). Revista Brasileira de
1879 Biologia 39: 233─36.

1880 Izecksohn, E.; Cruz, C. A. G.; Peixoto, O. L. 1998. Sobre *Proceratophrys*
1881 *appendiculata* algumas espécies afins (Amphibia: Anura: Leptodactylidae). Revista da
1882 Universidade Rural, Serie Ciencia da Vida 20: 37–54.

1883 Izecksohn, E.; Carvalho-e-Silva, S. P. de; Peixoto, O. L. 2009. Sobre *Gastrotheca*
1884 *fissipes* (Boulenger, 1888), com descrição de uma nova espécie (Amphibia, Anura,
1885 Amphignathodontidae). Arquivos do Museu Nacional. Rio de Janeiro 67: 81–91.

1886 Jansen, M.; Schulze, A. 2012. Molecular, morphology and bioacoustic data suggest
1887 Bolivian distribution of a large species of the *Leptodactylus pentadactylus* group
1888 (Amphibia: Anura: Leptodactylidae). Zootaxa 3307: 35-47.

Jansen, M.; Gonzales-Álvarez, L.; Köhler, G. 2007. New species of *Hydrolaetare* (Anura, Leptodactylidae) from Bolivia with some notes on its natural history. Journal of Herpetology 41: 724–732.

Jim, J.; Caramaschi, U. 1979. Uma nova espécie de *Hyla* da região de Botucatu, São Paulo, Brasil (Amphibia, Anura). Revista Brasileira de Biologia 40: 717–719.

Jim, J.; Caramaschi, U. 1980. Uma nova especie de *Odontophrynus* da região de Botucatu, São Paulo, Brasil (Amphibia, Anura). Revista Brasileira de Biologia 40: 357–360.

Jim, J.; Spirandeli-Cruz, E. F. 1973. Uma nova espécie de *Leptodactylus* da Bahia, Brasil (Amphibia, Anura). Anais da III Jornada Cientifica de Faculdade de Ciencias Medicas e Biologicas de Botucatu : 13.

Jiménez de la Espada, M. 1870. Fauna neotropicalis species quaedam nondum cognitae. Jornal de Sciências, Mathemáticas, Physicas e Naturaes. Lisboa 3: 57–65.

Jiménez de la Espada, M. 1872. Nuevos batrácios Americanos. Anales de la Sociedad Española de Historia Natural. Madrid 1: 84–88.

Jiménez de la Espada, M. 1875. Vertebrados del Viaje al Pacífico Verificado de 1862 a 1865 por una Comisión de Naturalistas Enviada por el Gobierno Español. Batracios. Madrid: A. Miguel Ginesta.

Juncá, F. A.; Altig, R.; Gascon, C. 1994. Breeding biology of *Colostethus stepheni*, a dendrobatid frog with a nontransported nidicolous tadpole. Copeia 1994: 747─750.

1909 Juncá, F. A.; Lugli, L. 2009. Reproductive biology, vocalizations, and tadpole
1910 morphology of *Rupirana cardosoi*, an anuran with uncertain affinities. South American
1911 Journal of Herpetology 4(2): 173 178.

1912 Juncá, F. A.; Nunes, I. 2008. A new species of marsupial frog of the genus *Gastrotheca*
1913 Fitzinger (Anura: Amphignatodontidae) from the state of Bahia, northeastern Brazil.
1914 Zootaxa 1907: 61–68.

1915 Juncá, F. A. 1998. Reproductive biology of *Colostethus stepheni* and *Colostethus*
1916 *marchesianus* (Dendrobatidae), with the description of a new anuran mating behaviour.
1917 Herpetologica 54: 377-387.

1918 Juncá, F. A.; Carneiro M. C. L.; Rodrigues, N. N. 2008. Is a dwarf population of
1919 *Corythomantis greeningi* Boulenger, 1896 (Anura, Hylidae) a new species? Zootaxa
1920 1686: 48─56.

1921 Juncá, F. A.; Camurugi, F.; Mercês, E. A. 2012. The tadpole of *Hypsiboas pombali*
1922 (Caramaschi, Pimenta & Feio, 2004) (Anura, Hylidae). Zootaxa 3184: 64─66.

1923 Juncá, F. A.; Napoli, M. F.; Cedraz, J.; Nunes, I. 2012. Acoustic characteristics of the
1924 advertisement and territorial calls of *Phyllodytes tuberculosus* Bokermann, 1966
1925 (Amphibia: Anura: Hylidae). Zootaxa 3506: 87–88.

1926 Juncá, F. A.; Röhr, D. L.; Lourenço-de-Moraes, R.; Santos, F. J. M.; Protázio, A. S.;
1927 Mercês, E. A.; Solé, M. 2012. Advertisement call of species of the genus *Frostius*
1928 Cannatella 1986 (Anura: Bufonidae). Acta Herpetologica 7: 189–201.

1929 Juncá, F. A.; Napoli, M. F.; Nunes, I.; Mercês, E. de A.; Abreu, R. O. de. 2015. A new
1930 species of the *Scinax ruber* clade (Anura, Hylidae) from the Espinhaço Range,
1931 northeastern Brazil. Herpetologica 71: 299–309.

1932 Jungfer, K. H.; Hödl, W. 2002. A new species of *Osteocephalus* from Ecuador and a
1933 redescription of *O. leprieurii* (Duméril & Bibron, 1841) (Anura: Hylidae). Amphibia-
1934 Reptilia 23: 21–46.

1935 Jungfer, K. H.; Schiesari, L. C. 1995. Description of a Central Amazonian and Guianan
1936 treefrog, genus *Osteocephalus* (Anura, Hylidae), with oophagous tadpoles. Alytes 13:1-
1937 13.

1938 Jungfer, K.-H.; Verdade, V. K.; Faivovich, J.; Rodrigues, M. T. 2016. A new species of
1939 spiny-backed treefrog (*Osteocephalus*) from Central Amazonian Brazil (Amphibia:
1940 Anura: Hylidae). Zootaxa 4114: 171–181.

1941 Kaefer, L. I.; Lima, A. P. 2012. Sexual signals of the Amazonian frog *Allobates*
1942 *paleovarzensis*: geographic variation and stereotypy of acoustic traits. Behaviour 149:
1943 15–33.

1944 Kaefer, I. L.; Erdtmann, L. K.; Lima, A. P. 2011. The advertisement call
1945 of *Physalaemus ephippifer* (Anura: Leiuperidae) from Brazilian Amazonia. Zootaxa
1946 2929: 57 58.

1947 Kaiser, K.; Hammers J. L. 2008. The effect of anthropogenic noise on male
1948 advertisement call rate in the neotropical treefrog, *Dendropsophus triangulum*.
1949 Behaviour 146: 1053 1069.

1950 Kehr, A. I.; Basso, N. G. 1990. Description of tadpole of *Lysapsus limellus* (Anura:
1951 Pseudidae) and some considerations on its biology. Copeia 1990: 573─575.

1952 Kehr, A. I.; Duré, M. I. 1995. Descripción de la larva de *Scinax nasica* (Cope, 1862)
1953 (Anura, Hylidae). Facena 11: 99─103.

1954 Kehr, A. I.; Schaffer, E. F.; Duré, M. I. 2004.The tadpole of *Physalaemus albonotatus*
1955 (Anura: Lepodactylidae). Journal of Herpetology 38: 145─148.

1956 Kenny, J. S. 1969. The Amphibia of Trinidad. Studies of Fauna of Curaçao and
1957 Carribean Islands 29: 1─78.

1958 Kher, A. I.; Schaefer, E. F. 2005. Description of the tadpole of *Pseudopaludicola*
1959 *boliviana* (Anura: Leptodactylidae). Herpetological Review 36: 250─251.

1960 Kime, N. M.; Turnere, W. R.; Ryan, M. J. 2000. The transmission of advertisement
1961 calls in Central American frogs. Behavioral Ecology 11(1): 71 83.

1962 Klappenbach, M. A. 1968. Notas herpetológicas, IV. El genero *Melanophryniscus*
1963 (Amphibia, Salientia) en el Uruguay, con descripción de dos nuevas especies..
1964 Comunicaciones Zoológicas del Museo de Historia Natural de Montevideo 9: 1–17.

1965 Köhler J.; Lötters S. 1999. Advertisement calls of two Bolivian *Leptodactylus*
1966 (Amphibia: Anura: Leptodactylidae). Amphibia-Reptilia 20(2): 215–219.

1967 Köhler, J.; Lötters, S. 1999. Annotated list of amphibian records from the Departamento
1968 Pando, Bolivia, with description of some advertisement calls. Bonner zoologische
1969 Beiträge 48: 259 273

1970 Köhler, J. 2000. Amphibian diversity in Bolivia: a study with special reference to
1971 montane forest regions. Bonner Zoologische Monographien 48: 1–243.

1972 Köhler, J.; Reichle, S.; Bonn, G. P. 1997. Advertisement calls of three species of *Bufo*
1973 (Amphibia: Anura: Bufonidae) from lowland Bolivia. Stuttgarter Beiträge zur
1974 Naturkunde 562: 1–8.

1975 Kok, P. J. R.; Means, D. B.; Bossuyt, F. 2011. A new highland species of *Pristimantis*
1976 Jiménez de la Espada, 1871 (Anura: Strabomantidae) from the Pantepui region,
1977 Northern South America. Zootaxa 2934: 1 19.

1978 Kok, P. J. R.; Willaert, B.; Means, D. B. 2013. A new diagnosis and description of
1979 *Anomaloglossus roraima* (La Marca, 1998) (Anura: Aromobatidae: Anomaloglossinae),
1980 with description of its tadpole and call. South American Journal of Herpetology 8(1):
1981 29─45.

1982 Kokubum, M. N. C.; Maciel, N. M.; Matsushita, R. H.; Queiróz-Júnior, A. T.; Sebben,
1983 A. 2009. Reproductive biology of the Brazilian sibilator frog *Leptodactylus troglodytes*.
1984 The Herpetological Journal 19(3): 119─126.

1985 Kolenc, F.; Borteiro, C.; Tedros, M. 2003. La larva de *Hyla uruguaya* Schmidt, 1944
1986 (Anura: Hylidae), con comentarios sobre su biología en Uruguay y su status
1987 taxonómico. Cuadernos de Herpetologia 17: 87 100.

1988 Kolenc, F.; Borteiro, C.; Tedros, M.; Nunez, D.; Maneyro, R. 2006. The tadpole of
1989 *Physalaemus henselii* (Peters) (Anura: Leiuperidae). Zootaxa 1360: 41─50.

1990 Kolenc, F.; Borteiro, C.; Tedros, M.; Prigioni, C. 2007. The tadpole of *Scinax*
1991 *aromothyella* (Anura: Hylidae) from Uruguay. Studies on Neotropical Fauna and
1992 Environment 42: 175─180.

1993 Kolenc, F.; Borteiro, C.; Alcalde, L.; Baldo, D.; Cardozo, D.; Faivovich, J. 2008b.
1994 Comparative larval morphology of eight species of *Hypsiboas* Wagler (Amphibia,
1995 Anura, Hylidae) from Argentina and Uruguay, with a review of the larvae of this genus.
1996 Zootaxa 1927: 1–66.

1997 Kolenc, F.; Borteiro, C.; Baldo, D.; Ferraro, D. P.; Prigioni, C. M. 2009. The tadpoles
1998 and advertisement calls of *Pleurodema bibroni* Tschudi and Pleurodema
1999 kriegi (Müller), with notes on their geographic distribution and conservation status
2000 (Amphibia, Anura, Leiuperidae). Zootaxa 1969: 1–35.

2001 Krügel, P.; Richter, S. 1995. Syncope antenori: a bromeliad breeding frog with free-
2002 swimming, nonfeeding tadpoles (Anura, Microhylidae). Copeia 1995(4): 955─963.

2003 Kurth, M.; Hörnes, D.; Rödder, D. 2013a. Race against desiccation: rapid larval
2004 development in *Melanophryniscus klappenbachi* (Anura: Bufonidae). Salamandra
2005 50(2): 117─124.

2006 Kurth, M.; Hörnes, D.; Esser, S.; Rödder, D. 2013b. Notes on the acoustic repertoire of
2007 *Melanophryniscus klappenbachi* Prigioni & Langone, 2000 . Zootaxa 3626: 597–600.

2008 Kwet, A.; Ângulo, A. 2002. A new species of *Adenomera* (Anura, Leptodactylidae)
2009 from the Araucaria forest of Rio Grande do Sul (Brazil), with comments on the
2010 systematic status of southern populations of the genus. Alytes 20: 28–43.

2011 Kwet, A.; Baldo, D. 2003. Advertisement call of the leptodactylid frog *Proceratophrys*
2012 *avelinoi*. Amphibia-Reptilia 24(1): 104 107.

Kwet, A.; Di-Bernardo, M. 1998. *Elachistocleis erythrogaster*, a new microhylid species from Rio Grande do Sul, Brazil. Studies on Neotropical Fauna and Environment 33: 7–18.

Kwet, A.; Faivovich, J. 2001. *Proceratophrys bigibbosa* species group (Anura: Leptodactylidae), with description of a new species. Copeia 2001: 203–215.

Kwet, A.; Solé, M. 2008. A new species of *Trachycephalus* (Anura: Hylidae) from the Atlantic Rain Forest in southern Brazil. Zootaxa 1947: 53–67.

Kwet, A. 1999. Biologie von *Phyllomedusa iheringii* und Bemerkungen zu deiser Gattung in Rio Grande do Sul, Sudbrasilien. Salamandra: 19 36.

Kwet, A. 2000. The genus *Pseudis* (Anura: Pseudidae) in Rio Grande do Sul, southern Brazil, with description of a new species. Amphibia-Reptilia 21: 39–55.

Kwet, A. 2001. Frösche im brasilianischen Araukarienwald— Anurengemeinschaft des Araukarienwaldes von Rio Grande do Sul: Diversität, Reproduktion und Ressourcenaufteilung. Natur und Tier-Verlag, Münster, Germany.

Kwet, A. 2007. Bioacoustic variation in the genus *Adenomera* in southern Brazil, with revalidation of Leptodactylus nanus Müller, 1922 (Anura, Leptodactylidae). Zoosystematics and Evolution 83: 56–68.

Kwet, A. 2008. New species of *Hypsiboas* (Anura: Hylidae) in the pulchellus group from southern Brazil. Salamandra 44: 1–14.

Kwet, A.; Di-Bernardo, M.; Garcia, P. C. A. 2001. The taxonomic status of *Leptodactylus geminus* Barrio, 1973. Journal of Herpetology 35: 56 62.

Kwet, A.; Maneyro, R.; Zillikens, A.; Mebs, D. 2005. Advertisement calls of *Melanophryniscus dorsalis* (Mertens, 1933) and *M. montevidensis* (Philippi, 1902), two parapatric species from southern Brazil and Uruguay, with comments on morphological variation in the Melanophryniscus stelzneri group (Anura: Bufonidae). Salamandra 41: 1–18.

Kwet, A.; Steiner, J.; Zillikens, A. 2009. A new species of *Adenomera* (Amphibia: Anura: Leptodactylidae) from the Atlantic rain forest in Santa Catarina, southern Brazil. Studies on Neotropical Fauna and Environment 2009: 1 15.

La Marca, E. 1996. Ranas del genero *Colostethus* (Amphibia: Anura: Dendrobatidae) de la Guayana Venezolana con la descripción de siete especies nuevas.. Publicaciones de la Asociación de Amigos de Doñana. Sevilla 9: 1–64.

Lacerda, J. V. A.; Bilate, M.; Feio, R. N. 2011. Advertisement call of *Sphaenorhynchus mirim* Caramaschi, Almeida and Gasparini, 2009 (Anura: Hylidae). South American Journal of Herpetology 6(3): 211 214.

Lacerda, J. V. A.; Peixoto, O. L.; Feio, R. N. 2012. A new species of the bromeligenous *Scinax perpusillus* group (Anura; Hylidae) from Serra do Brigadeiro, state of Minas Gerais, southeastern Brazil. Zootaxa 3271: 31–42.

Lacerda, J. V. A.; Moura, M. R. 2013. Vocal repertoire of *Sphaenorhynchus palustris* (Anura, Hylidae), with notes on S. botocudo. Salamandra 49: 105 108.

Lacerda, J. V. A.; Ferreira, R. B.; Souza, G. A.; Silva, H. R.; Feio, R. N. 2015. On the diagnosis and conservation of the poorly known bromeligenous *Scinax arduous* Peixoto, 2002 (Amphibia; Anura; Hylidae). Zootaxa 4021: 401–417.

2056 Laia, R. C.; Fatorelli, P.; Hatano, F. H.; Rocha, C. F. D. 2010. Tadpole of *Hylodes fredi*
2057 (Anura; Hylodidae), a frog endemic to an Atlantic Forest island (Ilha Grande, Rio de
2058 Janeiro State), Brazil. Zootaxa 2640: 62–64.

2059 Langone, J. A.; de Sá, R. O. 2005. Redescripción de la morfología larval externa de dos
2060 especies del grupo de *Leptodactylus fuscus* (Anura, Leptodactylidae). Phyllomedusa 4:
2061 49─59.

2062 Langone, J. A.; Segalla, M. V. 1996. Una nueva especie de *Eleutherodactylus* del
2063 Estado de Paraná, Brasil. Comunicaciones Zoológicas del Museo de Historia Natural de
2064 Montevideo 12: 1–5.

2065 Langone, J. A. 1989. Descripción de la larva de *Physalaemus gracilis* (Boulenger,
2066 1883) (Amphibia, Anura, Leptodactylidae). Comunicaciones Zoológicas del Museo de
2067 Historia Natural de Montevideo 12: 1─11.

2068 Langone, J. A.; Lavilla, E. O.; Echeverría, D.; Mangione, S.; Segalla, M. 2007.
2069 Morfología externa e interna de la larva de *Chiasmocleis leucosticta* (Boulenger, 1888)
2070 (Amphibia, Anura, Microhylidae). Publicación extra Museo Nacional de Historia
2071 Natural y Antropologia 2: 1─25.

2072 Langone, J. A.; Segalla, M. V.; Bornschein, M. R.; Sá, R. O. de. 2008. A new
2073 reproductive mode in the genus *Melanophryniscus* Gallardo, 1961 (Anura: Bufonidae)
2074 with description of a new species from the state of Paraná, Brazil. South American
2075 Journal of Herpetology 3: 1–9.

2076 Laurenti, J. N. 1768. Specimen Medicum, Exhibens Synopsin Reptilium Emendatum
2077 cum Experimentis Circa Venena et Antidota Reptilium Austriacorum. Wien, Austria:
2078 Joan. Thom. nob. de Trattnern.

Lavilla, E. O.; Fabrezi, M. 1992. Anatomía craneal de larvas de *Lepidobatrachus llanensis* y *Ceratophrys cranwelli* (Anura: Leptodactylidae). Acta Zoologica Lilloana 1: 5─11.

Lavilla, E. O. 1983. Contribucion ao conocimiento de los estados larvales de anuros argentinos. *Phyllomedusa pailona* Shreve. Acta Zoologica Lilloana 37: 5─13.

Lehr, E.; Moravec, J.; Gagliardi-Urrutia, L. A. G. 2010. A new species of *Pristimantis* (Anura: Strabomantidae) from the Amazonian lowlands of northern Peru. Salamandra 46: 197–203.

Lehr, E.; Catenazzi, A.; Rodríguez, D. 2009. A new species of *Pristimantis* (Anura: Strabomantidae) from the Amazonian lowlands of northern Peru (Region Loreto and San Martín). Zootaxa 1990: 30–40.

Leite, F. S. F.; Eterovick, P. C. 2010. Description of the tadpole of *Bokermannohyla martinsi* (Anura: Hylidae), morphological and ecological comparison with related *Bokermannohyla* tadpoles. Journal of Herpetology 44(3): 431─440.

Leite, F. S. F.; Pezzuti, T. L.; Drummond, L. de O. 2011. A new species of *Bokermannohyla* from the Espinhaço range, state of Minas Gerais, southeastern Brazil. Herpetologica 67: 440–448.

Leite, F. S. F.; Pezzuti, T. L.; Garcia, P. C. de A. 2012. A new species fo the *Bokermannohyla pseudopseudis* group from the Espinhaço Range, central Bahia, Brazil (Anura: Hylidae). Herpetologica 68: 401–409.

2099 Lemes, P.; Tessarolo, G.; Morais, A. R.; Bastos, R. P. 2012. Acoustic Repertoire of
2100 *Barycholos ternetzi* (Anura: Strabomantidae) in Central Brazil. South American Journal
2101 of Herpetology 7: 157 164.

2102 Leon, J. R. 1975. Desarrollo temprano y notas sobre la historia natural de la larva de
2103 *Hyla x-signata*. Caribbean Journal of Science 15: 57─65.

2104 Lescano, J. N. 2011. Description of the advertisement and distress call of *Chacophrys*
2105 *pierottii* and comments on the advertisement call of *Lepidobatrachus llanensis* (Anura:
2106 Ceratophrydae). Journal of Natural History 45(47-48): 2929 2938.

2107 Lescure, J.; Marty, C. 2000. Atlas des Amphibiens de Guyane. Collections Patrimoines
2108 Naturels. Paris 45: 1–388.

2109 Lescure, J. 1972. Contribution a l´étude des amphibians de Guyane Française. II.
2110 *Leptodactylus fuscus* (Schneider) observation écologiques et éthologiques. Annales
2111 Musée Histoire Naturelle Nice 1: 91─100.

2112 Lescure, J. 1974. Presence d'une sous-espèce *d'Atelopus pulcher* (Amphibien, Anoure)
2113 dans les Guyanes: *Atelopus pulcher hoogmoedi*. Bulletin du Museum National
2114 d'Histoire Naturelle. Paris. Serie 3, Zoologie 144: 997–1005.

2115 Lescure, J. 1975. Contribution à l'étude des Amphibiens de Guyane française. IV.
2116 Reproduction de *Phyllomedusa tomopterna* (Cope)(Hylidae). Bulletin de la Société
2117 Zoologique de France 100: 117─125.

2118 Lescure, J. 1976. Etude de deux tetards de *Phyllobates* (Dendrobatidae): *P. femoralis*
2119 (Boulenger) et *P. pictus* (Bibron). Bulletin de la Societe Zoologique 101: 299─306.

Lescure, J. 1981b. Contribution a l'etude des Amphibiens de Guyane Francaise. VIII. Validation d'Atelopus spumarius Cope, 1871, et designation d'um neotype. Bulletin du Muséum national d'histoire naturelle 3: 893 910.

Lescure, J. 1981a. Contribution à l'étude des Amphibiens de Guyane Française. IX. Le têtard gastromyzophore d' *Atelopus flavescens* Duméril et Bibron (Anura, Bufonidae). Amphibia-Reptilia 2: 209─215.

Lescure, J.; Marty, V. C.; Starege, F.; Auber-Thomay, M.; Letellier, F. 1995. Contribution à l'étude des Amphibiens de Guyane française. x. Les *Phyllomedusa* (Anura, Hylidae). Revue Francaise D'Aquariologie Herpetologie 22: 35─50.

Lescure, J.; Marty, V.; Marty, C.; Thomay-Auber, M. 1996. Contribution à l'étude des Amphibiens de Guyane française XI. Les *Phrynohyas* (Anura, Hylidae). Revue Francaise D'Aquariologie Herpetologie 23(12): 69─76.

Lichtenstein, H.; Martens, E. v. 1856. Nomenclator Reptilium et Amphibiorum Musei Zoologici Berolinensis. Namenverziechniss der in der zoologischen Sammlung der Königlichen Universität zu Berlin aufgestellten Arten von Reptilien und Amphibien nach ihren Ordnungen, Familien und Gattungen. Berlin.

Lichtenstein, H. 1823. Verzeichniss der Doubletten des zoologischen Museums der Königl. Universität zu Berlin nebst Beschreibung vieler bisher unbekannter Arten von Säugethieren, Vögeln, Amphibien und Fischen. Berlin: T. Trautwein.

Lima, A. P.; Caldwell, J. P. 2001. A new Amazonian species of *Colostethus* with sky blue digits. Herpetologica 57: 133–138.

2141 Lima, A. P. 1992. The tadpole of *Leptodactylus riveroi* Heyer and Pyburn, 1983
2142 (Anura: Leptodactylidae). Journal of Herpetology 26: 91─93.

2143 Lima, A. M. X. 2007. *Proceratophrys avelinoi* Advertisement call. Herpetological
2144 Review 38(2): 191.

2145 Lima, L. P.; Bastos, R. P.; Giaretta, A. A. 2005. A new *Scinax* Wagler, 1830 of the S.
2146 rostratus group from central Brazil (Amphibia, Anura, Hylidae). Arquivos do Museu
2147 Nacional 62: 505–512.

2148 Lima, A. P.; Menin, M.; Araújo, M.C. 2007a. A new species of *Rhinella* (Anura:
2149 Bufonidae) from Brazilian Amazon. Zootaxa 1663: 1─15.

2150 Lima, A. P.; Sanchez, D. E. A.; Souza, J. R. D. 2007b. A New Amazonian species of
2151 the frog genus *Colostethus* (Dendrobatidae) that lays its eggs on undersides of leaves.
2152 Copeia 2007: 114–122.

2153 Lima, M. G.; Lingnau, R.; Skuk, G. O. 2008. The advertisement call of *Phyllodytes*
2154 *edelmoi* (Anura, Hylidae). South American Journal of Herpetology 3(2): 118 121.

2155 Lima, A. P.; Caldwell, J. P.; Strussmann, C. 2009. Redescription of *Allobates brunneus*
2156 (Cope 1887) (Anura: Aromobatidae: Allobatinae), with a description of the tadpole,
2157 call, and reproductive behavior. Zootaxa 1988: 1─16.

2158 Lima, A. P.; Caldwell, J. P.; Biavati, G.; Montanarin, A. 2010b. A new species of
2159 *Allobates* (Anura: Aromobatidae) from Paleovárzea Forest in Amazonas, Brazil.
2160 Zootaxa 2337: 1─17.

2161 Lima, A. M. X.; Garey, M. V.; Noleto, R. B.; Verdade, V. K. 2010a. Natural history of
2162 the Lutz's Frog *Cycloramphus lutzorum* Heyer 1983 (Anura: Cycloramphidae) in the

2163 Brazilian Atlantic Forest: description of the advertisement call, tadpole and karyotype.
2164 Journal of Herpetology 44(3): 360─371.

2165 Lima, M. G. de; Cruz, C. A. G.; Azevedo, Jr., S. M. de. 2011. A new species belonging
2166 to the *Scinax catha*rinae group from the state of Alagoas, northeastern Brazil
2167 (Amphibia, Anura, Hylidae). Boletim do Museu Nacional. Nova Serie, Zoologia. Rio de
2168 Janeiro 529: 1–12.

2169 Lima, A. P.; Erdtmann, L. K.; Amézquita A. 2012. Advertisement call and colour in life
2170 of *Allobates crombiei* (Morales) "2000" [2002] (Anura: Aromobatidae) from the type
2171 Locality (Cachoeira do Espelho), Xingu River, Brazil. Zootaxa 3475: 86–88

2172 Lima, A. P.; Simões, P. I.; Kaefer, Í. L. 2014a. A new species of *Allobates* (Anura:
2173 Aromobatidae) from the Tapajós River basin, Pará State, Brazil. Zootaxa 3889: 355–
2174 387.

2175 Lima, D. C.; Borges-Nojosa, D. M.; Cechin, S. T. Z. 2014b. The advertisement call of
2176 *Adelophryne maranguapensis* (Anura, Eleutherodactylidae) . Zootaxa 3835: 299–300.

2177 Lima, A. P.; Simões, P. I.; Kaefer, Í. L. 2015. A new species of *Allobates* (Anura:
2178 Aromobatidae) from Parque Nacional da Amazônia, Pará State, Brazil. Zootaxa 3980:
2179 501–525.

2180 Lingnau, R.; Bastos, R. P. 2003. Vocalizações de duas espécies de anuros do Sul do
2181 Brasil (Amphibia: Hylidae). Arquivos do Museu Nacional 61(3): 203 207,.

2182 Lingnau, R.; Bastos, R. P. 2007. Vocalizations of the Brazilian torrent frog *Hylodes*
2183 *heyeri* (Anura: Hylodidae): Repertoire and influence of air temperature on
2184 advertisement call variation. Journal of Natural History 41: 1227 1235.

2185 Lingnau, R.; Guimarães, L. D. A.; Bastos, R. P. 2004. Vocalizações de *Hyla werneri*
2186 (Anura, Hylidae) no sul do Brasil. Phyllomedusa 3: 115 120,.

2187 Lingnau, R.; Canedo, C.; Pombal, Jr., J. P. 2008. A new species of *Hylodes* (Anura:
2188 Hylodidae) from the Brazilian Atlantic Forest. Copeia 2008: 595–602.

2189 Lingnau, R.; Solé, M.; Dallacorte, F.; Kwet, A. 2008. Description of the advertisement
2190 call of *Cycloramphus bolitoglossus* (Werner, 1897), with comments on other species in
2191 the genus from Santa Catarina, south Brazil (Amphibia, Cycloramphidae). North-
2192 Western Journal of Zoology 4: 224–235.

2193 Lingnau, R.; Zank, C.; Colombo, P.; Kwet, A. 2013. Vocalization of *Hylodes*
2194 *meridionalis* (Mertens 1927) (Anura, Hylodidae) in Rio Grande do Sul, Brazil, with
2195 comments on nocturnal calling in the family Hylodidae. Studies on Neotropical Fauna
2196 and Environment 48: 76 80.

2197 Linnaeus, C. 1758. Systema Naturae per Regna Tria Naturae, Secundum Classes,
2198 Ordines, Genera, Species, cum Characteribus, Differentiis, Synonymis, Locis. 10th
2199 Edition. Volume 1. Stockholm, Sweden: L. Salvii.

2200 Llusia, D.; Gómez, M.; Penna, M.; Márquez, R. 2013. Call transmission efficiency in
2201 native and invasive anurans: competing hypotheses of divergence in acoustic signals.
2202 PLoS One 8(10): e77312.

2203 Lobo, F. 1991. Descripción de la larva de *Pseudopaludicola mystacalis* (Anura:
2204 Leptodactylidae). Boletín de la Associación Herpetológica Argentina 7: 22─24.

Lobo, F. 1994. Descripción de una nueva especie de *Pseudopaludicola* (Anura: Leptodactylidae), redescripción de *P. falcipes* (Hensel, 1867) y *P. saltica* (Cope, 1887). Cuadernos de Herpetología 8: 177–199.

Lobo, F. 1996. Evaluación del status taxonómico de *Pseudopaludicola ternetzi* Miranda Ribeiro, 1937; *P. mystacalis* y *P. ameghini* (Cope, 1887). Osteología y distribuición de las espécies estudiadas. Acta Zoologica Lilloana 43:327-346.

Loebmann, D.; Zina, J.; Araújo, O. G. S. A.; Toledo, L. F.; Haddad, C. F. B. H. 2008. Acoustic repertory of *Hypsiboas exastis* (Caramaschi and Rodrigues, 2003) (Amphibia, Hylidae). South American Journal of Herpetology 3(2): 96 100.

Lönnberg, E.; Andersson, L. G. 1910. A new lizard and a new frog from Paranà. Arkiv för Zoologi. Stockholm 6(9): 1–11.

López-Rojas, J. J.; Ramalho, W. P.; Susçuarana, M. da S.; Souza, M. B. 2013. Three new records of *Pristimantis* (Amphibia: Anura: Craugastoridae) for Brazil and a comment of the advertisement call of *Pristimantis orcus*. Check List 9: 1548–1551.

Lötters, S.; Reichle, S.; Jungfer, K. H. 2003. Advertisement calls of Neotropical poison frogs (Amphibia: Dendrobatidae) of the genera *Colostethus*, *Dendrobates* and *Epipedobates*, with notes on dendrobatid call classification. Journal of Natural History37: 1899 1911.

Lourenço, A. C. C.; Baêta, D.; Monteiro, V. S.; Pires, M. R. S. 2009. O canto de anúncio de *Scinax luizotavioi* (Caramaschi & Kisteumacher, 1989). Arquivos do Museu Nacional 67: 73 79.

Lourenço, A. C. C.; Nascimento, L. B.; Pires, M. R. S. 2009b. A new species of the *Scinax catharinae* species group (Anura: Hylidae) from Minas Gerais, southeastern Brazil. Herpetologica 65: 468─479.

Lourenço, A. C. C.; Carvalho, A. L. G. de; Baêta, D.; Pezzuti, T. L.; Leite, F. S. F. 2013a. A new species of the *Scinax catharinae* group (Anura, Hylidae) from Serra da Canastra, southwestern state of Minas Gerais, Brazil. Zootaxa 3613: 573–588.

Lourenço, A. C. C.; Luna, M. C.; Pombal, Jr., J. P. 2014. A new species of the *Scinax catharinae* group (Anura: Hylidae) from northeastern Brazil. Zootaxa 3889: 259–276.

Lourenço, A. C. C.; Zina, J.; Catroli, G. F.; Kasahara, S.; Faivovich, J.; Haddad, C. F. B. 2016. A new species of the *Scinax catharinae* group (Anura: Hylidae) from southeastern Brazil. Zootaxa 4154: 415–435.

Lourenço-de-Moraes, R.; Solé, M.; Toledo, L. F. 2012. A new species of *Adelophryne* Hoogmoed and Lescure 1984 (Amphibia: Anura: Eleutherodactylidae) from the Atlantic rainforest of southern Bahia, Brazil. Zootaxa 3441: 59–68.

Lourenço-de-Moraes, R.; Lantyer-Silva, A. S. F.; Toledo, L. F.; Solé, M. 2013. Tadpole, oophagy, advertisement call, and geographic distribution of *Aparasphenodon arapapa* Pimenta, Napoli and Haddad 2009 (Anura, Hylidae). Journal of Herpetology 47(4): 575─579.

Lourenço-de-Moraes, R., R. B. Ferreira, A. Fouquet, and R. P. Bastos. 2014. A new diminutive frog species of *Adelophryne* (Amphibia: Anura: Eleutherodactylidae) from the Atlantic Forest, southeastern Brazil. Zootaxa 3846: 348–360.

Lourenço-de-Morais, R.; Campos, F. S.; Toledo, F. 2012. The tadpole of *Dendropsophus haddadi* (Bastos & Pombal, 1996) (Hylidae: Hylinae). Zootaxa 3476: 86─88.

Lugli, L.; Haddad, C. F. B. 2006a. A new species of the *Bokermannohyla pseudopseudis* group from central Bahia, Brazil (Amphibia, Hylidae). Herpetologica 62: 453–465.

Lugli, L.; Haddad, C. F. B. 2006b. New species of *Bokermannohyla* (Anura, Hylidae) from central Bahia, Brazil. Journal of Herpetology 40: 7–15.

Lutz, B.; Bokermann, W. C. A. 1963. A new tree frog from Santa Catarina, Brazil. Copeia 1963: 558–561.

Lutz, B.; Carvalho, A. L. de. 1958. Novos anfíbios anuros das Serras Costeiras do Brasil. Memórias do Instituto Oswaldo Cruz. Rio de Janeiro 56: 239–249.

Lutz, B.; Kloss, G. R. 1952. Anfíbios anuros do alto Solimões e Rio Negro. Apontamento sôbre algumas formas e suas vicariantes. Memórias do Instituto Oswaldo Cruz. Rio de Janeiro 50: 625–678.

Lutz, A.; Lutz, B. 1938. I. On *Hyla aurantiaca* Daudin and *Sphoenorhynchus* Tschudi and on two allied Hylae from south-eastern Brazil. II. Two new hylae: *H. albosignata* n. sp. & *H. pickeli*. Anais da Academia Brasileira de Ciências. Rio de Janeiro 10: 175–194.

Lutz, A.; Lutz, B. 1939. New Hylidae from Brazil/Hylideos novos do Brasil. Anais da Academia Brasileira de Ciências. Rio de Janeiro 11: 67–89.

Lutz, B.; Orton, G. L. 1946. *Hyla claresignata* Lutz and B. Lutz, 1939. Aspects of its life history and description of the rhyacophilous tadpole. Boletim do Museu Nacional 70: 1─27.

Lutz, A. 1924. Sur les rainettes des environs de Rio de Janeiro. Comptes Rendus et Mémoires Hebdomadaires des Séances de la Société de Biologie et des ses Filiales. Paris 90 (1925, vol. 1): 241.

Lutz, A. 1925a. Batraciens du Brésil. Comptes Rendus et Mémoires Hebdomadaires des Séances de la Société de Biologie et des ses Filiales. Paris 93 (1925, vol. 2): 137–139.

Lutz, A. 1925b. Batraciens du Brésil. Comptes Rendus et Mémoires Hebdomadaires des Séances de la Société de Biologie et des ses Filiales. Paris 93 (1925, vol. 2): 211–214.

Lutz, A. 1926. Observações sobre batrachios brasileiros/Observations on brazilian batrachians. Memórias do Instituto Oswaldo Cruz. Rio de Janeiro 19: 139–174.

Lutz, A. 1929a. Taxonomia e biologia do genero *Cyclorhamphus*/Taxonomy and biology of the genus *Cyclorhamphus*. Memórias do Instituto Oswaldo Cruz. Rio de Janeiro 22: 5–25.

Lutz, A. 1929b. Une nouvelle espèce de *Hyla*. Comptes Rendus et Mémoires Hebdomadaires des Séances de la Société de Biologie et des ses Filiales. Paris 101 (1929, vol. 2): 943.

Lutz, A. 1930a. Contribution to the knowledge of Brazilian batrachians. Taxonomy and biology of the Elosiinae. Memórias do Instituto Oswaldo Cruz 24: 223─249.

Lutz, A. 1930b. Segunda memoria sobre especies brasileiras do genero *Leptodactylus*, incluind outras alliadas/Second paper on Brasilian and some closely related species of the genus *Leptodactylus*. Memórias do Instituto Oswaldo Cruz. Rio de Janeiro 23: 1–34.

Lutz, A. 1934. Notas subre especies brasileiras do genero *Bufo*/ Zur Kenntnis der Brasilianischen Kroeten vom Genus Bufo. Memórias do Instituto Oswaldo Cruz. Rio de Janeiro 28: 111–134 (Portuguese), 135–159 (German).

Lutz, B. 1944. Biologia e taxonomia de *Zachaenus parvulus*. Boletim do Museu Nacional 17: 1─66.

Lutz, B. 1949. Anfibios anuros da coleção Adolpho Lutz. II. Especies verdes do genero *Hyla* do Leste-Meridional do Brasil. Memórias do Instituto Oswaldo Cruz. Rio de Janeiro 46: 551–577.

Lutz, B. 1950. Anfíbios anuros da coleção Adolpho Lutz do Instituto Oswaldo Cruz. V/ Frogs in the Adolpho Lutz collection of the Instituto Oswaldo Cruz. V. Memórias do Instituto Oswaldo Cruz. Rio de Janeiro 48: 599–637.

Lutz, B. 1951. Nota prévia sôbre alguns anfíbios anuros do Alto Itatiaia. O Hospital. Rio de Janeiro 39: 705–707.

Lutz, B. 1954. Anfibios anuros do Distrito Federal/The frogs of the Federal District of Brazil. Memórias do Instituto Oswaldo Cruz. Rio de Janeiro 52: 155–197 (Portuguese), 219–238 (English).

Lutz, B. 1958. Anfibios novos e raros das Serras Costeiras do Brasil (New or rare frogs from the Coastal Ranges of Brazil). Memórias do Instituto Oswaldo Cruz. Rio de Janeiro 56: 372–389 (Portuguese), 389–399 (English).

2310 Lutz, B. 1963. New species of *Hyla* from southeastern Brazil. Copeia 1963: 561–562.

2311 Lutz, B. 1966. *Pithecopus ayeaye*, a new Brazilian hylid with vertical pupils and
2312 grasping feet. Copeia 1966: 236–237.

2313 Lutz, B. 1968a. Geographic variation in Brazilian species of *Hyla*. Pearce-Sellards
2314 Series. Texas Memorial Museum. Austin 12: 1–13.

2315 Lutz, B. 1968b. New Brazilian forms of *Hyla*. Pearce-Sellards Series. Texas Memorial
2316 Museum. Austin 10: 3–18.

2317 Lutz, B. 1973. New Brazilian forms of *Hyla*. I. Two new races of *H. catharinae*.
2318 Boletim do Museu Nacional. Nova Serie, Zoologia. Rio de Janeiro 288: 1–7.

2319 Lutz, B. 1974. *Eleutherodactylus gualteri*, a new species from the Organ Mountains of
2320 Brazil. Journal of Herpetology 8: 293–295.

2321 Lynch, J. D..; Duellman, W. E. 1973. A review of the centrolenid frogs of Ecuador,
2322 with descriptions of new species. Occasional Papers of the Museum of Natural History,
2323 University of Kansas 16: 1–66.

2324 Lynch, J. D.; Hoogmoed, M. S. 1977. Two species of *Eleutherodactylus* (Amphibia:
2325 Leptodactylidae) from northeastern South America. Proceedings of the Biological
2326 Society of Washington 90: 424–439.

2327 Lynch, J. D.; Suárez Mayorga, Á. M. 2011. Clave ilustrada de los renacuajos em las
2328 tierras bajas al oriente de los Andes, com énfasis em Hylidae. Caldasia 33(1): 235─270.

2329 Lynch, J. D. 1968. Two new frogs of the genus *Eleutherodactylus* from eastern Ecuador
2330 (Amphibia: Leptodactylidae). Journal of Herpetology 2: 129–135.

2331 Lynch, J. D. 1974. New species of frogs (Leptodactylidae: Eleutherodactylus) from the
2332 Amazonian lowlands of Ecuador. Occasional Papers of the Museum of Natural History,
2333 University of Kansas 31: 1–22.

2334 Lynch, J. D. 1975. The identity of the frog *Eleutherodactylus conspicillatus* (Günther),
2335 with descriptions of two related species from northwestern South America (Amphibia,
2336 Leptodactylidae). Contributions in Science. Natural History Museum of Los Angeles
2337 County 272: 1–19.

2338 Lynch, J. D. 1976. Two new species of frogs of the genus *Euparkerella* (Amphibia:
2339 Leptodactylidae) from Ecuador and Perú. Herpetologica 32: 48–53.

2340 Lynch, J. D. 1980. A taxonomic and distributional synopsis of the Amazonian frogs of
2341 the genus *Eleutherodactylus*. American Museum Novitates 2696: 1–24.

2342 Lynch, J.D. 2006. The tadpoles of frogs and toads found in the lowlands of northern
2343 Colombia. Revista de la Academia Colombiana de Ciencias 30(116): 443─457.

2344 MacCulloch, R. D.; Lathrop, A.; Kok, P. J. R.; Minter, L. R.; Khan, S. Z.; Barrio-
2345 Amorós, C. 2008. A new species of *Adelophryne* (Anura: Eleutherodactylidae) from
2346 Guyana, with additional data on *A. gutturosa*. Zootaxa 1884: 36–50.

2347 MacCulloch, R. D.; Lathrop, A.; Minter, L. R.; Khan, S. Z. 2015. Otophryne (Anura:
2348 Microhylidae) from the highlands of Guyana: redescriptions, vocalisations, tadpoles and
2349 new distributions. Papeis Avulsos de Zoologia 48(22): 247 261.

2350 Maciel, D. B.; Nunes, I. 2010. A new species of four-eyed frog genus *Pleurodema*
2351 Tschudi, 1838 (Anura: Leiuperidae) from the rock meadows of Espinhaço range, Brazil.
2352 Zootaxa 2640: 53–61.

2353 Maciel, N. M.; Brandão, R. A.; Campos, L. A.; Sebben, A. 2007. A large new species of
2354 *Rhinella* (Anura: Bufonidae) from Cerrado of Brazil. Zootaxa 1627: 23–39.

2355 Maciel, N. M.; Vaz-Silva, W.; Oliveira, R. M.; Padial, J. M. 2012. A new species
2356 of *Pristimantis* (Anura: Strabomantidae) from the Brazilian Cerrado. Zootaxa 3265: 43–
2357 56.

2358 Magalhães, F.M.; Neves, M. O.; Fonseca, E.M.; Carvalho, R.M.H. 2012a. The tadpole
2359 of *Bokermannohyla ibitipoca* (Caramaschi & Feio, 1990) (Anura, Hylidae). Zootaxa
2360 3415: 58–62.

2361 Magalhães, F. D. M.; Santana, D. J.; Neto, A. M.; Garda, A. A. 2012b. The tadpole of
2362 *Elachistocleis cesarii* Miranda-Ribeiro, 1920 (Anura, Microhylidae). Zootaxa 3187: 54–
2363 56.

2364 Magalhães, F. de M.; Loebmann, D.; Kokubum, M. N. de C.; Haddad, C. F. B.; Garda,
2365 A. A. 2014. A new species of *Pseudopaludicola* (Anura: Leptodactylidae: Leiuperinae)
2366 from northeastern Brazil. Herpetologica 70: 77–88.

2367 Magalhães, F. M.; Mercês, E. A.; Santana, D. J.; Juncá, F. A.; Napoli, M. F.; Garda, A.
2368 A. 2015a. The tadpole of *Bokermannohyla flavopicta* Leite, Pezzuti and Garcia, 2012
2369 and oral cavity anatomy of the tadpole of *B. oxente* Lugli and Haddad, 2006 (Anura:
2370 Hylidae). South American Journal of Herpetology 10: 211─218.

2371 Magalhães, F. M.; Juncá, F. A.; Garda, A. A. 2015b. Tadpole and vocalisations of
2372 *Phyllodytes wuchereri* (Anura: Hylidae) from Bahia, Brazil. Salamandra, 51(2): 83─90.

2373 Magrini, L.; Giaretta, A. A. 2010. Calls of two Brazilian species of *Scinax* of the *S.*
2374 *ruber* clade (Anura: Hylidae). Herpetology Notes 3: 121 126.

2375 Magrini, L.; Carvalho-e-Silva, S. P.; Béda, A. F.; Giaretta, A. A. 2011. Calls of five
2376 species of the *Scinax ruber* (Anura: Hylidae) clade from Brazil with comments on their
2377 taxonomy. Zootaxa 3066: 37–51.

2378 Malagoli, L. R.; Mângia, S.; Haddad, C. F. B. 2016. The advertisement call of
2379 *Proceratophrys pombali* (Amphibia: Anura: Odontophrynidae) with comments on its
2380 distribution and natural history. South American Journal of Herpetology 11: 18 24.

2381 Maneyro, R.; Arrieta, D.; Sá, R. O. de. 2004. A new toad (Anura: Bufonidae) from
2382 Uruguay. Journal of Herpetology 38: 161–165.

2383 Maneyro, R.; Núnez, D.; Borteiro, C.; Tedros, M.; Kolenc, F. 2008. Advertisement call
2384 and female sexual cycle in Uruguayan populations of *Physalaemus henselii* (Anura,
2385 Leiuperidae). Iheringia 98(2): 210 214.

2386 Mângia, S.; Santana, D. J.; Feio, R. N. 2010. Advertisement call of the Cycloramphid
2387 Toad *Proceratophrys melanopogon* (Miranda-Ribeiro, 1926). South American Journal
2388 of Herpetology 5: 127–131.

2389 Mângia, S.; Santana, D. J.; Cruz, C. A. G.; Feio, R. N. 2014. Taxonomic review of
2390 *Proceratophrys melanopogon* (Miranda Ribeiro, 1926) with description of four new
2391 species (Amphibia, Anura, Odontophrynidae). Boletim do Museu Nacional 531: 1–33.

2392 Márquez, R.; De La Riva, I.; Bosch, J. 1993. Advertisement calls of Bolivian species of
2393 *Hyla* (Amphibia, Anura, Hylidae). Biotropica 25: 425 443.

2394 Márquez, R.; De La Riva, I.; Bosch, J. 1995. Advertisement calls of Bolivian
2395 Leptodactylidae (Amphibia, Anura). Journal of Zoology 237: 313–336.

Martins, M.; Cardoso, A. J. 1987. Novas especies de hilideos do Estado do Acre (Amphibia: Anura). Revista Brasileira de Biologia 47: 549–558.

Martins, L. B.; Giaretta, A. A. 2011. A new species of *Proceratophrys* Miranda-Ribeiro (Amphibia: Anura: Cycloramphidae) from central Brazil. Zootaxa 2880: 41–50.

Martins, L. B.; Giaretta, A. A. 2012a. Advertisement calls of two species of *Proceratophrys* (Anura: Odontophrynidae) from Minas Gerais, Brazil, with comments on their distribution, taxonomy and conservation status. South American Journal of Herpetology 7(3): 203-212.

Martins, L. B.; Giaretta, A. A. 2012b. *Ameerega flavopicta* (Lutz, 1925): First dart-poison frog (Anura: Dendrobatidae) recorded for the state of São Paulo, Brazil, with comments on its advertisement calls and taxonomy. Check List 8(3): 502 504.

Martins, L. B.; Giaretta, A. A. 2013. Morphological and acoustic characterization of *Proceratophrys goyana* (Lissamphibia: Anura: Odontophrynidae), with the description of a sympatric and related new species. Zootaxa 3750: 301–320.

Martins, M.; Haddad, C. F. B. 1988. Vocalizations and reproductive behaviour in the smith frog, *Hyla faber* Wied (Amphibia: Hylidae). Amphibia-Reptilia 9(1): 49 60,

Martins, I. A.; Haddad, C. F. B. 2010. A new species of *Ischnocnema* from highlands of the Atlantic Forest, Southeastern Brazil (Terrarana, Brachycephalidae). Zootaxa 2617: 55–65.

Martins, I. A.; Jim, J. 2003. Bioacoustic analysis of advertisement call in *Hyla nana* and *Hyla sanborni* (Anura, Hylidae) in Botucatu, São Paulo, Brazil. Brazilian Journal of Biology 63(3): 507 516.

Martins, I. A.; Jim, J. 2004. Advertisement call of *Hyla jimi* and *Hyla elianeae* (Anura, Hylidae) in the Botucatu Region, São Paulo, Brazil. Brazilian Journal of Biology 64: 645 654.

Martins, M.; Moreira, G. 1991. The nest and the tadpole of *Hyla wavrini*, Parker (Amphibia, Anura). Memorias do Instituto Butantan 53: 197–204.

Martins, I. A.; Zaher, H. 2013. A new species of the highland frog genus *Holoaden* (Amphibia, Strabomantidae) from cloud forests of southeastern Brazil. Zootaxa 3599: 178–188.

Martins, M. 1998. Biologia reprodutiva de *Leptodactylus fus*cus em Boa Vista, Roraima (Amphibia: Anura). Revista Brasileira de Biologia 48(4): 969 977.

Martins, M. 1989. Nova especie de *Colostethus* da Amazonia central (Amphibia: Dendrobatidae). Revista Brasileira de Biologia 49: 1009–1012.

Martins, I. A. 2010. Natural history of *Holoaden luederwaldti* (Amphibia: Strabomantidae: Holoadeninae) in southeastern of Brazil. Zoologia 27(1): 40 46.

Martins, I. A.; Almeida, S. C.; Jim, J. 2006. Calling sites and acoustic partitioning in species of the *Hyla nana* and *rubicundula* groups (anura, hylidae). Herpetological Journal 16: 239 247.

Martins, L. B.; Silva, W. R.; Giaretta, A. A. 2009. Distribution and calls of two South American frogs (Anura). Salamandra 45(2): 106 109.

Martins, L. B.; Giaretta, A. A.; Carvalho, T. R.; Miwa, R. Y. 2016. Vocalizations of *Hypsiboas beckeri* and *H. stenocephalus* (Anura: Hylidae), two species of the *H. polytaenius* group from southeastern Brazil. Phyllomedusa 15(1): 51 64.

2440 May, R. V.; Medina–Müller, M.; Donnelly, M. A.; Summers, K. 2008. The tadpole of
2441 the bamboo–breeding poison frog *Ranitomeya biolat* (Anura: Dendrobatidae). Zootaxa
2442 1857: 66─68.

2443 McCracken, S. F.; Forstner, M. R. J. 2006. Reproductive ecology and behavior of
2444 *Eleuterodactylus aureolineatus* (Anura: Brachycephalidae) in the canopy oh the upper
2445 Amazon Basin, Ecuador. Phyllomedusa 5: 135 243.

2446 Melin, D. E. 1941. Contributions to the knowledge of the Amphibia of South America.
2447 Göteborgs Kungl. Vetenskaps-och Vitterhets-samhälles. Handlingar. Serien B,
2448 Matematiska och Naturvetenskapliga Skrifter 1: 1–71.

2449 Melo-Sampaio, P. R.; Souza, M. B.; Peloso, P. L. V. 2013. A new, riparian, species of
2450 *Allobates* Zimmermann and Zimmermann, 1988 (Anura: Aromobatidae) from
2451 southwestern Amazonia . Zootaxa 3716: 336–348.

2452 Mendes, C. V. de M.; Ruas, D. S.; Lourenço-de-Moraes, R.; Rödder, D.; Solé, M. 2012.
2453 The advertisement call of *Gastrotheca fissipes* Boulenger, 1888 (Anura,
2454 Hemiphractidae) with comments on its distribution. Zootaxa 3312: 62–64.

2455 Mendes, C. V.; Junior E. M.; Ruas, D. S.; De Oliveira, R. M.; Solé, M. 2013.
2456 Advertisement call of *Scinax strigilatus* (Spix, 1824) (Anura: Hylidae) from southern
2457 Bahia, Brazil. Zootaxa 3647: 499 500.

2458 Menin, M.; Pegorini, R. J. 2014. The tadpole of *Amazophrynella manaos* Rojas,
2459 Carvalho, Gordo, Ávila, Farias and Hrbek, 2014 (Anura, Bufonidae) from the type
2460 locality and adjacent regions at Central Amazonia, Brazil. Zootaxa 3826(2): 393─396.

Menin, M.; Silva, R. A.; Giaretta, A. A. 2004. Reproductive biology of *Hyla goiana* (Anura, Hylidae). Iheringia 94(1): 49 52.

Menin, M.; Rodrigues D. J.; Lima, A. P. 2006. The tadpole of *Rhinella proboscidea* (Anura: Bufonidae) with notes on adult reproductive behavior. Zootaxa 1258: 47─56.

Menin, M.; Rodrigues, D. J.; Lima, A. P. 2007. Clutches, tadpoles and advertisement calls of *Synapturanus mirandaribeiroi* and *S.* cf. *salseri* in Central Amazonia, Brazil. Herpetological Journal 17: 86 91.

Menin, M.; Lima, A. P.; Rodrigues, D. J. 2009a. The Tadpole of *Vitreorana oyampiensis* (Anura, Centrolenidae) in Central Amazonia, Brazil. Zootaxa 2203: 65─68.

Menin, M.; Almeida, A. P.; Kokubum, M. N. C. 2009b. Reproductive aspects of *Leptodactylus hylaedactylus* (Anura: Leptodactylidae), a member of the *Leptodactylus marmoratus* species group, with a description of tadpoles and calls. Journal of Natural History 43: 2257─2270.

Menin, M.; Lima, A. P.; Rodrigues, D. J. 2010. The tadpole of *Leptodactylus pentadactylus* (Anura: Leptodactylidae) from Central Amazonia. Zootaxa 2508: 65─68.

Menin, M.; Melo, L. d. S.; Lima, A. P. 2011a. The tadpole of *Osteocephalus cabrerai* (Anura: Hylidae) from central Amazonia, Brazil. Phyllomedusa 10: 137─142.

Menin, M.; L. S. Souza; D. J.Rodrigues. 2011a. Tadpole and breeding habitats of *Chiasmocleis shudikarensis* (Anura, Microhylidae) in Central Amazonia, Brazil. South American Journal of Herpetology 6(3): 223─228.

Mercadal de Barrio, I. T. 1986. *Ceratophrys joazeirensis* sp. n. (Ceratophryidae, Anura) del noreste de Brasil. Amphibia-Reptilia 7: 313–334.

Mercadal de Barrio, I. T.; Barrio, A. 1993. Una nueva especies de *Proceratophrys* (Leptodactylidae) del nordeste de Argentina. Amphibia-Reptilia 14: 13–18.

Mercês, E. A.; Juncá, F. A. 2010. Girinos de três espécies de *Aplastodiscus* Lutz, 1950 (Anura-Hylidae) ocorrentes no Estado da Bahia, Brasil. Biota neotropica 10 (4): 167─172.

Mercês, E. A.; Juncá, F. A. 2012. The tadpole of *Scinax juncae* Nunes & Pombal, 2010 (Anura: Hylidae). Zootaxa 3416: 41─43.

Mercês, E. A.; Juncá, F. A.; Casal, F. S. C. 2009. Girinos de três espécies do gênero *Rhinella* Fitzinger, 1826 (Anura: Bufonidae) ocorrentes no estado da Bahia, Brasil. Sitientibus 9: 133─138.

Mercês, E. A.; Protazio, A. S. ; Juncá, F. A. 2011. The tadpole of *Bokermannohyla capra* Napoli & Pimenta 2009 (Anura, Hylidae). Zootaxa 3167: 66─68.

Mercês, E. A.; Camurugi, F.; Barreto, G.S.; Solé, M.; Juncá, F.A. 2015a. The tadpole of *Bokermannohyla lucianae* (Napoli & Pimenta 2003) (Amphibia, Anura, Hylidae). Zootaxa 3904 (2): 298–300.

Mercês, E. A.; Magalhães, F. M.; Amado, T. F.; Juncá, F. A.; Garda, A. A. 2015b. Tadpole of *Leptodactylus oreomantis* Carvalho, Leite & Pezzuti 2013 (Anura, Leptodactylidae). Zootaxa 3911 (4): 589–592.

Mertens, R. 1926. Herpetologischen Mitteilungen VIII–XV. Senckenbergiana Biologica 8: 137–155.

2504 Mertens, R. 1927. Neue Froschlurch aus Rio Grande do Sul, Brasilien. Blätter für
2505 Aquarien- und Terrarien-Kunde. Stuttgart 38: 287–290 [reprint paged 1–3].

2506 Mertens, R. 1933. Über zwei südamerikanische Froschlurche der Gattungen
2507 *Dendrophryniscus* und *Gastrotheca*. Zoologischer Anzeiger 102: 257–260.

2508 Mertens, R. 1937. Ein neuer Frosch der Gattung *Dendrophryniscus* aus Paraguay.
2509 Senckenbergiana Biologica 19: 175–177.

2510 Mertens, R. 1950a. Ein neuer Laubfrosch aus Venezuela. Senckenbergiana Biologica
2511 31: 1–10.

2512 Mertens, R. 1950b. Froschlurche aus Rio de Janeiro und seiner Umgebung.
2513 Wochenschrift für Aquarien- und Terrarienkunde. Braunschweig 44: 173–188.

2514 Mertens, R. 1952. Eine neue *Hyla aus* Santa Catharina, Brasilien. Senckenbergiana
2515 Biologica 33: 165–167.

2516 Mijares-Urrutia, A. 1993. The tadpole of *Hyla granosa* (Anura: Hylidae) from
2517 southeastern Venezuela. Revista Chilena de Historia Natural 66: 143─147.

2518 Milstead, W. W. 1960. Frogs of the genus *Physalaemus* in southern Brazil with the
2519 description of a new species. Copeia 1960: 83–89.

2520 Miranda, N.; Ferreira, 2008. A. Morfologia bucal interna dos girinos de *Leptodactylus*
2521 *labyrinthicus* Spix, 1824 (Amphibia: Anura: Leptodactylidae). Biota Neotropica 8: 1─6.

2522 Miranda, R. B.; Abrunhosa, P. A.; Silva, H. R. 2016. Serenading for ten thousand years:
2523 the mating call of insular populations of the green treefrog *Aplastodiscus eugenioi*
2524 (Anura: Hylidae). Tropical Conservation Science 9: 338 353.

Miranda-Ribeiro, A. 1920a. Algumas consideracões sobre *Holoaden lüderwaldti* e generos correlatos. Revista do Museu Paulista. São Paulo 12: 319–320.

Miranda-Ribeiro, A. 1920b. Algumas consideracoes sobre o genero *Ceratophrys* e suas especies. Revista do Museu Paulista. São Paulo 12: 291–304.

Miranda-Ribeiro, A. 1920c. As *Hyla coelonotas* do Museu Paulista. Revista do Museu Paulista. São Paulo 12: 323–328.

Miranda-Ribeiro, A. 1920d. O genero *Telmatobius* já foi constatado no Brasil? Revista do Museu Paulista. São Paulo 12: 261–278.

Miranda-Ribeiro, A. 1920e. Os Brachycephalideos do Museu Paulista (com tres especes novas). Revista do Museu Paulista. São Paulo 12: 307–316.

Miranda-Ribeiro, A. 1920f. Os engystomatideos do Museu Paulista (com um genero e tres especies novos). Revista do Museu Paulista. São Paulo 12: 281–288.

Miranda-Ribeiro, A. 1920g. Triprion, Diaglena, *Corythomantis*, etc. uma subsecção de Hylidae, com duas especies novas. Revista do Museu Paulista. São Paulo 12: 85–89.

Miranda-Ribeiro, A. 1923a. As *Phyllomesusas* do Museo Paulista. Boletim do Museu Nacional do Rio de Janeiro 1: 3–6.

Miranda-Ribeiro, A. 1923b. *Basanitia lactea* (Um novo batracchio das collecções do Museu Paulista). Revista do Museu Paulista. São Paulo 13: 851–852.

Miranda-Ribeiro, A. 1923c. *Elosia* Tsch. e os gêneros correlatos. Revista do Museu Paulista 8: 813─822.

Miranda-Ribeiro, A. 1923d. Observações sobre algumas phases evolutivas de *Ceratophrys* e *Stombus*. Arquivos do Museu Nacional 24: 201─205.

Miranda-Ribeiro, A. 1923e. Os hylodideos do Museu Paulista. Revista do Museu Paulista. São Paulo 13: 825–846 (reprint pagination 3–24).

Miranda-Ribeiro, A. 1924. De Batrachorum generos specibusque duobus in Collectio Musei Nationalis Servatis. Boletim do Museu Nacional do Rio de Janeiro 1: 255–257.

Miranda-Ribeiro, A. 1926. Notas para servirem ao estudo dos gymnobatrachios (Anura) brasileiros. Arquivos do Museu Nacional 27: 1─227.

Miranda-Ribeiro, A. 1937a. Alguns batrachios novos das colleçcões do Museo Nacional. O Campo 8: 66–69.

Miranda-Ribeiro, A. 1937b. Especies novas do genero *Stombus* da serie de appendices oculares reduzidos. O Campo 1937: 24.

Miranda-Ribeiro, A. 1937c. Sobre uma collecção de vertebrados do nordeste brasileiro. Primeira parte: peixes e batrachios. O Campo 1937: 54–56.

Mocquard, F. 1904. Description de quelques reptiles et d'un batracien nouveaux de la collection du Múseum. Bulletin du Museum National d'Histoire Naturelle. Paris 10: 301–309.

Morais, A. R.; Kwet, A. 2012. Description of the advertisement call of *Physalaemus lisei* (Anura: Leiuperidae). Salamandra 48: 227–229.

Morais, A. R.; Bastos, R. P.; Annunziata, B. B.; Kokubum, M. N. C.; Maciel, A. O. 2012a. Description of the advertisement call of *Rhinella mirandaribeiroi* (Gallardo, 1965) (Anura: Bufonidae). Zootaxa 3265: 66–68.

Morais, A. R.; Batista, V. G.; Gambale, P. G.; Signorelli, L.; Bastos, R. P. 2012. Acoustic communication in a Neotropical frog (*Dendropsophus minutus*): vocal repertoire, variability and individual discrimination. Herpetological Journal 22: 249 257.

Morais, A.R.; Siqueira, M. N.; Bastos, R. P. 2015. How do males of *Hypsiboas goianus* (Hylidae: Anura) respond to conspecific acoustic stimuli? Zoologia 32: 431 437.

Morales, V. R.; McDiarmid, R. W. 2009. A new species of *Chiasmocleis* (Anura: Microhylidae) from southern Amazonian Peru with comments on some other microhylids. Biotempo 9: 71–76.

Morales, V. R. 2002. Sistematica y biogeografía del grupo trilineatus (Amphibia, Anura, Dendrobatidae, Colostethus), con descripción de once nuevas especies. Publicaciones de la Asociación de Amigos de Doñana. Sevilla 13: 1–59.

Moravec, J.; Aparicio, J.; Guerrero-Reinhard, M.; Calderón, G.; Jungfer, K.-H.; Gvoždík, V. 2009a. A new species of *Osteocephalus* (Anura: Hylidae) from Amazonian Bolivia: first evidence of tree frog breeding in fruit capsules of the Brazil nut tree. Zootaxa 2215: 37–54.

Moravec, J.; Tuanama, I. A.; Pérez-Peña, P. E.; Lehr, E. 2009b. A new species of *Scinax* (Anura: Hylidae) from the area of Iquitos, Amazonian Peru. South American Journal of Herpetology 4: 9–16.

Motta, A. P.; Silva, E. T.; Feio, R. N.; Dergam, J. A. 2010. The tadpole of *Leptodactylus cupreus* Caramaschi, Feio & São Pedro, 2008 (Anura, Leptodactylidae). Zootaxa 2640: 65–68.

Moura, M. R.; Lacerda, J. V. A.; Feio, R. N. 2012. The advertisement call of *Haddadus binotatus* (Spix, 1824) (Anura; Craugastoridae). Zootaxa 3224: 67–68.

Mueses-Cisneros, J. J.; Cisneros-Heredia, D. F.; McDiarmid, R. W. 2012. A new Amazonian species of *Rhaebo* (Anura: Bufonidae) with comments on *Rhaebo glaberrimus* (Gunther, 1869) and *Rhaebo guttatus* (Schneider, 1799). Zootaxa 3447: 22–40.

Müller, L.; Hellmich, W. 1936. Amphibien und Reptilien. I. Teil: Amphibia, Chelonia, Loricata. Wissenschaftliche Ergebnisse der Deutschen Gran Chaco-Expedition. Amphibien und Reptilien: 1–120. Stuttgart, Strecker und Schröder.

Müller, F. 1883. Dritter Nachtrag. Katalog der herpetologischen Sammlung des Basler Museums. Basel: J. G. Bauer.

Müller, L. 1914. On a new species of the genus *Pipa* from northern Brazil. Annals and Magazine of Natural History, Series 8, 14: 102.

Müller, L. 1922. Über eine Sammlung Froschlurche von Sta. Catharina nebst Beschriebung zweier neuer Arten. Blätter für Aquarien- und Terrarien-Kunde. Stuttgart 33: 167–171.

Müller, L. 1923. Neue oder seltene Reptilien und Batrachier der zoologischen Sammlung des bayerischen Staates. Zoologischer Anzeiger 57: 38–42.

Müller, L. 1924a. Neue laubfrösche aus dem Staate Santa Catharina, S. O. Brasilien. Zoologischer Anzeiger 59: 233–238.

Müller, L. 1924b. Neue Batrachier aus Ost-Brasilien. Senckenbergiana Biologica 6: 169–177.

2611 Muniz, S.; Moura, C. M.; Moraes, A. A. de M.; Galindo, M. C. F.; Chaves, L. S.;
2612 Kokubum, M. N. de C.; Moura, G. J. B. 2016. Acoustic characteristics of the
2613 advertisement call of *Dendropsophus elegans* (Anura: Hylidae). Herpetology Notes 9:
2614 99 102.

2615 Muramatsu, C. S. Y.; Cruz, C. A. G. 1996. Descrição do girino de *Leptodactylus*
2616 *marambaiae* Izecksohn, 1976 (Amphibia, Anura, Leptodactylidae). Revista da
2617 Universidade Federal Rural do Rio de Janeiro 18: 53─57.

2618 Myers, G. S.; Carvalho, A. L. de. 1945. Notes on some new or little-known Brazilian
2619 amphibians, with an examination of the history of the Plata salamander, Ensatina
2620 platensis. Boletim do Museu Nacional. Nova Serie, Zoologia. Rio de Janeiro 35: 1–24.

2621 Myers, G. S.; Carvalho, A. L. de. 1952. A new dwarf toad from southeastern Brazil.
2622 Zoologica. New York 37: 1–3.

2623 Myers, C. W.; Donnelly, M. A. 1997. A tepui herpetofauna on a granitic mountain
2624 (Tamacuari) in the borderland between Venezuela and Brazil: Report from the Phipps
2625 Tapirapecó Expedition. American Museum Novitates 3213: 1–71.

2626 Myers, C. W.; Donnelly, M. A. 2008. The summit herpetofauna of Auyantepui,
2627 Venezuela: report from the Robert G Goelet American Museum–Terramar Expedition.
2628 Bulletin of the American Museum of Natural History 308: 1–147.

2629 Myers, C. W. 1982. Spotted poison frogs: Descriptions of three new *Dendrobates* from
2630 western Amazonia, and resurrection of a lost species from "Chiriqui". American
2631 Museum Novitates 2721: 1–23.

Myers, C. W.; Rodriguez, L. O.; Icochea, J. 1998. *Epipedobates simulans*, a new cryptic species of poison frog from southeastern Peru, with notes on *E. macero* and *E. petersi* (Dendrobatidae). American Museum Novitates 3238: 1 20.

Nali, R. C.; Prado, C. P. de A. 2014. Complex call with different messages in *Bokermannohyla ibitiguara* (Anura, Hylidae), a Gladiator Frog of the Brazilian Cerrado. Journal of Herpetology 48: 407–414.

Nali, R. C.; Borges, M. M.; Prado, C. P. A. 2015. Advertisement and release calls of *Phyllomedusa ayeaye* (Anura: Hylidae) with comments on the social context of emission. Zoologia 32(4): 263 269.

Napoli, M. F.; Caramaschi, U. 2004. Two new species of the *Hyla circumdata* group from Serra do Mar and Serra da Mantiqueira, southeastern Brazil, with description of the advertisement call of *Hyla ibitipoca* (Anura, Hylidae). Copeia 2004: 534–545.

Napoli, M. F.; Caramaschi, U. 1998. Duas novas espécies de *Hyla* Laurenti, 1768 do Brasil central afins de *H. tritaeniata* Bokermann, 1965. Boletim do Museu Nacional. Nova Serie, Zoologia. Rio de Janeiro 391: 1–12.

Napoli, M. F.; Caramaschi, U. 1999a. Geographic variation of *Hyla ru*bicundula and *Hyla anataliasiasi*, with the description of a new species (Anura, Hylidae). Alytes. Paris 16: 165–189.

Napoli, M. F.; Caramaschi, U. 1999b. Variation and description of two new Brazilian *Hyla* of the *H. tritaeniata* complex (Amphibia, Anura, Hylidae). Boletim do Museu Nacional. Nova Serie, Zoologia. Rio de Janeiro 407: 1–11.

Napoli, M. F.; Caramaschi, U. 2000. Description and variation of a new Brazilian species of the *Hyla rubicundula* group (Anura, Hylidae). Alytes. Paris 17: 165–184.

Napoli, M. F.; Juncá, F. A. 2006. A new species of the *Bokermannohyla circumdata* group (Amphibia: Anura: Hylidae) from Chapada Diamantina, State of Bahia, Brazil. Zootaxa 1244: 57–68.

Napoli, M. F.; Pimenta, B. V. S. 2003. Nova espécie do grupo de *Hyla circumdata* (Cope, 1870) do sul da Bahia, Brasil (Amphibia, Anura, Hylidae). Arquivos do Museu Nacional 61: 189–194.

Napoli, M. F.; Pimenta, B. V. S. 2009. A new species of the *Bokermannohyla circumdata* Group (Anura: Hylidae) from the coastal forests of Bahia, northeastern Brazil. Copeia 2009: 674–683.

Napoli, M. F. 2005. A new species allied to *Hyla circumdata* (Anura: Hylidae) from Serra da Mantiqueira, southeastern Brazil. Herpetologica 61: 63–69.

Napoli, M. F.; Caramaschi, U.; Cruz, C. A. G.; Dias, I. R. 2011a. A new species of flea-toad, genus *Brachycephalus* Fitzinger (Amphibia: Anura: Brachycephalidae), from the Atlantic rainforest of southern Bahia, Brazil. Zootaxa 2739: 33–40.

Napoli, M. F.; Cruz, C. A. G.; Abreu, R. O. de; Del Grande, M. L. 2011b. A new species of *Proceratophrys* Miranda-Ribeiro (Amphibia: Anura: Cycloramphidae) from the Chapada Diamantina, State of Bahia, northeastern Brazil. Zootaxa 3133: 37–49.

Napoli, M. F.; De Abreu, R. O.; Cruz, D.; Herrera, J. B.; Petersen, E.; Klein, W. 2015. Advertisement call of *Dendropsophus studerae* (Carvalho-e-Silva, Carvalho-e-Silva and

2674 Izecksohn, 2003) (Anura: Hylidae), with new record and geographic distribution
2675 extension. Zootaxa 3878: 593 596.

2676 Nascimento, F. A. C.; Skuk, G. O. 2006. O girino de *Chiasmocleis alagoanus* Cruz,
2677 Caramaschi and Freire, 1999 (Anura: Microhylidae). Biota Neotropica 6: 1─5.

2678 Nascimento, F. A. C.; Skuk, G. O. 2007. Description of the tadpole of *Hylomantis*
2679 *granulosa* (Anura: Hylidae). Zootaxa 1663: 59─65.

2680 Nascimento, L. B.; Carvalho Jr., R. R.; Wogel, H.; Fernandes, D. S.; Feio, R. N. 2001a.
2681 Reprodução e descrição do girino de *Physalaemus rupestres* Caramaschi, Carcerelli and
2682 Feio, 1991 (Amphibia, Anura, Leptodactylidae). Boletim do Museu Nacional 450: 1–
2683 10.

2684 Nascimento, L. B.; Pombal, Jr., J. P.; Haddad, C. F. B. 2001a. A new frog of the genus
2685 *Hylodes* (Amphibia: Leptodactylidae) from Minas Gerais, Brazil. Journal of Zoology
2686 254: 421–428.

2687 Nascimento, L. B.; Cruz, C. A. G.; Feio, R. N. 2005. A new species of diurnal frog in
2688 the genus *Crossodactylus* Duméril and Bibron, 1841 (Anura, Leptodactylidae) from
2689 southeastern Brazil. Amphibia-Reptilia 26: 497–505.

2690 Nascimento, F. A. C.; Lima, M. G.; Skuk, G. O.; de Sá, R. O. 2009. The tadpole of
2691 *Hypsiboas atlanti*cus (Anura, Hylidae) from northeastern Brazil. Iheringia 99: 431─436.

2692 Nelson, C. E. 1973. Mating calls of the Microhylinae: descriptions and phylogenetic
2693 and ecological considerations. Herpetologica 29(2): 163–176.

2694 Nelson, C. E. 1975. Another new miniature 4-toed South American microhylid frog
2695 (genus: Syncope). Journal of Herpetology 9: 81–84.

Noble, G. K. 1923. New batrachians from the Tropical Research Station British Guiana. Zoologica. New York 3: 289–299.

Noble, G. K. 1924. Some neotropical batrachians preserved in the United States National Museum with a note on the secondary sexual characters of these and other amphibians. Proceedings of the Biological Society of Washington 37: 65–72.

Nogueira-Costa, P.; Wachlevsi, M. 2015. The tadpole of *Hylodes meridionalis* (Mertens, 1927), a lotic stream anura from the Atlantic Rainforest of Brazil. Zootaxa 4032 (2): 199–202.

Nogueira-Costa, P.; Almeida-Santos, P.; Cruz, C. A. G.; Caramaschi, U. 2012. The giant tadpoles of *Megaelosia jordanensis* (Heyer, 1983). Zootaxa 3581: 86–88.

Nomura, F.; Rossa-Feres, D. C.; Prado, V. H. M. 2003. The tadpole of *Physalaemus fuscomaculatus* (Anura: Leptodactylidae), with a description of internal oral morphology. Zootaxa 370: 1─8.

Novaes, G.; Zina, J. 2016. Advertisement call of *Scinax camposseabrai* (Bokermann, 1968) (Anura: Hylidae), with comments on the call of three species of the *Scinax ruber* clade. Zootaxa 4084: 258 266.

Nunes, I.; Juncá, F. A. 2006. Advertisement calls of three leptodactylid frogs in the state of Bahia, Northeastern Brazil (Amphibia, Anura, Leptodactylidae), with considerations on their taxonomic status. Arquivos do Museu Nacional 64(2): 151 157.

Nunes, I.; Pombal, Jr.; J. P. 2010. A new *Scinax* Wagler (Amphibia, Anura, Hylidae) from the Atlantic rain forest remains of southern State of Bahia, north-eastern Brazil. Amphibia-Reptilia 31: 347–353.

Nunes, I.; Pombal, Jr., J. P. 2011. A new snouted treefrog of the speciose genus *Scinax* Wagler (Anura, Hylidae) from northeastern Brazil. Herpetologica 67: 80–88.

Nunes, I.; Fusinatto, L. A.; Cruz, C. A. G. 2007a. The tadpole and advertisement call of *Sphaenohrynchus palustris* Bokermann, 1966 (Amphibia, Anura, Hylidae). South American Journal of Herpetology 2:123-128.

Nunes, I.; Santiago, R. S.; Juncá, F. A. 2007b. Advertisement calls of four hylid frogs from the State of Bahia, northeastern Brazil (Amphibia, Anura, Hylidae). South American Journal of Herpetology 2(2): 89 96.

Nunes, I.; Canedo, C.; Carvalho, Jr., R. R. 2010. Advertisement call and geographic distribution of *Elachistocleis piauiensis* Caramaschi & Jim, 1983 (Amphibia, Microhylidae), with notes on the presence of post-commissural gland in the genus. South American Journal of Herpetology 5: 30–34.

Nunes, I.; Carvalho, Jr., R. R.; Pereira, E. G. 2010. A new species of *Scinax* Wagler (Anura: Hylidae) from Cerrado of Brazil. Zootaxa 2514: 24–34.

Nunes, I.; Kwet, A.; Pombal, Jr., J. P. 2012. Taxonomic revision of the *Scinax alter* species complex (Anura: Hylidae). Copeia 2012: 554–569.

Nunes, I.; Suárez, P.; Gordo, M.; Pombal, Jr. J. P. 2013. A second species of *Trachycephalus* Tschudi (Anura: Hylidae) with a single vocal sac from the Brazilian Amazon. Copeia 2013: 634–640.

Nunes, I.; Loebmann, D.; Cruz, C. A. G.; Haddad, C. F. B. 2015. Advertisement call, colour variation, natural history, and geographic distribution of *Proceratophrys caramaschii*. Salamandra 51: 103–110.

2740 Nunes-de-Almeida, C. H. L.; Toledo, L. F. 2012. A new species of *Elachistocleis*
2741 Parker (Anura, Microhylidae) from the state of Acre, northern Brazil. Zootaxa 3424:
2742 43–50.

2743 Nunes-de-Almeida, C. H. L.; Zamudio, K. R.; Toledo, L. F. 2016a. The semiterrestrial
2744 tadpole of *Cycloramphus rhyakonastes* Heyer, 1983 (Anura, Cycloramphidae). Journal
2745 of Herpetology 50: 289─294.

2746 Nunes-de-Almeida, C. H. L.; Assis, C. L.; Feio, R. N.; Toledo, L. F. 2016b.
2747 Redescription of the advertisement call of five species of *Thoropa* (Anura,
2748 Cycloramphidae), including recordings of rare and endangered species. Plos One 11:
2749 e0162617.

2750 Oliveira, F. F.; Lírio Jr., G. P. 2000. Anfíbios anuros do Campus da Universidade
2751 Federal de Sergipe. Biologia Geral e Experimental 1(1): 42─74.

2752 Oliveira, L. E.; Oliveira, R. M. C.; Giaretta, A. A. 2008. *Ischnocnema hoehnei*.
2753 Advertisement call. Herpetological Review 39(2): 207 208.

2754 Oliveira, R. M.; Ruas, D. S.; Mendes, C. V. M.; Solé, M. 2014. Advertisement call of
2755 *Rhinella crucifer* (Wied-Neuwied, 1821) (Anura: Bufonidae) from southern Bahia,
2756 Brazil. Zootaxa 3784: 97– 98.

2757 Oliveira-Filho, J. C.; Giaretta, A. A. 2006. Tadpole and advertisement call of
2758 *Chiasmocleis albopunctata* (Anura, Microhylidae) from Brazil. Zootaxa 1353: 63–68.

2759 Oliveira-Filho, J. C.; Giaretta, A. A. 2008. Biologia reprodutiva de *Leptodactylus*
2760 *mystacinus* (Anura, Leptodactylidae) com notas sobre o canto de corte de outras
2761 espécies de *Leptodactylus*. Iheringia 98(4): 508 515.

Oliveria, M. I. R. R.; Weber, L. N.; Ruggeri, J. 2010. The tadpole of *Physalaemus albifrons* (Spix, 1824) (Anura, Leiuperidae). South American Journal of Herpetology 5(3): 249–254.

Orrico, V. G. D.; Carvalho-e-Silva, A. M.; Carvalho-e-Silva, S. P. 2006. Redescription of the advertisement call of *Aplastodiscus arildae* (Cruz & Peixoto) and description of the call of *Aplastodiscus weygoldti* (Cruz & Peixoto) with general notes about the genus in Southeastern Brazil (Anura, Hylidae). Revista Brasileira de Zoologia 23(4): 994 1001.

Orrico, V. G. D.; Mongin, M. M.; Carvalho-e-Silva, A. M. P. T. de. 2007. The tadpole of *Hypsiboas latistriatus* (Caramaschi & Cruz, 2004), a species of the *Hypsiboas polytaenius* (Cope, 1870) clade (Amphibia, Anura, Hylidae). Zootaxa 1531: 25–37.

Orrico, V. G. D.; Peloso, P. L. V.; Sturaro, M. J.; Silva, Filho, H. F.; Neckel-Oliveira, S.; Gordo, M.; Faivovich, J.; Haddad, C. F. B. 2014. A new "Bat-Voiced" species of *Dendropsophus* Fitzinger, 1843 (Anura, Hylidae) from the Amazon Basin, Brazil. Zootaxa 3881: 341–361.

Padial, J. M.; De la Riva, I. 2005. Rediscovery, redescription, and advertisement call of *Eleutherodactylus heterodactylus* (Miranda Ribeiro, 1937) (Anura: Leptodactylidae), and notes on other *Eleutherodactylus*. Journal of Herpetology 39: 372–379.

Padial, J. M.; De la Riva, I. 2009. Integrative taxonomy reveals cryptic Amazonian species of *Pristimantis* (Anura: Strabomantidae). Zoological Journal of the Linnean Society 155: 97–122.

Padial, J. M.; Köhler, J. 2001. First record of *Physalaemus centralis* (Anura: Leptodactylidae) for Bolivia, with description of its advertisement call. Boletín de la Asociación Herpetológica Española 12: 6–8.

Pansonato, A.; Ávila, R. W.; Kawashita-Ribeiro, R. A.; Morais, D. H. 2011. Advertisement call and new distribution records of *Hypsiboas leucocheilus* (Anura: Hylidae). Salamandra 47: 55–58.

Pansonato, A.; Morais, D. H.; Ávila, L. J.; Kawashita-Ribeiro, R. A.; Strüssmann, C.; Martins, I. A. 2012. A new species of *Pseudopaludicola* Miranda-Ribeiro, 1926 (Anura: Leiuperidae) from the state of Mato Grosso, Brazil, with comments on the geographic distribution of *Pseudopaludicola canga* Giaretta & Kokubum, 2003. Zootaxa 3523: 49–58.

Pansonato, A.; Strüssmann, C.; Mudrek, J. R.; Martins, I. A. 2013. Morphometric and bioacoustic data on three species of *Pseudopaludicola* Miranda-Ribeiro, 1926 (Anura: Leptodactylidae: Leiuperinae) described from Chapada dos Guimarães, Mato Grosso, Brazil, with the revalidation of *Pseudopaludicola ameghini* (Cope, 1887). Zootaxa 3620: 147–162.

Pansonato, A.; Mudrek, J. R.; Veiga-Menoncello, A. C. P.; Rossa-Feres, D. C.; Martins, I. A.; Strüssmann, C. 2014. A new species of *Pseudopaludicola* Miranda-Ribeiro, 1926 (Anura: Leptodactylidae: Leiuperinae) from northwestern state of São Paulo, Brazil . Zootaxa 3861: 249–264.

Pansonato, A.; Mudrek, J. R.; Simioni, F.; Martins, I. A.; Strüssmann, C. 2014. Geographical variation in morphological and bioacoustic traits of *Pseudopaludicola mystacalis* (Cope, 1887) and a reassessment of the taxonomic status of

Pseudopaludicola serrana Toledo, 2010 (Anura: Leptodactylidae: Leiuperinae). Advances in Zoology 2014: 1–13.

Pansonato, A.; Veiga-Menoncello, A. C. P.; Mudrek, J. R.; Jansen, M.; Recco-Pimentel, S. M.; Martins, I. A.; Strüssmann, C. 2016. Two new species of *Pseudopaludicola* (Anura: Leptodactylidae: Leiuperinae) from eastern Bolivia and western Brazil. Herpetologica 72: 235–255.

Parker, W. K. 1876. On the structure and development of the skull in the Batrachia. Part II. Philos Trans R Soc Lond 166: 601─669.

Parker, H. W. 1926. A new brachycephalid frog from Brazil. Annals and Magazine of Natural History, Series 9, 18: 201–203.

Parker, H. W. 1927a. A revision of the frogs of the genera *Pseudopaludicola*, *Physalaemus*, and *Pleurodema*. Annals and Magazine of Natural History, Series 9, 20: 450–478.

Parker, H. W. 1927b. The brevicipitid frogs allied to the genus *Hypopachus*. Occasional Papers of the Museum of Zoology, University of Michigan 187: 1–6.

Parker, H. W. 1935. The frogs, lizards and snakes of British Guiana. Proceedings of the Zoological Society of London 1935: 505–530.

Parker, H. W. 1936. A collection of reptiles and amphibians from the Upper Orinoco. Bulletin du Musée Royal d'Histoire Naturelle de Belgique 12: 1–4.

Parker, H. W. 1940. Undescribed anatomical structures and new species of reptiles and amphibians. Annals and Magazine of Natural History, Series 11, 5: 257–274.

Pavan, D.; Narvaes, P.; Rodrigues, M. T. 2001. A new species of Leptodactylid frog from the Atlantic forests of southeastern Brazil, with notes on the status and on the speciation of the *Hylodes* species groups. Papéis Avulsos de Zoologia 41: 407─425.

Pederassi, J.; Lima, M. S.; Caramaschi, U.; Souza, P. S.; Santos, M. C.; Silva, I. C. 2015. Redescription of the advertisement call of *Physalaemus albifrons* (Spix, 1824) (Amphibia, Anura, Leptodactylidae). Zootaxa 3994(3): 449 450.

Peixoto, O. L.; Cruz, C. A. G. 1980. Observações sobre a larva de *Proceratophrys appendiculata* (Günther, 1873) (Amphibia, Anura, Leptodactylidae). Revista Brasileira de Biologia 40: 491─493.

Peixoto, O. L.; Cruz, C. A. G. 1983. Girinos de espécies de *Hyla* do grupo "albomarginata" do sudeste brasileiro (Amphibia, Anura, Hylidae). Arquivos da Universidade Federal Rural do Rio de Janeiro 6: 155─163.

Peixoto, O. L.; Cruz, C. A. G. 1988. Descrição de duas espécies novas do gênero *Phyllodytes* Wagler (Amphibia, Anura, Hylidae). Revista Brasileira de Biologia 48: 265–272.

Peixoto, O. L.; Cruz, C. A. G. 1992. New species of *Hyla* from the "Serra da Mantiqueira, Itatiaia, Rio de Janeiro" State—(Amphibia, Anura, Hylidae) (Nova espécie da Hyla da Serra da Mantiqueira, Itatiaia, Estado do Rio de Janeiro—(Amphibia, Anura, Hylidae)). Memórias do Instituto Oswaldo Cruz 87: 197–200.

Peixoto, O. L.; Gomes, M. d. R. 1999. The tadpole of *Hyla nahdereri* Lutz and Bokermann, 1963. Journal of Herpetology 33: 477─479.

Peixoto, O. L.; Weygoldt, P. 1987. Notes on *Ololygon heyeri* Weygoldt, 1986, from Espirito Santo, Brazil (Amphibia: Salientia: Hylidae). Senckenbergiana Biologica 68: 1–9.

Peixoto, O. L. 1981a. Nova espécie de *Hyla* da Serra dos Órgãos, Estado do Rio de Janeiro, Brasil (Amphibia, Anura, Hylidae). Revista Brasileira de Biologia 41: 515─520.

Peixoto, O. L. 1981b. Notas sobre o girino de *Crossodactylus pintoi* Cochran (Amphibia, Anura, Leptodactylidae). Revista Brasileira de Biologia 41:339-341.

Peixoto, O. L. 1982a. Duas novas especies de *Crossodactylodes* de Santa Tereza, Estado do Espirito Santo, Brasil (Amphibia, Anura, Leptodactylidae). Revista Brasileira de Biologia 42: 619–626.

Peixoto, O. L. 1982b. Observações sobre a larva de *Pleurodema diplolistris* (Peters, 1870) (Amphibia, Anura, Leptodactylidae). Revista Brasileira de Biologia 42: 631─633.

Peixoto, O. L. 1987. Caracterização do grupo "perpusilla" e reavaliação da posição taxonômica de *Ololygon perpusilla perpusilla* e *Ololygon perpusilla v-signata* (Amphibia, Anura, Hylidae). Arquivos da Universidade Federal Rural do Rio de Janeiro 10: 37─49.

Peixoto, O. L. 1988a. Sobre o "status" taxonômico de *Hyla catharinae alcatraz* B. Lutz 1973, com a descrição de uma nova espécie para o grupo perpusilla (Amphibia, Anura, Hylidae). Acta Biologica Leopoldensia 10: 253─267.

Peixoto, O. L. 1988b. Duas novas espécies de Ololygon do grupo "perpusilla" (Amphibia, Anura, Hylidae) Arquivos da Universidade Federal Rural do Rio de Janeiro 11: 27-37.

Peixoto, O. L. 1989. Duas novas espécies de *Ololygon* do grupo perpusilla (Amphibia, Anura, Hylidae). Arquivos de Universidade Federal Rural do Rio de Janeiro 11: 27–37.

Peixoto, O. L. 2002. Uma nova espécie de *Scinax* do grupo "perpusillus" para Santa Tereza, estado do Espírito Santo, Brasil. Boletim do Museu de Biologia Mello Leitão 13: 7─15.

Peixoto, O. L.; Izecksohn, E.; Cruz, C. A. G. 1981. Notas sobre o girino de *Proceratophrys laticeps* Izecksohn and Peixoto (Amphibia, Anura, Leptodactylidae). Revista Brasileira de Biologia 41: 553─555.

Peixoto, O. L.; Cruz, C. A. G.; Izecksohn, E.; Silva, S. P. C. 1984. Notas sobre o girino de *Proceratophrys precrenulata* (Amphibia, Anura, Leotidactylidae). Arquivos da Universidade Federal Rural do Rio de Janeiro 7: 83─86.

Peixoto, O. L.; Caramaschi, U.; Freire, E. M. X. 2003. Two new species of *Phyllodytes* (Anura: Hylidae) from the state of Alagoas, northeastern Brazil. Herpetologica 59: 235–246.

Peixoto, M. A.; Guimarães, C.; Lacerda, J. V. A.; Leal, F.; Rocha, P. C.; Feio, R. N. 2016. Vocal repertoire of *Scinax v-signatus* (Lutz, 1968) (Anura, Hylidae) and comments on bioacoustical synapomorphies for *Scinax perpusillus* species group. Acta Herpetologica 11: 53 57.

Peloso, P. L. V.; Sturaro, M. J. 2008. A new species of narrow-mouthed frog of the genus *Chiasmocleis* Méhelÿ 1904 (Anura, Microhylidae) from the Amazonian rainforest of Brazil. Zootaxa 1947: 39–52.

Peloso, P. L. V.; Faivovich, J.; Grant, T.; Gasparini, J. L.; Haddad, C. F. B. 2012. An extraordinary new species of *Melanophryniscus* (Anura, Bufonidae) from southeastern Brazil. American Museum Novitates 3762: 1–32.

Peloso, P. L. V.; Sturaro, M. J.; Forlani, M. C.; Gaucher, P.; Motta, A. P.; Wheeler, W. C. 2014. Phylogeny, taxonomic revision, and character evolution of the genera *Chiasmocleis* and *Syncope* (Anura, Microhylidae) in Amazonia, with descriptions of three new species. Bulletin of the American Museum of Natural History 136: 1–96.

Peloso, P. L. V.; Orrico, V. G. D.; Haddad, C. F. B.; Lima, Filho, G. R.; Sturaro, M. J. 2016. A new species of Clown Tree Frog, *Dendropsophus leucophyllatus* species group, from Amazonia (Anura, Hylidae). South American Journal of Herpetology 11: 66–80.

Pereira, E. G.; Nascimento, L. B. 2004. Descrição da vocalização e do girino de *Pseudopaludicola mineira* Lobo, 1994, com notas sobre a morfologia de adultos (Amphibia, Anura, Leptodactylidae). Arquivos do Museu Nacional 62: 233─240.

Pérez, L. G.; Heyer, W. R. 1993. Description of the advertisement call and resolution of the systematic status of *Leptodactylus gracilis delattini* Muller, 1968 (Amphibia: Leptodactylidae). Proceedings of the Biological Society of Washington 106(1): 51 56.

Peres, J.; Simon, J. E. 2012. *Physalaemus maximus* Feio, Pombal Jr., and Caramaschi, 1999 (Anura: Leiuperidae): distribution extension and advertisement call. Check List 8: 507–509.

2912 Peres, J.; Simon, J. E. 2011. The advertisement call of *Scinax bolloni* Faivovich,
2913 Gasparini & Haddad, 2010 (Amphibia, Anura: Hylidae). Revista Científica Faesa 7 (1):
2914 47 54.

2915 Pereyra, M. O.; Borteiro, C.; Baldo, D.; Kolenc, F.; Conte, C. E. 2012. Advertisement
2916 call of the closely related species *Scinax aromothyella* Faivovich 2005 and *S. berthae*
2917 (Barrio 1962), with comments on the complex calls in the *S. catharinae* group.
2918 Herpetological Journal 22: 133–137.

2919 Pérez-Peña, P. E.; Chávez, G.; Twomey, E.; Brown, J. L. 2010. Two new species of
2920 *Ranitomeya* (Anura: Dendrobatidae) from eastern Amazonian Peru. Zootaxa 2439: 1–
2921 23.

2922 Peters, W. C. H. 1862. Eine neue Gattung von Laubfröschen Plectromantis, aus
2923 Ecuador. Monatsberichte der Königlichen Preussische Akademie des Wissenschaften zu
2924 Berlin 1862: 232–233.

2925 Peters, W. C. H. 1863. Fernere Mittheilungen über neue Batrachier. Monatsberichte der
2926 Königlichen Preussische Akademie des Wissenschaften zu Berlin 1863: 445–470.

2927 Peters, W. C. H. 1867. Über Fledertheire (Pteropus Gouldii, Rhinolophus Deckenii,
2928 *Vespertilio lobipes*, *Vesperugo Temminckii*) und Amphibien (*Hypsilurus godeffroyi*,
2929 *Lygosoma scutatum*, *Stenostoma narisostre*, *Onychocephalus unguirostris*, *Ahaetulla*
2930 *polylepis*, *Pseudechis scutellatus*, *Hoplobatrachus reinhardtii*, *Hyla coriacea*).
2931 Monatsberichte der Königlichen Preussische Akademie des Wissenschaften zu Berlin
2932 1867: 703–712.

2933 Peters, W. C. H. 1870. Über neue Amphien (*Hemidactylus*, *Urosaura*, *Tropdolepisma*,
2934 *Geophis*, *Uriechis*, *Scaphiophis*, *Hoplocephalus*, *Rana*, *Entomoglossus*, *Cystignathus*,

2935 *Hylodes*, *Arthroleptis*, *Phyllobates*, *Cophomantis*) des Königlich Zoologisch Museum.
2936 Monatsberichte der Königlichen Preussische Akademie des Wissenschaften zu Berlin
2937 1870: 641–652.

2938 Peters, W. C. H. 1871. Über einige Arten der herpetologischen Sammlung des Berliner
2939 zoologischen Museums. Monatsberichte der Königlichen Preussische Akademie des
2940 Wissenschaften zu Berlin 1871: 644–652.

2941 Peters, W. C. H. 1872a. Über die von Spix in Brasilien gesammelten Batrachier des
2942 Königl. Naturalienkabinet zu München. Monatsberichte der Königlichen Preussische
2943 Akademie des Wissenschaften zu Berlin 1872: 196–227.

2944 Peters, W. C. H. 1872b. Über eine Sammlung von Batrachiern aus Neu Freiburg in
2945 Brasilien. Monatsberichte der Königlichen Preussische Akademie des Wissenschaften
2946 zu Berlin 1872: 680–684.

2947 Peters, W. C. H. 1873. Über eine, zwei neue Gattungen enthaltende, Sammlung von
2948 Batrachiern des Hrn. Dr. O. Wucherer aus Bahia, so wie über einge neue oder weniger
2949 bekannte Saurier. Monatsberichte der Königlichen Preussische Akademie des
2950 Wissenschaften zu Berlin 1872: 768–7723 +1 plate.

2951 Pezzuti, T.L.; Leite, F. S. F.; Nomura, F. 2009. The tadpole of *Phyllomedusa itacolomi*
2952 (Anura, Hylidae), with a description of the internal oral morphology. South American
2953 Journal of Herpetology 4: 117─124.

2954 Pezzuti, T. L.; Santos, M. T. T.; Martins, S. V.; Leite, F. S. F.; Garcia, P. C. A.;
2955 Faivovich, J. 2015. The tadpoles of two species of the *Bokermannohyla circumdata*
2956 group (Hylidae, Cophomantini). Zootaxa 4048: 151.

Pezzuti, T. L.; Fernandes, I. R.; Leite, F. S. F.; De Sousa, C. E.; Garcia, P. C. A.; Rossa-Feres, D. 2016. The tadpoles of the neotropical *Scinax catharinae* group (Anura, Hylidae): Ecomorphology and descriptions of two new forms. Zoologischer Anzeiger 261: 22─32.

Philippi, R. A. 1902. Suplemento a los Batraquios Chilenos Descritos en la Historia Física i Política de Chile de don Claudio Gay. Santiago de Chile: Libreria Alemana de Jose Ivens.

Pie, M. R.; Ribeiro, L. F. 2015. A new species of *Brachycephalus* (Anura: Brachycephalidae) from the Quiriri mountain range of southern Brazil. PeerJ 3(e1179): 1–9.

Pimenta, B. V. S.; Caramaschi, U. 2007. New species of toad, genus *Frostius* Cannatella, 1986, from the Atlantic Rain Forest of Bahia, Brazil (Amphibia, Anura, Bufonidae). Zootaxa 1508: 61–68.

Pimenta, B. V. S.; Cruz, C. A. G. 2004. The tadpole and advertisement call of *Physalaemus aguirrei* Bokermann, 1966 (Amphibia, Anura, Leptodactylidae). Amphibia-Reptilia 25: 197–204.

Pimenta, B. V. S.; Cruz, C. A. G.; Silvano, D. L. 2005. A new species of the genus *Physalaemus* Fitzinger, 1826 (Anura, Leptodactylidae) from the Atlantic Rain Forest of southern Bahia, Brazil. Amphibia-Reptilia 26: 201─210.

Pimenta, B. V. S.; Nunes, I.; Cruz, C. A. G. 2007. Notes on the poorly known phyllomedusine frog *Hylomantis aspera* Peters, 1872 (Anura, Hylidae). South American Journal of Herpetology 2(3): 206 214.

2979 Pimenta, B. V. S.; Wachlevski, M.; Cruz, C. A. G. 2008. Morphological and acoustical
2980 variation, geographic distribution, and conservation status of the Spinythumb Frog
2981 *Crossodactylus bokermanni* Caramaschi and Sazima, 1985 (Anura, Hylodidae). Journal
2982 of Herpetology 42: 481–492.

2983 Pimenta, B. V. S.; Napoli, M. F.; Haddad, C. F. B. 2009. A new species of casque-
2984 headed tree frog, genus *Aparasphenodon* Miranda-Ribeiro (Amphibia: Anura: Hylidae),
2985 from the Atlantic Rainforest of southern Bahia, Brazil. Zootaxa 2123: 46–54.

2986 Pimenta, B. V. S.; Cruz, C. A. G.; Caramaschi, U. 2014. Taxonomic review of the
2987 species complex of *Crossodactylus dispar* A. Lutz, 1925 (Anura, Hylodidae). Arquivos
2988 de Zoologia. São Paulo 45: 1–33.

2989 Pimenta, B. V. S.; Caramaschi, U.; Cruz, C. A. G. 2015. Synonymy of *Crossodactylus*
2990 *bokermanni* Caramaschi & Sazima, 1985 with *Crossodactylus trachystomus* (Reinhardt
2991 & Lütken, 1862) and description of a new species from Minas Gerais, Brazil (Anura:
2992 Hylodidae). Zootaxa 3955: 65–82.

2993 Pinheiro, P. D. P.; Pezzuti, T. L.; Garcia, P. C. A. 2012. The tadpole and vocalizations
2994 of *Hypsiboas polytaenius* (Cope, 1870) (Anura, Hylidae, Hylinae). South American
2995 Journal of Herpetology 7: 123 133.

2996 Pinheiro, P. D. P.; Taucce, P. P. G.; Leite, F. S. F.; Garcia, P. C. A. 2014. The
2997 advertisement call of the endemic *Bokermannohyla martinsi* (Bokermann, 1964)
2998 (Anura: Hylidae) from southern Espinhaço Range, southeastern Brazil. Zootaxa 3815:
2999 147–150.

3000 Pinheiro, P. D. P.; Pezzuti, T. L.; Leite, F. S. F.; Garcia, P. C. A.; Haddad, C. F. B.;
3001 Faivovich, J. 2016. A new species of the *Hypsiboas pulchellus* group from the Serra da

3002 Mantiqueira, southeastern Brazil (Amphibia: Anura: Hylidae). Herpetologica 72: 256–
3003 270.

3004 Pinto, R.M., Py-Daniel, S.S., Menin, M. 2013. Redescription of the tadpole of
3005 *Phyllomedusa bicolor* (Anura: Hylidae) from Central Amazonia. South American
3006 Journal of Herpetology, 8(1), 2013, 67–72.

3007 Pirani, R. M.; Manga, S.; Santana, D. J.; Assis, B.; Feio, R. N. 2010. Rediscovery,
3008 distribution extention and natural history notes of *Hylodes babax* Heyer, 1982 (Anura,
3009 Hylodidae) with comments on the biogeographic in southeastern Brazil. South
3010 American Journal of Herpetology 5(2): 83 88.

3011 Pirani, R. M.; Pezzuti, T. L.; Motta, A. P.; Feio, R. N. 2011. The tadpole of *Hylodes*
3012 *babax* Heyer, 1982 (Amphibia, Anura, Hylodidae). Zootaxa 2800: 64–68.

3013 Pombal, Jr., J. P.; Bastos, R. P. 1996. Nova especie de Scinax Wagler, 1830 do Brasil
3014 Central (Amphibia, Anura, Hylidae). Boletim do Museu Nacional 371: 1–11.

3015 Pombal, Jr., J. P.; Bastos, R. P. 1998. Nova espécie de *Hyla* Laurenti, 1768 do centro-
3016 oeste brasileiro e a posição taxonômica de *H. microcephala werneri* Cochran, 1952 e *H.*
3017 *microcephala meridiana* B. Lutz, 1952 (Anura, Hylidae). Boletim do Museu
3018 Nacional 390: 1–13.

3019 Pombal, Jr., J. P.; Bastos, R. P. 2003. Vocalizações de *Scinax perpusillus* (A. Lutz & B.
3020 Lutz) e *S. arduous* Peixoto (Anura, Hylidae), com comentários taxonômicos. Revista
3021 Brasileira de Zoologia 20(4): 607 610.

Pombal, Jr., J. P.; Cruz, C. A. G. 1999. Redescrição de *Eleutherodactylus bolbodactylus* (A. Lutz, 1925) e a posição taxonômica de *E. gehrti* (Miranda-Ribeiro, 1926) (Anura, Leptodactylidae). Boletim do Museu Nacional 404: 1 10.

Pombal, Jr., J. P.; Gasparini, J. L. 2006. A new *Brachycephalus* (Anura: Brachycephalidae) from the Atlantic Rainforest of Espírito Santo, southeastern Brazil. South American Journal of Herpetology 1: 87–93.

Pombal, Jr., J. P.; Gordo, M. 1991. Duas novas espécies de *Hyla* da Floresta Atlântica no estado de São Paulo (Amphibia, Anura). Memórias do Instituto Butantan 53: 135─144.

Pombal, Jr., J. P.; Haddad, C. F. B. 1992. Espécies de *Phyllomedusa* do grupo burmeisteri do Brasil oriental, com descição de uma espécie nova (Amphibia, Hylidae). Revista Brasileira de Biologia 52: 217–229.

Pombal, Jr., J. P.; Haddad, C. F. B. 1993. *Hyla luctuosa*, a new treefrog from southeastern Brazil (Amphibia, Hylidae). Herpetologica 49: 16─21.

Pombal, Jr., J. P.; Haddad, C. F. B. 1999. Frogs of the genus *Paratelmatobius* (Anura: Leptodactylidae) with descriptions of two new species. Copeia 1999: 1014─1026.

Pombal, Jr., J. P.; Izecksohn, E. 2011. Uma nova espécie de *Brachycephalus* (Anura, Brachycephalidae) do estado do Rio de Janeiro. Papeis Avulsos de Zoologia. São Paulo 51: 443-451.

Pombal, Jr., J. P.; Madureira, C. A. 1997. A new species of *Physalaemus* (Anura, Leptodactylidae) from the Atlantic rain forest of northeastern Brazil. Alytes 15: 105─112.

Pombal, Jr., J. P. 1993. New species of *Aparasphenodon* (Anura: Hylidae) from southeastern Brazil. Copeia 1993: 1088–1091.

Pombal, Jr., J. P. 2001. A new species of *Brachycephalus* (Anura: Brachycephalidae) from Atlantic Rain Forest of southeastern Brazil. Amphibia-Reptilia 22: 179–185.

Pombal, Jr., J. P. 2010. O espaço acústico em uma taxocenose de anuros (Amphibia) do Sudeste do Brasil. Arquivos do Museu Nacional 68(1-2): 135 144.

Pombal, Jr., J. P.; Sazima, I.; Haddad, C. F. B. 1994. Breeding behavior of the pumpkin toadlet, *Brachycephalus ephippium* (Brachycephalidae). Journal of Herpetology 28: 516–519.

Pombal, Jr., J. P.; Haddad, C. F. B.; Kasahara, S. 1995. A new species of *Scinax* (Anura: Hylidae) from southeastern Brazil, with comments on the genus. Journal of Herpetology 29: 1–6.

Pombal, Jr., J. P.; Bastos, R. P.; Haddad, C. F. B. 1995. Vocalizações de algumas espécies do gênero *Scinax* (Anura, Hylidae) do sudeste do Brasil e comentários taxonômicos. Naturalia 20: 213 255.

Pombal, Jr., J. P.; Wistuba, E.; Bornschein, M. R. 1998. A new species of brachycephalid (Anura) from the Atlantic rain forest of Brazil. Journal of Herpetology 32: 70–74.

Pombal, Jr., J. P.; Feio, R. N.; Haddad, C. F. B. 2002. A new species of torrent frog genus *Hylodes* (Anura: Leptodactylidae) from southeastern Brazil. Herpetologica 58: 462–471.

Pombal, Jr., J. P.; Haddad, C. F. B.; Cruz, C. A. G. 2003a. New species of *Phrynohyas* from Atlantic rain forest of southeastern Brazil (Anura, Hylidae). Copeia 2003: 379–383.

Pombal, Jr., J. P.; Prado, G. M.; Canedo, C. 2003b. A new species of giant torrent frog, genus *Megaelosia*, from the Atlantic Rain Forest of Espirito Santo, Brazil (Amphibia: Leptodactylidae). Journal of Herpetology 37: 453–460.

Pombal, Jr., J. P.; Siqueira, C. C.; Dorigo, T. A.; Vrcibradic, D.; Rocha, C. F. D. da. 2008. A third species of the rare frog genus *Holoaden* (Terrarana, Strabomantidae) from a montane rainforest area of southeastern Brazil. Zootaxa 1938: 61–68.

Pombal, Jr., J. P.; Carvalho, Jr., R. R.; Canelas, M. A. S.; Bastos, R. P. 2010. A new *Scinax* of the *S. catharinae* species group from central Brazil (Amphibia: Anura: Hylidae). Zoologia 27: 795–802.

Pombal, Jr. P., Jr.; Menezes, V. A.; Fontes, A. F.; Nunes, I.; Rocha, C. F. D. da; Van Sluys, M. 2012. A second species of the casque-headed frog genus *Corythomantis* (Anura: Hylidae) from northeastern Brazil, the distribution of *C. greeningi*, and comments on the genus. Boletim do Museu Nacional 530: 1–14.

Pontes, R.; Mattedi, C.; Baêta, D. 2013. Vocal repertory of *Scinax littoreus* (Anura: Hylidae) with comments on the advertisement call of the *Scinax perpusillus* species group. Zoologia 30(4): 363 370.

Pontes, R. C.; Caramaschi, U.; Pombal, Jr. J. P. 2014. A remarkable new glass frog (Centrolenidae: Vitreorana) from the northeast Atlantic forest, Brazil. Herpetologica 70: 298–308.

Prado, C. P. A.; D'Heursel, A. 2006. The tadpole of *Leptodactylus elenae* (Anura: Leptodactylidae), with the description of the internal buccal anatomy. South American Journal of Herpetology 1: 79─86.

Prado, G. M.; Pombal, Jr., J. P. 2008. Espécies de *Proceratophrys* Miranda-Ribeiro, 1920 com apendices palpebrais (Anura; Cycloramphidae). Arquivos de Zoologia. São Paulo 39: 1–85.

Prado, G. M.; Borgo, J. H.; Abrunhosa, P. A.; Wogel, H. 2003. Comportamento reprodutivo, vocalização e redescrição do girino de *Phrynohyas mesophaea* (Hensel, 1867) do sudeste do Brasil (Amphibia, Anura, Hylidae). Boletim do Museu Nacional do Rio de Janeiro 510: 1 11.

Prigioni, C.; Arrieta, D. 1992. Descripción de la larva de *Melanophryniscus sanmartini* Klappenbach, 1968 (Amphibia, Anura, Bufonidae). Boletín de la Sociedad Zoológica del Uruguay 7: 57─58.

Prigioni, C. M.; Langone, J. A. 2000. Una nueva especie de *Melanophryniscus* Gallardo, 1961, de Argentina y Paraguay (Amphibia, Anura, Bufonidae). Comunicaciones Zoológicas del Museo de Historia Natural de Montevideo 12: 1–11.

Provete, D. B.; Garey, M. V.; Dias, N. Y. N.; Rossa-Feres, D. C. 2011. The Tadpole of *Physalaemus moreirae* (Anura: Leiuperidae). Herpetologica 67: 258─270.

Provete, D. B.; Garey, M. V.; Toledo, L. F.; Nascimento, J.; Lourenço, L. B.; Rossa-Feres, D. C.; Haddad, C. F. B. 2012. Resdescription of *Physalaemus barrioi* (Anura: Leiuperidae). Copeia 2012: 507–518

3108 Provete, D. B.; Melo, L. S. O.; Garey, M. V., Gomes, F. B. R.; Martins, I. A; and Rossa-
3109 Feres, D. C. 2013. The larvae of *Proceratophrys melanopogon* (Amphibia: Anura), with
3110 emphasis on internal oral morphology and comparisons with *P. moratoi* and *P. cururu*.
3111 Herpetologica 69(2): 163─174.

3112 Pugliese, A.; Bastos, R. P. 2001. Description of the tadpole of *Scinax perereca* (Anura,
3113 Hylidae). Amphibia-Reptilia 22(484): 1─4.

3114 Pugliese, A.; Alves, A. C. R.; Carvalho-e-Silva, S. P. 2000. The tadpole of *Hyla*
3115 *oliveirai* and *Hyla decipiens* with notes on the *Hyla microcephala* group (Anura,
3116 Hylidae). Alytes 18: 73─80.

3117 Pugliese, A.; Alves, A. C. R. et al. 2001. The tadpole of *Hyla rubicundula* (Anura,
3118 Hylidae). Journal of Herpetology 35: 686─688.

3119 Pugliese, A.; Pombal, Jr., J. P., Sazima, I. 2004. A new species of *Scinax* (Anura:
3120 Hylidae) from rocky montane fields of the Serra do Cipo, southeastern Brazil. Zootaxa
3121 688: 1–5.

3122 Pugliese, A.; Baêta, D.; Pombal, Jr., J. P. 2009. A new species of *Scinax* (Anura:
3123 Hylidae) from rocky montane fields in southeastern and central Brazil. Zootaxa 2269:
3124 53–64.

3125 Pyburn, W. F.; Glidwell, J. R. 1971. Nests and breeding behavior of *Phyllomedusa*
3126 *hypochondrialis* in Colombia. Journal of Herpetology 5: 49─52.

3127 Pyburn, W. F. 1967. Breeding and larval development of the Hylid frog *Phrynohyas*
3128 *spilomma* in southern Veracruz, México. Herpetologica 23: 184─194.

Pyburn, W. F. 1975. A new species of microhylid frog of the genus *Synapturanus* from southeastern Colombia. Herpetologica 31: 439–443.

Pyburn, W. F. 1977. A new hylid frog (Amphibia, Anura, Hylidae) from the Vaupés River of Colombia with comments on related species. Journal of Herpetology 11: 405–410.

Pyburn, W. F. 1981. A new poison-dart frog (Anura: Dendrobatidae) from the forest of southeastern Colombia. Proceedings of the Biological Society of Washington 94: 67–75.

Pyburn, W. F. 1992. A new tree frog of the genus *Scinax* from the Vaupes River of northwestern Brazil. Texas Journal of Science 44: 405–411.

Rada De Martinez, D. 1990. Contribución al conocimiento de las larvas de anfibios de Venezuela. Memória de la Sociedad de Ciencias Naturales La Salle 49/50: 391─403.

Raddi, G. 1823. Continuazione della descrizione dei rettili Brasiliani. Memorie della Societa Italiana delle Scienze. Modena 19: 58–73.

Recoder, R. S.; Teixeira, Jr.,M.; Cassimiro, J.; Camacho, A.; Rodrigues, M. T. 2010. A new species of *Dendrophryniscus* (Amphibia, Anura, Bufonidae) from the Atlantic Rainforest of southern Bahia, Brazil. Zootaxa 2642: 36–44.

Reinhardt, J. T.; Lütken, C. F. 1862. Bidrag til Kundskab om Brasiliens Padder og Krybdyr. Förste Afdeling: Padderne og Öglerne. Videnskabelige Meddelelser fra Dansk Naturhistorisk Forening i Kjøbenhavn, Serie 2, 3: 143–242.

Ribeiro, L. F.; Alves, A. C. R.; Haddad, C. F. B.; Reis, S. F. dos. 2005. Two new species of *Brachycephalus* Günther, 1858 from the state of Paraná. southern Brazil. Boletim do Museu Nacional 519: 10–18.

Ribeiro, L. F.; Bornschein, M. R.; Belmonte-Lopes, R.; Firkowski, C. R.; Morato, S. A. A.; Pie, M. R. 2015. Seven new microendemic species of *Brachycephalus* (Anura: Brachycephalidae) from southern Brazil. PeerJ 3 (e1011): 1–35.

Rivero, J. A.; Serna, M. A. 1985. Una nueva *Pseudopaludicola* (Amphibia: Leptodactylidae) cornuda del sureste de Colombia. Caribbean Journal of Science 20: 169–171.

Rivero, J. A. 1961. Salientia of Venezuela. Bulletin of the Museum of Comparative Zoology 126: 1–207.

Rivero, J. A. 1971. Tres nuevos records y una nueva especies de anfibios de Venezuela. Caribbean Journal of Science 11: 1–9.

Roberto, I. J.; Ávila, R. W. 2013. The advertisement call of *Phyllodytes gyrinaethes* Peixoto, Caramaschi & Freire, 2003 (Anura, Hylidae). Zootaxa 3669: 193–196.

Roberto, I. J.; Brito, L.; Cascon, P. 2011. Temporal and spatial patterns of reproductive activity in *Rhinella hoogmoedi* (Anura: Bufonidae) from a Tropical Rainforest in northeastern Brazil, with the description of it's advertisement call. South American Journal of Herpetology 6(2): 87 97.

Roberto, I. J., D. Cardozo, and R. W. Ávila. 2013. A new species of *Pseudopaludicola* (Anura, Leiuperidae) from western Piauí State, Northeast Brazil. Zootaxa 3636: 348–360.

Roberto, I. J.; Cardozo, D.; Ávila, R. W. 2013. A new species of *Pseudopaludicola* (Anura, Leiuperidae) from western Piauí State, Northeast Brazil. Zootaxa 3636: 348–360.

Roberto, I. J.; Brito, L.; Thomé, M. T. C. 2014. A new species of *Rhinella* (Anura: Bufonidae) from northeastern Brazil. South American Journal of Herpetology 9: 190–199.

Rocha, P. C.; Pezzuti, T. L.; Garcia, P. C. de A. 2016. Advertisement call of *Leptodactylus viridis* (Anura: Leptodactylidae) from Minas Gerais, Brazil. Salamandra 52(2): 342─344.

Rocha, P. C.; Thompson, J. R.; Leite, F. S. F.; Garcia, P. C. A. 2016. The advertisement call of *Bokermannohyla flavopicta* Leite, Pezzuti & Garcia, 2012 (Anura: Hylidae) from the mountains of Chapada Diamantina, Bahia, Brazil. Zootaxa 4061: 277.

Rodrigues, D. J.; Menin, M.; Lima, A. P. 2007. Redescription of the tadpole of *Leptodactylus rhodomystax* (Anura: Leptodactylidae) with natural history notes. Zootaxa 1509: 61─67.

Rodrigues, D. J.; Menin, M.; Lima, A. P.; Mokross, K. S. 2008. Tadpole and vocalizations of *Chiasmocleis hudsoni* (Anura, Microhylidae) in Central Amazonia, Brazil. Zootaxa 1680: 55–58.

Rodríguez, L. O.; Myers, C. W. 1993. A new poison frog from Manu National Park, southeastern Peru (Dendrobatidae, Epipedobates). American Museum Novitates 3068: 1–15.

Rodríguez, L. O. 1994. A new species of the *Eleutherodactylus conspicillatus* group (Leptodactylidae) from Peru, with comments on its call. Alytes 12: 49–63.

Roithmair, M. E. 1994. Male Territoriality and Female Mate Selection in the Dart-Poison Frog *Epipedobates trivittatus* (Dendrobatidae, Anura). Copeia 1994(1): 107 115.

Rojas-Zamora, R. R., V. T. de Carvalho, M. Gordo, R. W. Ávila, I. P. Farias, and T. Hrbek. 2014. A new species of *Amazophrynella* (Anura: Bufonidae) from the southwestern part of the Brazilian Guiana Shield. Zootaxa 3753: 79–95.

Ron, S. R.; Pramuk, J. B. 1999. A new species of *Osteocephalus* (Anura: Hylidae) from amazonian Ecuador and Peru. Herpetologica 55: 433 446.

Ron, S. R.; Venegas, P. J.; Toral, E.; Read, M.; Ortiz, D. A.; Manzano, A. L. 2012. Systematics of the *Osteocephalus buckleyi* species complex (Anura, Hylidae) from Ecuador and Peru. ZooKeys 229: 1 52.

Ron, S. R.; Venegas, P. J.; Ortega-Andrade, H. M.; Gagliardi-Urrutia, G.; Salerno, P. 2016. Systematics of Ecnomiohyla tuberculosa with the description of a new species and comments on the taxonomy of *Trachycephalus typhonius* (Anura, Hylidae). ZooKeys 630: 115–154.

Rosa, C. N. 1965. Sistemática e biologia de alguns girinos do Estado de São Paulo. Boletim da Faculdade de Filosofia, Ciências e Letras da USP 287:467-487.

Rossa-Feres, D. C.; Jim, J. 1993. Tadpole of *Physalaemus centralis* (Anura, Leptodactylidae). Copeia 1993: 566─569.

Rossa-Feres, D. C.; Jim, J. 1996. Tadpole of *Odontophrynus moratoi* (Anura, Leptodactylidae). Journal of Herpetology 30: 536─539.

Rossa-Feres, D. C.; Nomura, F. 2006. Characterization and taxonomic key for tadpoles (Amphibia: Anura) from the northwestern region of São Paulo State, Brazil. Biota Neotropica 6: 1─26.

Rosset, S. D.; Baldo, D. 2014. The advertisement call and geographic distribution of *Odontophrynus lavillai* Cei, 1985 (Anura: Odontophrynidae). Zootaxa 3784: 79–83.

Rosset, S. D. 2008. New species of *Odontophrynus* Reinhardt and Lütken 1862 (Anura: Neobatrachia) from Brazil and Uruguay. Journal of Herpetology 42: 134–144.

Ruas, D. S.; Mendes, C. V. M.; Dias, I. R.; Solé, M. 2012a. Description of the advertisement call of *Dendropsophus haddadi* (Bastos and Pombal 1996) (Anura: Hylidae) from southern Bahia, Brazil. Zootaxa 3250: 63–65.

Ruas, D. S.; Mendes, C. V. D. M.; Szpeiter, B. B.; Solé, M. 2012b. The tadpole of *Rhinella crucifer* (WIED-NEUWIED, 1821) (Amphibia: Anura: Bufonidae) from southern Bahia, Brazil. Zootaxa 3299: 66–8.

Ruthven, A. G. 1919. The amphibians of the University of Michigan–Walker Expedition to British Guiana. Occasional Papers of the Museum of Zoology, University of Michigan 69: 1–14.

Sá, R. O.; Grant, T.; Camargo, A.; Heyer, W. R.; Ponssa, M. L.; Stanley, E. 2014. Systematics of the Neotropical genus *Leptodactylus* Fitzinger, 1826 (Anura: Leptodactylidae): Phylogeny, the relevance of non-molecular evidence, and species accounts. South American Journal of Herpetology 9: 1–128.

Sá, F. P.; Canedo, C.; Lyra, M. L.; Haddad, C. F. B. 2015. A new species of *Hylodes* (Anura, Hylodidae) and its secretive underwater breeding behavior. Herpetologica 71: 58–71.

Sá, F. P.; Zina, J.; Haddad, C. F. B. 2016. Sophisticated communication in the Brazilian Torrent Frog *Hylodes japi*. Plos One 11: e0145444.

Salas, N. E.; Zavattieri, M. V.; Di Tada, I. E.; Martino, A. L.; Bridarolli, M. E. 1998. Bioacustical and etho-ecological features in amphibian communities of southern Córdoba province (Argentina). Cuaderno de Herpetología 12: 37 46.

Santana, D. J.; Sant'Anna, A. C.; São-Pedro, V.A.; Feio, R.N. 2009. The advertisement call of *Chiasmocleis bassleri* (Anura, Microhylidae) from southern Amazon, Mato Grosso, Brazil. South American Journal of Herpetology 4: 225–228.

Santana, D. J.; São-Pedro, V. A.; Bernarde, P. S.; Feio, R. N. 2010. Descrição do canto de anúncio e dimorfismo sexual em *Proceratophrys concavitympanum* Giaretta, Bernarde & Kokubum, 2000. Papéis Avulsos de Zoologia 50(11): 167 174.

Santana, D. J.; Mesquita, D. O.; Garda, A. A. 2011a. Advertisement call of *Dendropsophus oliveirai* (Anura, Hylidae). Zootaxa 2997: 67 68.

Santana, D. J.; Rodrigues, R.; Albuquerque, R. L.; Laranjeiras, D. O.; Protázio, A. S.; França, F. G. R.; Mesquita, D. O. 2011b. The advertisement call of *Proceratophrys renalis* (Miranda-Ribeiro, 1920) (Amphibia: Anura: Cycloramphidae). Zootaxa 2809: 67 68.

Santana, D. J.; Fonseca, E. M. da; Neves, M. de O.; Carvalho, R. M. H. de. 2012a. A new species of *Adelophryne* (Anura: Eleutherodactylidae) from the Atlantic forest, southeastern Brazil. Salamandra 48: 187–192.

Santana, D. J.; Motta, A. P.; Pirani, R. M.; Silva, E. T.; Feio, R. N. 2012b. Advertisement call and tadpole of *Chiasmocleis mantiqueira* Cruz, Feio and Cassini, 2007 (Anura, Microhylidae). Journal of Herpetology 46(1): 14─18.

Santana, D. J.; Queiroz, S. S.; Wanderley, P. S.; São-Pedro, V. A.; Leite, F. S. F.; Garda, A. A. 2013. Calls and tadpoles of the species of *Lysapsus* (Anura, Hylidae, Pseudae). Amphibia-Reptilia 34: 201─215.

Santana, D. J.; Barros, A. B.; Pontes, R. C.; Feio, R. N. 2015. A new species of Glassfrog genus *Vitreorana* (Anura, Centrolenidae) from the Cerrado Domain, southeastern Brazil. Herpetologica 71: 289–298.

Santana, D.J.; Magalhães, F. d. M.; Pedro, V. A. S.; Mângia, S.; Amado, T. F.; Garda, A. A. 2016. Calls and tadpoles of the species of *Pseudis* (Anura, Hylidae, Pseudae). Herpetological Journal 26: 139–148.

Santos, C. S.; Alves, A. C. R.; Silva, S. P. C. 1998. Description of the tadpoles of *Hyla giesleri* and *Hyla microps* from Southeastern Brazil. Journal of Herpetology 32(1): 61─66.

Santos, M. T. T.; Pezzuti, T. L.; Leite, F. S. F.; Garcia, P. C. A. 2015. The tadpole of *Chiasmocleis schubarti* Bokermann, 1952 (Amphibia, Anura: Microhylidae). Zootaxa 4000 (1): 137–140.

Santos-Silva, C. R.; Ferrari, S. F.; Juncá, F. A. 2012. Acoustic characteristics of the advertisement call of *Trachycephalus atlas* Bokermann, 1966 (Anura: Hylidae). Zootaxa 3424: 66 68.

São-Pedro, V. A.; Medeiros, P. E.; Garda, A. A. 2011. The advertisement call of *Rhinella granulosa* (Anura, Bufonidae). Zootaxa 3092: 60–62.

Savage, J. M.; Cei, J. M. 1965. A review of the leptodactylid frog genus *Odontophrynus*. Herpetologica 21: 178─195.

Sazima, I.; Bokermann, W. C. A. 1977. Anfíbios da Serra do Cipó, Minas Gerais, Brasil. Observações sobre a biologia de *Hyla alvarengai* Bok. (Anura, Hylidae). Revista Brasileira de Biologia 37: 413─417.

Sazima, I.; Bokermann, W. C. A. 1978. Cinco novas espécies de *Leptodactylus* do centro e sudeste brasileiro (Amphibia, Anura, Leptodactylidae). Revista Brasileira de Biologia 38: 899─912.

Sazima, I.; Bokermann, W. C. A. 1982. Anfíbios da Serra do Cipó, Minas Gerais, Brasil. 5: *Hylodes otavioi* sp.n. (Anura, Leptodactylidae). Revista Brasileira de Biologia 42: 767–771.

Sazima, I.; Caramaschi, U. 1988. Descricao de *Physalaemus deimaticus* sp. n., e observacoaes dobre comportamento deimatico em *P. nattereri* (Steindn.) — Anura. Revista de Biologia. Lisboa 13: 91–101.

Sazima, I.; Cardoso, A. J. 1978. Uma espécie nova de *Eleutherodactylus* do sudeste Brasileiro (Amphibia, Anura, Leptodactylidae). Revista Brasileira de Biologia 38: 921–925.

Sazima, I.; Werner, C.; Bokermann, A. 1977. Anfíbios da Serra do Cipó, Minas Gerais, Brasil. 3: Observações sobre a biologia de *Hyla alvarengai* Bok. (Anura, Hylidae). Revista Brasileira de Biologia 37(2): 413 417

Schiesari, L. C.; Moreira, G. 1996. The tadpole of *Phrynohyas coriacea* (Hylidae) with comments on the species' reproduction. Journal of Herpetology 30: 404─407.

Schiesari, L. S., Grillitsch, B.; Vogl, C. 1996. Comparative morphology of phytotelmonous and pond-dwelling larvae of four neotropical treefrog species (Anura, Hylidae, *Osteocephalus oophagus*, *Osteocephalus taurinus*, *Phrynohyas resinifictrix*, *Phrynohyas venulosa*). Alytes 13(4): 109─139.

Schlüter, A.; Salas, A. W. 1991. Reproduction, tadpoles, and ecological aspects of three syntopic microhylid species from Peru (Amphibia: Mycrohylidae). Stuttgarter Beiträge zur Naturkunde, Serie A 458: 1─17.

Schlüter, A. 1979. Bio-akustische Untersuchungen an Hyliden in einem begrenzten Gebiet des tropischen Regenwaldes von Peru (Amphibia: Salientia: Hylidae). Salamandra 15: 211 236.

Schlüter, A. 1980a. Bio-akustische Untersuchungen an Dendrobatiden in einem begrenzten Gebiet des tropischen Regenwaldes von Peru (Amphibia: Salientia: Dendrobatidae). Salamandra 16: 149–161.

Schlüter, A. 1980b. Bio-akustische Untersuchungen an Leptodactyliden in einem begrenzten Gebiet des tropischen Regenwaldes von Peru (Amphibia: Salientia: Leptodactylidae). Salamandra 16: 227 247.

Schlüter, A. 1981. Bio-akustische Untersuchungen an Bufoniden in einem begrenzten Gebiet des tropischen Regenwaldes von Peru. (Amphibia: Salientia: Bufonidae). Salamandra 17: 99–105.

Schlüter, A. 1990. Reproduction and tadpole of *Edalorhina perezi* (Amphibia, Leptodactylidae). Studies on Neotropical Fauna and Environment 25: 49 56.

Schmidt, K. P. 1944. New frogs from Misiones and Uruguay. Field Museum of Natural History Publication. Zoological Series 29: 153–160.

Schneider, J. G. 1799. Historia Amphibiorum Naturalis et Literarariae. Fasciculus Primus. Continens Ranas, Calamitas, Bufones, Salamandras et Hydros in Genera et Species Descriptos Notisque suis Distinctos. Jena: Friederici Frommanni.

Schneider, H.; Joermann, G.; Hödl, W. 1988. Calling and antiphonal calling in four neotropical anuran species of the family Leptodactylidae. Zoologische Jahrbücher Abteilung für Allgemeine Zoologie und Physiologie der Tiere 92: 77–103.

Shreve, B. 1935. On a new teiid and Amphibia from Panama, Ecuador, and Paraguay. Occasional Papers of the Boston Society of Natural History 8: 209–218.

Schulte, R. 1999. Pfeilgiftfrösche. "Artenteil—Peru". Waiblingen: Karl Hauck.

Schulze, A.; Jansen, M.; Köhler, G. 2015. Tadpole diversity of Bolivia's lowland anuran communities: molecular identification, morphological characterisation, and ecological assignment. Zootaxa 4016: 1.

Schwartz, J. J.; Wells, K. D. 1984. Interspecific acoustic interactions of the Neotropical treefrog *Hyla ebraccata*. Behavioral Ecology and Sociobiology 14: 211–224.

Schwartz, J. J.; Wells, K. D. 1985. Intra- and interspecific vocal behavior of the neotropical treefrog *Hyla microcephala*. Copeia 1985(1): 27 38.

Scott-Birabén, M. T.; Fernandez-Marcinowski, K. 1921. Variaciones locales de caracteres específicos em larvas de anfibios. Anales de la Sociedad Cientifica Argentina 92: 129─144.

Señaris, J. C.; Ayarzagüena, J. 2001. Una nueva especie de rana de cristal del género *Hyalinobatrachium* (Anura: Centrolenidae) del Delta del Río Orinoco, Venezuela. Revista de Biología Tropical 49: 1083–1093.

Señaris, J. C.; Ayarzagüena, J. 2005. Revisión taxonómica de la Familia Centrolenidae (Amphibia; Anura) de Venezuela. Sevilla: Publicaciones del Comité Español del Programa Hombre y Biosfera – Red IberoMaB de la UNESCO. No. 7, 337 p.

Señaris, J. C.; DoNascimiento, C.; Villarreal, O. 2005. A new species of the genus *Oreophrynella* (Anura; Bufonidae) from the Guiana Highlands. Papeis Avulsos de Zoologia. São Paulo 45: 61–67.

Shaw, G. 1802. General Zoology or Systematic Natural History. Volume III, Part 1. Amphibia. London: Thomas Davison.

Sheil, C. A.; Mendelson, III, J. R. 2001. A new species of *Hemiphractus* (Anura: Hylidae: Hemiphractinae), and a redescription of *H. johnsoni*. Herpetologica 57: 189–202.

Signorelli, L.; Morais, A. R.; Vieira, R. R. S.; Bastos, R. P. 2016. Vocalizations of *Hypsiboas goianus* (Lutz, 1968) (Anura: Hylidae) in Central Brazil. Studies on Neotropical Fauna and Environment 51(3): 188─196.

Silva, H. R. da; Alves-Silva, R. 2008. New coastal and insular species of the bromeligenous *Scinax perpusillus* group, from the State of Rio de Janeiro, Brazil (Anura, Hylidae). Zootaxa 1914: 34–44.

Silva, H. R. da; Alves-Silva, R. 2011. A new bromeligenous species of the *Scinax perpusillus* group from the hills of the state of Rio de Janeiro, Brazil (Anura, Hylidae). Zootaxa 3043: 54–68.

Silva, H. R. da; Benmaman, P. 2008. Uma nova especie de *Hylodes* Fitzinger da Serra da Mantiqueira, Minas Gerais, Brasil (Anura: Hylodidae). Revista Brasileira de Zoologia 25: 89–99.

Silva, H. R. da; Ouvernay, D. 2012. A new species of stream-dwelling frog of the genus *Cycloramphus* (Anura, Cycloramphidae) from the state of Rio de Janeiro, Brazil. Zootaxa 3407: 49–60.

Silva, R.; Martins, I. A.; Rossa-Feres, D. C. 2008. Bioacústica e sítio de vocalização em taxocenoses de anuros de área aberta no noroeste paulista. Biota Neotropica 8: 123–134.

Silva-Filho, I. S. N.; Juncá, F. A. 2006. Evidence of full species status of the neotropical leaf-frog *Phyllomedusa burmeisteri bahiana* (A. Lutz, 1925) (Amphibia, Anura, Hylidae). Zootaxa 1113: 51 64.

Silva-Soares, T.; Costa, P. N.; Ferreira, R. B.; Weber, L. N. 2010. The tadpole of the hylid frog *Scinax belloni* (Anura: Hylidae). Zootaxa 2727: 63─68.

Silva-Soares, T.; Nogueira-Costa, P.; Borges Jr., V. N. T.; Weber, L.N.; Rocha, C. F. D. 2015. The Larva of *Crossodactylus aeneus* Müller, 1924: Morphology and Ecological Aspects. Herpetologica 71(1): 46─57.

3384 Silverstone, P. A. 1976. A revision of the poison-arrow frogs of the genus *Phyllobates*
3385 Bibron in Sagra (family Dendrobatidae). Science Bulletin. Natural History Museum of
3386 Los Angeles County 27: 1–53.

3387 Simmons, A. M. 2004. Call recognition in the bullfrog, *Rana catesbeiana*:
3388 Generalization along the duration continuum. Journal of the Acoustical Society of
3389 America 115(3): 1345–1355.

3390 Simões, P. I.; Lima, A. P. 2011. The complex advertisement calls of *Allobates myersi*
3391 (Pyburn, 1981) (Anura: Aromobatidae) from São Gabriel da Cachoeira, Brazil. Zootaxa,
3392 2988: 66–68.

3393 Simões, P. I.; Lima, A.P. 2012. The tadpole of *Allobates sumtuosus* (Morales, '2000'
3394 2002) (Anura: Aromobatidae) from its type locality at Reserva Biológica do Rio
3395 Trombetas, Pará, Brazil. Zootaxa 3499: 86–88.

3396 Simões, P. I. 2016. A new species of nurse-frog (Aromobatidae, Allobates) from the
3397 Madeira River basin with a small geographic range. Zootaxa 4083: 501–525.

3398 Simões, P. I.; Lima, A. P.; Farias, I. P. 2010. The description of a cryptic species related
3399 to the pan-Amazonian frog *Allobates femoralis* (Boulenger 1883) (Anura:
3400 Aromobatidae). Zootaxa 2406: 1–28.

3401 Simões, P. I.; Sturaro, M. J.; Peloso, P. L. V.; Lima, A. P. 2013. A new diminutive
3402 species of *Allobates* Zimmermann and Zimmermann, 1988 (Anura, Aromobatidae) from
3403 the northwestern Rio Madeira—Rio Tapajós interfluve, Amazonas, Brazil. Zootaxa
3404 3609: 251–273.

3405 Simões, P. I.; Kaefer, Í. L.; Farias, I. P.; Lima, A. P. 2013. An integrative appraisal of
3406 the diagnosis and distribution of *Allobates sumtuosus* (Morales, 2002) (Anura,
3407 Aromobatidae) . Zootaxa 3746: 401–421.

3408 Simon, J. E.; Gasparini, J. L. 2003. Descrição da vocalização de *Phyllodytes kautskyi*
3409 Peixoto e Cruz, 1988 (Amphibia, Anura, Hylidae). Boletim do Museu de Biologia
3410 Mello Leitão 16: 47 54.

3411 Sinsch, U.; Juraske, N. 2006. Advertisement calls of hemiphractine marsupial frogs: III
3412 *Flectonotus* spp. Proceedings of the 13th Congress of Societas Europaea Herpetologica
3413 153 157.

3414 Smith, E. N.; Noonan,B. P. 2001. A new species of *Osteocephalus* (Anura: Hylidae)
3415 from Guyana. Revista de Biología Tropical. San José 49: 347–357.

3416 Sokol, O. M. 1977. The free swimming *Pipa* larvae, with a review of pipid phylogeny
3417 (Anura: Pipidae). Journal of Morphology 154: 357–426.

3418 Souza, M. B.; Haddad, C. F. B. 2003. Redescription and reevaluation of the generic
3419 status of *Leptodactylus dantasi* (Amphibia, Anura, Leptodactylidae), and description of
3420 its unusual advertisement call. Journal of Herpetology 37: 490–497.

3421 Spix, J. B. v. 1824. Animalia nova sive Species novae Testudinum et Ranarum quas in
3422 itinere per Brasiliam annis MDCCCXVII–MDCCCXX jussu et auspiciis Maximiliani
3423 Josephi I. Bavariae Regis. München: F. S. Hübschmann.

3424 Steffen, G. A. 1815. De Ranis nonnullis Observationes Anatomicae quas Consensu
3425 Gratiosae Facultatis Medicae. Berlin: Joannis Friderici Starckii.

Steinbach-Padilha, G. C. 2009. A new species of Melanophryniscus (Anura, Bufonidae) from the Campos Gerais region of southern Brazil. Phyllomedusa. Belo Horizonte 7: 99–108.

Steindachner, F. 1862. Über zwei noch unbeschriebene Batrachier aus des Sammlung des K. K. zoologischn Museum zu Wien. Archivio per La Zoologia L"Anatomia E La Fisiologia, Genova, Fasciolo 1, 2: 77–82.

Steindachner, F. 1863. Über einige neue Batrachier aus den Sammlungen des Wiener Museums. Sitzungsberichte der Kaiserlichen Akademie der Wissenschaften, Mathematisch-Naturwissenschaftliche Classe 48: 186–192.

Steindachner, F. 1864. Batrachologische Mittheilungen. Verhandlungen des Zoologisch-Botanischen Vereins in Wien 14: 239–288.

Steindachner, F. 1867. Reise der österreichischen Fregatte Novara um die Erde in den Jahren 1857, 1858, 1859 unter den Bafehlen des Commodore B. von Wüllerstorf-Urbair. Zologischer Theil. 1. Amphibien. Wien: K. K. Hof- und Staatsdruckerei.

Stevaux, M. N. 2002. A new species of *Bufo* Laurenti (Anura, Bufonidae) from northeastern Brazil. Revista Brasileira de Zoologia 19: 235–242.

Straughan, I. R.; Heyer, W. R. 1976. A functional analysis of the mating calls of the Neotropical frog genera of the *Leptodactylus* complex (Amphibia, Leptodactylidae). Papéis Avulsos de Zoologia 29: 221 245.

Strüssmann, C.; Pansonato, A.; Soares, D. 2011. Vocalização de anúncio e ampliação da distribuição de *Pristimantis crepitans* (Bokermann) (Amphibia, Anura, Strabomantidae). Iheringia 101(4): 296 303.

Sturaro, M. J.; Peloso, P. L. V. 2014. A new species of *Scinax* Wagler, 1830 (Anura: Hylidae) from the Middle Amazon River Basin, Brazil. Papéis Avulsos de Zoologia. São Paulo 54: 9–23.

Suárez-Mayorga, Á. M.; Lynch, J. D. 2001. Los renacuajos colombianos de *Sphaenorhynchus* (Hylidae): descripciones, anotaciones sistemáticas y ecológicas. Revista de la Academia Colombiana de Ciências Exactas, Físicas y Naturales 25: 411─420.

Tárano, Z. 2010. Advertisement calls and calling habitats of frogs from a flooded savanna of Venezuela. South American Journal of Herpetology 5(3): 221–240.

Targino, M., P. N. da Costa; Carvalho-e-Silva, S. P. de. 2009. Two new species of the *Ischnocnema lactea* species series from Itatiaia highlands, southeastern Brazil (Amphibia, Anura, Brachycephalidae). South American Journal of Herpetology 4: 139–150.

Taucce, P. P. G.; Leite, F. S. F.; Santos, P. S.; Feio, R. N.; Garcia, P. C. A. 2012. The advertisement call, color patt erns and distribution of *Ischnocnema izecksohni* (Caramaschi and Kisteumacher, 1989) (Anura, Brachycephalidae). Papeis Avulsos de Zoologia 52: 111–119.

Taucce, P. P. G.; Pinheiro, P. D. P.; Leite, F. S. F.; Garcia, P. C. A. 2015. Advertisement call and morphological variation of the poorly known and endemic *Bokermannohyla juiju* Faivovich, Lugli, Lourenço and Haddad, 2009 (Anura: Hylidae) from central Bahia, Brazil . Zootaxa 3915: 99–110.

Teixeira, B. F. V.; Giaretta, A. A. 2015. Setting a fundament for taxonomy: advertisement calls from the type localities of three species of the *Dendropsophus rubicundulus* group (Anura: Hylidae). Salamandra 51: 137–146.

Teixeira, B. F. da V.; Giaretta, A. A. 2016. Rediscovery of *Dendropsophus tintinnabulum* (Anura: Hylidae) in the upper Rio Negro Drainage (Amazonas, Brazil), with a description of its advertisement call and external morphology. Phyllomedusa 15(2):119–126.

Teixeira, Jr., M.; Amaro, R. C.; Recoder, R. S.; Sena, M. A.; Rodrigues, M. T. 2012a. A relict new species of *Oreobates* (Anura, Strabomantidae) from the seasonally dry tropical forests of Minas Gerais, Brazil, and its implication to the biogeography of the genus and that of South American dry forests. Zootaxa 3158: 37–52.

Teixeira, Jr., M.; Amaro, R. C.; Recoder, R. S.; Vechio, F. D.; Rodrigues, M. T. 2012b. A new dwarf species of *Proceratophrys* Miranda-Ribeiro, 1920 (Anura, Cycloramphidae) from the highlands of Chapada Diamantina, Bahia, Brazil. Zootaxa 3551: 25–42.

Teixeira, Jr., M.; Vechio, F. D.; Recoder, R. S.; Carnaval, A. C. O. Q.; Strangas, M.; Damasceno, R. P.; Sena, M. A. de; Rodrigues, M. T. 2012c. Two new species of marsupial tree-frogs genus *Gastrotheca* Fitzinger, 1843 (Anura, Hemiphractidae) from the Brazilian Atlantic Forest. Zootaxa 3437: 1–23.

Teixeira, B. F. V.; Giaretta, A. A.; Pansonato, A. 2013. The advertisement call of *Dendropsophus tritaeniatus* (Bokermann, 1965) (Anura: Hylidae). Zootaxa 3669: 189–192.

Teixeira, Jr., M.; Recoder, R. S.; Amaro, R. C.; Damasceno, R. P.; Cassimiro, J.; Rodrigues, M. T. 2013b. A new *Crossodactylodes* Cochran, 1938 (Anura: Leptodactylidae: Paratelmatobiinae) from the highlands of the Atlantic Forests of southern Bahia, Brazil . Zootaxa 3702: 459–472.

Teixeira, B. F. da V.; Zaracho,V. H.; Giaretta, A. A. 2016. Advertisement and courtship calls of *Dendropsophus nanus* (Boulenger, 1889) (Anura: Hylidae) from its type locality (Resistencia, Argentina). Biota Neotropica 16(4): e20160183.

Tessarolo, G.; Maciel, N. M.; Morais, A. R.; Bastos, R. P. 2016. Geographic variation in advertisement calls among populations of *Dendropsophus cruzi* (Anura: Hylidae). Herpetological Journal 26: 221 226.

Toledo, L. F.; Haddad, C. F. B. 2004. Acoustic repertoire and calling behavior of *Scinax fuscomarginatus* (Anura, Hylidae). Journal of Herpetology 39(3): 455 464,.

Toledo, L. F. 2010. A new species of *Elachistocleis* (Anura; Microhylidae) from the Brazilian Amazon. Zootaxa 2496: 63–68.

Toledo, L. F.; Castanho, L. M.; Haddad, C. F. B. 2005. Recognition and distribution of *Leptodactylus mystaceus* (Anura: Leptodactylidae) in the state of São Paulo, Southeastern Brazil. Biota Neotropica 5(1): 57 62.

Toledo, L. F.; Araújo, O. G. S.; Guimarães, L. D.; Lingnau, R.; Haddad, C. F. B. 2007. Visual and acoustic signaling in three species of Brazilian nocturnal tree frogs (Anura, Hylidae). Phyllomedusa 6(1): 61 68.

Toledo, L. F.; Garcia, P. C. A.; Lingnau, R.; Haddad, C. F. B. 2007. A new species of *Sphaenorhynchus* (Anura: Hylidae) from Brazil. Zootaxa 1658: 57–68.

Toledo, L. F.; Siqueira, Jr., S.; Duarte, T. C.; Veiga-Menoncello, A. C. P.; Recco-Pimentel, S. M.; Haddad, C. F. B. 2010. Description of a new species of *Pseudopaludicola* Miranda-Ribeiro, 1926 from the state of São Paulo, southeastern Brazil (Anura, Leiuperidae). Zootaxa 2496: 38–48.

Toledo, L. F.; Loebmann, D.; Haddad, C. F. B. 2010. Revalidation and redescription of *Elachistocleis cesarii* (Miranda-Ribeiro, 1920) (Anura: Microhylidae). Zootaxa 2418: 50–60.

Tolledo, J.; Toledo, L. F. 2010. Tadpole of *Rhinella jimi* (Anura: Bufonidae) with comments on the tadpoles of species of the *Rhinella marina* group. Journal of herpetology 44(3): 480─483.

Tonini, J. F. R.; Forlani, M. C.; Sá, R. O. 2014 A new species of *Chiasmocleis* (Microhylidae, Gastrophyninae) from the Atlandti Forest od Espirito Santo State, Brazil. ZooKeys 428: 109 132.

Troschel, F. H. 1848. Theil 3. Versuch einer Zusammenstellung der Fauna und Flora von Britisch-Guiana. Schomburgk, R. ed., Reisen in Britisch-Guiana in den Jahren 1840–44. Im Auftrage Sr. Majestät des Königs von Preussen ausgeführt: 645–661. Leipzig, J. J. Weber.

Tschudi, J. J. v. 1838. Classification der Batrachier mit Berücksichtigung der fossilen Thiere dieser Abtheilung der Reptilien. Neuchâtel: Petitpierre.

Tsuji-Nishikido, B. M.; Kaefer, Í. L.; Freitas, F. C.; Menin, M.; Lima, A. P. 2012. Significant but not diagnostic: differentiation through morphology and calls in the Amazonian frogs *Allobates nidicola* and *A. masniger*. Herpetological Journal 22: 105–114.

Twomey, E.; Brown, J. L. 2009. Another new species of *Ranitomeya* (Anura: Dendrobatidae) from Amazonian Colombia. Zootaxa 2302: 48–60.

Twomey, E.; Delia, J. R. J.; Castroviejo-Fisher, S. 2014. A review of northern Peruvian glassfrogs (Centrolenidae), with the description of four new remarkable species. Zootaxa 3851: 1–87 .

Valetti, J. A.; Salas, N. E.; Martino, A. L. 2013. Bioacústica del canto de advertencia de *Ceratophrys cranwelli* (Anura: Ceratophrydae). Revista de Biología Tropical 61: 273–280.

Van Lidth de Jeude, T. W. 1904. Reptiles and batrachians from Surinam. Notes from the Leyden Museum 25: 83–94.

Vasconcelos, E. G. de; Giaretta, A. A. 2003. A new species of *Hyla* (Anura: Hylidae) from southeastern Brazil. Revista Española de Herpetología 17: 21–27.

Vasconcelos, J. P.; Sousa, G. L.; Leite, J. R. S. A.; Andrade, E. B. 2014. New record and geographic distribution map of *Physalaemus centralis* (Anura: Leptodactylidae), in Piauí, northeastern Brazil. Herpetol Notes. 7: 325–327.

Vaz-Silva, W.; Maciel, N. M. 2011. A new cryptic species of *Ameerega* (Anura: Dendrobatidae) from Brazilian Cerrado. Zootaxa 2826: 57–68.

Vaz-Silva, W.; Di-Bernardo, M.; Guimarães, L. D.; Bastos, R. P. 2007. Territoriality, agonistic behavior, and vocalization in *Pseudis bolbodactylus* A. Lutz, 1925 (Anura: Hylidae) from Central Brazil. Salamandra 43(1): 35 42.

Vaz-Silva, W.; Valdujo, P. H.; Pombal, Jr., J. P. 2012. New species of the *Rhinella crucifer* group (Anura, Bufonidae) from the Brazilian Cerrado. Zootaxa 3265: 57–65.

Vaz-Silva, W.; Maciel, N. M.; Bastos, R. P.; Pombal, Jr., J. P. 2015. Revealing two new species of the *Rhinella margaritifera* species group (Anura, Bufonidae): An enigmatic taxonomic group of Neotropical toads. Herpetologica 71: 212–222..

Verdade, V. K.; Rodrigues, M. T. 2003. A new species of *Cycloramphus* (Anura, Leptodactylidae) from the Atlantic Forest, Brazil. Herpetologica 59: 513–518.

Verdade, V. K.; Rodrigues, M. T. 2007. Taxonomic review of *Allobates* (Anura, Aromobatidae) from the Atlantic Forest, Brazil. Journal of Herpetology 41: 566─580.

Verdade, V. K.; Rodrigues, M. T.; Cassimiro, J.; Pavan, D.; Liou, N.; Lange, M. C. 2008. Advertisement call, vocal activity, and geographic distribution of *Brachycephalus hermogenesi* (Giaretta and Sawaya, 1998) (Anura, Brachycephalidae). Journal of Herpetology 42(3): 542 549.

Vieira, W. L. S.; Arzabe, C. 2008. Descrição do girino de *Physalaemus cicada* (Anura, Leiuperidae). Iheringia 98: 266─269.

Vieira, W. L. S.; Santana, G. G.; Vieira, K. S. 2007b. Description of the tadpole of *Leptodactylus vastus* (Anura: Leptodactylidae). Zootaxa 1529: 61─68.

Vieira, W. L. S.; Vieira, K. S.; Santana, G. G. 2007a. Description of the tadpoles of *Proceratophrys cristiceps* (Anura: Cycloramphidae, Odontophrynini). Zootaxa 1397:17-24.

Vieira, W. L. S.; Santana, G. G.; Nóbrega, S. C.; Santos, C.; Alves, R. R. N.; Pereira-Filho, G. A. 2009. Description of the tadpoles of *Phyllodytes brevirostris* (Anura: Hylidae). Zootaxa 2119: 66─68.

3579 Vieira, R. R. S.; Guerra, V. B.; Bastos, R. P. 2016. Acoustic communication in two
3580 species of the *Hypsiboas albopuncttus* group (Anura: Hylidae) in sympatry and
3581 allopatry. Zoologia 33: 1 10.

3582 Vigle, G. O.; Goberdhan-Vigle, D. C. I. 1990. A new species of small colorful *Hyla*
3583 from the lowland rainforest of Amazonian Ecuador. Herpetologica 46: 467–473.

3584 Vilaça, T. R. A.; Silva, J. R. S.; Solé, M. 2011. Vocalization and territorial behaviour of
3585 *Phyllomedusa nordestina* Caramaschi, 2006 (Anura: Hylidae) from southern Bahia,
3586 Brazil. Journal of Natural History 45(29-30): 1823 1834.

3587 Vilela, B.; Lisboa, B. S.; Nascimento, F. A. C. 2014. Reproduction of *Agalychnis*
3588 *granulosa* (Cruz, 1989) (Anura: Hylidae). Journal of Natural History 48: 1 9.

3589 Vizotto, L. D. 1967. Desenvolvimento de anuros da região norte-ocidental de Estado de
3590 São Paulo. Tipografia Rio Preto, São José do Rio Preto.

3591 Walker, C. F. 1973. A new genus and species of microhylid frog from Ecuador.
3592 Occasional Papers of the Museum of Natural History, University of Kansas 20: 1–7.

3593 Walker, M.; Lourenço, A. C. C.; Pimenta, B. V. S.; Nascimento, L. B. 2015.
3594 Morphological variation, advertisement call, and tadpoles of *Bokermannohyla nanuzae*
3595 (Bokermann, 1973), and taxonomic status of *B. feioi* (Napoli & Caramaschi, 2004)
3596 (Anura, Hylidae, Cophomantini). Zootaxa 3937: 161–178.

3597 Walker, M., J. L. Gasparini, and C. F. B. Haddad. 2016. A new polymorphic species of
3598 egg-brooding frog of the genus *Fritziana* from southeastern Brazil (Anura:
3599 Hemiphractidae). Salamandra 52: 221–229.

Wandolleck, B. 1907. Einige neue und weniger bekannte Batrachier von Brazilien. Abhandlungen und Berichte des Zoologischen und Anthropologisch-Ethnographischen Museums zu Dresden 11: 1–15.

Wassersug, R.; Heyer ,W. R. 1988. A survey of internal oral features of Leptodactyloid larvae (Amphibia: Anura). Smithsonian Contributions to Zoology 457: 1-97.

Weber, L. N.; Carvalho-e-Silva, S. P. 2001. Descrição da larva de *Physalaemus signifer* (Giradr, 1853) (Amphibia, Anura, Leptodactylidae) e informações sobre a reprodução e a distribuição geográfica. Boletim do Museu Nacional 462: 1─6.

Weber, L. N.; Carvalho-e-Silva, S. P.; Gonzaga, L. P. 2005. The tadpole of *Physalaemus soaresi* Izecksohn, 1965 (Anura: Leptodactylidae), with comments on taxonomy, reproductive behavior, and vocalizations. Zootaxa 1072: 35─42.

Weber, L. N.; Gonzaga, L. P.; Carvalho-e-Silva, S. P. 2005. A new species of *Physalaemus* Fitzinger, 1826 from the lowland Atlantic Forest of Rio de Janeiro state, Brazil (Amphibia, Anura, Leptodactylidae). Arquivos do Museu Nacional 63: 677–684.

Weber, L. N.; Bilate, M.; Procaci, L. S.; Silva, S. P. 2007. Amphibia, Anura, Hylodidae, *Hylodes charadranetes*: distribution extension and notes on advertisement calls. Check List 3: 336–337.

Weber, L. N.; Verdade, V. K.; Salles, R. de O. L.; Fouquet, A.; Carvalho-e-Silva, S. P. de. 2011. A new species of *Cycloramphus* Tschudi (Anura: Cycloramphidae) from the Parque Nacional da Serra dos Órgãos, southeastern Brazil. Zootaxa 2737: 19–33.

Werner, F. 1894. Herpetologische Nova. Zoologischer Anzeiger 17: 410–415.

Werner, F. 1897. Über einige noch unbeschriebene Reptilien und Batrachier. Zoologischer Anzeiger 20: 261–267.

Werner, F. 1899. Beschreibung neuer Reptilien und Batrachier. Zoologischer Anzeiger 22: 479–484.

Werner, F. 1903. Neue Reptilien und Batrachier aus dem naturhistorischen Museum in Brussel. Nebst bemerkungen uber einige andere Arten. Zoologischer Anzeiger 26: 246–253.

Wettstein, O. 1934. Hypopachus parkeri nov. spec. ein neuer Termitenfrosch aus Brasilien. Zoologischer Anzeiger 105: 270–272.

Weygoldt, P.; Carvalho-e-Silva, S. P. 1992. Mating and oviposition in the hylodine frog *Crossodactylus gaudichaudii* (Anura: Leptodactylidae). Amphibia-Reptilia 13: 35–45.

Weygoldt, P.; Peixoto, O. L. 1985. A new species of horned toad (*Proceratophrys*) from Espírito Santo, Brazil (Amphibia: Salientia: Leptodactylidae). Senckenbergiana Biologica 66: 1─8.

Weygoldt, P.; Peixoto, O. L. 1987. *Hyla ruschii* n. sp., a new frog from the Atlantic Forest domain in the state of Espirito Santo, Brazil (Amphibia, Hylidae). Studies on Neotropical Fauna and Environment 22: 237─247.

Weygoldt, P. 1976. Observations on the biology and ethology of *Pipa* (Hemipipa) carvalhoi Mir. RiB. 1937 (Anura, Pipidae). Z Tierpsychol. 40(1): 80 99.

Weygoldt, P. 1981. Beobachtungen zur Fortpflanzungsbiologie von *Phyllodytes luteolus* (Wied 1824) im Terrarium (Amphibia: Salientia: Hylidae). Salamandra 17: 1 11.

Weygoldt, P. 1986. Beobachtungen zur Ökologie und Biologie von Fröschen an einem neotropischen Bergbach. Zoologische Jahrbücher, Abteilung für Systematik, Ökologie und Geographie der Tiere, 113: 429 454.

Wied-Neuwied, M. A. P., Prinz zu. 1821b. Reise nach Brasilien in den Jahren 1815 bis 1817. volume 2. Franfurt a. M.: Henrich Ludwig Brönner.

Wied-Neuwied, M. A. P., Prinz zu. 1824a. Abbildungen zur Naturgeschichte Brasiliens. Heft 7. Weimar: Landes-Industrie-Comptoir.

Wied-Neuwied, M. A. P., Prinz zu. 1824b. Abbildungen zur Naturgeschichte Brasiliens. Heft 8. Weimar: Landes-Industrie-Comptoir.

Wied-Neuwied, M. A. P., Prinz zu. 1824c. Verzeichnis der Amphibien welche in zweiten Bande der Naturgeschichte Brasiliens von Prinz Max von Neuwied werden beschreiben Werden. Isis von Oken 14: 661–673.

Wild, E. R. 1992. The tadpoles of *Hyla fasciata* and *H. allenorum*, with a key to the tadpoles of the *Hyla parviceps* group (Anura: Hylidae). Herpetologica 48: 439–447.

Wild, E. R. 1995. New genus and species of Amazonian microhylid frog with a phylogenetic analysis of New World genera. Copeia 1995: 837–849.

Wogel, H.; Abrunhosa, P. A.; Pombal Jr., J. P. 2000. Girinos de cinco espécies de anuros do sudeste do Brasil (Amphibia: Hylidae, Leptodactylidae, Microhylidae). Boletim do Museu Nacional 427: 1─16.

Wogel, H.; Abrunhosa, P. A.; Pombal, Jr., J.P. 2002. Atividade reprodutiva de *Physalaemus signifer* (Anura, Leptodactylidae) em ambiente temporário. Iheringia 92(2): 57 70.

Wogel, H.; Abrunhosa, P. A.; Pombal, Jr., J. P. 2004a. Vocalizations and aggressive behavior of *Phyllomedusa rohdei* (Anura: Hylidae). Herpetological Review 35(3): 239 243.

Wogel, H.; Abrunhosa, P. A.; Prado, G. M. 2004b. The tadpole of *Chiasmocleis carvalhoi* and the advertisement calls of three species of *Chiasmocleis* (Anura, Microhylidae) from the Atlantic rainforest of southeastern Brazil. Phyllomedusa 3(2): 133 140.

Wogel, H.; Abrunhosa, P. A.; Weber, L. N. 2004c. The tadpole, vocalizations and visual displays of *Hylodes nasus* (Anura: Leptodactylidae). Amphibia-Reptilia 25: 219 227.

Wogel, H.; Weber, L. N.; Abrunhosa, P. A. 2006. The tadpole of the casque-headed frog, *Aparasphenodon brunoi* Miranda-Ribeiro (Anura: Hylidae). South American Journal of Herpetology 1: 54─60.

Zaidan, B. F.; Leite, F.S.F. 2012. Advertisement call of the rare, explosive breeding caatinga horned frog *Ceratophrys joazeirensis* Mercadal de Barrio, 1986 (Anura, Ceratophryidae). Zootaxa 3540: 65–66.

Zank, C.; Bernardo, M.; Lingnau, R.; Colombo, P.; Fusinatto, L. A.; Da Fonte, L. F. M. 2008. Calling activity and agonistic behavior of *Pseudis minuta* Günther, 1858 (Anura, Hylidae, Hylinae) in the Reserva Biológica do Lami, Porto Alegre, Brazil. South American Journal of Herpetology 3: 51 57.

Zaracho, V. H. 2014. Re-Description of the advertisement call of *Vitreorana uranoscopa* (Müller, 1924) (Anura, Centrolenidae) from the Argentinean Atlantic Forest, with Notes on Natural History. South American Journal of Herpetology 9: 83–89.

Zaracho, V. H.; Céspedez, J. A.; Alvarez, B. B. 2003. Descripcion de caracteres morfológicos em larvas prometamorficas de *Physalaemus biligonigerus* (Anura, eptodactylidae). Facena 19: 97─108.

Zimmerman, B. L.; Bogart, J. P. 1984. Vocalizations of primary forest frog species in the Central Amazon. Acta Amazonica 14(3-4): 473 520.

Zimmerman, B. L.; Bogart, J. P. 1988. Ecology and calls of four species of Amazonian forest frogs. Journal of Herpetology 22: 97–108.

Zimmerman, B.; Höld, W. 1983. Distinction of *Phrynohyas resinifictrix* (GOELDI, 1907) from Phrynohyas venulosa (LAURENTI, 1786) based on acoustical and behavioural parameters (Amphibia, Anura, Hylidae). Zoologischer Anzeiger 211: 341 352.

Zimmerman, B. L. 1983. A comparison of structural features of calls of open and forest habitat frog species in the central Amazon. Herpetologica 39(3): 235 246.

Zimmermann, H.; Zimmermann, E. 1988. Etho-Taxonomie und zoogeographische Artengruppenbildung bei Pfeilgiftfroschen (Anura: Dendrobatidae). Salamandra 24: 125–160.

Zina, J.; Haddad, C. F. B. 2005. Reproductive activity and vocalizations of *Leptodactylus labyrinthicus* (Anura: Leptodactylidae) in southeastern Brazil. Biota Neotropica 5(2): 119 129.

Zina, J.; Haddad, C. F. B. 2006. Acoustic repertoire of Aplastodiscus arildae and *A. leucopygius* (Anura: Hylidae) in Serra do Japi, Brazil. South American Journal of Herpetology 1(3): 227 236.

3709 Zweifel, R. G.; Myers, C. W. 1989. A new frog of the genus *Ctenophryne*
3710 (Microhylidae) from the Pacific lowlands of northwestern South America. American
3711 Museum Novitates 2947: 1–16.

3712 Zweifel, R.G. 1986. A new genus and species of microhylid frog from the Cerro de la
3713 Neblina region of Venezuela and a discussion of relationships among New World
3714 microhylid genera. American Museum Novitates: 1-24.
